# Microwave-assisted multi-component green synthesis of bioactive pyrazol-5-ol and its derivatives using graphene oxide as a recyclable catalyst: a route to EGFR inhibitors

**DOI:** 10.1039/d5ra06014a

**Published:** 2025-11-10

**Authors:** Dhruvi Chaudhari, Sarmita Jana, Vijay M. Khedkar, Sneha Nair, Kushan Parikh, Shanta Raj Lakshmi

**Affiliations:** a Department of Chemical Science, Faculty of Applied Sciences, Parul University Vadodara – 391 760 India rajlakshmi.shanta0@gmail.com kamser2057@gmail.com +91-8140299246 +91-9898182990; b Department of Life Sciences, Faculty of Applied Sciences, Parul University Vadodara – 391 760 India; c School of Pharmacy, Vishwakarma University Pune – 411 048 India; d Department of Chemistry, L. J. Institute of Applied Sciences, L. J. University Ahmedabad – 382 210 India

## Abstract

Traditional methods for synthesizing heterocyclic compounds often involve multistep procedures and harsh conditions, leading to environmental concerns and inefficient use of resources. Herein, a sustainable and rapid microwave-assisted multi-component reaction (MCR) strategy was developed for the synthesis of 3-methyl-4-(2-nitro-1-phenylethyl)-1*H*-pyrazol-5-ol (4) using graphene oxide (GO) as a heterogeneous catalyst in various polar solvents. Under optimized conditions (180 W, 4 min, 0.05 wt% GO in water), the reaction afforded up to 95% yield. GO, synthesized *via* a modified Hummers' method, exhibited excellent catalytic efficiency and reusability over five consecutive cycles with minimal loss of activity. Structural analyses (XRD, XPS, Raman, FT-IR, TGA, and TEM) revealed that GO retained its nanoscale flake-like morphology (∼5–9 nm crystallite size), few-layered sheet structure, and partially ordered graphitic domains even after repeated microwave exposure, confirming its thermal and structural stability. The optimized protocol efficiently accommodated various substituted reactants, yielding pyrazol-5-ol derivatives (4, 6 and 8 series) in the range of 80–96%. Computational docking of all synthesized compounds against EGFR tyrosine kinase (PDB ID: 1M17) showed favourable π–π stacking and hydrogen bonding interactions, while compound 6a exhibited the strongest binding affinity and potent cytotoxicity toward human lung cancer (A549) cells (IC_50_ = 15.29 μM). This green, fast, and reusable GO-catalysed MCR approach offers a promising route for the sustainable development of EGFR-targeted anticancer agents.

## Abstract 2

GO-catalyzed aqueous synthesis of pyrazol-5-ol derivatives under microwave irritation yielded potent EGFR inhibitors, with strong A549 cytotoxicity.

## Graphical abstract

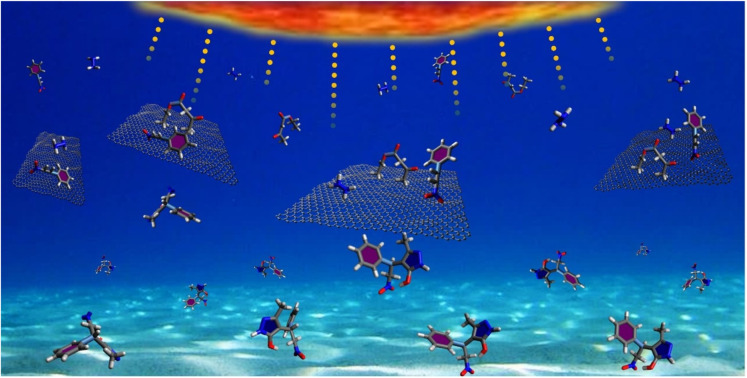

## 1. Introduction

**Fig. 1:**
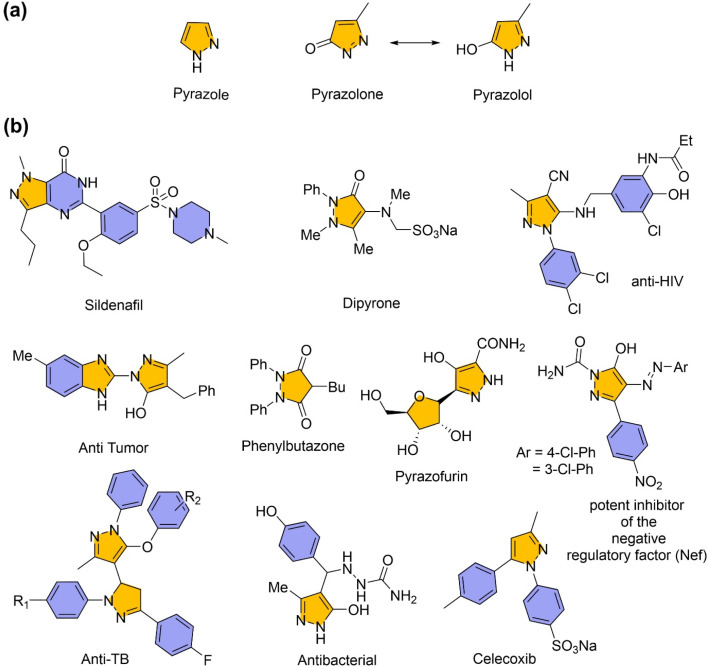
Representative structures of (a) common scaffolds and (b) selected bioactive derivatives, of pyrazole, pyrazolone and pyrazolol.

Multi-component reactions (MCRs) have drawn considerable interest as powerful synthetic strategies for generating molecular diversity.^1^ These are extensively utilized in organic synthesis for the efficient construction of structurally complex molecules.^2^ MCRs closely align with the principles of green chemistry, offering several advantages including high atom economy, operational simplicity, and reduced synthetic steps. By reducing the need for intermediate isolation and purification, these reactions help conserve time, energy, and resources.^3,4^ Among their diverse applications, MCRs play a vital role in the synthesis of nitrogen-containing heterocycles. Such heterocycles, including pyrazole, pyrazolone, and pyrazolol (Fig. 1a), are of significant interest owing to their broad spectrum of biological and pharmacological activities, as well as their synthetic versatility.^5,6^ Several well-known therapeutic agents, such as Sildenafil, Dipyrone, Phenylbutazone, Celecoxib, Pyrazofurin, as well as compounds exhibiting anti-HIV, antitumor, and antituberculosis activities (notably potent inhibitors of the negative regulatory factor, Nef), feature the pyrazole, pyrazolone, and pyrazolol scaffolds (Fig. 1b).

Among these, pyrazolol (Fig. 1a) represents a vital class of heterocyclic compounds with diverse applications spanning agrochemicals, materials science, and pharmaceuticals.^7^ In particular, pyrazol-5-ol and its derivatives constitute an important subclass of these compounds, known for a wide range of biological properties including antimicrobial,^8^ analgesic,^9^ antidiabetic,^10^ antiviral,^11^ anti-inflammatory,^12^ and anticancer activities.^13^

Over the years, synthetic approaches for pyrazol-5-ol derivatives have evolved significantly, each offering distinct advantages and limitations.^14^ Traditional methods typically involve the condensation of hydrazine derivatives with 1,3-dicarbonyl or α, β-unsaturated carbonyl compounds. However, these conventional routes often suffer from harsh reaction conditions, prolonged reaction times, the use of toxic or expensive solvents, non-recyclable catalysts, and low product yields.^15,16^ To overcome these challenges, several green and sustainable methodologies have been developed, improving reaction efficiency and selectivity while reducing environmental impact. These include the use of deep eutectic solvents (DES),^17^ microwave irradiation,^18^ ultrasonication,^19^ mechano-chemical synthesis,^20^ photo-chemical,^21^ electro-chemical,^22^ and ionic liquid-based strategies.^23^ Such approaches embody the principles of green chemistry, emphasizing atom economy, energy efficiency, reduced time and waste, and the use of renewable resources.^24–26^ Consequently, numerous methodologies for synthesizing pyrazol-5-ol and its derivatives have been reported over the past two decades, employing diverse catalyst (amine thiourea, K_2_CO_3_, *etc.*), solvents (H_2_O, toluene, xylene, CHCl_3_, acetone, DES, ionic liquids, *etc.*), methodology (microwave, ultrasonication, ball milling, *etc.*) and reaction conditions to enhance efficiency with reduction in time, selectivity and sustainability.^27–36^ Among all, microwave offer rapid, energy-efficient, and high-yielding routes for organic reactions, particularly heterocyclic scaffolds, enabling novel transformations that are difficult under conventional reaction methods.^36^

In recent years, nanoscience has opened new avenues for developing innovative systems, with significant emphasis on heterogeneous catalysts in organic synthesis that enable, efficient, selective, and sustainable transformations.^37–40^ Carbon-based nanomaterials such as activated carbon, carbon nanotubes, carbon nanofibers, and particularly graphene oxide (GO) have emerged as promising heterogeneous catalyst due to their low cost, high surface area, and remarkable stability.^41^ Graphene and GO, composed of single-layered two-dimensional carbon atoms, exhibit exceptional thermal and electrical conductivity, mechanical strength, and biocompatibility.^42–44^ Notably, GO's hydrophilicity and dispersibility in polar solvents make it highly suitable for various applications, including catalysis, energy storage, water purification, and biomedicine.^45,46^ During the last decade, GO has been effectively employed as a metal-free, environmentally benign catalyst for the synthesis of heterocycles, including pyrazol-5-ol derivatives, in both polar and non-polar solvents under reflux conditions.^47–50^ However, based on the literature survey, the use of GO as a catalyst in an aqueous medium employing green methodology (such as microwave irradiation) has not yet been reported for the synthesis of pyrazol-5-ol derivatives.

Cancer remains one of the most complex and life-threatening diseases, characterized by the uncontrolled proliferation of abnormal cells.^51–53^ The continuous search for new anticancer agents, particularly for human lung carcinoma (A549) cells, remains an urgent global challenge. Understanding the structure–activity relationship (SAR) is crucial in the rational design of anticancer drugs. Among known therapeutic targets, protein kinases rank second only to G protein-coupled receptors (GPCRs) in importance, owing to their role in cancer progression.^54,55^ In particular, the epidermal growth factor receptor (EGFR), a receptor tyrosine kinase, plays a central role in regulating cell proliferation and survival through ligand-induced dimerization and autophosphorylation, activating downstream signalling pathways.^56–58^ Dysregulation or overexpression of EGFR is strongly associated with several epithelial cancers,^59^ and EGFR inhibitors are well-established in clinical oncology.^60^ Notably, specific pyrazole-based scaffolds, such as pyrazolo[3,4-*b*]pyridines, pyrazoline hybrids, and 3-methyl-1-phenyl-2-pyrazolin-5-one, have shown broad-spectrum cytotoxic activity across human cancer cell lines, particularly A549.^61–67^ However, molecular docking studies of pyrazol-5-ol derivatives with EGFR (PDB ID: 1M17) remain unexplored, despite evidence of such interactions for pyrazoline analogues (highlighting the potential anticancer agents).^68^

To address this gap, we report the green synthesis and biological evaluation of 3-methyl-4-(2-nitro-1-phenylethyl)-1*H*-pyrazol-5-ol (denoted as pyrazol-5-ol, 4) synthesized *via* condensation reactions of hydrazine hydrate, ethyl acetoacetate, and β-nitrostyrene. The reaction was systematically optimized in various polar solvents (MeOH, EtOH, DCM, DMSO, DMF, H_2_O) both in the presence and absence of graphene oxide (GO, at different wt%) as a catalyst, under reflux or microwave irradiation (180 W). The GO catalyst was synthesized *via* the Hummers' method and its catalytic performance and recyclability (I–V cycles) were evaluated under optimized condition. The structural and surface characteristics of GO, across the catalytic cycles, was comprehensively analysed by XRD, XPS, Raman spectroscopy, TGA, FT-IR and TEM. Furthermore, the optimized methodology was extended to broad range of substrates (4, 6 and 8 series), incorporating hydrazine hydrate/phenylhydrazine, substituted nitrostyrene, and heteroaryl derivatives, to demonstrate the versatility and generality of the developed protocol. To explore the therapeutic potential of these pyrazol-5-ol scaffolds, cytotoxicity assays, *in vitro*, were performed against human lung cancer (A549) cells, and molecular docking studies were conducted with the epidermal growth factor receptor tyrosine kinase (EGFR, PDB ID: 1M17), to elucidate their binding interactions. The combined experimental and computational results will provide valuable insights into the structure–activity relationship (SAR) and contribute to the rational design of next-generation EGFR-targeted anticancer agents.

## 2. Result and discussion

### 2.1. Synthesis and optimization of 3-methyl-4-(2-nitro-1-phenylethyl)-1*H*-pyrazol-5-ol (4)

Driven by the exceptional reactivity and pharmacological relevance of pyrazolol derivatives, the development of efficient and recyclable catalytic methods has gained considerable interest. Previous reports indicate that in the absence of a catalyst, no product formation occurs even after several hours in aqueous media.^28,69^ However, the use of graphene oxide (GO) as a catalyst has shown notable improvements, *e.g.*, GO refluxed in water for 2 hours yielded 72% of the desired product,^70^ while smaller GO loadings (5 mg and 0.02 g) achieved yields of 82% and 80% within 45 and 25 minutes,^48,49^ respectively.

**Scheme 1:**
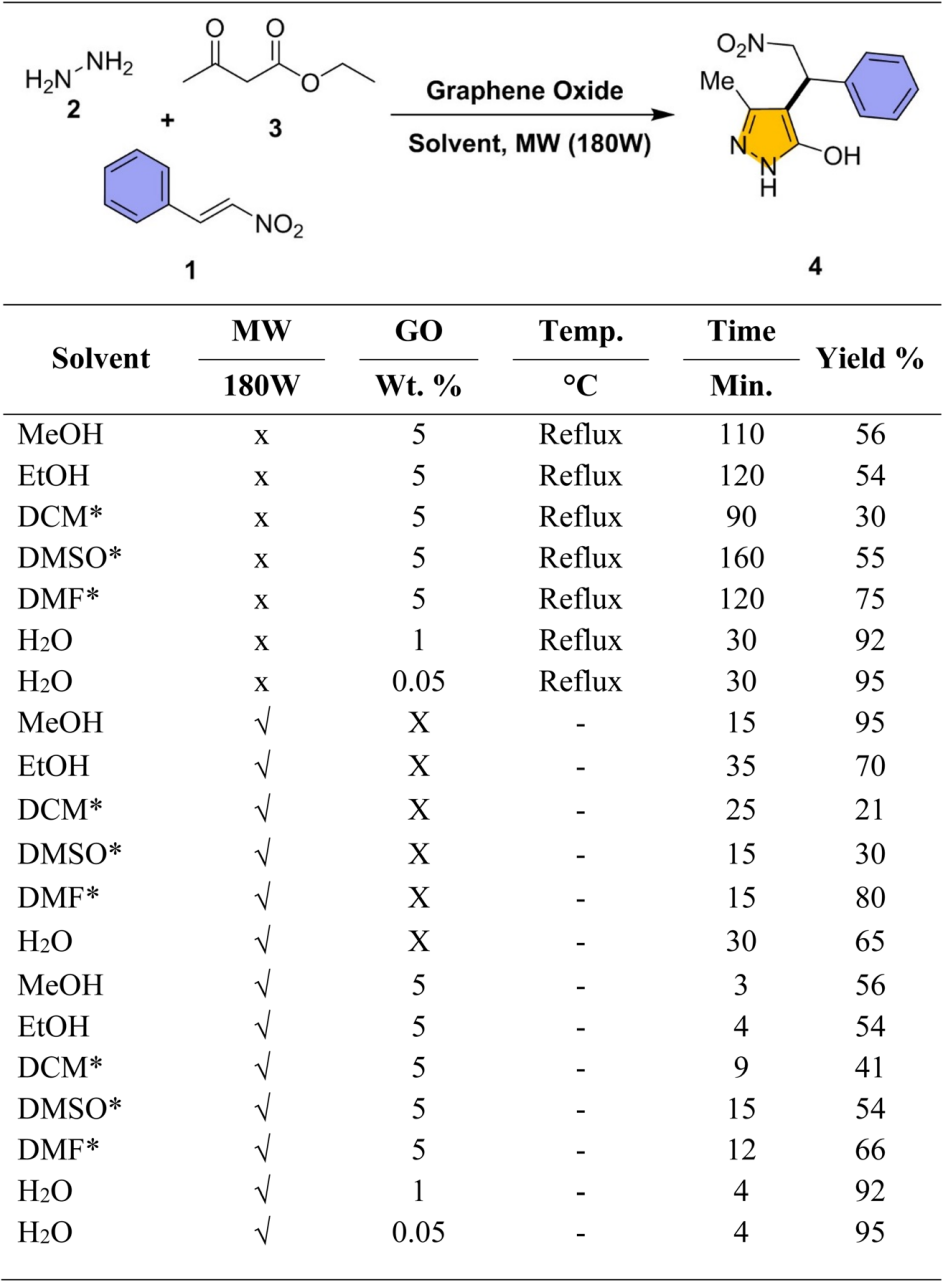
Optimization of pyrazol-5-ol reaction condition on graphene oxide (GO) as catalyst with/without exposure of microwave (MW) in different polar solvents.^*a*^Reaction condition: nitrostyrene 1 (1 eq.), hydrazine hydrate 2 (1 eq.), ethyl acetoacetate 3 (1 eq.), and GO (0–5 wt%) was added in various polar solvent and reflux or exposed with microwave (180 W). *Not clear the solution.

In continuation, we systematically optimized the GO-catalysed synthesis of 3-methyl-4-(2-nitro-1-phenylethyl)-1*H*-pyrazol-5-ol (denoted as pyrazol-5-ol or 4 throughout the article) using stoichiometric amounts of β-nitrostyrene, hydrazine hydrate, and ethyl acetoacetate as multi-component reaction (MCR). Reaction was conducted with GO at different wt% in various polar solvents (MeOH, EtOH, DCM, DMSO, DMF, H_2_O) under reflux or microwave irradiation (180 W) (Scheme 1). These polar solvents were selected to encompass a broad spectrum of dielectric properties, enabling us to evaluate the impact of solvent polarity, hydrogen-bonding capability on the overall reaction outcome. Reaction progress was tracked by TLC and visually indicated by the formation of a precipitate. The structure was confirmed using ^1^H & ^13^C NMR, FT-IR spectroscopy & HR-MS (please see SI for detailed characterization data).

The IR spectrum of the compound 4 displayed a broad adsorption band at 3433 cm^−1^, which was attributed to the stretching vibrations of an –OH or –NH functional group. Characteristic C–H stretching bands were observed at 2923 and 2852 cm^−1^, while, a strong adsorption at 1558 cm^−1^ confirms the presence of a nitro (–NO_2_) group. Furthermore, aromatic C–H out-of-plane bending vibrations at 817, 767, and 743 cm^−1^ provide additional evidence for an aromatic moiety within the molecule. The ^1^H NMR spectrum exhibited aromatic proton signals in the region *δ* 7.40 and 7.23 ppm along with distinct diastereotopic CH_2_–NO_2_ protons resonances at *δ* 5.22 and 4.97 ppm. A benzylic methine proton signal also observed at *δ* 4.62 ppm consistent with the expected structural environment. Collectively, the IR and NMR spectroscopic data confirmed the presence of both aromatic and aliphatic carbons, supporting the proposed structure of pyrazol-5-ol (4).

Notably, under ambient reflux conditions using GO, relatively longer reaction times (30–160 min.) and higher catalyst loadings (5 wt%) were necessary to achieve moderate yields (30–90%). The results are detailed in the corresponding Scheme 1 the accompanying optimization table. For instance, using 5% GO, MeOH and DMSO afforded yields of 56% and 55% in 110 and 160 minutes, respectively, while DMF and EtOH gave 75% and 54% yields after 120 minutes. H_2_O emerged as the most efficient solvent, delivering excellent yields of 92% and 95% within 30 minutes using only 1% and 0.05% GO, respectively. These findings emphasize the catalytic efficiency and potential of GO in green solvent, H_2_O, for efficient heterocyclic synthesis.

To further accelerate and reduce time, the reaction was conducted under microwave irradiation (180 W) in the absence of GO catalyst. The reaction proceeded in similar polar solvents, yielding (21–95%) moderate product outputs with shorter reaction times (15–35 min). Among them, DCM gave the lowest yield (21%) in 25 minutes, while H_2_O improved the yield to 65% in 30 minutes. This aqueous solvent illustrates the useful role of hydrogen bonding and the high dielectric constant of aqueous media under microwave conditions. DMF and DMSO yielded of 80% and 30%, respectively, under identical conditions. At 35 minutes, EtOH produced a 70% yield, in contrast, MeOH provided the highest yield of 95%, highlighting its superior efficiency due to its high polarity, excellent miscibility (with the reactants), and effective absorption of energy under microwave-assisted conditions. These findings further support the influence of solvent polarity and dielectric properties in optimizing microwave-assisted heterocyclic synthesis to reduce the time.

To achieve both higher yield and reduced reaction time, a combined strategy, microwave irradiation (180 W) and GO catalysis, was employing and explored. Reactions were performed in various solvents with GO, resulting in significantly enhanced efficiency compared to individual methods. Under these conditions, DMF yielded 66% in 12 minutes, and DCM gave 41% in 9 minutes. EtOH and DMSO both afforded 54% yields, though with different reaction times. MeOH delivered 56% yield in just 3 minutes using 5 wt% GO. Most notably, H_2_O proved to be the most effective solvent, yielding a 95% conversion in just 4 minutes using lower catalyst concentrations (1% and 0.05%). Interestingly, the lowest GO loading (0.05 wt%) resulted in the highest yield, indicating enhanced catalytic efficiency at minimal concentrations. This suggests that using excessive catalyst may lead to aggregation or reduced accessibility of active sites. While minimal loading allows for optimal dispersion and surface activity. The optimized condition (0.05 wt% GO, H_2_O as solvent, microwave irradiation (180 W)) and results confirm the synergistic advantage of combining GO with microwave methodology for rapid and sustainable synthesis.

### 2.2. Catalytic cycle and structural characterization of graphene oxide (GO)

**Fig. 2:**
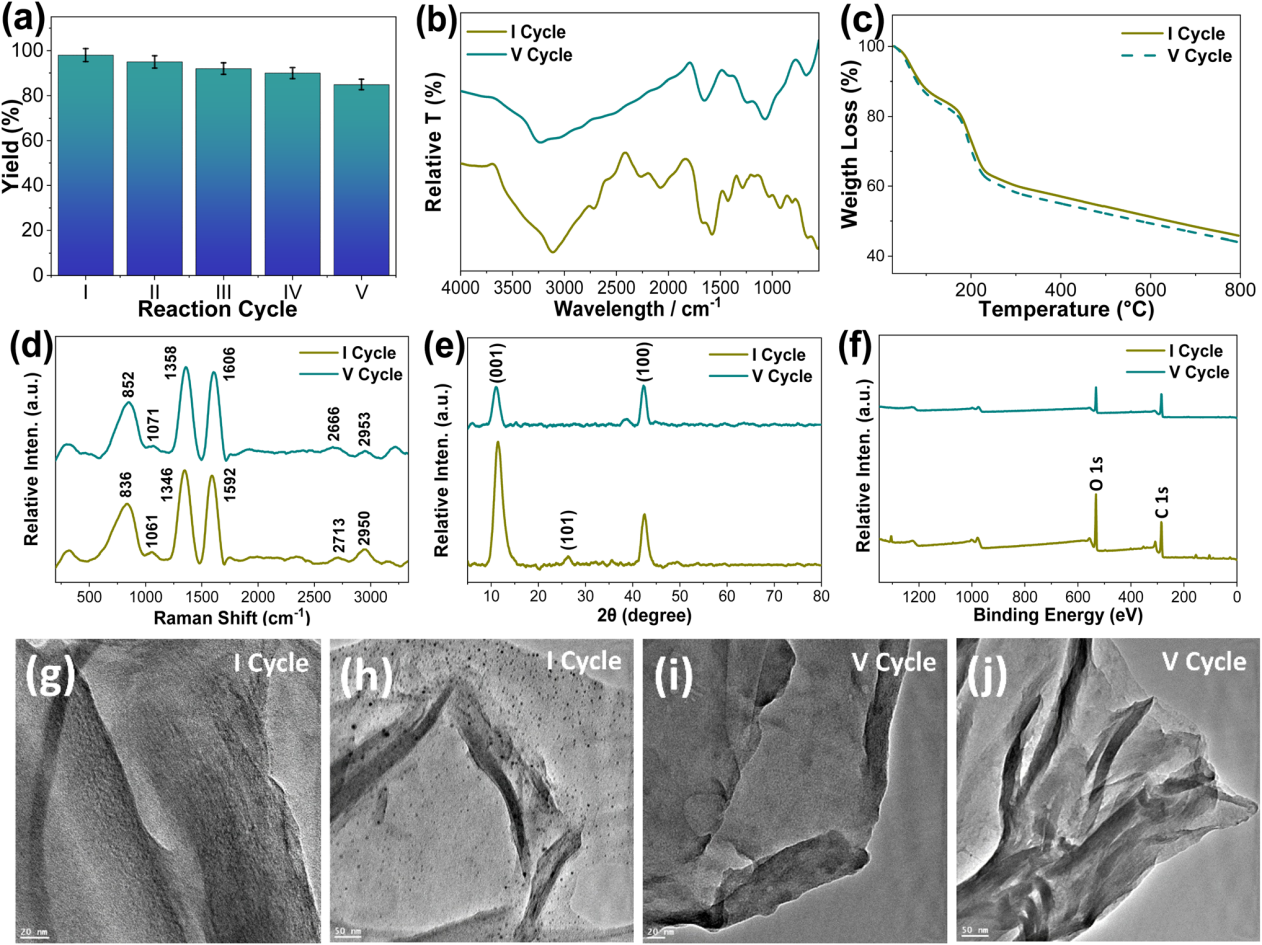
(a) Percentage (%) yield *vs.* reaction cycle, of pure and recycled Graphene Oxide (GO, after each cycle) at optimized reaction conditions. Catalytic characterization (before first (I) and after fifth (V) reaction cycles) by using FT-IR (b); TGA (c); Raman (d); XRD (e); XPS (f); and HR-TEM ((g–j), scale bar represents 20 (g and i) and 50 (h & j) nm), respectively.

The synthesized GO, *via* a modified Hummers' method, was applied as a heterogeneous catalyst for the synthesis of pyrazol-5-ol under optimized microwave conditions (180 W, 4 min) using 0.05 wt% GO in water (Scheme 1), and its reusability was evaluated over five consecutive reaction cycles (I–V) (Fig. 2). Catalytic efficiency was well-retained across cycles, with yields decreasing marginally from 98% (Cycle I) to 85% (Cycle V) (Fig. 2a), confirming GO's robustness and reusability.

The structural and surface characteristics of GO was analysed before (Cycle I) and after use (Cycle V) through XRD, XPS, Raman spectroscopy, FT-IR, TGA, and TEM analyses. FT-IR spectra (Fig. 2b) revealed consistent signals for key functional groups such as O–H (3240 cm^−1^), C=O (1573 cm^−1^), and C–O (1641, 1490, 1067 cm^−1^), indicating the preservation of chemical integrity of GO. Minor suppression of specific peaks in Cycle V suggested a minimal loss of free edge-functional groups.

TGA profiles (Fig. 2c) exhibited multi-step thermal degradation (at 150 °C, 150–300 °C and, >300 °C) with up to 50% weight loss observed at 800 °C. Before Cycle I, a weight loss of GO was reduced and recorded, particularly in the 200–800 °C range. After Cycle V, insignificant weight loss was retained in the same range, indicating consistent thermal stability, which is align with FT-IR results.

The Raman spectrum (Fig. 2d) of the GO sample shows characteristic peaks corresponding to the oxygenated carbon structures. The prominent band transitions (from Cycle I (before) to Cycle V (after)) at 1346 to 1358 cm^−1^ (D-band) and from 1592 to 1606 cm^−1^ (G-band) correspond to disorder carbon (sp^3^ hybridized) and graphitic (GO, sp^2^ hybridized) carbon domains, respectively.^71^ The *I*_D_/*I*_G_ intensity ratio (from 1.32 to 1.24) reflects the partial degree of disorder due to oxidation and structural evolution upon cycling. Additional peaks at 852 and 1071 cm^−1^ correspond to C–O and C–O–C stretching vibrations, confirming the presence of oxygen-containing functional groups in GO even after Cycle V. The broad features around 2713 and 2950 cm^−1^ are attributed to the 2D and C–H stretching modes, respectively. The observed spectral variations between the I and V cycles also suggest structural rearrangement and partial reduction of GO during cycling.

Fig. 2e presents the X-ray diffraction (XRD) patterns of GO recorded for before Cycle I and after Cycle V. The diffraction peaks observed at approximately 2*θ* = 11°, 26° and 42° correspond to the (001), (101) and (100) crystal planes, respectively. These reflections confirm the presence of oxygen-containing functional groups and the formation of a well-defined, partially ordered crystalline phase. The parameters derived from the XRD patterns are summarized in Table S1 (see SI). The change in crystallite size (Table S1), calculated using Scherrer equation, indicates that both samples exhibit nanocrystalline characteristics, as evidenced by broad diffraction peaks. This broadening reflects the presence of few-layered graphene oxide sheets. Morphologically, both GO samples display typical flake-like structures. However, GO after Cycle V shows thinner and more distinct layers, likely resulting from increased oxidation and expanded interlayer spacing due to microwave irradiation. Such changes suggest the occurrence of structural modification or defect formation during repeated cycling, consistent with previous reports.^72^ The interlayer spacing (*d*-spacing) increases slightly from 0.77 nm (Cycle I) to 0.80 nm (Cycle V), indicating a partial loss of functional groups and an increase in layer separation (Table S1). Moreover, a significant decrease in the peak intensity from Cycle I to Cycle V suggests increased structural disorder and reduced crystallinity upon cycling. This behavior have arisen from lattice rearrangement or stress relaxation processes. Nevertheless, the retention of the main diffraction peaks without significant shifting demonstrates that the GO framework remains structurally stable, maintaining its phase purity and crystallinity even after multiple cycles.

The X-ray photoelectron spectroscopy (XPS) analysis of GO both samples reveals pronounced variations in surface chemical composition and bonding states (Fig. 2f, S1 and Table S2, SI). In Cycle I, the C 1s spectrum exhibits three main components at 284.62 eV (C–C, sp^2^), 286.63 eV (C–O, hydroxyl/epoxy), and 287.98 eV (C=O, carbonyl), confirming a highly oxygenated structure. Correspondingly, the O 1s spectrum shows peaks at 532.53 eV (O–C=O) and 533.97 eV (C=O), indicating the abundance of oxygen-containing functional groups. The C/O atomic ratio of 1.80 reflects a typical oxidized graphene framework. After repeated cycling (Cycle V), distinct spectral modifications observed, signifying partial reduction and structural reorganization of GO. The C 1s spectrum primarily comprises peaks at 284.47 eV (C–C) and 286.42 eV (C–O), with the disappearance of the 287.98 eV (C=O) feature, demonstrating the removal of carbonyl functionalities and the restoration of sp^2^-hybridized domains. In the O 1s spectrum, peaks at 530.68 eV and 532.55 eV are attributed to lattice oxygen (C=C) and C–O species, respectively, while the absence of the characteristic 533 eV peak provides strong evidence of deoxygenation. These changes correspond to an increase in the carbon atomic percentage (75.38%) and a decrease in oxygen content (24.62%), yielding an elevated C/O ratio of 3.06. Collectively, these results confirm that repeated catalytic cycling promotes progressive deoxygenation, partial reduction, and enhanced graphitic ordering of GO. This chemical evolution aligns with the XRD findings, which show an increased in crystallite size and improved graphitic domain growth, substantiating the structural restoration of GO upon cycling.

Finally, the morphological features of GO examined using TEM analysis (Fig. 2g–j). The images revealed the characteristic crumpled sheet-like morphology of fresh GO, which remained preserved mainly even after five cycles, showing only minor wrinkling and slight agglomeration. This structural integrity reflects the durability of GO under optimized reaction condition. Collectively, these findings demonstrate that GO maintains its size, shape, crystal structure, thermal, and chemical stability, with minimal loss of edge functional groups and crystal structure discorded, under microwave-assisted conditions, enabling efficient catalysis and reusability, thereby offering a sustainable strategy for heterocyclic synthesis.

### 2.3. Substrate scope and functional group tolerance

#### 2.3.1. Substrate scope with substituted nitrostyrenes (4 series)

**Scheme 2:**
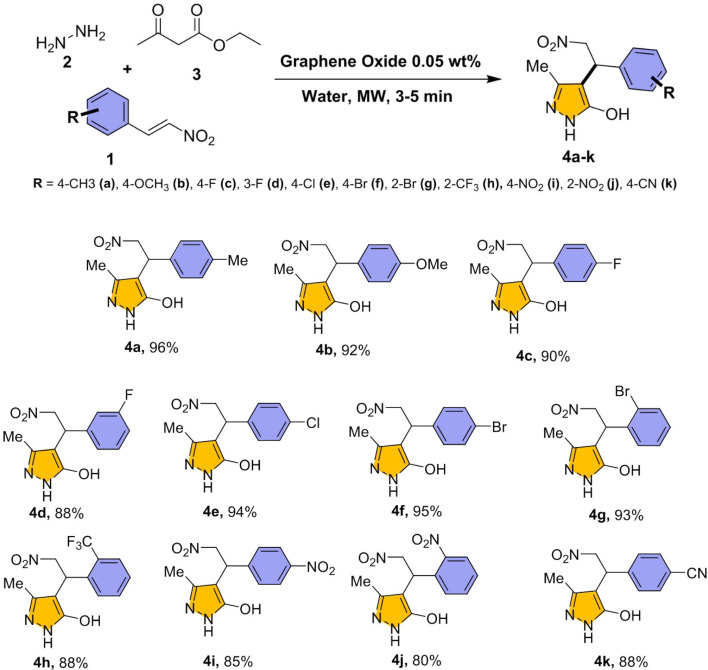
Substrate scope with substituted nitrostyrenes Reaction condition: nitrostyrene 1 (1 eq.), hydrazine hydrate 2 (1 eq.), ethyl acetoacetate 3 (1 eq.), and GO (0.05 wt%) was added in water and exposed under microwave (180 W) for 3–5 min.

Under the optimized reaction conditions (Scheme 1), the scope of the reaction extended to various substituted nitrostyrenes in the presence of hydrazine hydrate, ethyl acetoacetate. As shown in Scheme 2, the protocol demonstrated excellent substrate compatibility, delivering high yields across a broad range of electron-donating and electron-withdrawing groups (compounds 4a–k).

The 4 series compounds were characterized using multiple spectroscopic techniques and the detailed data provided in SI. The results confirmed the presence of key functional groups, including –OH, –OCH_3_ (4b), –CH_3_ (4a), –CF_3_ (4h), –NO_2_ (4i–j), –F (4c–d), –Cl (4e), –Br (4g–f), and –CN (4k). A broad IR absorption band observed at 3390–3410 cm^−1^ corresponds to the stretching vibrations of –OH groups, while strong bands at 1550–1520 cm^−1^ are characteristics of –NO_2_ functionality. In the ^1^H NMR spectra, *para*-substituted derivatives exhibited two aromatic doublets in the range of *δ* 6.8–7.4 ppm, consistent with symmetrical substitution patterns. In contrast, *ortho*-substituted derivatives displayed four distinct aromatic signals between *δ* 7.1 and 8.1 ppm, reflecting increased chemical non-equivalence arising from steric and electronic effects. The signals observed at *δ* 2.05–2.17 ppm attributed to the pyrazole –CH_3_ group, *δ* 4.5–4.7 ppm corresponds to the benzylic –CH proton, and *δ* 4.9–5.3 ppm assigned to the –CH_2_–NO_2_ group. The ^13^C NMR spectra further support the proposed structures, showing aliphatic carbons resonating at *δ* 9–54 ppm, benzylic carbons at *δ* 38–78 ppm, and aromatic/heterocyclic carbons within the *δ* 97–163 ppm. The observed shifts clearly demonstrated the influence of substituents on the electronic environment of the core structure., particularly depending on their orientation on the aromatic ring.

Electron-donating substituents, particularly *para*-methyl groups (4a), afforded superior yields up to 96%, with *para*-methoxy derivatives (4b) yielding 92%. Electron-withdrawing groups such as fluoro (4c, 4d), trifluoromethyl (4h), and nitrile (4k) also showed good reactivity, with yields reaching 88–90%. Additionally, halogenated derivatives—including chloro (4e, 94%), bromo (4f, 95% and 4g, 93%), performed effectively. Substituents in *ortho* (4j) and *para* (4i) positions of nitro derivatives yielded slightly lower but still respectable outcomes (80% and 85%), likely due to steric influence and electron-withdrawing nature.^70^

#### 2.3.2. Substrate scope with substituted nitrostyrenes and phenylhydrazine (6 series)

**Scheme 3:**
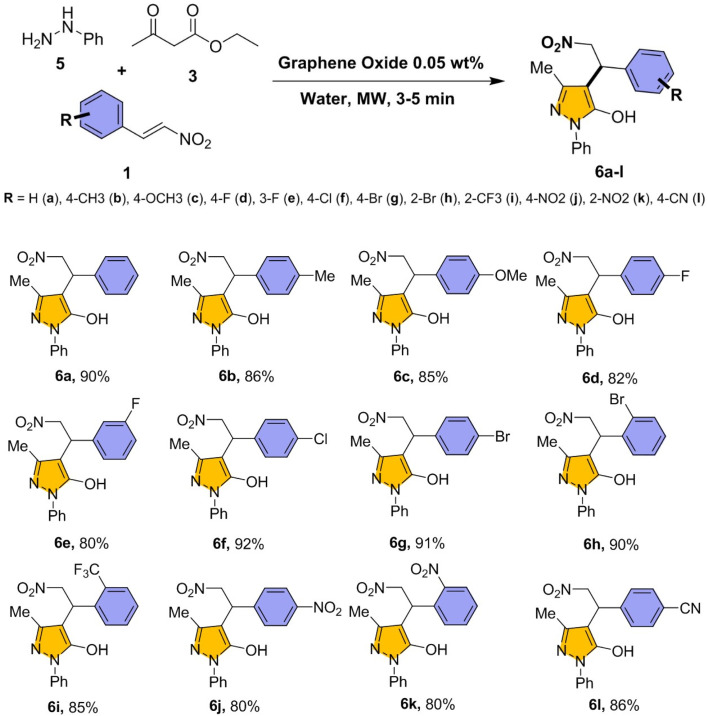
Substrate scope with phenylhydrazine and substituted nitrostyrenes. Reaction condition: nitrostyrene 1 (1 eq.), phenyl hydrazine 5 (1 eq.), ethyl acetoacetate 3 (1 eq.), and GO (0.05 wt%) was added in water and exposed under microwave (180 W) for 3–5 min.

The catalytic efficiency and versatility of GO further evaluated using substituted nitrostyrenes, phenylhydrazine, and ethyl acetoacetate under optimized reaction conditions (Scheme 3). The reaction proceeded rapidly and delivered high yields across a broad range of substituents (products 6a–l), confirming the effectiveness of the protocol.

The 6 series compounds were structurally confirmed by the same spectroscopic techniques (please see SI) and spectral features closely parallel those previously discussed. Key functional groups including –OH and –NO_2_, exhibit similar IR signatures, with broad –OH stretching bands at 3400–3700 cm^−1^ and strong –NO_2_ absorptions at 1550–1520 cm^−1^, consistent with 4-series pattern. In the ^1^H NMR spectra, *para*-substituted derivatives show two aromatic doublets at *δ* 6.8–7.5 ppm, *ortho*-substituted derivatives displayed four resonances at *δ* 7.1–8.3 ppm, and the meta compound (3-F) exhibited a more complex splitting pattern between *δ* 6.9–7.4 ppm. An observed the 4-series, pyrazole methyl protons resonate at *δ* 2.0–2.3 ppm, the CH_2_–NO_2_ protons at *δ* 4.8–5.5 ppm, and the benzylic ArCH signals at *δ* 4.3–4.9 ppm. The ^13^C NMR spectra further corroborate the structural framework, showing an aliphatic signal at *δ* 9–55 ppm, benzylic carbons at *δ* 38–78 ppm, and aromatic/heterocyclic carbons in the *δ* 97–163 ppm region. The overall spectral profile reaffirms the substituent-dependant electronic effects on aromatic core, consistent with trends observed in 4-series.

Nitrostyrenes (6a) and halogenated substituted nitrostyrenes with chloro (6f) and bromo (6g, 6h) groups in *para* and *ortho* positions yielded 90–92%, while methyl (6b) and *para*-methoxy (6c) derivatives gave 85–86%. Fluoro-substituted nitrostyrenes (6d, 6e) showed slightly lower but consistent yields (80–82%). The *para*-trifluoromethyl derivative (6i) afforded 85%, and *ortho*- and *para*-nitro groups (6j, 6k) produced yields of 80%. Notably, *para*-benzonitrile (6l) yielded 86%. Phenylhydrazine (6 series) was effective across the substrate set, though hydrazine hydrate (4 series) consistently produced superior yields.

#### 2.3.3. Substrate scope with heteroaryl hydrazine compounds (8 series)

**Scheme 4:**
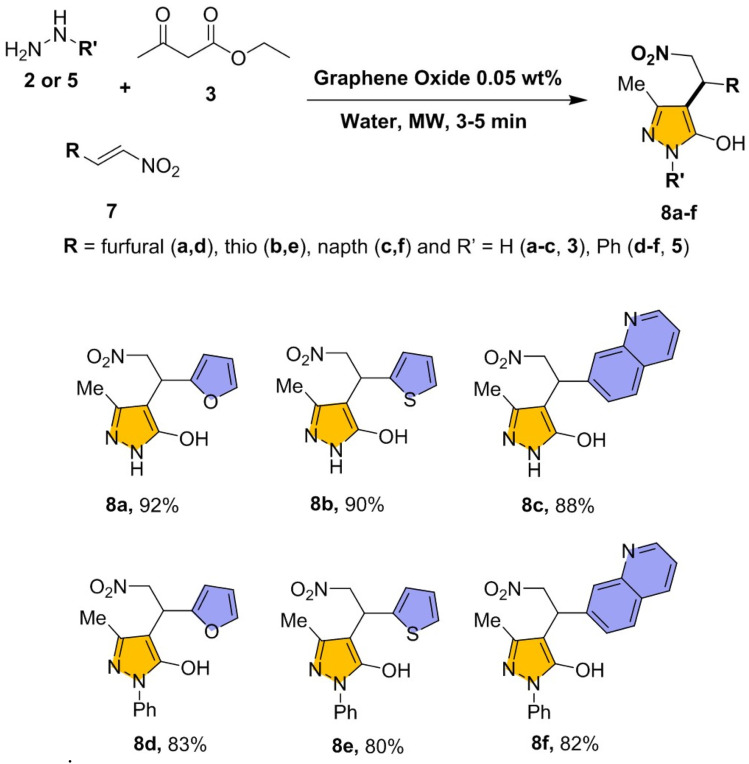
Substrate scope with substituted hydrazine and heteroaryl compounds. Reaction condition: nitro-ethylene based heteroaryl compounds 7 (1 eq.), hydrazine hydrate 2 or phenyl hydrazine 5 (1 eq.), ethyl acetoacetate 3 (1 eq.) and GO (0.05 wt%) was added in water and exposed under microwave (180 W) for 3–5 min.

Building upon above substrate scope, a series of pyrazol-5-ol derivatives (8a–f) was synthesized using heteroaryl hydrazine compounds under optimized conditions (Scheme 4). The reaction exhibited high compatibility with heteroaryl hydrazines such as furan (8a), thiophene (8b), and quinoline (8c), affording excellent yields of 92%, 90%, and 88%, respectively. When phenyl hydrazine used in place of hydrazine hydrate, yields slightly declined (83–82%), due to steric factors.^73^

The 8 series compounds characterized (please see SI) and their core spectral features including –OH and –NO_2_ functional groups, closely align with the previously discussed patterns. Broad IR absorptions at 3230–3700 cm^−1^ (–OH stretch) and strong bands near 1550 cm^−1^ (–NO_2_) were found consistent with these earlier series, along with aromatic C=C stretching in the 1600–1500 cm^−1^ region. Distinct heteroaromatic signatures differentiate the current series from the earlier one. In the ^1^H NMR spectra, furan ring protons resonate at *δ* 6.1–7.5 ppm, thiophene protons at *δ* 6.8–7.2 ppm, and quinoline protons at *δ* 7.4–8.3 ppm, confirmed the incorporation of heteroaryl substituents. Additionally, phenyl-substituted analogues exhibited extra aromatic signals between *δ* 7.0–7.7 ppm, corresponding to the 1-phenylpyrazole moiety. As observed for the 4- and 6-series, the pyrazole methyl group appears as a singlet at *δ* 2.0–2.2 ppm, CH_2_–NO_2_ protons resonate at *δ* 4.8–5.3 ppm, and the benzylic ArCH signal at *δ* 4.6–4.9 ppm. The ^13^C NMR spectra displayed chemical shift distributions analogous to the earlier series (aliphatic *δ* 9–55 ppm, benzylic *δ* 38–78 ppm, and aromatic/heterocyclic *δ* 97–163 ppm), with additional heteroaryl carbon resonances indicative of furan, thiophene, and quinoline rings. Collectively, these data confirmed the successful incorporation of heteroaryl substituents into the pyrazol-5-ol framework, while maintaining the core structural features observed in the 4- and 6-series.

Nevertheless, the method demonstrated broad substrate scope and high efficiency, further confirming its value for the synthesis of structurally diverse pyrazole derivatives featuring heteroaryl functionalities.

### 2.4. Plausible reaction mechanism

**Fig. 3:**
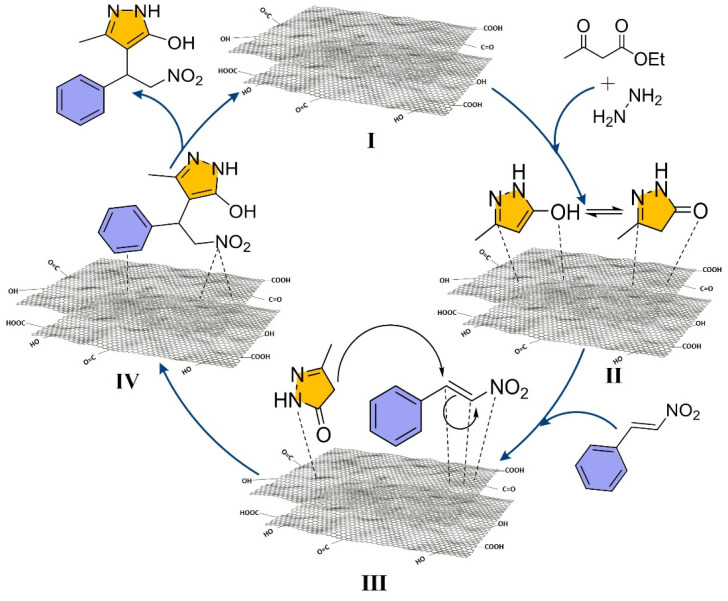
Plausible mechanism pyrazol-5-ol on graphene oxide (GO) under microwave irradiation.

The plausible mechanism describes a multi-component (MCR) synthesis of pyrazol-5-ol in presence of GO and H_2_O under microwave irradiation (180 W) as illustrated in Fig. 3. The catalytic cycle initiated by the adsorption of ethyl acetoacetate and hydrazine hydrate on the GO surface (Intermediate I), The abundant oxygenated functional groups (–OH, –COOH, C–O, and epoxy groups) on GO facilitate strong hydrogen bonding (H-bonding) and π–π stacking interactions, effectively activating the carbonyl moiety of the ethyl acetoacetate. This dual activation polarizes the carbonyl bond and enhancing the electrophilicity of the carbonyl carbon, making it more susceptible to nucleophilic attack. The nucleophilic nitrogen of hydrazine hydrate then attacks the carbonyl carbon, resulting in the formation of tetrahedral intermediate. Proton transfers facilitated by the acidic functionalities on the GO promote dehydration, yielding a stable hydrazone intermediate. This intermediate remains adsorbed on the GO surface, preventing premature desorption and allowing for further intramolecular transformations.

Upon microwave irradiation, the nucleophilic nitrogen of hydrazine hydrate attacks the activated β-ketoester, leading to the rapid formation of a five-membered hydrazone intermediate (Intermediate II). This step again assisted by the acidic sites on GO, which promote proton transfer and stabilize the transition state. The hydrazone then undergoes intramolecular cyclization, forming a pyrazolone-type intermediate.

In the next step (Intermediate III), the activated nitrostyrene undergoes a Knoevenagel-type condensation with the pyrazolone intermediate on GO surface. The GO sheet provides an excellent platform through π–π stacking interactions and H-bonding, aligning the reactants in proximity and thereby facilitating the formation of the C–C bond under mild conditions. The nitrostyrene substrate activated on the GO surface *via* H-bonding interactions between nitro group and –OH group of the GO. In addition, π–π stacking between the aromatic ring of nitrostyrene and the extended π-conjugated GO surface enhances substrate orientation. This combined effect increases the electrophilic character of the β-carbon in nitrostyrene, making it more susceptible to nucleophilic attack by the activated hydrazone. This nucleophilic addition, stabilized by the GO surface, results in the formation of a new C–C bond.

Subsequently, tautomerization and proton rearrangement yield the stable pyrazol-5-ol framework (Intermediate IV) accompanied by the elimination of water or ethanol molecules. This elimination serves as the driving force for the aromatization of the pyrazolol system. Once the product formed, it exhibits weaker interactions with the GO surface compared to the activated intermediates, facilitating desorption. This desorption event is crucial, as it releases the desired product and regenerates the catalytic surface of GO. The catalyst remains structurally intact at the end of the reaction, confirming its stability and reusability.

The reaction progress monitored by TLC, which confirmed the initial formation of the pyrazolol ring, followed by its conjugate addition with nitrostyrene to afford the final product. This stepwise transformation is consistent with the high catalytic efficiency, selectivity, and reusability of GO, underscoring its effectiveness as a sustainable heterogeneous catalyst. The combined use of GO and microwave irradiation creates a synergistic effect, enabling rapid conversion under mild conditions while minimizing energy consumption and waste generation.

### 2.5. Anti-cancer activity, *in vitro*

In the post COVID-19 era, cardiovascular disease, along with cancer, have emerged as the leading causes of morbidity and mortality worldwide.^74^ Among these, cancer remains most lethal, contributing to a significant number of deaths annually across the globe.^75^ Although, new and often more expensive treatments are continuously adopted as standards of care to mitigate cancer-related mortality, the associated costs continue to rise. Given these trends, it's evident that while progress is being made, further research is required to develop more affordable and accessible cancer treatments.

**Fig. 4:**
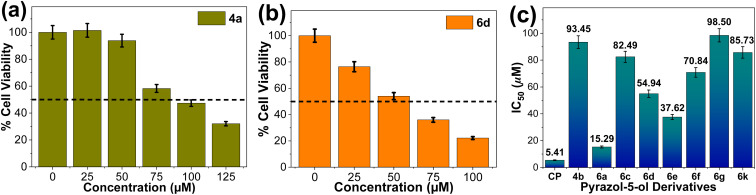
(a and b) Representative percentage (%) cell viability *vs.* concentration graph of pyrazol-5-ol derivatives (4a and 6d). (c) Histogram of IC_50_ (μM) *vs.* individual pyrazol-5-ol derivatives and positive control (Cisplatin, CP) against human lung cancer (A549) cells, *in vitro*. IC_50_ values are indicated on the bars.

To address this need, the present study investigates the cytotoxicity of pyrazol-5-ol derivatives (4, 6 and 8 series) against human lung cancer (A549) cells using an MTT assay, *in vitro* (Fig. 4 and S2, see SI). The result demonstrates that A549 cell viability decreases in a dose-dependent manner upon exposure to pyrazole-5-ol derivatives (0–125 μM) over a 48 h period. Among the tested compounds, 4a and selected derivatives from the 6 series exhibited notable cytotoxic activity, at concentration below 100 μM. In contrast, most derivatives from the 4 and 8 series exhibited no significant cytotoxicity against A549 cells at concentrations up to 100 μM (Fig. S2). The IC_50_ values (concentration required to inhibit 50% of the cell viability), summarized in Fig. 4, ranged from 15 to 98 μM, indicating varying degree of cytotoxicity potency among the tested compounds. For comparison, Cisplatin was employed as a standard (positive control) anticancer drug^76^ and depicted in Fig. 4.

The structure-cytotoxicity relationship revealed several key insights. Among the synthesized all compounds, 6a exhibited the highest anticancer activity, with an IC_50_ value of 15.29 μM, making it the most potent pyrazol-5-ol derivatives in the series. In contrast, 4a and 6g demonstrated the lowest cytotoxicity activity. The enhanced cytotoxicity activity observed in the 6 series derivatives relative to the 4 series suggests that the introduction of the phenyl ring at the mono position of pyrazol-5-ol core improves drug-cell receptor interactions, thereby increasing cytotoxicity potency. Furthermore, halogen substitution (F, Cl, or Br) within 6 series provided an opportunity for fine-tune the cytotoxicity assay. The electronic effects of fluorine substituents on cytotoxicity have been well-documented,^66^ and a similar trend was observed in the present study, supporting our findings. Specifically, cytotoxicity activity decreased in the order of F (6d, 6e) > Cl > Br, underscoring the influence of halogen electronegativity and steric effects on biological activity. Additionally, compound 6k exhibited moderate cytotoxicity against A549 cells. Notably, heterocyclic substitution in pyrazol-5-ol scaffolds completely suppressed the cytotoxic activity, suggesting an unfavourable impact on receptor binding affinity. When compared to the positive control, Cisplatin (5.41 μM), compound 6a demonstrated cytotoxicity potency near to that reference drug, highlighting its potential as a promising lead for further anticancer drug development.

The introduction of Me/OMe and halogen (F, Cl, or Br) functionalities particularly at the *para* and *meta* positions, with phenyl ring substitution, significantly enhanced the cytotoxic activity against the A549 cells. These findings underscore the potential of pyrazol-5-ol derivatives for further development in cancer therapeutics.

### 2.6. Molecular docking against EGFR tyrosine kinase

To rationalize the anticancer potential of pyrazol-5-ol derivatives (4a–k, 6a–l, and 8a–f series) against the A549 cells and guide future structure–activity relationship (SAR) studies, comprehensive molecular docking study was conducted to evaluate the binding affinities and interaction profiles using the Epidermal Growth Factor Receptor Tyrosine Kinase (EGFR-TK) as a target. Docking was performed using the Glide XP module of the Schrödinger Suite,^77^ with the crystal structure of EGFR in complex with erlotinib (PDB ID: 1M17).

The protein structure pre-processed using the protein preparation wizard, including water removal, hydrogen addition, assignment of bond orders, and energy minimization (OPLS-2005 force field). A receptor grid generated around the co-crystallized ligand with a 12.0 Å radius. Ligands prepared using LigPrep, ensuring correct protonation states and geometries.

**Fig. 5:**
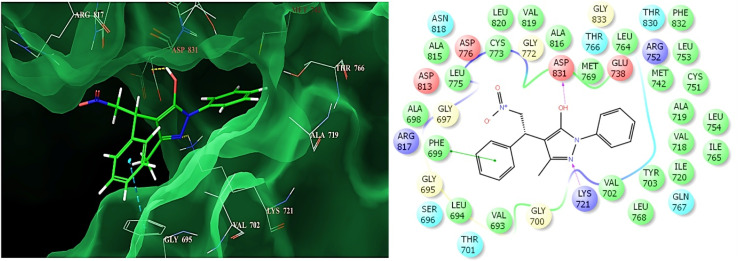
Representative molecular docking of efficient phenyl-based pyrazol-5-ol derivative (6a) with their binding mode into the active site of Epidermal Growth Factor Receptor (EPGR) tyrosine kinase (on right side, docking image binding with pink and green lines of compound with receptor signifies the H-bonding and π–π interactions, respectively).

Flexible docking revealed that all pyrazol-5-ol-based scaffolds fit well within the EGFR active site, with docking scores correlating well with observed cytotoxic activity (Fig. 5 and S3, see SI). The quantitative data are summarized in Table 1. Glide scores (GS) ranged from −6.55 to −8.98 kcal mol^−1^, while Glide interaction energies (GIE) spanned −30.77 to −48.91 kcal mol^−1^, indicating favourable binding across the library for all pyrazol-5-ol derivatives.

**Table 1:** Molecular docking quantitative per-residue interaction analysis (Glide Score, GS; Glide Interaction Energy, GIE; Hydrogen Bonding, H-Bonding; π–π stacking) for the pyrazol-5-ol derivatives (4a–k, 6a–l and 8a–f) with Epidermal Growth Factor Receptor (EGFR) tyrosine kinase

Pyrazole derivatives	GS	GIE kcal mol^−1^	H-bonding Å	π–π Å
4a	−8.407	−48.909	Gln767: 1.85, Met769: 2.26	—
4c	−7.964	−40.278	Gln767: 2.03, Met769: 2.47	—
4e	−7.106	−38.005	Gln767: 2.09, Met769: 2.29	—
4g	−6.562	−34.335	Gln767: 2.06, Thr766: 2.06	—
4i	−8.195	−45.204	Gln767: 2.03, Thr766: 2.05	—
4j	−8.067	−43.182	Met769: 1.91, Met769: 2.32	—
4k	−7.129	−38.924	Gln767: 2.05, Thr766: 2.06, Thr766: 1.90	
6a	−8.980	−47.522	Lys721(2.29), Asp831(2.48)	Phe699(2.567)
6b	−7.496	−39.356	Asp831(2.19)	—
6c	−8.079	−43.932	Asp831(2.20)	—
6d	−8.325	−45.076	Asp831(2.23)	—
6e	−8.707	−46.485	Asp831(2.18)	—
6f	−7.549	−40.047	Asp831(2.20)	—
6g	−7.992	−41.613	Asp831(2.20)	—
6h	−6.962	−31.434	Lys721(2.47)	—
6i	−6.994	−32.034	Asp831(2.09)	—
6j	−7.055	−34.725	Asp831(1.85)	—
6k	−7.349	−37.432	Asp831(1.77)	—
6l	−7.279	−35.885	Asp831(2.19)	—
8d	−6.654	−30.776	Asp831(2.63)	—
8e	−7.171	−35.530	Asp831(2.74)	—
8f	−7.839	−41.010	Asp831(2.02)	—

Compounds in the 4-series exhibited average docking scores of −7.632 kcal mol^−1^ and Glide binding energies around −41.262 kcal mol^−1^. Notably, compound 4a displayed the best performance (Glide score: −8.407 kcal mol^−1^, binding energy: −48.909 kcal mol^−1^), stabilized *via* hydrogen bonding with Gln767 (1.85 Å) and Met769 (2.26 Å), which is well align with cytotoxicity data (Fig. 4). Similarly, 4e and 4f also showed strong binding *via* interactions with Thr766, Gln767, and Met769.

Among the 6-series, compound 6a demonstrated the highest affinity (Glide score: −8.980 kcal mol^−1^, binding energy: −47.522 kcal mol^−1^), forming dual hydrogen bonds with Lys721 (2.29 Å) and Asp831 (2.48 Å), along with a π–π stacking interaction with Phe699 (2.567 Å). These strong affinities and interactions were also demonstrated through improved toxicity against A549 (Fig. 4). Compounds 6e (GS: −8.707, GIE: −46.485 kcal mol^−1^) and 6d also showed firm binding profiles, driven by hydrogen bonding interactions, primarily involving Asp831.

In the 8-series, only phenylhydrazine-based derivatives (8d–f) showing capable docking results compared to 8a–c compounds, indicating consistent interactions. However, compound 8f emerged as the most promising (Glide score: −7.839 kcal mol^−1^, binding energy: −41.010 kcal mol^−1^), forming a key hydrogen bond with Asp831 (2.02 Å). Other derivatives such as 8d and 8e showed comparatively weaker binding. Per-residue interaction analysis highlighted Asp831, Lys721, Gln767, and Met769 as critical residues in ligand recognition. The consistent engagement of these amino acids across potent compounds underscores their importance in EGFR inhibition.

Overall, compound 6a demonstrated the most favourable binding profile, with strong hydrogen bonding and π–π stacking interactions. The nature and positioning of substituents appeared to influence interaction strength and type significantly. These findings suggest that electron-rich or hydrogen-bond-accepting groups near pharmacophoric regions enhance binding efficiency, with series 6 compounds emerging as promising EGFR inhibitors warranting further biological evaluation.

## 3. Conclusion

In conclusion, this study presents a sustainable and highly efficient approach for the aqueous synthesis of pyrazol-5-ol derivatives (4, 6 and 8 series) using graphene oxide (GO) as a green and, reusable catalyst under microwave (180 W) irradiation. The methodology offers remarkable benefits, including short reaction times, excellent yields (80–95%), mild conditions, and broad substrate compatibility. GO exhibited excellent catalytic stability and reusability, while maintaining its nanoscale flake-like morphology, uniform sheet-like structure, and crystalline integrity, as confirmed by various spectroscopic and microscopic analyses, highlighting its potential for eco-friendly heterocyclic synthesis. The synthesized compounds, particularly 6-series, showed strong binding affinities to the EGFR active site, as revealed by molecular docking studies and key interactions with residues critical to kinase inhibition. The anticancer activities, *in vitro*, further supported these findings, displaying significant cytotoxicity against human lung cancer A549 cells, with compound 6a emerging as the most potent (IC_50_ = 15.29 μM) one. The synergistic integration of green chemistry principles, catalysis, and bio-activity-guided synthesis underscores the therapeutic potential of these compounds. Overall, this work not only highlights the effectiveness of GO-catalyzed synthesis in microwave conditions in drug discovery but also provides a valuable platform for the development of next-generation EGFR-targeted anticancer agents.

## 4. Experimental section

### 4.1. General

All reactions examined by thin-layer chromatography (TLC) on Merck TLC Silica gel 60 F_254_ silica gel plates. The spots were visualized under ultraviolet light and/or by staining with *p*-anisaldehyde, followed by heating as revealing agent. Melting point obtained on an open-capillary electrothermal apparatus and reported as observed. Nuclear Magnetic Resonance (NMR) were recorded on a Bruker 500 MHz FT-NMR spectrometer, with ^1^H NMR and ^13^C NMR (at 126 MHz) measurements chemical shifts are reported relative to the tetramethylsilane (TMS) as an internal standard, and compounds were dissolved in CDCl_3_ and DMSO-d_6_. Multiplicity is indicated as follows: singlet (s), broad singlet (bs), doublet (d), triplet (t), quartet (q), multiplet (m), doublet of doublets (dd) with coupling constants *J* given in Hz. IR spectra recorded using an FT-IR-5700 spectrometer, while high-resolution mass spectrometry (HR-MS) performed to determine the molecular weight of synthesized compounds. Human lung adenocarcinoma cell line (A549) was purchased from the National Centre for Cell Science, Pune, India. All chemicals and solvents, sourced from Merck, Sigma Aldrich, Hi-media and Spectrochem, were used as received without further purification. Distilled water (2–5 μS cm^−1^, hereafter referred as water) used throughout the research work.

### 4.2. Preparation of graphene oxide (GO)

In the present study, graphene oxide (GO) prepared by modified Hummer's method.^72,78,79^ Flake graphite (10 g) along with oxidants (KMnO_4_, 6 g) and stabilizer (boric acid, 0.01 g) were first slowly dispersed in 100 mL of conc. H_2_SO_4_ in a vessel with continuous stirring for 1.5 h at a temperature of less than 5 °C. After that, an additional KMnO_4_ (5 g) was added, and the vessel transferred to a water bath at approximately 35 °C, where it was stirred for another 3 h to complete the deep oxidation process. Next, 250 mL of deionized water slowly added, and the temperature raised to 95 °C, where it maintained for 15 minutes. The diluted suspension turned brown, indicating the hydrolysis and complete exfoliation of the intercalated graphite oxide. Finally, this brown suspension was treated with 12 mL of 30% H_2_O_2_ to reduce the remaining oxidants and intermediates to soluble sulphate. The mixture was centrifuged at 10 000 rpm for 20 minutes to remove residual graphite. It was washed repeatedly with 1 mol per L HCl and deionized water, yielding the final product of GO. The synthesized GO was characterized using FT-IR (Bruker, Alpha 2 Platinum), TEM (JEOL, JEM F20 electron microscope, CSMCRI Bhavnagar), TGA (SDT Q600 V20.9 Build 20, N2 atm, 200.14 to 989.72 °C), XRD (EMPYREAN X-ray diffractometer (Analytical, Almelo, Netherland) with Cu-Kα radiation (*λ* = 1.5406 Å), operating at 45 kV and 30 mA. The *d*-spacing values obtained using Bragg's equation), XPS (Thermo Scientific ESCALAB 250Xi, Al Kα (1486.6 eV)), and Raman Spectroscopy (DXR3xi, Thermo Fisher Scientific) to confirm its size, shape, crystal structural, morphological, thermal, and chemical characteristics.

### 4.3. Multi-component synthesis of 3-methyl-4-(2-nitro-1-phenylethyl)-1*H*-pyrazol-5-ol (4)

Multi-component, nitrostyrene (1, 1 mmol, 0.15 g) and hydrazine hydrate (2, 1 mmol, 0.32 g), with/without graphene oxide (GO, at varying weight percentage (wt%) of 0–5%) were dissolved in a polar solvent (MeOH, EtOH, DMSO, DCM, DMF, or H_2_O, 5 mL) and transferred into round-bottom flask (RBF, 50 mL) with continuous stirring. Ethyl acetoacetate (3, 1 mmol, 0.13 g) was then added drop-wise over 2–4 min into the same RBF. The reaction mixture was either refluxed or subjected to microwave irradiation (180 W) until a light brown to whitish-yellow precipitate (ppt) formed, as indicated by TLC. The ppt was filtered, washed with cold water (10 mL) and brine (10 mL), passed through a celite pad to remove GO, and dried over sodium sulfate. The solvent evaporated under vacuum, and the crude product was recrystallize using a suitable solvent to obtain a desired white solid product (4) with an appropriate yield (20–90%). *R*_f_: 0.40 (70% EtOAc/hexane); M.P.: 166–168 °C; IR (KBr, cm^−1^): 3433, 2923, 2852, 1558, 1430, 1382, 1198, 1050, 817, 767, 743; ^1^H NMR (500 MHz, CDCl_3_): *δ* 2.15 (s, 3H, C3–CH_3_) 4.62 (t, *J* = 8.0 Hz, 1H, Ar*C*H), 4.97 (dd, *J* = 12.9, 7.3 Hz, 1H, C*H*_*2*_NO_2_), 5.22 (dd, *J* = 12.8, 9.0 Hz, 1H, C*H*_*2*_NO_2_), 7.23 (d, *J* = 7.3 Hz, 1H, ArH), 7.30 (dd, *J* = 14.4, 7.2 Hz, 2H, ArH), 7.40 (d, *J* = 7.6 Hz, 2H, ArH); ^13^C NMR (126 MHz, CDCl_3_): *δ* 10.4 (C6) 39.6 (C7), 78.0 (C8), 100.6 (C4), 127.6 (C15), 127.8 (C17,13), 129.0 (C16, 14), 139.6 (C3), 139.7 (C12), 161.3 (C5); HR-MS (+SI): *m*/*z* calcd for C_12_H_13_N_3_O_3_ [M + H]^+^: 248.10351, found 248.09420.

### 4.4. Synthesis of pyrazol-5-ol derivatives (4a–k)

Substituted nitrostyrene (1, 1 mmol) and hydrazine hydrate (2, 1 mmol), were dissolved in water (3–5 mL) along with graphene oxide (GO, fixed at 0.05 wt%), and transferred into round bottom flask (RBF, 50 mL) under continuous stirring. Ethyl acetoacetate (3, 1 mmol) was then added drop-wise over 2–4 min into the same RBF. The reaction mixture subjected to microwave irradiation (180 W) until the light brown to whitish-yellow precipitate (ppt) formed, as confirmed by TLC. The work-up procedure was remained same as described earlier for synthesis of pyrazol-5-ol (4). The desired white to yellowish solid (pyrazole-5-ol derivatives, 4a–k) product obtained in good yields (80–96%).

#### 4.4.1. 3-Methyl-4-(2-nitro-1-(p-tolyl)ethyl)-1*H*-pyrazol-5-ol (4a)

96% (0.24 g) yield as white solid; *R*_f_: 0.41 (70% EtOAc/hexane); M.P.: 171–173 °C; IR (KBr, cm^−1^): 3395, 3025, 2923, 2855, 1906, 1711, 1603, 1551, 1514, 1429, 1376, 1218, 1151, 1115, 1077, 1021, 1037; ^1^H NMR (500 MHz, CDCl_3_): *δ* 2.11 (s, 3H, C3–CH_3_), 2.30 (s, 3H, C15–CH_3_), 4.56 (t, *J* = 8.0 Hz, 1H, Ar*C*H), 4.93 (dd, *J* = 12.8, 7.2 Hz, 1H, C*H*_*2*_NO_2_), 5.20 (dd, *J* = 12.6, 9.0 Hz, 1H, C*H*_*2*_NO_2_), 7.10 (d, *J* = 7.7 Hz, 2H, ArH), 7.27 (d, *J* = 8.0 Hz, 2H, ArH); ^13^C NMR (126 MHz, CDCl_3_): *δ* 8.2 (C6), 19.1 (C15–*C*H_3_), 36.9 (C7), 76.2 (C8), 97.5 (C4), 125.7 (C15), 127.4 (C17, C13), 134.3 (C16, 14), 135.7 (C3), 135.9 (C12), 157.9 (C5); HR-MS (+SI): *m*/*z* calcd for C_13_H_15_N_3_O_3_ [M + H] ^+^: 262.11916, found 262.11208.

#### 4.4.2. 4-(1-(4-Methoxyphenyl)-2-nitroethyl)-3-methyl-1*H*-pyrazol-5-ol (4b)

92% (0.26 g) yield as white solid; *R*_f_: 0.40 (70% EtOAc/hexane); M.P.: 159–162 °C; IR (KBr, cm^−1^): 3414, 2923, 2852, 1613, 1551, 1512, 1462, 1378, 1249, 1179, 1114, 1030; ^1^H NMR (500 MHz, CDCl_3_): *δ* 2.14 (s, 3H, C3–CH_3_), 3.77 (s, 3H, O–CH_3_), 4.56 (dd, *J* = 18.1, 9.9 Hz, 1H, ArC*H*), 4.93 (dd, *J* = 12.8, 7.4 Hz, 1H, C*H*_*2*_NO_2_), 5.18 (dd, *J* = 12.7, 8.9 Hz, 1H, C*H*_*2*_NO_2_), 6.84 (d, *J* = 8.3 Hz, 2H, ArH), 7.31 (d, *J* = 8.3 Hz, 2H, ArH); ^13^C NMR (126 MHz, CDCl_3_): *δ* 9.1 (C6), 37.6 (C7), 54.1 (C15–O*C*H3), 77.2 (C8), 98.3 (C4), 112.9 (C15), 127.7 (C17, C13), 131.3 (C16,14), 137.2 (C3), 157.4 (C12), 159.3 (C5); HR-MS (+SI): *m*/*z* calcd for C_13_H_15_N_3_O_4_ [M + H]^+^: 278.11408, found 278.10445.

#### 4.4.3. 4-(1-(4-Fluorophenyl)-2-nitroethyl)-3-methyl-1*H*-pyrazol-5-ol (4c)

90% (0.23 g) yield as white solid; *R*_f_: 0.42 (70% EtOAc/hexane); M.P.: 163–166 °C; IR (KBr, cm^−1^): 3389, 2923, 2851, 1605, 1557, 1509, 1435, 1378, 1227, 1160, 1103; ^1^H NMR (500 MHz, CDCl_3_): *δ* 2.13 (s, 3H, C3–CH_3_), 4.57 (dd, *J* = 17.9, 9.9 Hz, 1H, ArC*H*), 4.93 (dd, *J* = 12.9, 7.4 Hz, 1H, C*H*_*2*_NO_2_), 5.17 (dd, *J* = 12.8, 8.8 Hz, 1H, C*H*_*2*_NO_2_), 7.05–6.92 (m, 2H, ArH), 7.37 (dd, *J* = 7.5, 5.6 Hz, 2H, ArH); ^13^C NMR (126 MHz, CDCl_3_): *δ* 10.4 (C6), 38.9 (C7), 100.5 (C8), 115.8 (C4), 116.0 (C15), 129.4 (C17,C13), 135.5 (C16,14), 139.8 (C3), 161.2 (C12), 163.1 (C5); HR-MS (+SI): *m*/*z* calcd for C_12_H_12_FN_3_O_3_ [M + H] ^+^: 266.09409, found 266.08781.

#### 4.4.4. 4-(1-(3-Fluorophenyl)-2-nitroethyl)-3-methyl-1*H*-pyrazol-5-ol (4d)

88% (0.23 g) yield as white solid; *R*_f_: 0.42 (70% EtOAc/hexane); M.P.: 156–158 °C; IR (KBr, cm^−1^): 3394, 2852, 2568, 1592, 1552, 1522, 1488, 1450, 1256, 1229, 1190, 1140; ^1^H NMR (500 MHz, CDCl_3_): *δ* 7.35 (d, *J* = 12.1 Hz, 1H, ArH), 7.32–7.12 (m, 3H, ArH), 6.92 (t, *J* = 8.0 Hz, 1H, ArH), 5.12 (dd, *J* = 12.8, 8.3 Hz, 1H, C*H*_*2*_NO_2_), 5.03 (dd, *J* = 12.8, 7.9 Hz, 1H, C*H*_*2*_NO_2_), 4.59 (t, *J* = 7.9 Hz, 1H, ArC*H*), 2.60 (s, 1H), 2.09 (d, *J* = 45.1 Hz, 3H, C3–CH_3_); ^13^C NMR (126 MHz, CDCl_3_): *δ* 9.1 (C6), 28.5 (C7), 38.1 (C8), 97.6 (C4), 112.9 (C13), 113.5 (C17), 122.6 (C14), 129.3 (C16), 137.4 (C3), 142.1 (C12), 159.1 (C15), 162.6 (C5); HR-MS (+SI): *m*/*z* calcd for C_12_H_12_FN_3_O_3_ [M + H]^+^: 266.09409, found 266.08873.

#### 4.4.5. 4-(1-(4-Chlorophenyl)-2-nitroethyl)-3-methyl-1*H*-pyrazol-5-ol (4e)

94% (0.26 g) yield as white solid; *R*_f_: 0.43 (70% EtOAc/hexane); M.P.: 147–149 °C; IR (KBr, cm^−1^): 3394, 2924, 2853, 2573, 1907, 1713, 1606, 1552, 1522, 1492, 1430, 1411, 1378, 1289, 1275, 1201, 1180, 1152, 919, 738, 645, 591, 557; ^1^H NMR (500 MHz, DMSO): *δ* 2.10 (s, 3H, C3–CH_3_), 4.54 (t, *J* = 8.0 Hz, 1H, ArC*H*), 5.03 (dd, *J* = 12.8, 7.9 Hz, 1H, C*H*_*2*_NO_2_), 5.11 (dd, *J* = 12.6, 8.5 Hz, 1H, C*H*_*2*_NO_2_), 7.25 (d, *J* = 8.2 Hz, 2H, ArH), 7.38 (d, *J* = 8.2 Hz, 2H, ArH), 7.77 (s, 1H, ArH); ^13^C NMR (126 MHz, DMSO): *δ* 9.7 (C6), 38.2 (C7), 77.4 (C8), 98.3 (C4), 128.2 (C17, C13), 129.0 (C16, C14), 131.8 (C3), 137.6 (C15), 139.1 (C12), 159.4 (C5); HR-MS (+SI): *m*/*z* calcd for C_12_H_12_ClN_3_O_3_ [M + H]^+^: 282.06454, found 282.05832.

#### 4.4.6. 4-(1-(4-Bromophenyl)-2-nitroethyl)-3-methyl-1*H*-pyrazol-5-ol (4f)

95% (0.30 g) yield as white solid; *R*_f_: 0.43 (70% EtOAc/hexane); M.P.: 172–175 °C; IR (KBr, cm^−1^): 3391, 2924, 1606, 1550, 1520, 1488, 1378, 1073, 858, 850, 756, 734, 650, 591, 557; ^1^H NMR (500 MHz, CDCl_3_): *δ* 2.11 (s, 3H, C3–CH_3_), 4.55 (t, *J* = 8.0 Hz, 1H, ArC*H*), 5.02 (dd, *J* = 12.8, 8.0 Hz, 1H, C*H*_*2*_NO_2_), 5.12 (dd, *J* = 12.6, 8.5 Hz, 1H, C*H*_*2*_NO_2_), 7.31 (d, *J* = 8.1 Hz, 2H, ArH), 7.41 (d, *J* = 7.8 Hz, 2H, ArH), 7.57–7.49 (m, 1H, ArH); ^13^C NMR (126 MHz, CDCl_3_): *δ* 9.5 (C6), 39.0 (C7), 77.2 (C8), 98.1 (C4), 120.3 (C17, 13), 129.0 (C16, C14), 131.1 (C3), 138.0 (C15), 138.6 (C12), 159.9 (C5); HR-MS (+SI): *m*/*z* calcd for C_12_H_12_BrN_3_O_3_ [M + H]^+^: 326.01402, found 326.01456.

#### 4.4.7. 4-(1-(2-Bromophenyl)-2-nitroethyl)-3-methyl-1*H*-pyrazol-5-ol (4g)

93% (0.30 g) yield as white solid; *R*_f_: 0.43 (70% EtOAc/hexane); M.P.: 200–202 °C; IR (KBr, cm^−1^): 3389, 2956, 2923, 2852, 1609, 1547, 1515, 1467, 1434, 1377, 1261, 1222, 1146, 1075, 1023, 857, 803, 709, 663, 639, 595, 537, 522; ^1^H NMR (500 MHz, CDCl_3_): *δ* 2.17 (s, 3H, C3–CH_3_), 4.87–4.77 (m, 1H, ArC*H*), 5.14 (d, *J* = 9.8 Hz, 1H, C*H*_*2*_NO_2_), 5.24 (t, *J* = 11.2 Hz, 1H, C*H*_*2*_NO_2_), 7.12 (d, *J* = 6.3 Hz, 1H, ArH), 7.28 (d, *J* = 7.0 Hz, 1H, ArH), 7.56 (d, *J* = 7.7 Hz, 1H, ArH), 7.68 (d, *J* = 6.4 Hz, 1H, ArH); ^13^C NMR (126 MHz, CDCl_3_): *δ* 10.3 (C6), 38.2 (C7), 76.1 (C8), 98.1 (C4), 127.8 (C17), 128.7 (C14), 130.0 (C16), 132.8 (C3), 138.5 (C12), 139.3 (C13), 160.6 (C5); HR-MS (+SI): *m*/*z* calcd for C_12_H_12_BrN_3_O_3_ [M + H]^+^: 326.01402, found 326.01289.

#### 4.4.8. 3-Methyl-4-(2-nitro-1-(2-(trifluoromethyl)phenyl)ethyl)-1*H*-pyrazol-5-ol (4h)

88% (0.27 g) yield as white solid; *R*_f_: 0.43 (70% EtOAc/hexane); M.P.: 253–257 °C; IR (KBr, cm^−1^): 3389, 2253, 1653, 1552, 1455, 1428, 1378, 1312, 1227, 1161, 1127, 1026; ^1^H NMR (500 MHz, CDCl_3_): *δ* 2.05 (s, 3H, C3–CH_3_), 4.62 (dd, *J* = 12.9, 4.6 Hz, 1H, C*H*_*2*_NO_2_), 4.98 (dd, *J* = 10.6, 4.5 Hz, 1H, C*H*_*2*_NO_2_), 5.42 (dd, *J* = 26.2, 14.3 Hz, 1H, C*H*_*2*_NO_2_), 7.39 (t, *J* = 7.5 Hz, 1H, ArH), 7.56 (t, *J* = 7.6 Hz, 1H, ArH), 7.66 (d, *J* = 7.8 Hz, 1H, ArH), 8.07 (d, *J* = 7.8 Hz, 1H); ^13^C NMR (126 MHz, DMSO): *δ* 9.4 (C6), 34.9 (C7), 76.8 (C8), 97.8 (C4), 123.1 (C18), 125.5 (C13), 125.5 (C16), 127.1 (C17, 15), 130.7 (C13–CF_3_), 132.4 (C14), 138.4 (C12), 138.8 (C3), 159.8 (C5); HR-MS (+SI): *m*/*z* calcd for C_13_H_12_F_3_N_3_O_3_ [M + H]^+^: 316.09090, found 316.08966.

#### 4.4.9. 3-Methyl-4-(2-nitro-1-(4-nitrophenyl)ethyl)-1*H*-pyrazol-5-ol (4i)

85% (0.24 g) yield as white solid; *R*_f_: 0.39 (70% EtOAc/hexane); M.P.: 128–132 °C; IR (KBr, cm^−1^): 3388, 2923, 2852, 1716, 1606, 1557, 1516, 1430, 1378, 1214, 1110, 1014; ^1^H NMR (500 MHz, CDCl_3_): *δ* 2.16 (s, 3H, C3–CH_3_), 2.59 (s, 1H, ArC*H*), 4.70 (t, *J* = 7.9 Hz, 1H, C*H*_*2*_NO_2_), 5.13 (td, *J* = 21.4, 13.1 Hz, 2H, C*H*_*2*_NO_2_), 7.62 (d, *J* = 8.3 Hz, 2H, ArH), 8.15 (d, *J* = 8.3 Hz, 2H, ArH); ^13^C NMR (126 MHz, CDCl_3_): *δ* 9.7 (C6), 38.8 (C7), 97.7 (C4), 123.5 (C4), 128.4 (C17, C13), 138.6 (C16, C14), 146.5 (C3), 147.4 (C15), 160.0 (C12), 173.4 (C5); HR-MS (+SI): *m*/*z* calcd for C_12_H_12_N_4_O_5_ [M + H]^+^: 293.08859, found 293.08622.

#### 4.4.10. 3-Methyl-4-(2-nitro-1-(2-nitrophenyl)ethyl)-1*H*-pyrazol-5-ol (4j)

80% (0.23 g) yield as white solid; *R*_f_: 0.38 (70% EtOAc/hexane); M.P.: 148–152 °C; IR (KBr, cm^−1^): 3406, 2924, 1709, 1550, 1527, 1466, 1383, 1079, 860, 788, 756, 712; ^1^H NMR (500 MHz, CDCl_3_): *δ* 2.13 (s, 3H, C3–CH_3_), 5.00 (dd, *J* = 16.8, 10.0 Hz, 1H, ArC*H*), 5.34–5.16 (m, 2H, C*H*_*2*_NO_2_), 7.40 (t, *J* = 7.6 Hz, 1H, ArH), 7.57 (t, *J* = 7.4 Hz, 1H, ArH), 7.82 (d, *J* = 7.8 Hz, 1H, ArH), 7.96 (d, *J* = 7.7 Hz, 1H, ArH); ^13^C NMR (126 MHz, CDCl_3_): *δ* 9.4 (C6), 28.9 (C7), 76.2 (C8), 97.3 (C4), 123.7 (C16), 127.6 (C15), 130.2 (C13), 132.6 (C14), 133.7 (C17), 138.8 (C3), 148.3 (C12), 159.9 (C5); HR-MS (+SI): *m*/*z* calcd for C_12_H_12_N_4_O_5_ [M + H]^+^: 293.08859, found 293.08705.

#### 4.4.11. 4-(1-(5-Hydroxy-3-methyl-1*H*-pyrazol-4-yl)-2-nitroethyl)benzonitrile (4k)

88% (0.23 g) yield as white solid; *R*_f_: 0.42 (70% EtOAc/hexane); M.P.: 168–172 °C; IR (KBr, cm^−1^): 3394, 2956, 2853, 2924, 2226, 1607, 1554, 1517, 1465, 1379, 1276, 1210, 1177, 1152 cm^−1^; ^1^H NMR (500 MHz, CDCl_3_): *δ* 2.13 (s, 3H, C3–CH_3_), 4.64 (t, *J* = 7.8 Hz, 1H, ArC*H*), 5.10–5.04 (m, 1H, C*H*_*2*_NO_2_), 5.18–5.10 (m, 1H, C*H*_*2*_NO_2_), 7.58 (dd, *J* = 17.6, 7.9 Hz, 4H, ArH); ^13^C NMR (126 MHz, CDCl_3_): *δ* 14.1 (C6), 31.8 (C7), 98.3 (C8), 111.0 (C4–CN), 114.0 (C15), 128.6 (C17, C13), 132.5 (C16, C14), 139.2 (C3), 145.5 (C12), 160.5 (C5); HR-MS (+SI): *m*/*z* calcd for C_13_H_12_N_4_O_3_ [M + H]^+^: 273.09876, found 273.09451.

### 4.5. Synthesis of phenyl pyrazol-5-ol derivatives (6a–l)

Substituted nitrostyrene (1, 1 mmol) and phenylhydrazine (5, 1 mmol), were solubilized in water (3–5 mL) along with graphene oxide (GO, fixed at 0.05 wt%) and transferred into round bottom flask (RBF, 50 mL) under continuous stirring. Ethyl acetoacetate (3, 1 mmol) was then added drop-wise over 2–5 min to the same RBF. The reaction mixture was subjected to microwave irradiation (180 W) until a light brown to whitish-yellow precipitate (ppt) began to form, as confirmed by TLC. The work-up procedure remained the same as described earlier for the synthesis of pyrazol-5-ol (4). The desired phenyl pyrazol-5-ol derivatives (6a–l) obtained as white to yellowish solids with a good yield (80–90%).

#### 4.5.1. 3-Methyl-4-(2-nitro-1-phenylethyl)-1-phenyl-1*H*-pyrazol-5-ol (6a)

90% (0.22 g) yield as yellow solid; *R*_f_: 0.27 (30% EtOAc/hexane); M.P.: 162–165 °C; IR (KBr, cm^−1^): 3257, 2922, 1956, 1671, 1626, 1595, 1551, 1492, 1456, 1411, 1376, 1299, 1138, 1003, 983, 910, 808; ^1^H NMR (500 MHz, CDCl_3_): *δ* 7.46 (d, *J* = 7.9 Hz, 2H), 7.33–7.23 (m, 4H), 7.22–7.14 (m, 4H), 7.12 (d, *J* = 6.7 Hz, 2H), 5.35 (dd, *J* = 13.6, 4.3 Hz, 1H), 5.13 (dd, *J* = 13.6, 10.3 Hz, 1H), 4.09–3.95 (m, 1H), 2.17 (s, 3H); ^13^C NMR (126 MHz, CDCl_3_): *δ* 171.95, 161.45, 136.50, 131.81, 129.12, 129.02, 128.92, 128.61, 127.91, 126.08, 119.47, 119.41, 80.53, 73.90, 48.59, 13.05; HR-MS (+SI): *m*/*z* calcd for C_18_H_17_N_3_O_3_ [M + H]^+^: 324.13481, found 324.13618.

#### 4.5.2. 3-Methyl-4-(2-nitro-1-(p-tolyl)ethyl)-1-phenyl-1*H*-pyrazol-5-ol (6b)

86% (0.21 g) yield as yellow solid; *R*_f_: 0.29 (30% EtOAc/hexane); M.P.: 168–170 °C; IR (KBr, cm^−1^): 3024, 2920, 1729, 1615, 1594, 1577, 1551, 1499, 1458, 1373, 1261, 1106, 1022; ^1^H NMR (500 MHz, CDCl_3_): *δ* 7.70 (d, *J* = 8.0 Hz, 1H), 7.55 (s, 2H), 7.36 (t, *J* = 11.5 Hz, 5H), 7.23–7.14 (m, 2H), 7.11 (t, *J* = 8.4 Hz, 2H), 7.06 (q, *J* = 8.0 Hz, 2H), 5.48 (dd, *J* = 13.9, 7.9 Hz, 1H), 5.02–4.91 (m, 1H), 4.49 (t, *J* = 7.4 Hz, 1H), 2.31 (s, 3H), 2.15 (s, 3H); ^13^C NMR (126 MHz, CDCl_3_): *δ* 147.2, 137.0, 136.7, 129.5, 128.8, 127.6, 125.4, 77.4, 39.6, 21.0, 10.8; HR-MS (+SI): *m*/*z* calcd for C_19_H_19_N_3_O_3_ [M + H]^+^: 338.15046, found 338.15234.

#### 4.5.3. 4-(1-(4-Methoxyphenyl)-2-nitroethyl)-3-methyl-1-phenyl-1*H*-pyrazol-5-ol (6c)

85% (0.22 g) yield as pale-yellow solid; *R*_f_: 0.30 (30% EtOAc/hexane); M.P.: 170–173 °C; IR (KBr, cm^−1^): 2923, 2853, 2924, 1739, 1613, 1549, 1512, 1497, 1456, 1375, 1304, 1247, 1178, 1113, 1029; ^1^H NMR (500 MHz, CDCl_3_): *δ* 7.44 (d, *J* = 7.6 Hz, 2H), 7.36 (dd, *J* = 14.3, 8.6 Hz, 3H), 7.25 (dd, *J* = 12.8, 5.0 Hz, 3H), 7.14–7.07 (m, 2H), 6.79 (t, *J* = 9.1 Hz, 2H), 5.33 (dd, *J* = 12.7, 8.9 Hz, 1H), 4.88 (dd, *J* = 12.9, 6.8 Hz, 1H), 4.49–4.38 (m, 1H), 3.76 (s, 3H), 2.05 (s, 3H); ^13^C NMR (126 MHz, CDCl_3_): *δ* 158.81, 157.89, 146.79, 135.62, 131.35, 128.87, 128.70, 126.11, 120.80, 114.15, 103.39, 77.01, 62.24, 55.27, 39.34, 38.95, 29.73, 14.18, 10.61; HR-MS (+SI): *m*/*z* calcd for C_19_H_19_N_3_O_4_ [M + H]^+^: 354.14538, found 354.14253.

#### 4.5.4. 4-(1-(4-Fluorophenyl)-2-nitroethyl)-3-methyl-1-phenyl-1*H*-pyrazol-5-ol (6d)

82% (0.19 g) yield as yellow solid; *R*_f_. 0.28 (30% EtOAc/hexane); M.P.: 152–155 °C; IR (KBr, cm^−1^) 3648, 3058, 2916, 2856, 2791, 1939, 1884, 1713, 1615, 1571, 1548, 1507, 1502, 1460, 1304, 1229, 1137, 1076, 1036; ^1^H NMR (500 MHz, CDCl_3_): *δ* 7.40 (dt, *J* = 11.7, 7.2 Hz, 4H), 7.25 (dd, *J* = 14.8, 7.0 Hz, 3H), 7.10 (dd, *J* = 15.5, 8.1 Hz, 1H), 6.95 (t, *J* = 8.5 Hz, 2H), 5.33–5.24 (m, 1H), 4.89 (dd, *J* = 12.9, 6.9 Hz, 1H), 4.45 (t, *J* = 7.8 Hz, 1H), 2.04 (d, *J* = 2.2 Hz, 3H); ^13^C NMR (126 MHz, CDCl_3_): *δ* 163.0, 161.0, 146.5, 135.3, 135.1, 135.1, 129.3, 129.2, 128.9, 126.3, 121.1, 115.7, 115.5, 102.3, 76.8, 60.7, 38.9, 10.4; HR-MS (+SI): *m*/*z* calcd for C_19_H_19_FN_3_O_3_ [M + H]^+^: 357.14887, found 354.28867.

#### 4.5.5. 4-(1-(3-Fluorophenyl)-2-nitroethyl)-3-methyl-1-phenyl-1*H*-pyrazol-5-ol (6e)

80% (0.19 g) yield as yellow solid; *R*_f_: 0.28 (30% EtOAc/hexane); M.P.: 144–145 °C; IR (KBr, cm^−1^): 2923, 1943, 1870, 1713, 1615, 1551, 1495, 1448, 1426, 1375, 1307, 1258, 1144, 1042; ^1^H NMR (500 MHz, CDCl_3_): *δ* 7.22–7.14 (m, 3H), 7.10–7.02 (m, 4H), 6.98 (dd, *J* = 16.1, 8.6 Hz, 1H), 6.88 (dd, *J* = 16.2, 8.1 Hz, 1H), 5.09 (dd, *J* = 12.7, 9.3 Hz, 1H), 4.74 (dd, *J* = 13.0, 6.8 Hz, 1H), 4.37 (t, *J* = 7.8 Hz, 1H), 1.86 (s, 3H); ^13^C NMR (126 MHz, CDCl_3_): *δ* 163.03, 161.07, 146.52, 141.73, 141.70, 129.71, 129.64, 128.21, 124.74, 122.84, 119.21, 114.17, 113.99, 113.63, 113.46, 76.00, 28.90, 13.49, 13.05; HR-MS (+SI): *m*/*z* calcd for C_18_H_16_FN_3_O_3_ [M + H]^+^: 342.12539, found 342.12858.

#### 4.5.6. 4-(1-(4-Chlorophenyl)-2-nitroethyl)-3-methyl-1-phenyl-1*H*-pyrazol-5-ol (6f)

92% (0.23 g) yield as yellow solid; *R*_f_: 0.28–0.31 (30% EtOAc/hexane); M.P.: 166–168 °C; IR (KBr, cm^−1^): 3421, 3066, 2923, 1712, 1613, 1551, 1492, 1457, 1410, 1374, 1308, 1091, 1014; ^1^H NMR (500 MHz, CDCl_3_): *δ* 7.32 (dd, *J* = 16.3, 8.2 Hz, 4H), 7.25–7.16 (m, 4H), 7.08 (t, *J* = 7.2 Hz, 1H), 5.22 (dd, *J* = 12.9, 8.9 Hz, 1H), 4.83 (dd, *J* = 13.0, 6.8 Hz, 1H), 4.41 (t, *J* = 7.8 Hz, 1H), 1.99 (s, 3H); ^13^C NMR (126 MHz, CDCl_3_): *δ* 175.1, 171.5, 161.5, 160.9, 146.7, 137.7, 135.3, 133.3, 129.0, 12.0, 128.9, 128.9, 126.4, 121.1, 102.5, 76.5, 39.0, 10.6; HR-MS (+SI): *m*/*z* calcd for C_18_H_16_ClN_3_O_3_ [M + H]^+^: 358.09584, found 358.09589.

#### 4.5.7. 4-(1-(4-Bromophenyl)-2-nitroethyl)-3-methyl-1-phenyl-1*H*-pyrazol-5-ol (6g)

91% (0.23 g) yield as yellow solid: *R*_f_: 0.33 (30% EtOAc/hexane); M.P.: 171–173 °C; IR (KBr, cm^−1^) 3648, 3064, 2923, 2853, 2588, 1722, 1658, 1552, 1499, 1486, 1261, 1100, 1032, 973, 901, 813, 867, 747, 725, 591, 512, 500; ^1^H NMR (500 MHz, DMSO): *δ* 7.51 (d, *J* = 7.8 Hz, 2H), 7.34–7.18 (m, 7H), 7.02 (s, 1H), 5.24 (s, 1H), 4.99–4.86 (m, 1H), 4.30 (s, 1H), 2.01 (s, 3H); ^13^C NMR (126 MHz, DMSO): *δ* 162.5, 147.4, 147.1, 138.8, 131.9, 129.6, 128.9, 125.6, 121.3, 120.2, 118.8, 103.1, 76.7, 48.0, 29.6, 10.8; HR-MS (+SI): *m*/*z* calcd for C_18_H_16_BrN_3_O_3_ [M + H]^+^: 402.04532, found 404.04949.

#### 4.5.8. 4-(1-(2-Bromophenyl)-2-nitroethyl)-3-methyl-1-phenyl-1*H*-pyrazol-5-ol (6h)

90% (0.22 g) yield as yellow solid; *R*_f_: 0.32 (30% EtOAc/hexane); M.P: 102–104 °C; IR (KBr, cm^−1^): 3430, 2853, 2923, 1691, 1592, 1550, 1497, 1474, 1306, 1108, 1074, 1027, 906, 872, 833, 792, 743, 689, 590; ^1^H NMR (500 MHz, CDCl_3_): *δ* 7.89 (d, *J* = 7.2 Hz, 2H), 7.67 (d, *J* = 7.6 Hz, 1H), 7.54 (dd, *J* = 13.4, 8.0 Hz, 3H), 7.45–7.29 (m, 2H), 7.29–7.22 (m, 1H), 7.13 (dt, *J* = 23.4, 7.5 Hz, 1H), 5.53 (dd, *J* = 12.8, 10.5 Hz, 1H), 5.08 (dd, *J* = 9.8, 5.2 Hz, 1H), 4.79 (dd, *J* = 13.1, 5.1 Hz, 1H), 3.48 (s, 1H), 2.23 (s, 3H); ^13^C NMR (126 MHz, CDCl_3_): *δ* 162.2, 147.9, 137.9, 135.6, 133.0, 130.1, 129.2, 129.0, 128.0, 126.1, 123.5, 123.4, 120.6, 102.6, 75.3, 38.7, 11.2; HR-MS (+SI): *m*/*z* calcd for C_18_H_16_BrN_3_O_3_ [M + H]^+^: 402.04532, found 402.04533.

#### 4.5.9. 3-Methyl-4-(2-nitro-1-(2-(trifluoromethyl)phenyl)ethyl)-1-phenyl-1*H*-pyrazol-5-ol (6i)

85% (0.21 g) yield as yellow solid; *R*_f_: 0.32 (30% EtOAc/hexane); M.P.: 193–195 °C; IR (KBr, cm^−1^): 2921, 2624, 1609, 1593, 1580, 1552, 1455, 1434, 1311, 1220, 1196, 1071, 1062, 975, 904, 842; ^1^H NMR (500 MHz, CDCl_3_): *δ* 8.35 (d, *J* = 7.8 Hz, 1H), 7.67 (d, *J* = 7.9 Hz, 1H), 7.58 (d, *J* = 8.0 Hz, 2H), 7.52 (t, *J* = 7.6 Hz, 1H), 7.38 (t, *J* = 7.6 Hz, 3H), 7.20 (t, *J* = 7.4 Hz, 1H), 5.87–5.79 (m, 1H), 5.00 (dd, *J* = 10.9, 3.9 Hz, 1H), 4.55 (dd, *J* = 13.4, 3.9 Hz, 1H), 3.48 (d, *J* = 7.6 Hz, 1H), 2.17 (s, 3H); ^13^C NMR (126 MHz, DMSO): *δ* 162.5, 148.0, 138.2, 135.4, 132.7, 131.3, 129.0, 127.8, 126.5, 126.1, 126.1, 125.9, 125.6, 123.4, 121.1, 102.4, 75.7, 35.6, 10.5; HR-MS (+SI): *m*/*z* calcd for C_19_H_16_F_3_N_3_O_3_ [M + H]^+^: 392.12220, found 392.12102.

#### 4.5.10. 3-Methyl-4-(2-nitro-1-(4-nitrophenyl)ethyl)-1-phenyl-1*H*-pyrazol-5-ol (6j)

80% (0.19 g) yield as yellow solid; *R*_f_: 0.27 (30% EtOAc/hexane); M.P.: 184–187 °C; IR (KBr, cm^−1^): 3069, 2932, 2853, 1559, 1520, 1406, 1377, 1348, 1305, 1110, 856, 744; ^1^H NMR (500 MHz, CDCl_3_): *δ* 8.16 (d, *J* = 8.3 Hz, 2H), 7.80 (d, *J* = 8.3 Hz, 2H), 7.68 (d, *J* = 7.8 Hz, 2H), 7.58 (s, 1H), 7.41 (t, *J* = 7.6 Hz, 2H), 7.21 (t, *J* = 7.2 Hz, 1H), 5.42 (dd, *J* = 13.2, 8.0 Hz, 1H), 5.26 (dd, *J* = 13.2, 7.5 Hz, 1H), 4.64 (t, *J* = 7.6 Hz, 1H), 2.24 (s, 3H); ^13^C NMR (126 MHz, CDCl_3_): *δ* 146.80, 146.05, 128.11, 124.40, 122.99, 118.68, 75.36, 39.55, 39.46, 39.38, 39.29, 39.21, 39.13, 38.96, 38.79, 38.62, 28.65, 10.09; HR-MS (+SI): *m*/*z* calcd for C_18_H_16_N_4_O_5_ [M + H]^+^: 369.11989, found 369.12377.

#### 4.5.11. 3-Methyl-4-(2-nitro-1-(2-nitrophenyl)ethyl)-1*H*-pyrazol-5-ol (6k)

80% (0.19 g) yield as yellow solid; *R*_f_: 0.25 (30% EtOAc/hexane); M.P.: 124–126 °C; IR (KBr, cm^−1^): 3716, 2925, 2854, 1736, 1595, 1554, 1527, 1499, 1371, 1081, 757, 691; ^1^H NMR (500 MHz, CDCl_3_): *δ* 8.06 (t, *J* = 10.3 Hz, 1H), 7.80 (d, *J* = 8.1 Hz, 1H), 7.48 (dd, *J* = 15.0, 7.4 Hz, 1H), 7.38 (t, *J* = 6.2 Hz, 3H), 7.29–7.21 (m, 3H), 7.13 (t, *J* = 7.2 Hz, 1H), 5.38 (q, *J* = 11.8 Hz, 1H), 5.22–5.15 (m, 1H), 4.89 (dd, *J* = 13.2, 5.5 Hz, 1H), 2.11 (s, 3H); ^13^C NMR (126 MHz, CDCl_3_): *δ* 162.0, 148.8, 135.3, 133.3, 133.2, 130.8, 129.0, 128.5, 126.4, 124.5, 120.8, 101.8, 75.5, 33.8, 10.6; HR-MS (+SI): *m*/*z* calcd for C_18_H_16_N_4_O_5_ [M + H]^+^: 369.11989, found 369.12440.

#### 4.5.12. 4-(1-(5-Hydroxy-3-methyl-1-phenyl-1*H*-pyrazol-4-yl)-2-nitroethyl)benzonitrile (6l)

86% (0.21 g) yield as yellow solid; *R*_f_: 0.30 (30% EtOAc/hexane); M.P.: 166–168 °C; IR (KBr, cm^−1^): 3045, 2886, 2781, 2231, 1945, 1812, 1714, 1610, 1595, 1579, 1498, 1458, 1374, 1348, 1304, 1251, 1189, 1155, 1074, 1042; ^1^H NMR (500 MHz, CDCl_3_): *δ* 7.56 (s, 4H), 7.42 (d, *J* = 8.0 Hz, 2H), 7.26 (t, *J* = 7.7 Hz, 3H), 7.14 (t, *J* = 7.4 Hz, 1H), 5.33–5.25 (m, 1H), 4.94 (dd, *J* = 13.2, 6.9 Hz, 1H), 4.51 (t, *J* = 7.7 Hz, 1H), 2.09 (s, 3H); ^13^C NMR (126 MHz, CDCl_3_): *δ* 161.4, 146.7, 144.7, 135.3, 132.6, 129.0, 128.5, 126.5, 121.0, 118.6, 111.2, 101.7, 76.0, 39.5, 10.5; HR-MS (+SI): *m*/*z* calcd for C_19_H_16_N_4_O_3_ [M + H]^+^: 349.13006, found 349.13356.

### 4.6. Synthesis of heteroaryl substituted pyrazol-5-ol derivatives (8a–f)

Substituted nitro-ethylene conjugated heteroaryl compounds (7, 1 mmol) and hydrazine hydrate/phenylhydrazine (2 or 5, 1 mmol) were dissolved in water (5 mL) along with graphene oxide (GO, fixed at 0.05 wt%) and transferred into a round bottom flask (RBF, 50 mL) under continuous stirring. Ethyl acetoacetate (3, 1 mmol) was then added drop-wise over 2–4 min to the same RBF. The reaction mixture was subjected to microwave irradiation (180 W) until a light brown to whitish-yellow precipitate (ppt) began to form, as confirmed by TLC. The work-up procedure remained same as described earlier for the synthesis of pyrazol-5-ol (4). The desired phenyl pyrazol-5-ol conjugated heterocyclic derivatives (8a–f) obtained as white to yellowish solids with a good yield (80–92%).

#### 4.6.1. 4-(1-(Furan-2-yl)-2-nitroethyl)-3-methyl-1*H*-pyrazol-5-ol (8a)

92% (0.21 g) yield as yellow solid. *R*_f_: 0.40 (70% EtOAc/hexane); M.P.: 185–188 °C; IR (KBr, cm^−1^): 3428, 2957, 2924, 2852, 1723, 1630, 1548, 1465, 1426, 1276, 1220, 1189, 1076; ^1^H NMR (500 MHz, CDCl_3_): *δ* 2.13 (s, 3H, C3–CH_3_), 4.77 (t, *J* = 7.7 Hz, 1H, ArC*H*), 5.05–4.95 (m, 2H, C*H*_*2*_NO_2_), 6.13 (s, 1H, ArH), 6.29 (s, 1H, ArH), 7.49–7.43 (m, 1H, ArH); ^13^C NMR (126 MHz, CDCl_3_): *δ* 9.3 (C6), 28.7 (C7), 96.0 (C8), 105.9 (C16), 109.7 (C15), 113.4 (C13), 141.0 (C14), 151.3 (C3), 158.9 (C12), 172.3 (C5); HRMS (+SI): *m*/*z* calcd for C_10_H_11_N_3_O_4_ [M + H]^+^: 238.08278, found 238.06609.

#### 4.6.2. 3-Methyl-4-(2-nitro-1-(thiophen-2-yl)ethyl)-1*H*-pyrazol-5-ol (8b)

90% (0.22 g) yield as white solid. *R*_f_: 0.41 (70% EtOAc/hexane); M.P.: 179–181 °C; IR (KBr, cm^−1^): 3230, 2852, 2923, 1605, 1551, 1430, 1376, 1213, 1038, 967, 909, 872; ^1^H NMR (500 MHz, CDCl_3_): *δ* 2.17 (s, 3H, C3–CH_3_), 4.93–4.86 (m, 1H, ArC*H*), 4.96 (dd, *J* = 12.7, 6.7 Hz, 1H, C*H*_*2*_NO_2_), 5.18 (dd, *J* = 12.6, 9.0 Hz, 1H, C*H*_*2*_NO_2_), 6.95–6.89 (m, 1H, ArH), 6.98 (d, *J* = 2.6 Hz, 1H, ArH), 7.17 (d, *J* = 5.0 Hz, 1H, ArH); ^13^C NMR (126 MHz, CDCl_3_): *δ* 10.3 (6), 31.0 (C7), 77.7 (C8), 124.8 (C13), 125.1 (C14), 127.2 (C15,16), 139.8 (C3), 142.1 (C12), 160.6 (C5); HR-MS (+SI): *m*/*z* calcd for C_10_H_11_N_3_O_3_S [M + H]^+^: 254.05993, found 254.05197.

#### 4.6.3. 3-Methyl-4-(2-nitro-1-(quinolin-7-yl)ethyl)-1*H*-pyrazol-5-ol (8c)

88% (0.26 g) yield as white solid; *R*_f_: 0.38 (70% EtOAc/hexane); M.P.: 220–225 °C; IR (KBr, cm^−1^): 3394, 2923, 2851, 1715, 1598, 1552, 1511, 1428, 1375, 1150, 1029, 967, 872, 646; ^1^H NMR (500 MHz, CDCl_3_): *δ* 1.99 (s, 3H, C3–CH_3_), 4.96 (dd, *J* = 13.1, 6.0 Hz, 1H, C*H*_*2*_NO_2_), 5.39–5.31 (m, 1H, C*H*_*2*_NO_2_), 5.57–5.49 (m, 1H, C*H*_*2*_NO_2_), 7.41 (dd, *J* = 14.9, 7.1 Hz, 2H, ArH), 7.52 (t, *J* = 7.4 Hz, 1H ArH), 7.66–7.56 (m, 2H, ArH), 7.77 (d, *J* = 8.1 Hz, 1H, ArH), 7.89 (d, *J* = 8.0 Hz, 1H), 8.26 (d, *J* = 8.5 Hz, 1H); ^13^C NMR (126 MHz, CDCl_3_): *δ* 10.2 (C6), 34.6 (C7), 98.5 (C8), 122.3 (C4), 125.2 (C13), 126.6 (C14), 127.8 (C15), 129.0 (C17, C16), 130.6 (C17), 133.7 (C18), 134.5 (C19), 138.9 (C3), 160.8 (C12), 174.0 (C5); HR-MS (+SI): *m*/*z* calcd for C_15_H_14_N_4_O_3_ [M + H]^+^: 299.11441, found 299.11910.

#### 4.6.4. 4-(1-(Furan-2-yl)-2-nitroethyl)-3-methyl-1-phenyl-1*H*-pyrazol-5-ol (8d)

83% (0.20 g) yield as yellow solid; *R*_f_: 0.26 (30% EtOAc/hexane); M.P.: 177–179 °C; IR (KBr, cm^−1^): 2926, 2926, 2854, 1716, 1596, 1557, 1498, 1411, 1375, 1261, 1095, 1019; ^1^H NMR (500 MHz, CDCl_3_): *δ* 7.67 (d, *J* = 8.3 Hz, 1H), 7.44 (d, *J* = 8.0 Hz, 2H), 7.38–7.22 (m, 5H), 7.12 (dt, *J* = 14.6, 7.3 Hz, 2H), 6.22 (s, 1H), 6.13 (s, 1H), 5.08 (dd, *J* = 13.2, 8.7 Hz, 1H), 4.86 (dd, *J* = 13.2, 6.6 Hz, 1H), 4.67 (t, *J* = 7.6 Hz, 1H), 3.56 (d, *J* = 4.5 Hz, 1H), 2.07 (s, 3H); ^13^C NMR (126 MHz, CDCl_3_): *δ* 160.9, 150.8, 147.2, 142.0, 135.3, 129.0, 128.9, 126.6, 121.0, 110.6, 107.1, 100.9, 74.8, 33.0, 10.7; HR-MS (+SI): *m*/*z* calcd for C_16_H_15_N_3_O_4_ [M + H]^+^: 314.11408, found 314.11153.

#### 4.6.5. 3-Methyl-4-(2-nitro-1-(thiophen-2-yl)ethyl)-1-phenyl-1*H*-pyrazol-5-ol (8e)

80% (0.19 g) yield as yellow solid; *R*_f_: 0.28 (30% EtOAc/hexane); M.P.: 180–184 °C; IR (KBr, cm^−1^) 3341, 2782, 1712, 1592, 1548, 1496, 1457, 1426, 1376, 1314, 1272, 1236, 1159, 1113, 1023; ^1^H NMR (500 MHz, CDCl_3_): *δ* 7.34 (d, *J* = 7.8 Hz, 2H), 7.26 (s, 1H), 7.18 (dd, *J* = 17.5, 10.3 Hz, 3H), 7.12–7.04 (m, 2H) 6.92 (d, *J* = 10.3 Hz, 1H), 6.86 (dd, *J* = 11.7, 8.0 Hz, 1H), 5.19 (dd, *J* = 23.9, 12.5 Hz, 1H), 4.80 (dd, *J* = 12.6, 6.0 Hz, 1H), 4.77–4.72 (m, 1H), 2.20 (s, 1H), 2.00 (s, 3H); ^13^C NMR (126 MHz, CDCl_3_): *δ* 171.75, 147.51, 142.20, 136.21, 129.64, 127.76, 126.95, 125.77, 125.44, 121.42, 119.99, 119.77, 103.70, 77.43, 35.26, 11.30; HR-MS (+SI): *m*/*z* calcd for C_16_H_15_N_3_O_3_S [M + H]^+^: 330.09123, found 330.09344.

#### 4.6.6. 3-Methyl-4-(2-nitro-1-(quinolin-7-yl)ethyl)-1-phenyl-1*H*-pyrazol-5-ol (8f)

82% (0.20 g) yield as yellow solid; *R*_f_: 0.33 (30% EtOAc/hexane); M.P.: 207–210 °C; IR (KBr, cm^−1^): 3700, 2922, 1734, 1550, 1497, 1457, 1407, 1373, 1309, 1029, 969, 838, 778, 752, 690; ^1^H NMR (500 MHz, CDCl_3_): *δ* 8.11 (d, *J* = 8.4 Hz, 1H), 7.84 (d, *J* = 8.0 Hz, 1H), 7.71 (d, *J* = 8.2 Hz, 1H), 7.60–7.46 (m, 4H), 7.33–7.25 (m, 4H), 7.07 (t, *J* = 7.5 Hz, 2H), 6.98 (t, *J* = 7.3 Hz, 1H), 5.37 (dt, *J* = 14.2, 9.8 Hz, 2H), 4.72 (dd, *J* = 12.1, 4.6 Hz, 1H), 2.04 (d, *J* = 6.6 Hz, 1H), 2.03 (s, 2H); ^13^C NMR (126 MHz, CDCl_3_): *δ* 171.8, 161.7, 146.7, 135.3, 134.1, 134.0, 134.0, 130.7, 129.3, 128.9, 128.8, 128.3, 126.8, 126.3, 125.9, 125.3, 125.3, 122.5, 121.1, 101.9, 75.9, 60.7, 34.9, 10.8; HR-MS (+SI): *m*/*z* calcd for C_21_H_18_N_4_O_3_ [M + H]^+^: 375.14571, found 374.15615.

### 4.7. Cytotoxicity assay, *in vitro*

A cell viability assay, using 3-(4,5-dimethylthiazole)-2,5-diphenyltetraazolium bromide (MTT) was conducted to evaluate the anticancer activity of synthesized pyrazol-5-ol derivatives against human lung cancer cells (A549). Cells (7000–10,000 cells per well) seeded in 96-well culture plates (Tarsons India Pvt. Ltd) using DMEM medium supplemented with 10% FBS and allowed to grow 24 h or overnight at 37 °C in a 5% CO_2_ incubator. The synthesized pyrazol-5-ol derivatives (4, 6 and 8 series) were dissolved separately in complete DMEM at varying concentrations (0–125 μM) for cytotoxicity activity. Control cells maintained in complete DMEM without any test compounds. After incubation for 48 h, the culture media containing the derivatives precisely replaced with fresh DMEM media (10% FBS) and MTT dye solution (0.5 mg ml^−1^ in serum free culture medium) was added to each well. The plates were incubated at 37 °C for 4 h, allowing the formation of purple forzman crystals, indicative of viable cells. Following incubation, the medium carefully removed, and the wells washed with phosphate-buffer saline (PBS, Hi-Media, India Pvt. Ltd). Subsequently, 200 μl of DMSO was added to dissolve the formazan crystals. The plate was covered and genetly agitated at room temperature for 10 min. to ensure complete dissolution. Absorbance was measured spectrophotometrically at 595 nm using synergy (H1) hybrid micro-plate reader (BioTek, USA). The percentage of cell viability was calculated as previously reported.^80^ Experiments were performed in triplicates, and mean values obtained to minimize errors. Cytotoxicity (IC_50_ value) determined from the percentage cell viability *vs.* concentration response curve, where IC_50_ represents the concentration required to reduce MTT reduction by 50% compared to the untreated control. A 5–10% error bar indicates the uncertainty associated with each value.

### 4.8. Molecular docking

A molecular docking study conducted to identify the putative binding modes of Pyrazol-5-ol derivatives (4, 6 and 8 series). All docking calculations performed using the Glide (Grid-Based Ligand Docking with Energetics) program,^77,81^ integrated within the Schrödinger molecular modelling suite (Schrödinger, LLC, New York, NY, 2018). For this purpose, the crystal structure of Epidermal Growth Factor Receptor (EGFR) tyrosine kinase in complex with the 4-anilinoquinazoline inhibitor erlotinib (PDB ID: 1M17) retrieved from the Protein Data Bank (RCSB PDB) and processed using the protein preparation wizard module. All crystallographic water molecules removed, as no conserved interaction with the target were reported. Missing hydrogen atoms/side chains added to the proteins and all atom force field (OPSL-2005) charges with types were assigned. The structure was then energy minimized until the root mean square deviation (RMSD) of non-hydrogen atoms reached 0.3 Å. The shape and properties of active site for ligand binding, was generated using the receptor grid generation protocol. A grid box (12 × 12 × 12 Å) was placed around the centroid of the co-crystallized ligand, ensuring sufficient space for ligand exploration. The 2D chemical structures of pyrazol-5-ol derivatives initially drawn using ChemDraw software (PerkinElmer, Cambridge, USA) and subsequently converted to 3D geometrics using build panel in maestro. The 3D structures were refined through the Ligand Preparation (LigPrep) protocol, ensuring correct protonation states and atom types. Partial atomic charges were assigned using the OPLS-2005 force field, followed by energy minimization to obtain a single low energy 3D structure for each each molecule. Following setup, the pyrazole derivatives were subjected to molecular docking against EGFR tyrosine kinase target using the extra precision (GlideXP) scoring function. This method generates optimized ligand poses within the active site of EGFR. The resulting docking poses visualized to assess the spatial fit of the ligands, and thermodynamic interactions with the in the active site of amino acid residues quantitatively analysed using Maestro's Pose Viewer utility.

## Author contributions

Dhruvi Chaudhari: formal analysis, investigation, methodology, resources, visualization, validation. Sarmita Jana: formal analysis, investigation, methodology, resources, visualization, validation, writing – original draft. Vijay M. Khedkar: formal analysis, investigation, methodology, resources, validation. Sneha Nair: formal analysis, investigation, methodology, software, writing – original draft. Kushan Parikh: methodology, supervision, writing – original draft, writing – review & editing. Shanta Raj Lakshmi: conceptualization, funding acquisition, methodology, project administration, supervision, data curation, writing – original draft, writing – review & editing.

## Conflicts of interest

The authors declare that they have no known competing financial interests or personal relationships that could have influenced the work reported in this manuscript. This article does not contain any studies involving human or animals subjects performed by any of the authors.

## Supplementary Material

RA-015-D5RA06014A-s001

## Data Availability

Additional datasets or raw data, are available from the corresponding author upon reasonable request. All data supporting the findings of this study are available within the article and its supplementary information (SI). Supplementary information: XPS (with C 1s and O 1s graphical representation) and XRD obtained parameters in tabular form, graphical representation of % cell viability & molecular docking data against EGFR tyrosine kinase and analytical data (^1^H NMR, ^13^C NMR and FT-IR) of synthesized all pyrazol-5-ol derivatives (4, 6 and 8 series). See DOI: https://doi.org/10.1039/d5ra06014a.
