# Supplementary material for: Microwave-assisted multi-component green synthesis of bioactive pyrazol-5-ol and its derivatives using graphene oxide as a recyclable catalyst: a route to EGFR inhibitors
Source: RSC Adv. 2025 Nov 10;15(51):43542–63. doi: 10.1039/d5ra06014a (PMC12598462; doi:10.1039/d5ra06014a)
Supplement: RA-015-D5RA06014A-s001 [file RA-015-D5RA06014A-s001.pdf]

Supplementary Information (SI) for RSC Advances.
This journal is © The Royal Society of Chemistry 2025

Supplementary Information

"Microwave-Assisted Multi-Component Green Synthesis of Bioactive Pyrazol-5-ol and its Derivatives Using Graphene Oxide as Recyclable Catalyst: A Route to EGFR Inhibitors"

Dhruvi Chaudhari[a], Sarmita Jana[b], Vijay M. Khedkar[c] Sneha Nair[d], Kushan Parikh[a,], Shanta Raj Lakshmi[a,*]*

[a]Department of Chemical Science, Faculty of Applied Sciences, Parul University, Vadodara – 391 760, India

[b]Department of Life Sciences, Faculty of Applied Sciences, Parul University, Vadodara – 391 760, India

[c]School of Pharmacy, Vishwakarma University, Pune – 411 048, India

[d]Department of Chemistry, L. J. Institute of Applied Sciences, L. J. University, Ahmedabad – 382 210, India

rajlakshmi.shanta0@gmail.com, kamser2057@gmail.com

Supplementary Figures:

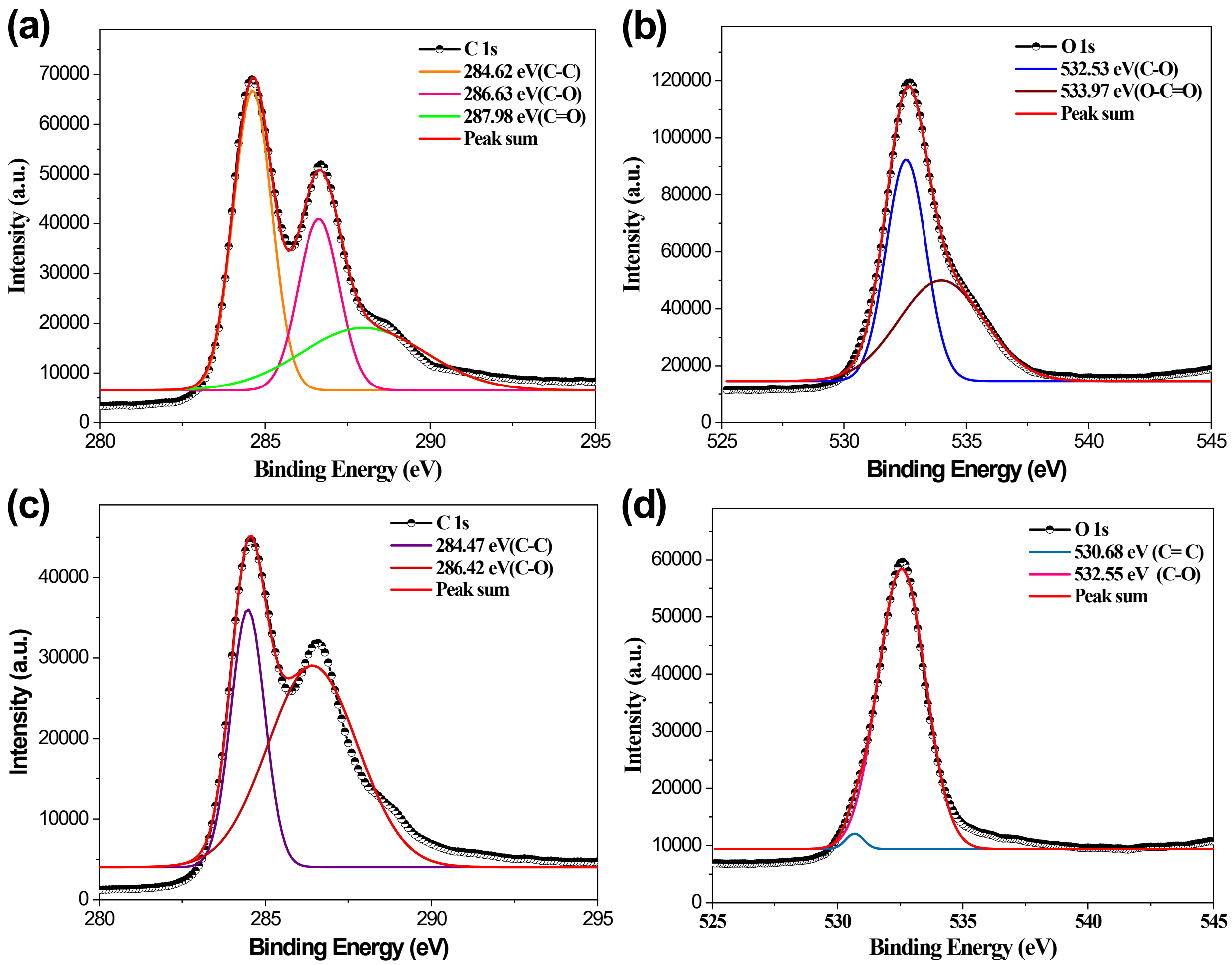

Figure S1. High-resolution C 1s (*a* & *c*) and O 1s (*b* & *d*) XPS spectra of, (*a*, *b*) pure GO (before Cycle I) and (*c*, *d*) GO after Cycle V.

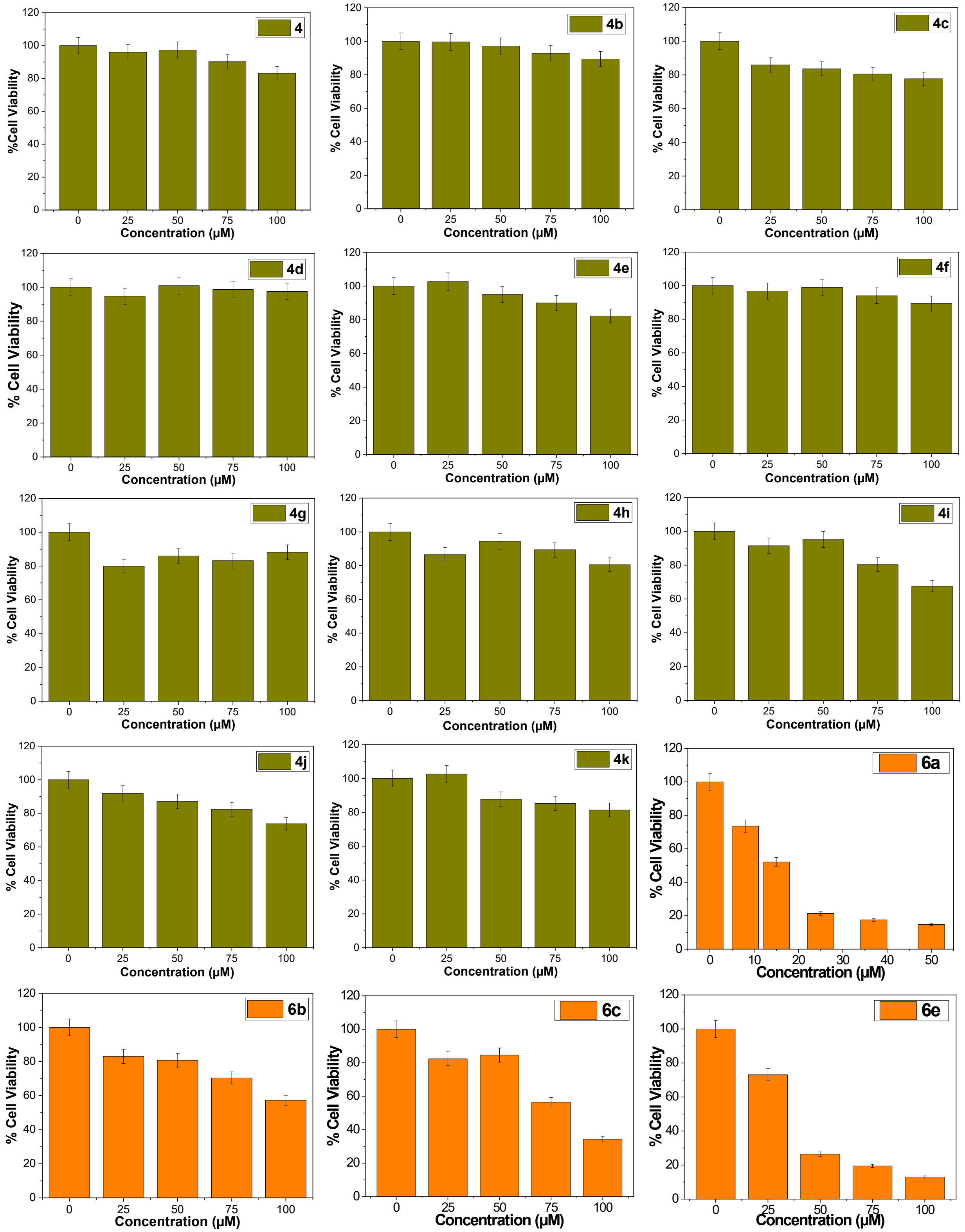
4
%Cell Viability
Concentration (μM)
4b
% Cell Viability
Concentration (μM)
4c
% Cell Viability
Concentration (μM)
4d
% Cell Viability
Concentration (μM)
4e
% Cell Viability
Concentration (μM)
4f
% Cell Viability
Concentration (μM)
4g
% Cell Viability
Concentration (μM)
4h
% Cell Viability
Concentration (μM)
4i
% Cell Viability
Concentration (μM)
4j
% Cell Viability
Concentration (μM)
4k
% Cell Viability
Concentration (μM)
6a
% Cell Viability
Concentration (μM)
6b
% Cell Viability
Concentration (μM)
6c
% Cell Viability
Concentration (μM)
6e
% Cell Viability
Concentration (μM)

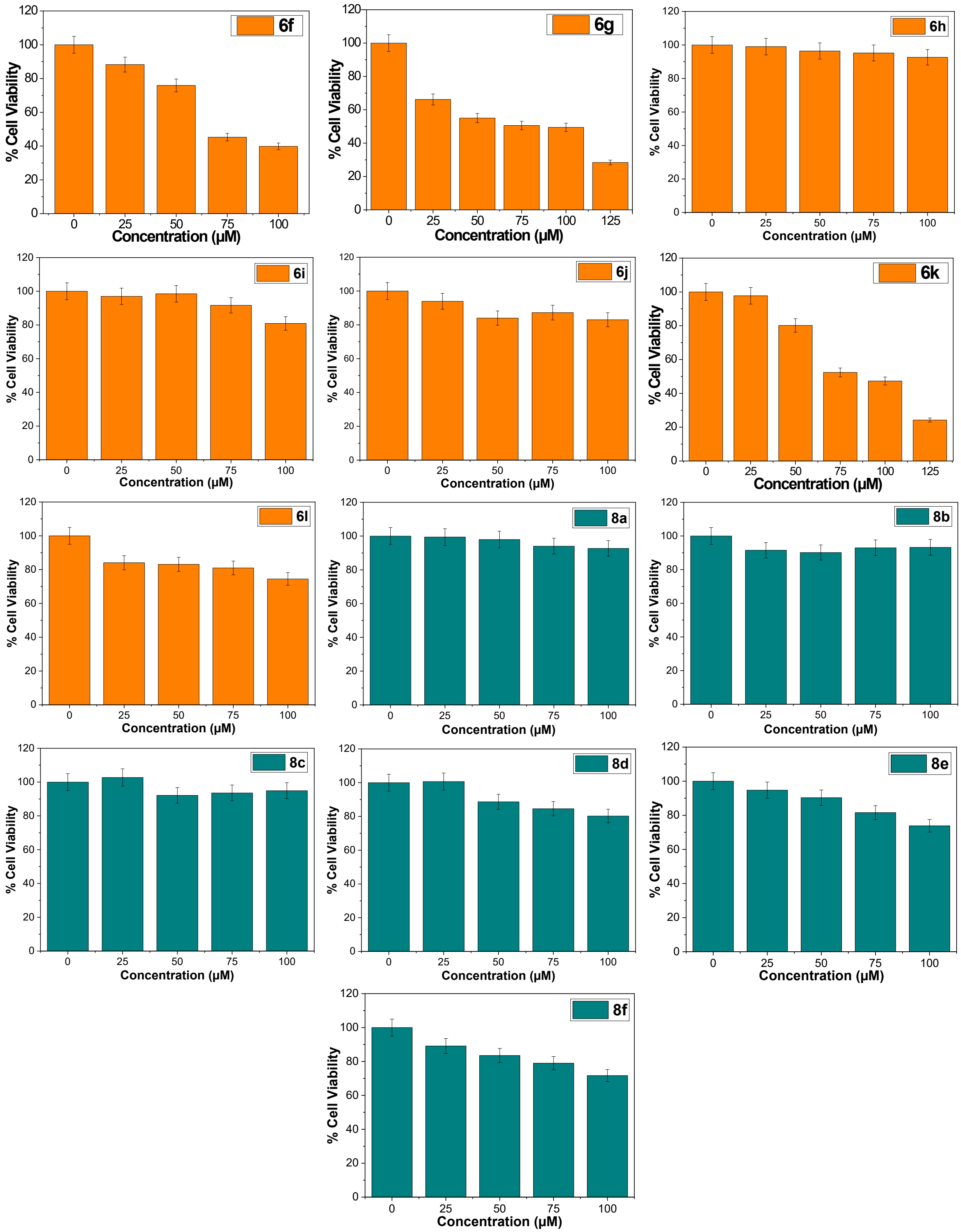

Figure S2. Cell viability (%) *vs* concentration of pyrazol-5-ol derivatives (**4**,**6** & **8** series).

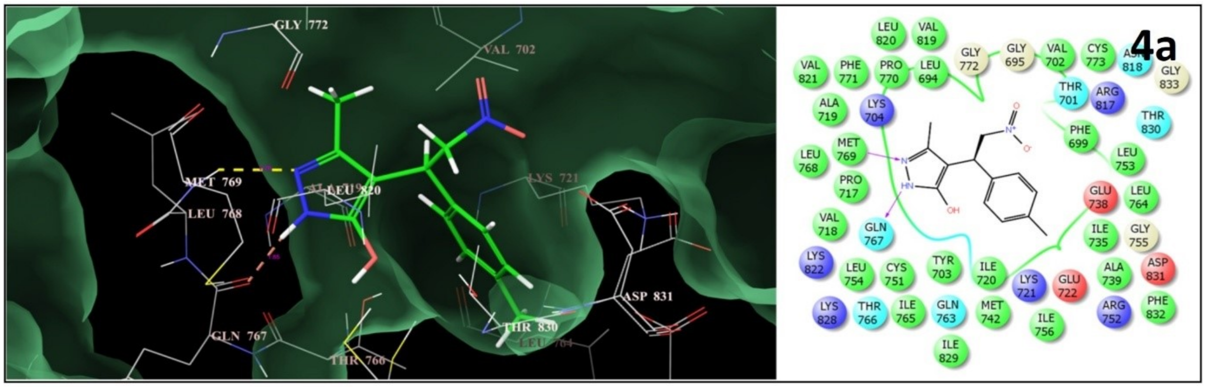
4a
GLY 772
VAL 702
MET 769
LEU 768
LEU 820
LYS 721
ASP 831
THR 830
GLN 767
THR 766
LEU 820
VAL 819
GLY 772
GLY 695
VAL 702
CYS 773
GLY 833
VAL 821
PHE 771
PRO 770
LEU 694
THR 701
ARG 817
ALA 719
LYS 704
THR 830
PHE 699
MET 769
LEU 768
LEU 753
PRO 717
GLU 738
LEU 764
VAL 718
GLN 767
ILE 735
GLY 755
LYS 822
LEU 754
CYS 751
TYR 703
ILE 720
LYS 721
GLU 722
ALA 739
ASP 831
LYS 828
THR 766
ILE 765
GLN 763
MET 742
ILE 756
ARG 752
PHE 832
ILE 829

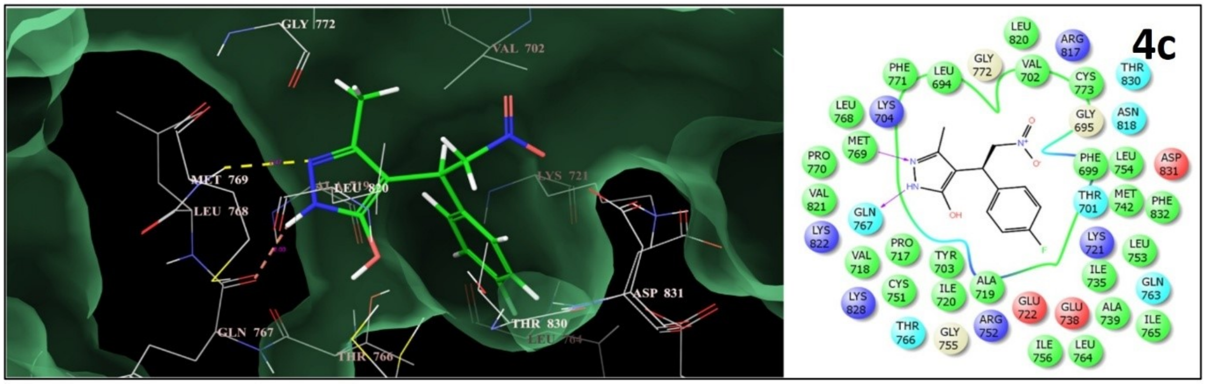
4c
GLY 772
VAL 702
MET 769
LEU 768
LEU 820
LYS 721
ASP 831
THR 830
GLN 767
THR 766
LEU 820
ARG 817
PHE 771
LEU 694
GLY 772
VAL 702
CYS 773
THR 830
LEU 768
LYS 704
GLY 695
ASN 818
MET 769
PRO 770
PHE 699
LEU 754
ASP 831
VAL 821
THR 701
MET 742
PHE 832
GLN 767
LYS 822
LYS 721
LEU 753
PRO 717
VAL 718
TYR 703
ILE 735
GLN 763
CYS 751
ALA 719
ILE 720
LYS 828
GLU 722
GLU 738
ALA 739
ILE 765
THR 766
GLY 755
ARG 752
ILE 756
LEU 764

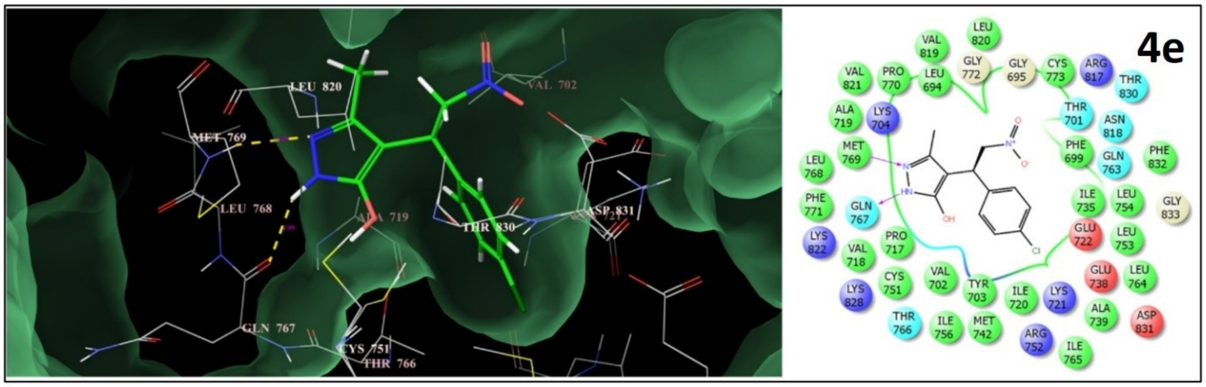
4e
LEU 820
VAL 702
MET 769
LEU 768
ALA 719
THR 830
ASP 831
GLN 767
CYS 751
THR 766
LEU 820
VAL 819
VAL 821
PRO 770
LEU 694
GLY 772
GLY 695
CYS 773
ARG 817
THR 830
THR 701
ALA 719
LYS 704
ASN 818
MET 769
PHE 699
PHE 832
LEU 768
GLN 763
PHE 771
GLN 767
ILE 735
LEU 754
GLY 833
PRO 717
GLU 722
LYS 822
VAL 718
LEU 753
CYS 751
VAL 702
TYR 703
GLU 738
LEU 764
LYS 828
ILE 720
LYS 721
ALA 739
THR 766
ILE 756
MET 742
ARG 752
ASP 831
ILE 765

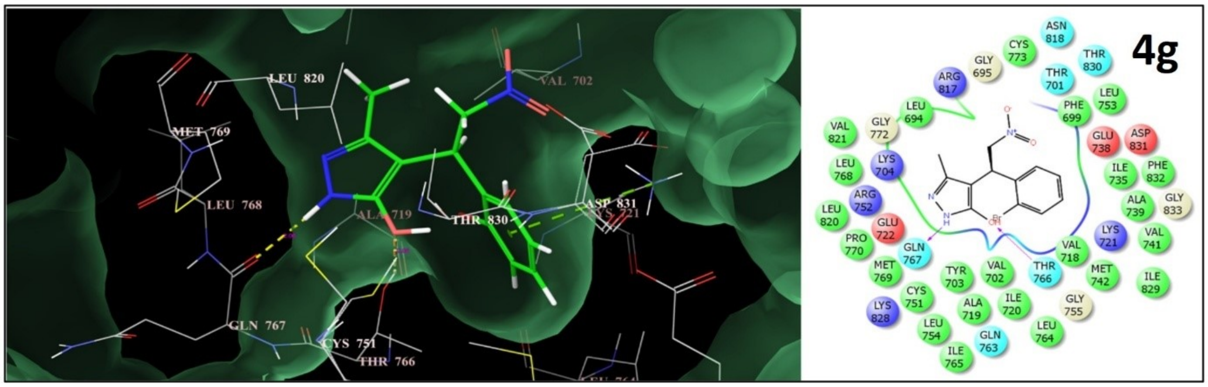
4g
LEU 820
VAL 702
MET 769
LEU 768
ALA 719
THR 830
ASP 831
LYS 721
GLN 767
CYS 751
THR 766
ASN 818
CYS 773
THR 830
GLY 695
THR 701
ARG 817
LEU 753
PHE 699
VAL 821
GLY 772
LEU 694
ASP 831
GLU 738
LEU 768
LYS 704
ILE 735
PHE 832
ARG 752
ALA 739
GLY 833
LEU 820
GLU 722
LYS 721
VAL 741
VAL 718
PRO 770
GLN 767
THR 766
MET 769
TYR 703
VAL 702
MET 742
ILE 829
CYS 751
ALA 719
ILE 720
GLY 755
LYS 828
LEU 754
GLN 763
LEU 764
ILE 765

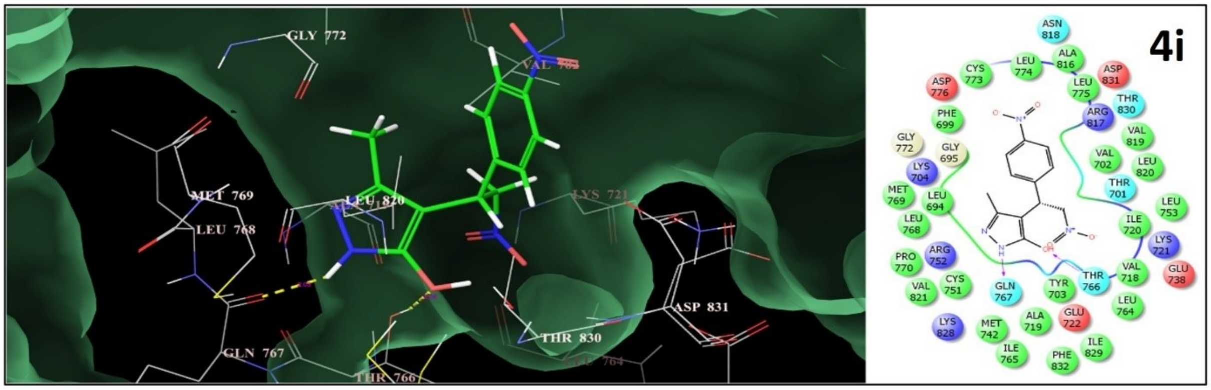
4i

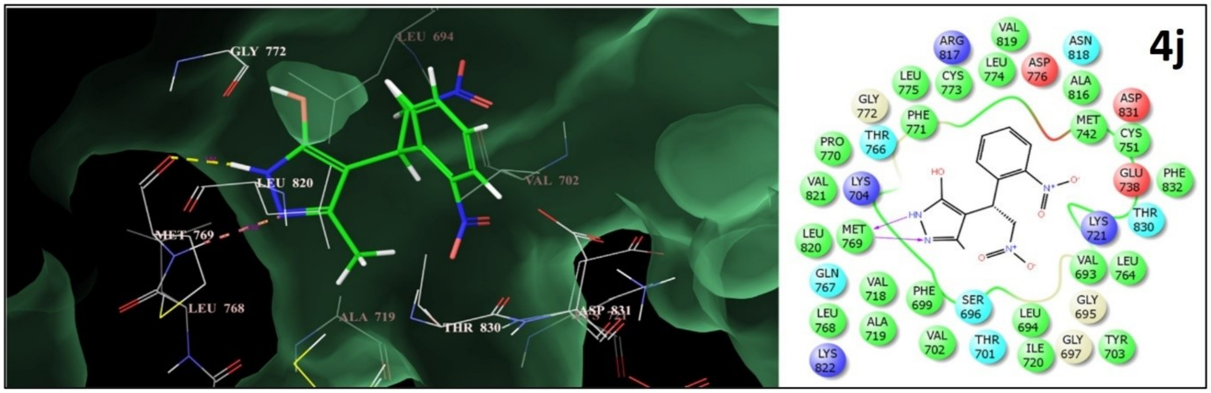
4j

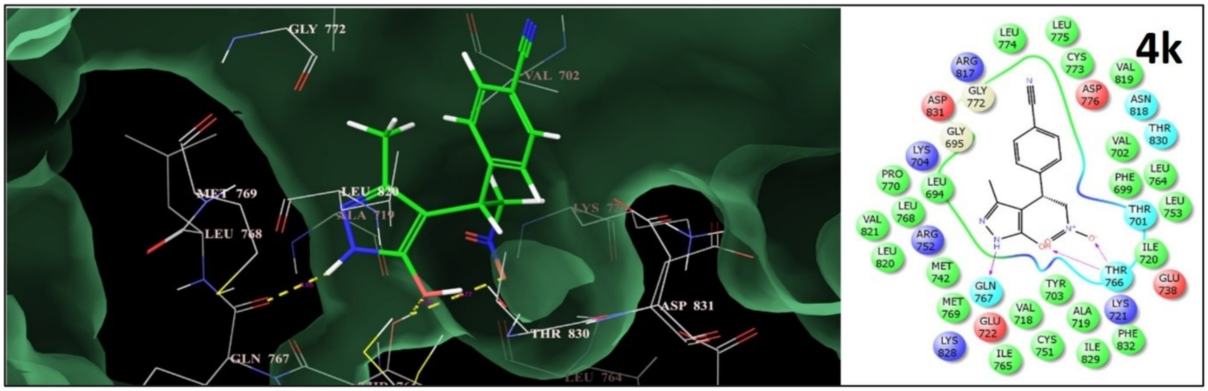
4k

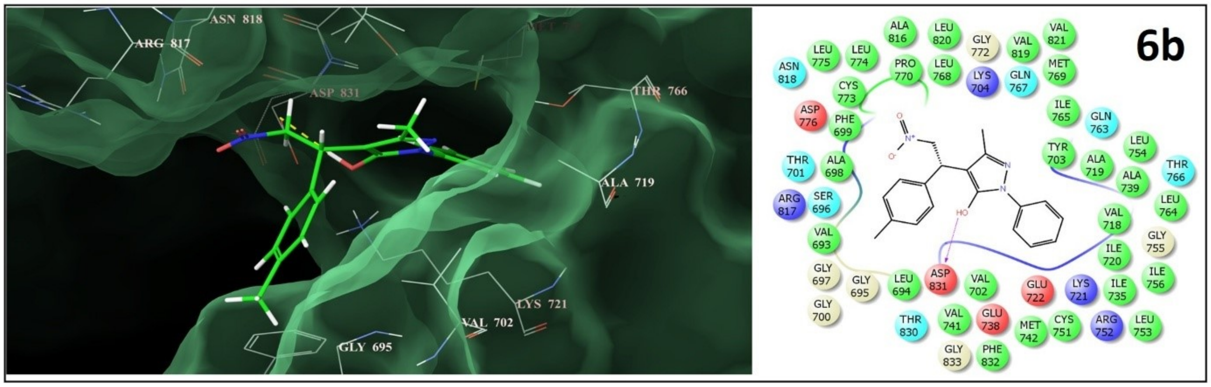
6b

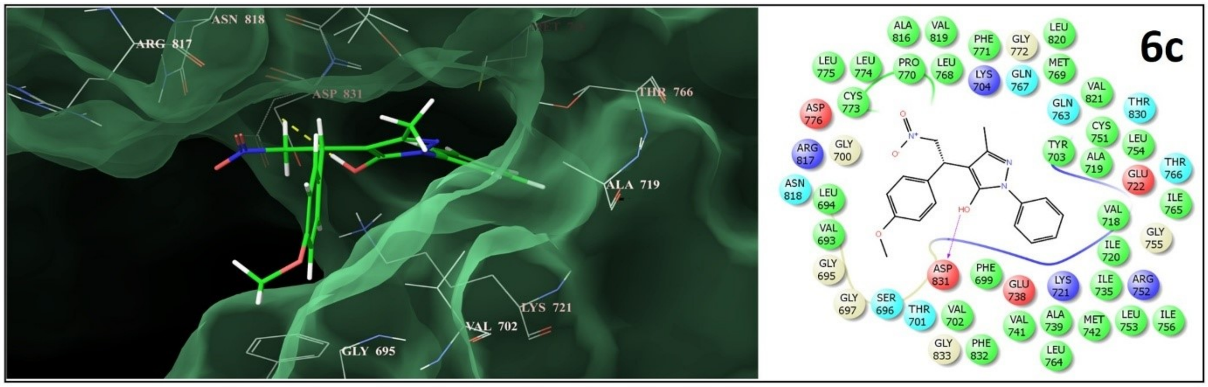
6c
ASN 818
ARG 817
ASP 831
THR 766
ALA 719
LYS 721
VAL 702
GLY 695
ALA 816
VAL 819
PHE 771
GLY 772
LEU 820
LEU 775
LEU 774
PRO 770
LEU 768
LYS 704
GLN 767
MET 769
VAL 821
CYS 773
GLN 763
THR 830
ASP 776
CYS 751
LEU 754
ARG 817
GLY 700
TYR 703
ALA 719
THR 766
GLU 722
ASN 818
LEU 694
ILE 765
VAL 718
VAL 693
GLY 755
ILE 720
GLY 695
ASP 831
PHE 699
GLU 738
LYS 721
ILE 735
ARG 752
GLY 697
SER 696
THR 701
VAL 702
VAL 741
ALA 739
MET 742
LEU 753
ILE 756
GLY 833
PHE 832
LEU 764

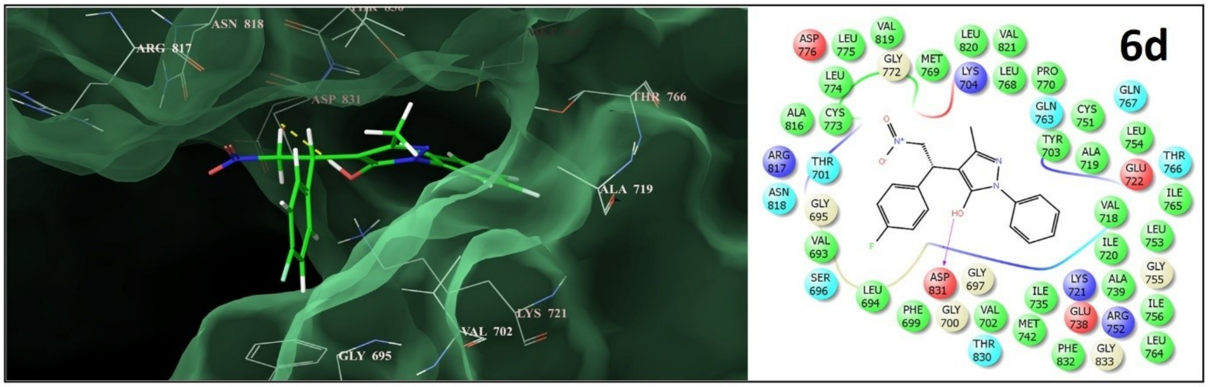
6d
ASN 818
ARG 817
ASP 831
THR 766
ALA 719
LYS 721
VAL 702
GLY 695
ASP 776
LEU 775
VAL 819
LEU 820
VAL 821
GLY 772
MET 769
LYS 704
LEU 768
PRO 770
LEU 774
GLN 767
ALA 816
CYS 773
GLN 763
CYS 751
LEU 754
ARG 817
THR 701
TYR 703
ALA 719
THR 766
GLU 722
ASN 818
ILE 765
GLY 695
VAL 718
LEU 753
VAL 693
ILE 720
SER 696
ASP 831
GLY 697
GLY 755
LEU 694
ILE 735
LYS 721
ALA 739
PHE 699
GLY 700
VAL 702
MET 742
GLU 738
ARG 752
ILE 756
THR 830
PHE 832
GLY 833
LEU 764

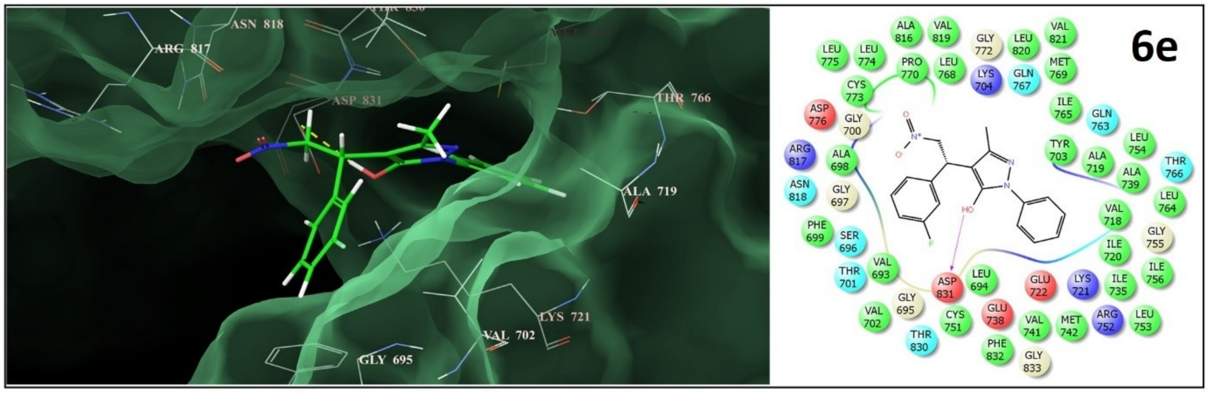
6e
ASN 818
ARG 817
ASP 831
THR 766
ALA 719
LYS 721
VAL 702
GLY 695
ALA 816
VAL 819
GLY 772
LEU 820
VAL 821
LEU 775
LEU 774
PRO 770
LEU 768
LYS 704
GLN 767
MET 769
CYS 773
ILE 765
GLN 763
ASP 776
GLY 700
LEU 754
ARG 817
ALA 698
TYR 703
ALA 719
ALA 739
THR 766
ASN 818
GLY 697
LEU 764
VAL 718
PHE 699
SER 696
GLY 755
ILE 720
THR 701
VAL 693
ILE 756
ASP 831
LEU 694
GLU 722
LYS 721
ILE 735
GLY 695
VAL 702
CYS 751
GLU 738
VAL 741
MET 742
ARG 752
LEU 753
THR 830
PHE 832
GLY 833

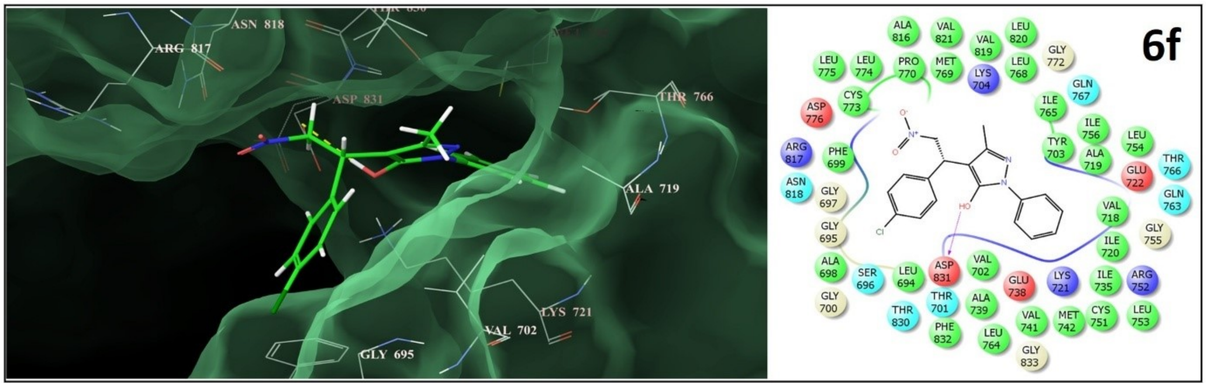
6f
ASN 818
ARG 817
ASP 831
THR 766
ALA 719
LYS 721
VAL 702
GLY 695
ALA 816
VAL 821
VAL 819
LEU 820
GLY 772
LEU 775
LEU 774
PRO 770
MET 769
LYS 704
LEU 768
GLN 767
CYS 773
ILE 765
ASP 776
ILE 756
LEU 754
ARG 817
PHE 699
TYR 703
ALA 719
THR 766
GLU 722
ASN 818
GLY 697
GLN 763
VAL 718
GLY 695
GLY 755
ILE 720
ALA 698
SER 696
LEU 694
ASP 831
VAL 702
GLU 738
LYS 721
ILE 735
ARG 752
GLY 700
THR 701
ALA 739
VAL 741
MET 742
CYS 751
LEU 753
THR 830
PHE 832
LEU 764
GLY 833

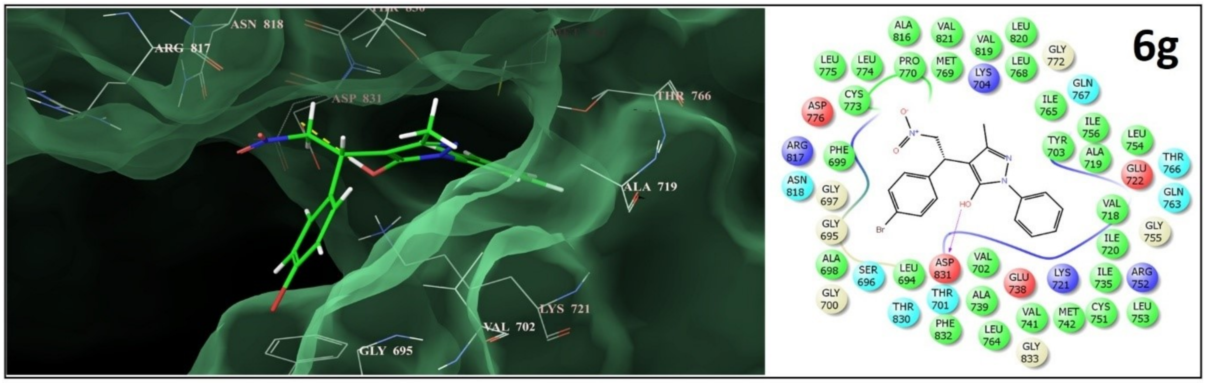
6g
ARG 817
ASN 818
ASP 831
THR 766
ALA 719
LYS 721
VAL 702
GLY 695

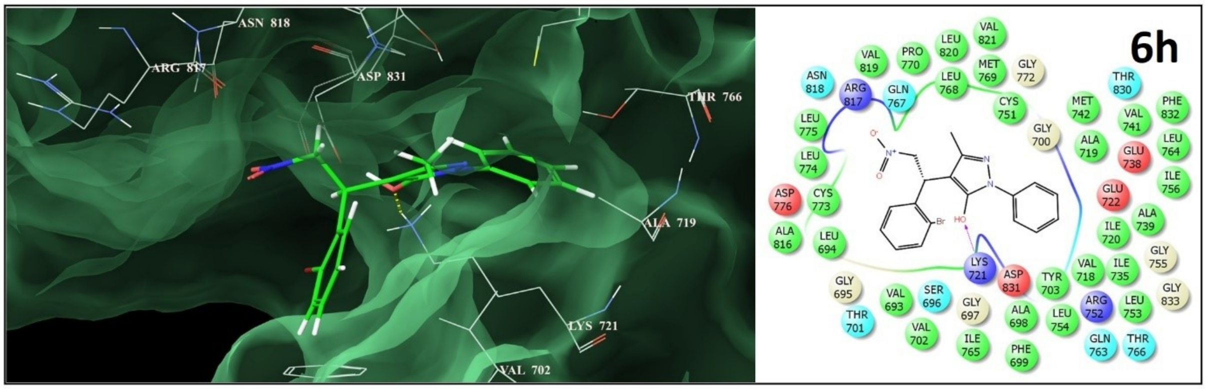
6h
ASN 818
ARG 817
ASP 831
THR 766
ALA 719
LYS 721
VAL 702

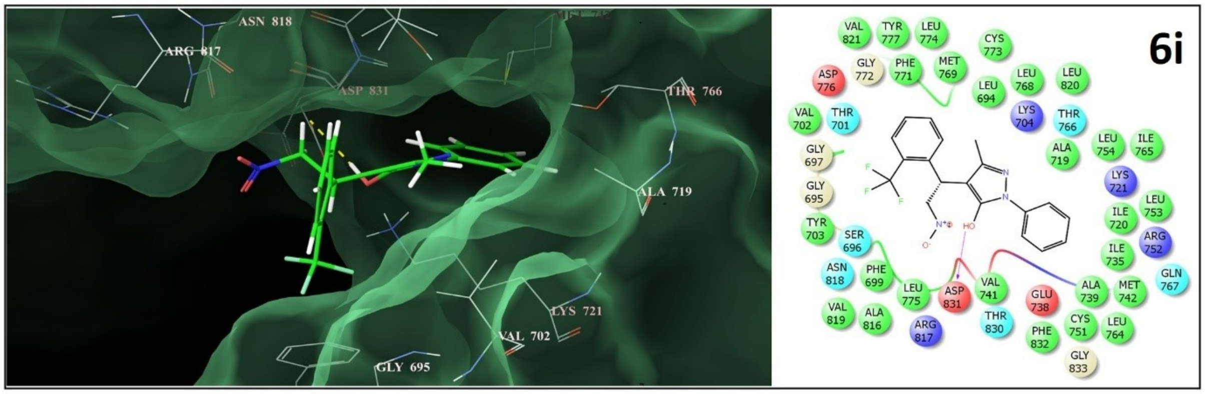
6i
ASN 818
ARG 817
ASP 831
THR 766
ALA 719
LYS 721
VAL 702
GLY 695

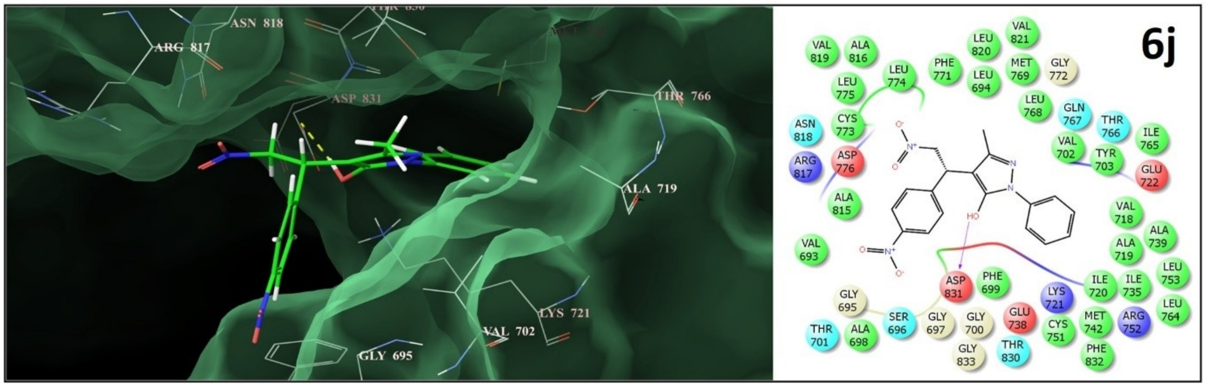
6j
ASN 818
ARG 817
ASP 831
THR 766
ALA 719
LYS 721
VAL 702
GLY 695

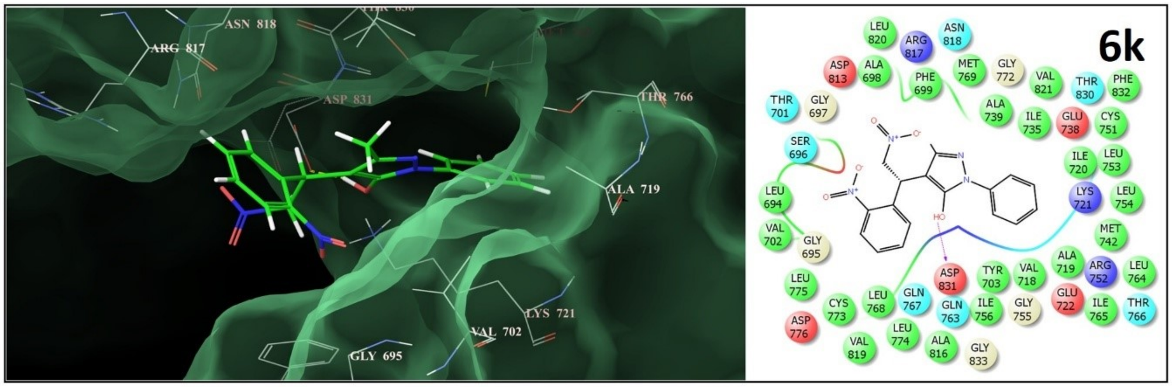
6k
ASN 818
ARG 817
ASP 831
THR 766
ALA 719
LYS 721
VAL 702
GLY 695
LEU 820
ARG 817
ASN 818
ASP 813
ALA 698
PHE 699
MET 769
GLY 772
VAL 821
THR 830
PHE 832
THR 701
GLY 697
ALA 739
ILE 735
GLU 738
CYS 751
SER 696
ILE 720
LEU 753
LEU 694
LYS 721
LEU 754
VAL 702
GLY 695
MET 742
ALA 719
ARG 752
LEU 764
LEU 775
ASP 831
TYR 703
VAL 718
CYS 773
LEU 768
GLN 767
GLN 763
ILE 756
GLY 755
GLU 722
ILE 765
THR 766
ASP 776
LEU 774
ALA 816
VAL 819
GLY 833

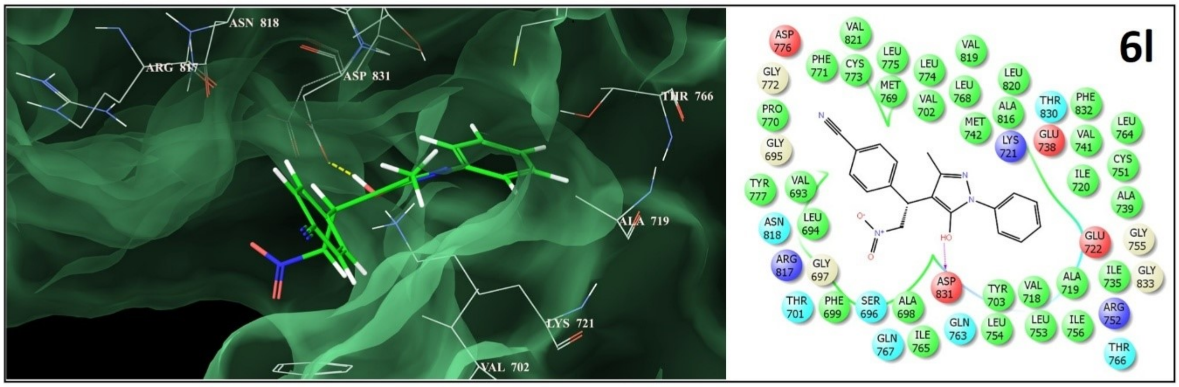
6l
ASN 818
ARG 817
ASP 831
THR 766
ALA 719
LYS 721
VAL 702
ASP 776
VAL 821
PHE 771
CYS 773
LEU 775
LEU 774
VAL 819
GLY 772
MET 769
LEU 768
LEU 820
PRO 770
VAL 702
ALA 816
THR 830
PHE 832
GLY 695
MET 742
LYS 721
GLU 738
VAL 741
LEU 764
CYS 751
TYR 777
VAL 693
ILE 720
ALA 739
ASN 818
LEU 694
GLU 722
GLY 755
ARG 817
GLY 697
ASP 831
ALA 719
ILE 735
GLY 833
THR 701
PHE 699
SER 696
ALA 698
TYR 703
VAL 718
ARG 752
GLN 767
ILE 765
GLN 763
LEU 754
LEU 753
ILE 756
THR 766

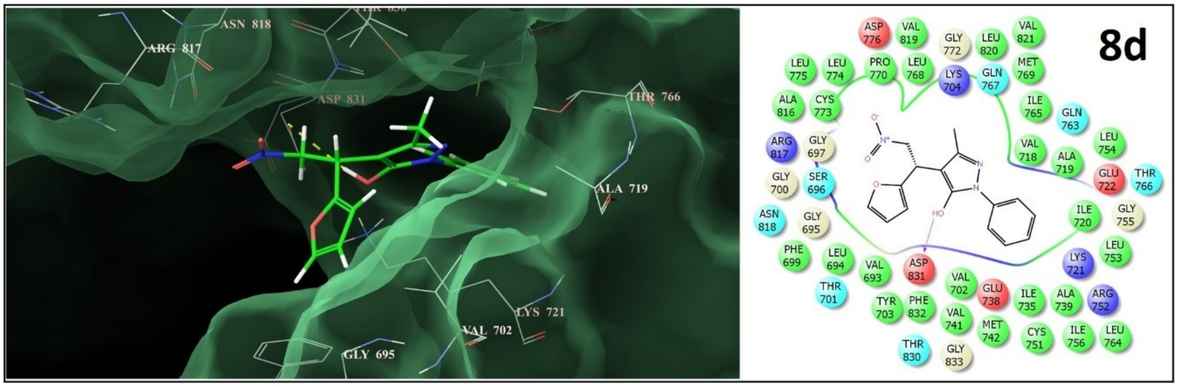
8d
ASN 818
ARG 817
ASP 831
THR 766
ALA 719
LYS 721
VAL 702
GLY 695
ASP 776
VAL 819
GLY 772
LEU 820
VAL 821
LEU 775
LEU 774
PRO 770
LEU 768
LYS 704
GLN 767
MET 769
ALA 816
CYS 773
ILE 765
GLN 763
ARG 817
GLY 697
LEU 754
GLY 700
SER 696
VAL 718
ALA 719
GLU 722
THR 766
ASN 818
GLY 695
ILE 720
GLY 755
PHE 699
LEU 694
VAL 693
LYS 721
LEU 753
THR 701
ASP 831
VAL 702
GLU 738
ILE 735
ALA 739
ARG 752
TYR 703
PHE 832
VAL 741
MET 742
CYS 751
ILE 756
LEU 764
THR 830
GLY 833

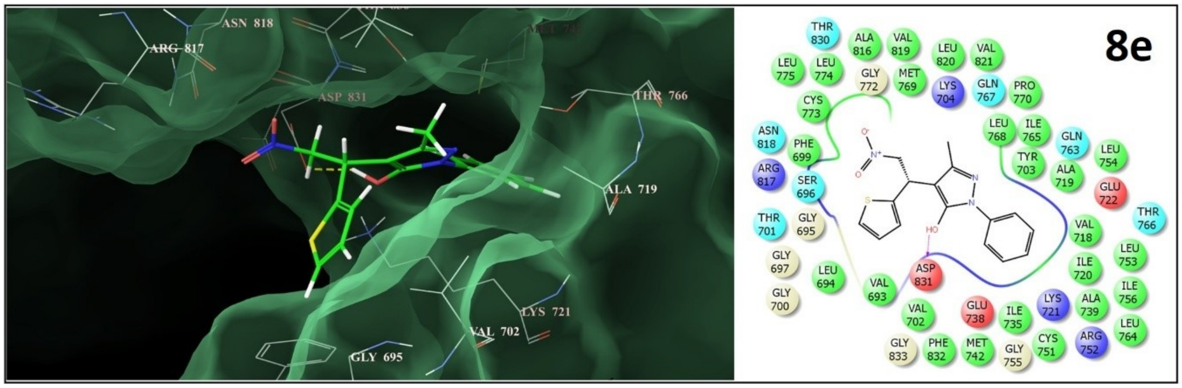
8e
ASN 818
ARG 817
ASP 831
THR 766
ALA 719
LYS 721
VAL 702
GLY 695
THR 830
ALA 816
VAL 819
LEU 820
VAL 821
LEU 775
LEU 774
GLY 772
MET 769
LYS 704
GLN 767
PRO 770
CYS 773
LEU 768
ILE 765
GLN 763
LEU 754
ASN 818
PHE 699
TYR 703
ALA 719
ARG 817
SER 696
GLU 722
THR 766
THR 701
GLY 695
VAL 718
LEU 753
GLY 697
ILE 720
LEU 694
ASP 831
ILE 756
GLY 700
VAL 693
LYS 721
ALA 739
VAL 702
GLU 738
ILE 735
CYS 751
ARG 752
LEU 764
GLY 833
PHE 832
MET 742
GLY 755

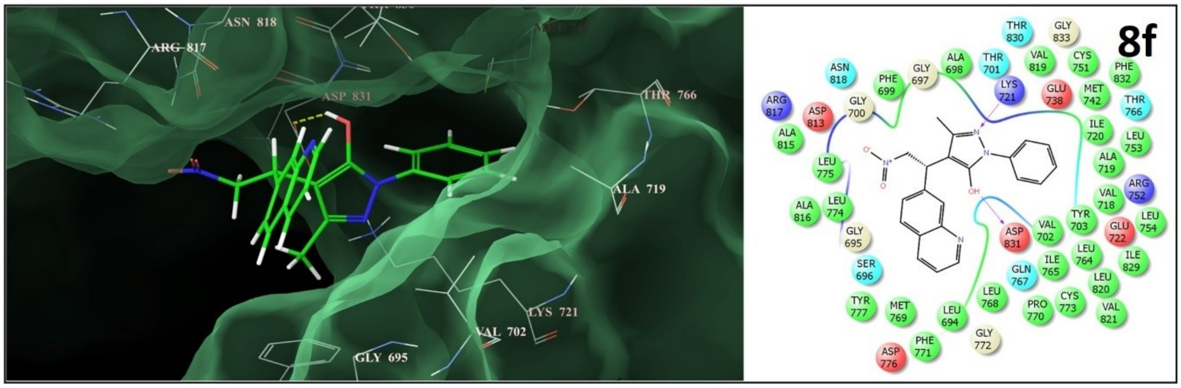

Figure S3. Representative molecular docking of efficient pyrazol-5-ol derivatives (**4**, **6** and **8** series) with their binding mode into the active site of Epidermal Growth Factor Receptor (EPGR) Tyrosine Kinase. (On right side of every docking image binding with pink line of compound with receptor signifies the H-bonding interactions).

Supplementary Tables

Table S1. X-ray diffraction (XRD) parameters (2θ, full width at half maxim., FWHM, interplanar distance, and crystallite size) of graphene oxide (GO) before Cycle I and after Cycle V.

GO	2θ	FWHM	Interplanar distance (nm)	Crystallite Size (nm)
Cycle I	11.47	1.72	0.77	5.16
	42.53	1.45	0.21	4.07
Cycle V	11.01	1.11	0.80	8.66
	42.28	1.02	0.21	6.32

Table S2. X-ray photoelectron spectroscopy (XPS) peak parameters (binding energy, peak area, FWHM, and atomic concentration) and corresponding functional groups of graphene oxide (GO) before Cycle I and after Cycle V.

GO	Peaks	Binding Energy (eV)	Peak Area	FWHW (eV)	At Conc. (%)	(C/O)	Func. Groups
Cycle I	C 1s	284.62	79606.47	1.27	26.64	1.80	C-C
	C 1s	286.63	53759.91	1.47	17.99		C-O
	C 1s	287.98	58730.95	4.39	19.65		C=O
	O 1s	532.53	158792.62	1.92	18.14		O-C=O
	O 1s	533.97	153934.44	4.10	17.58		C=O
Cycle V	C 1s	284.47	40854.58	1.20	24.52	3.06	C-C
	C 1s	286.42	84752.94	3.19	50.86		C-O
	O 1s	530.68	2416.26	0.84	0.49		C=C
	O 1s	532.55	117773.94	2.25	24.12		C-O

Spectral data (^{1}H NMR and ^{13}C NMR) of pyrazol-5-ol derivative (4, 4*a-k*, 6*a-l*, 8*a-d*)

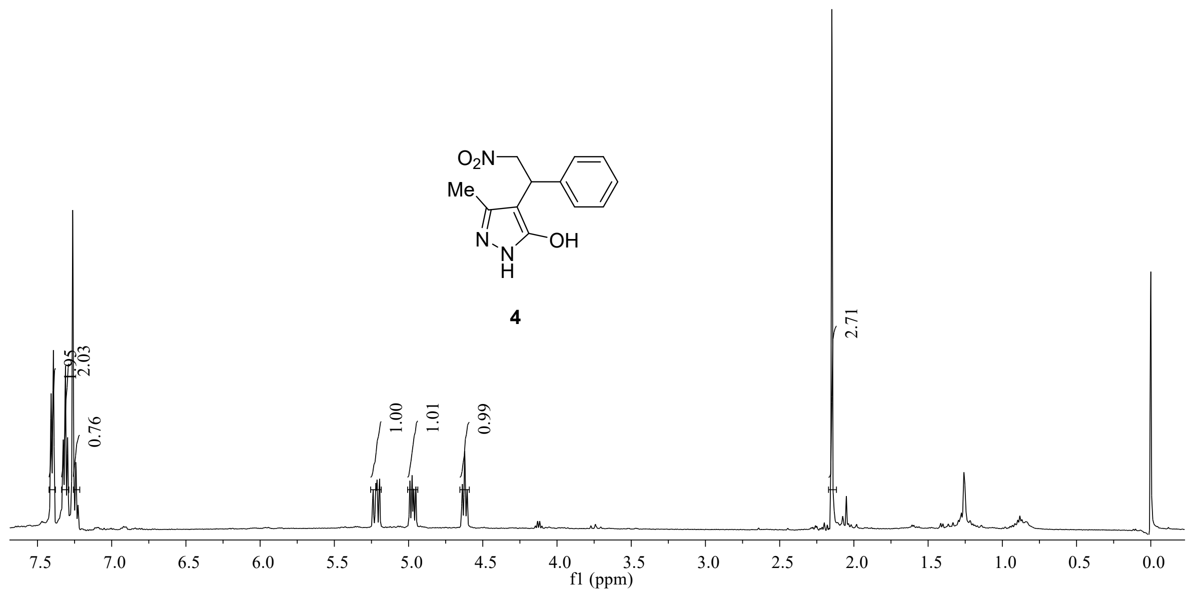

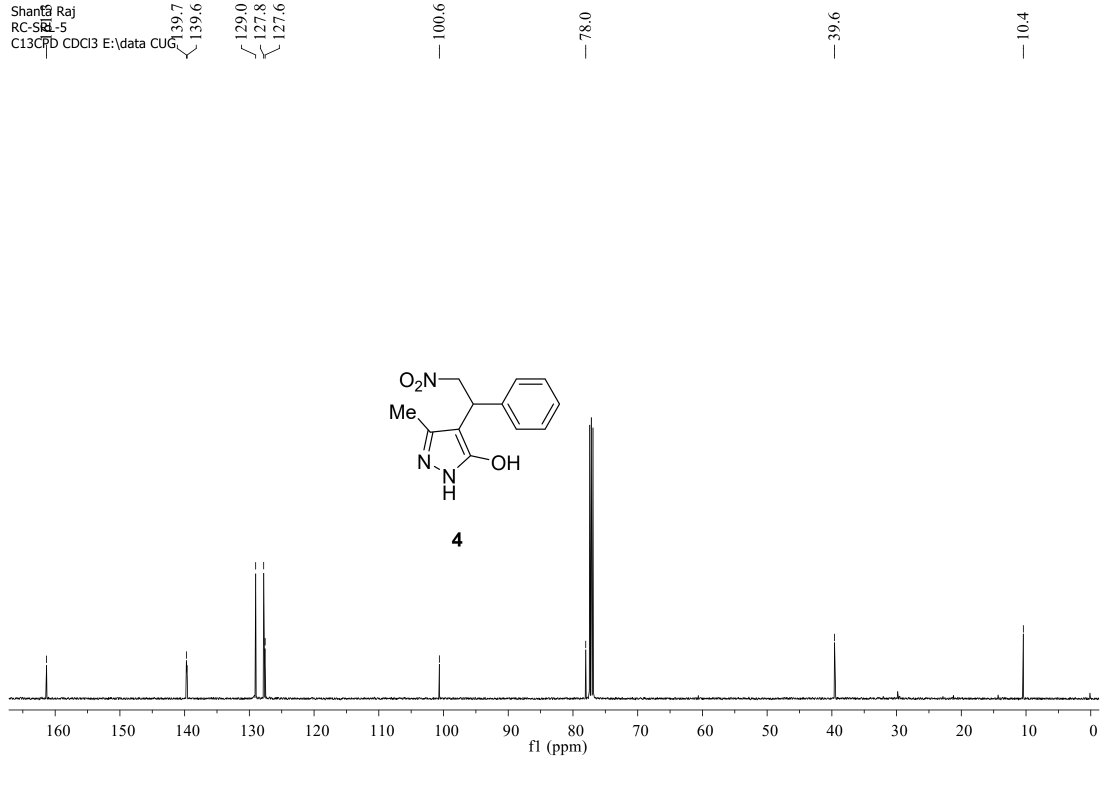

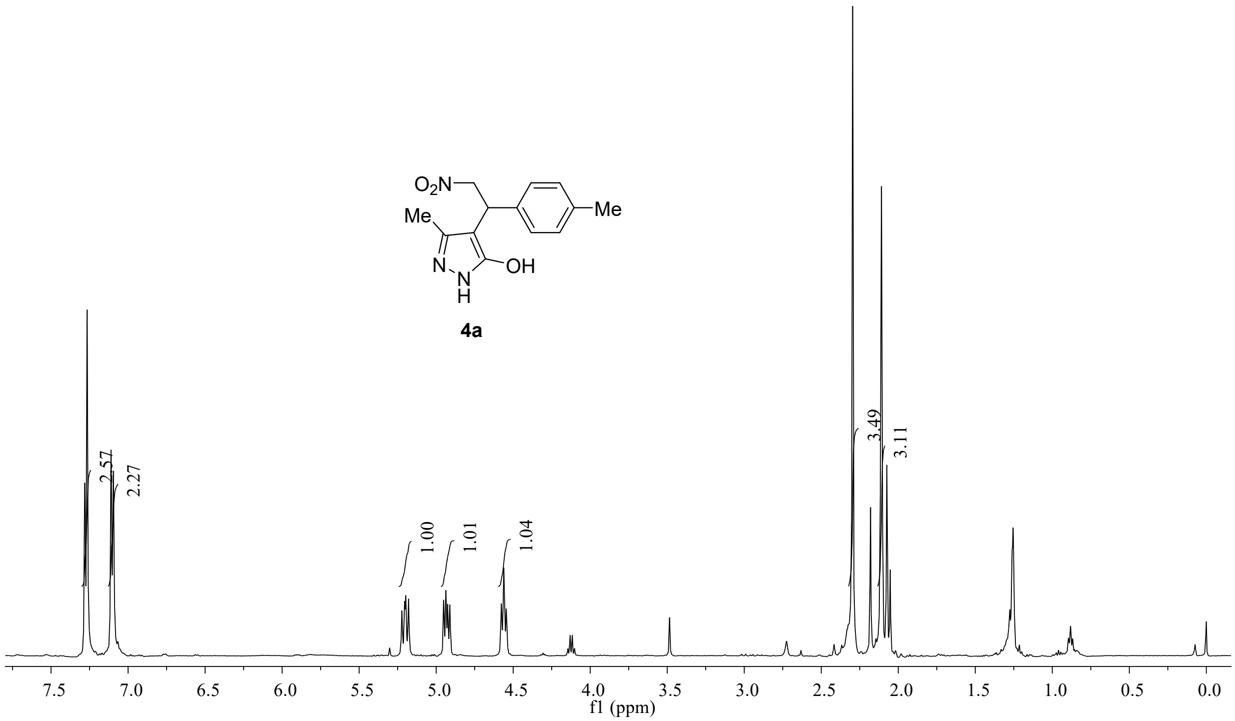

O2N
Me
Me
N
N
H
OH
4a
2.57
2.27
1.00
1.01
1.04
3.49
3.11
f1 (ppm)

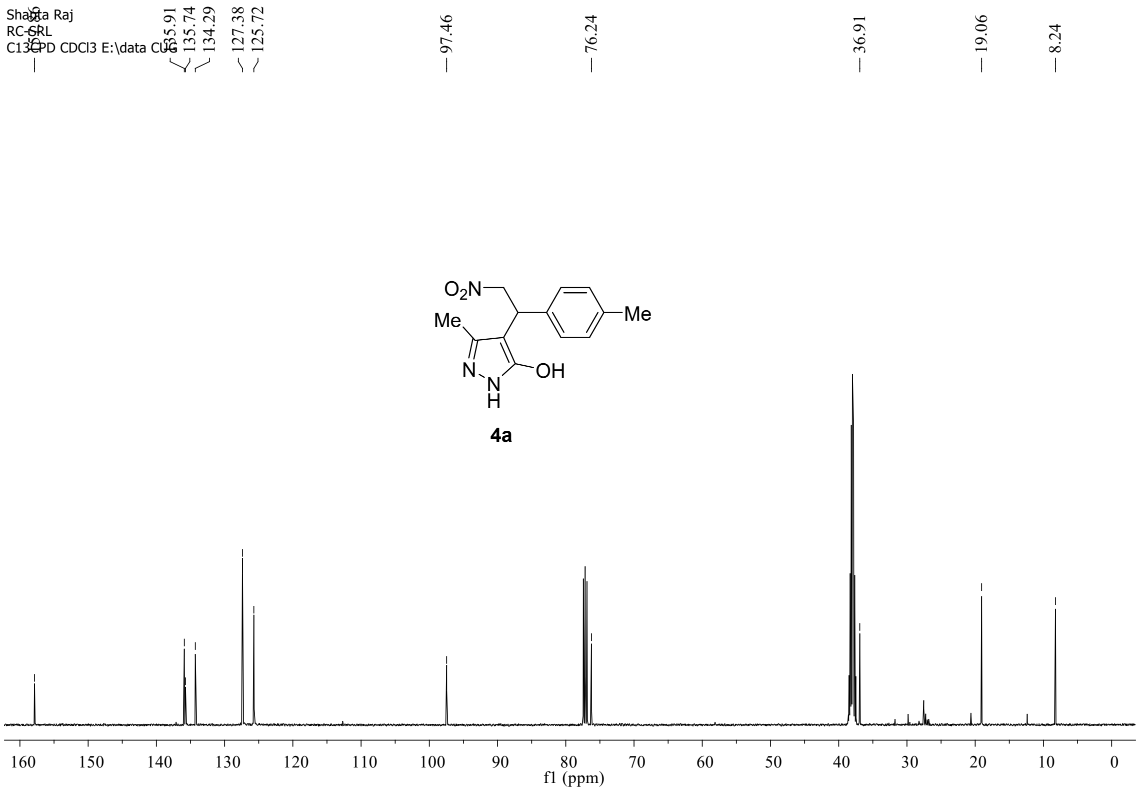

Shanta Raj
RC-SRL
C13CPD CDCl3 E:\data CUG
135.91
135.74
134.29
127.38
125.72
97.46
76.24
36.91
19.06
8.24
O2N
Me
Me
N
N
H
OH
4a
f1 (ppm)

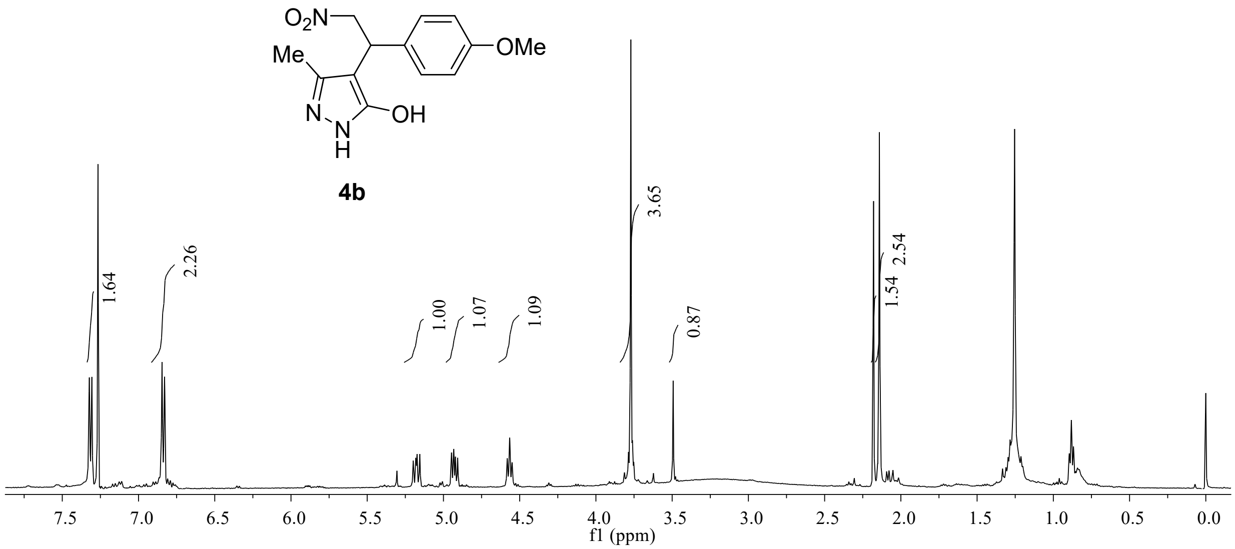

O2N
OMe
Me
N
N
H
OH
4b
1.64
2.26
1.00
1.07
1.09
3.65
0.87
1.54
2.54
7.5
7.0
6.5
6.0
5.5
5.0
4.5
4.0
3.5
3.0
2.5
2.0
1.5
1.0
0.5
0.0
f1 (ppm)

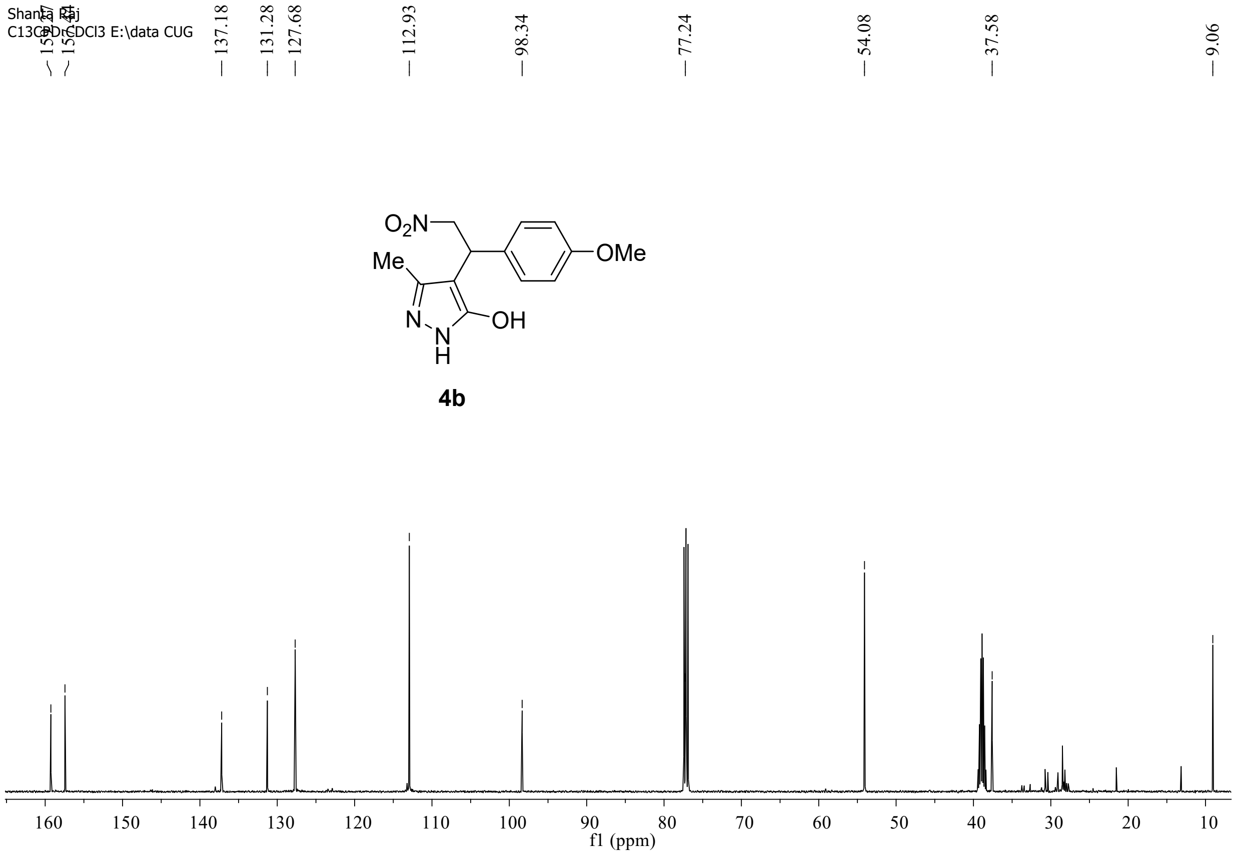

Shanta Raj
C13CPD CDCl3 E:\data CUG
137.18
131.28
127.68
112.93
98.34
77.24
54.08
37.58
9.06
O2N
OMe
Me
N
N
H
OH
4b
160
150
140
130
120
110
100
90
80
70
60
50
40
30
20
10
f1 (ppm)

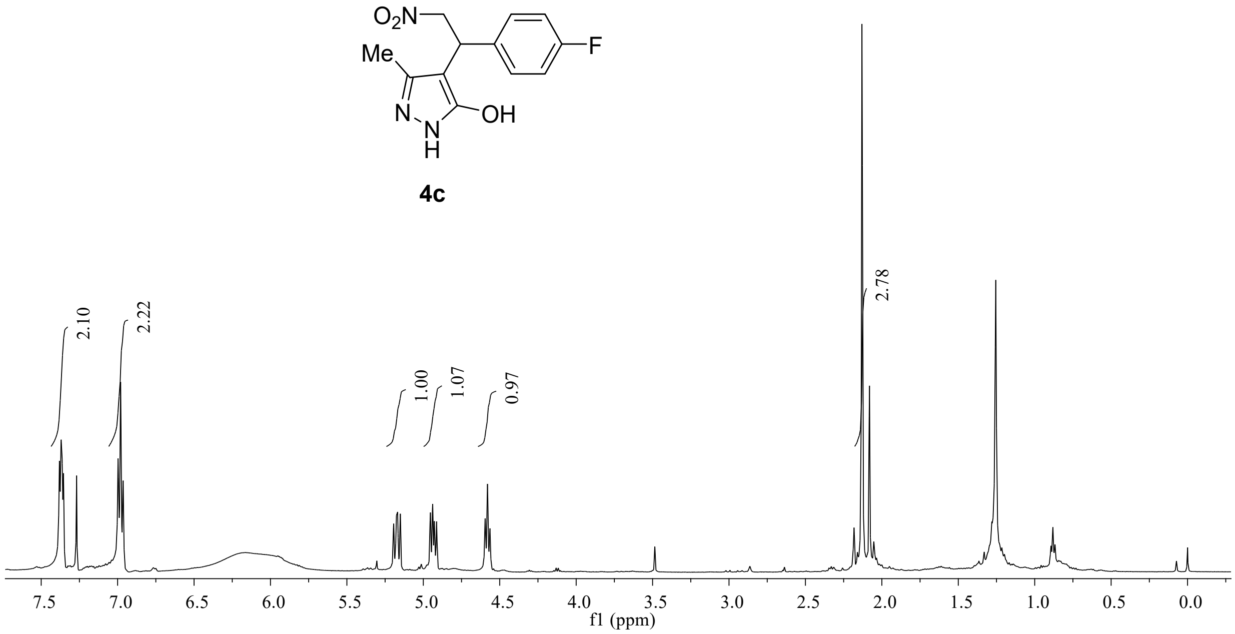
O2N
Me
N
N
H
OH
F
4c
2.10
2.22
1.00
1.07
0.97
2.78
f1 (ppm)

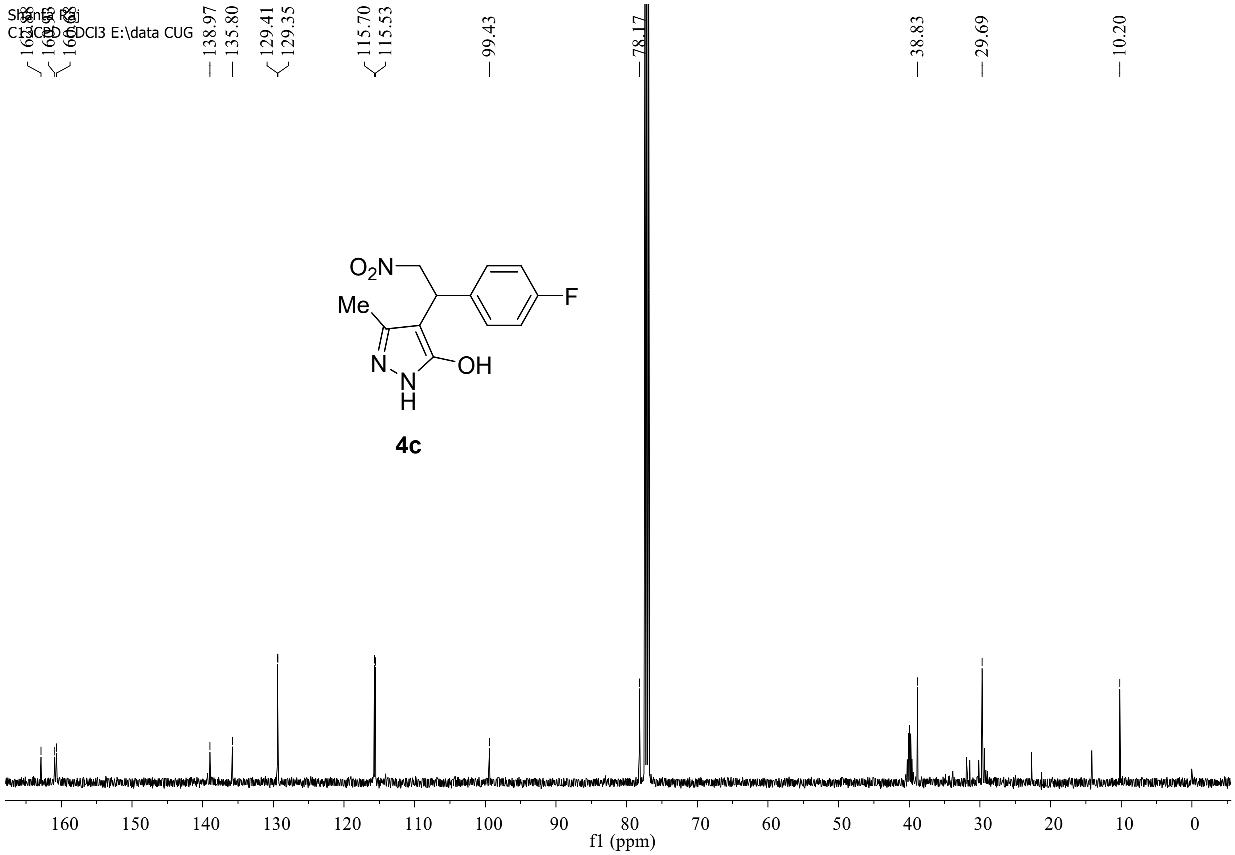
C13CPD CDCl3 E:\data CUG
138.97
135.80
129.41
129.35
115.70
115.53
99.43
78.17
38.83
29.69
10.20
O2N
Me
N
N
H
OH
F
4c
f1 (ppm)

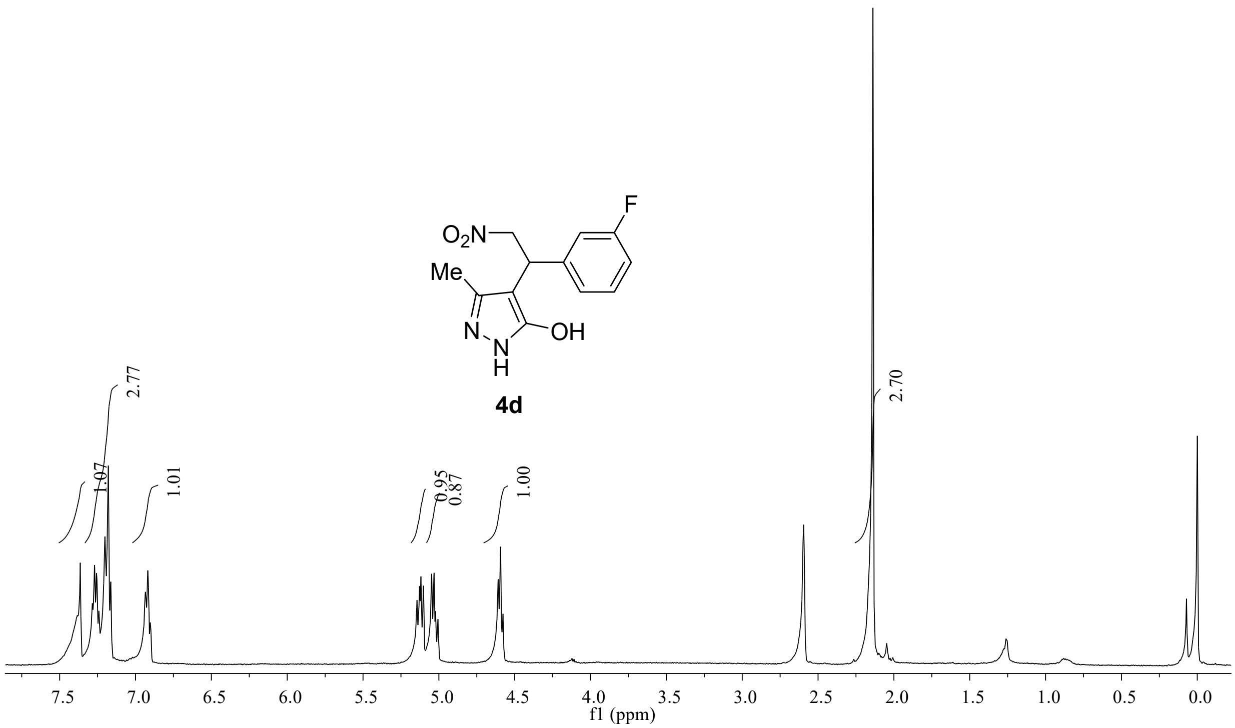

O2N
F
Me
N
N
H
OH
4d
2.77
1.07
1.01
0.95
0.87
1.00
2.70
7.5
7.0
6.5
6.0
5.5
5.0
4.5
4.0
3.5
3.0
2.5
2.0
1.5
1.0
0.5
0.0
f1 (ppm)

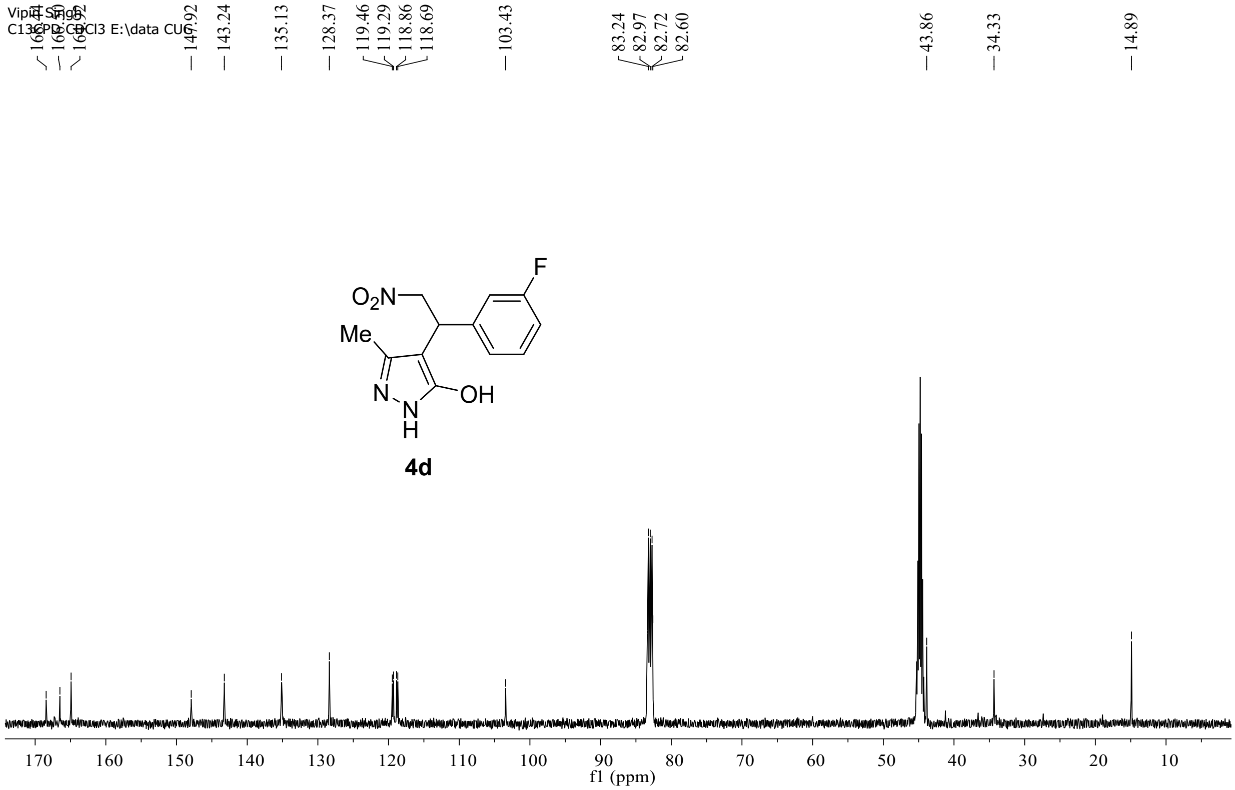

Vipin Singh
C13CPD CDCl3 E:\data CUG
147.92
143.24
135.13
128.37
119.46
119.29
118.86
118.69
103.43
83.24
82.97
82.72
82.60
43.86
34.33
14.89
O2N
F
Me
N
N
H
OH
4d
170
160
150
140
130
120
110
100
90
80
70
60
50
40
30
20
10
f1 (ppm)

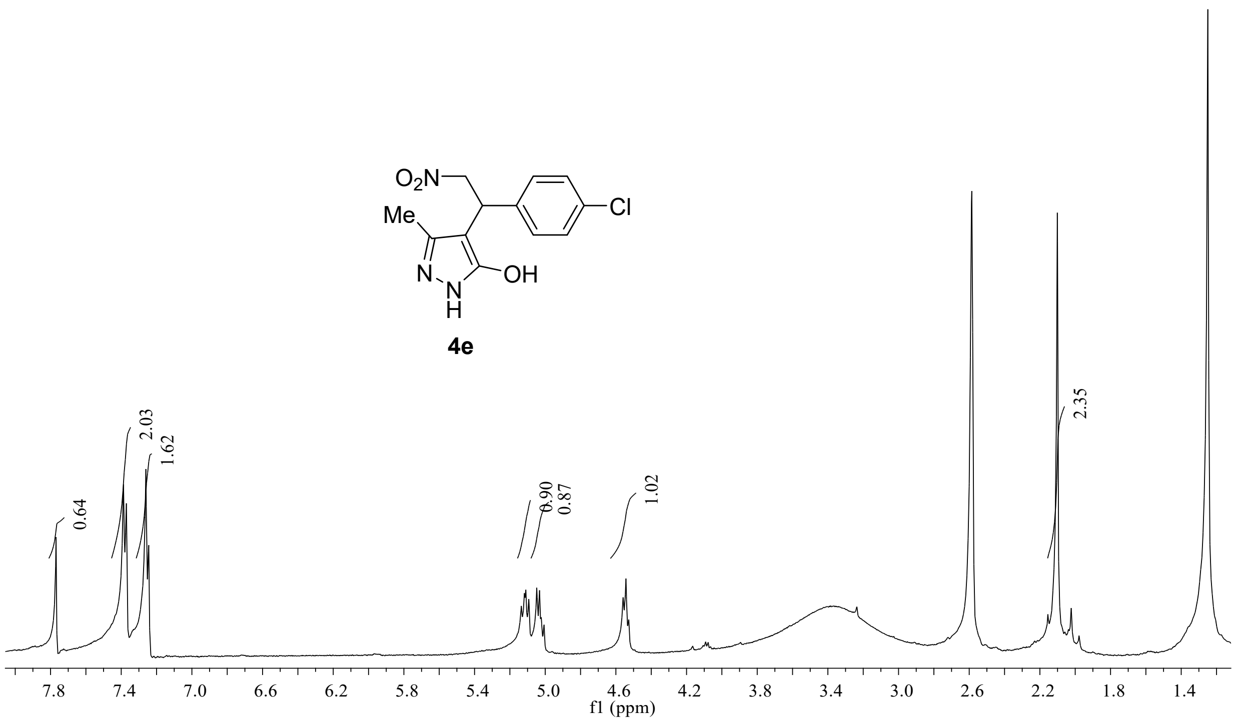

O2N
Me
Cl
N
N
H
OH
4e
0.64
2.03
1.62
0.90
0.87
1.02
2.35
f1 (ppm)

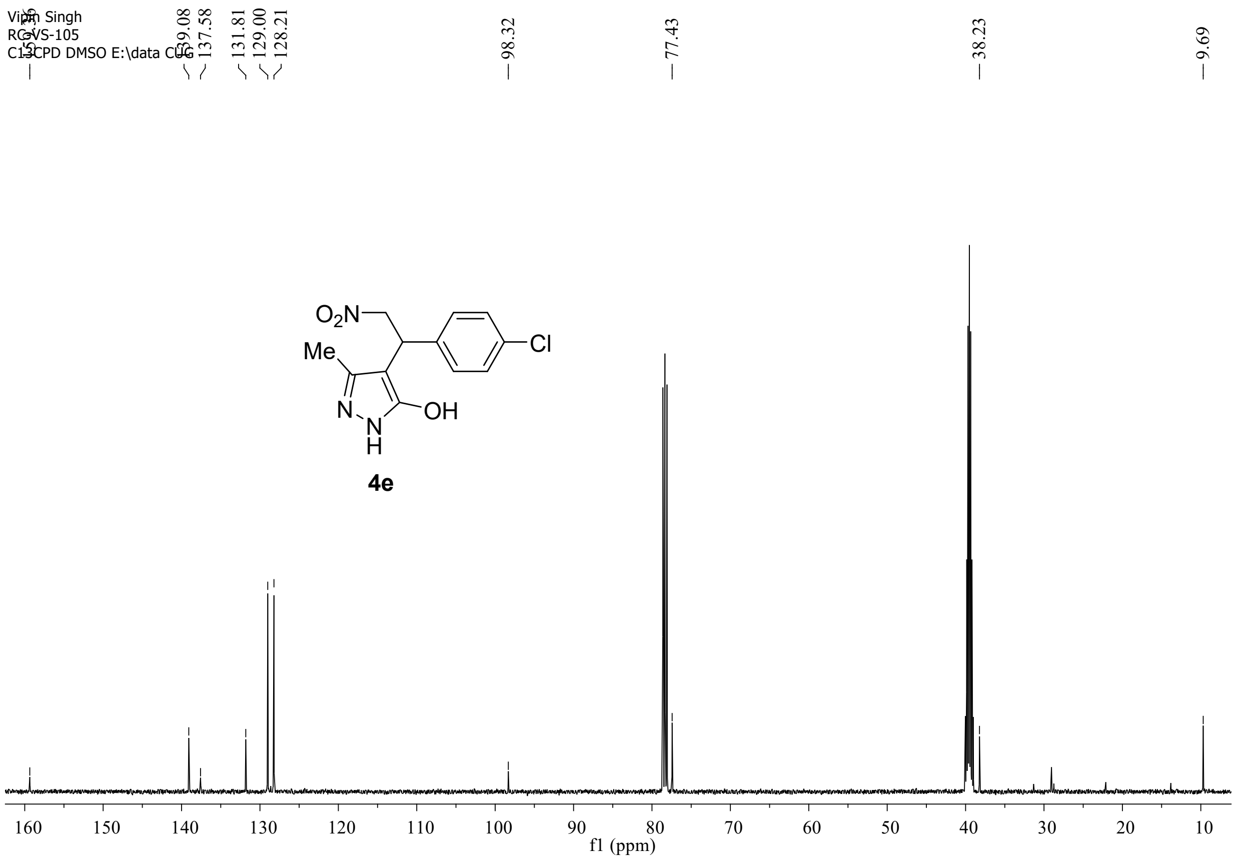

Vipin Singh
RC-VS-105
C13CPD DMSO E:\data CUG
139.08
137.58
131.81
129.00
128.21
98.32
77.43
38.23
9.69
O2N
Me
Cl
N
N
H
OH
4e
f1 (ppm)

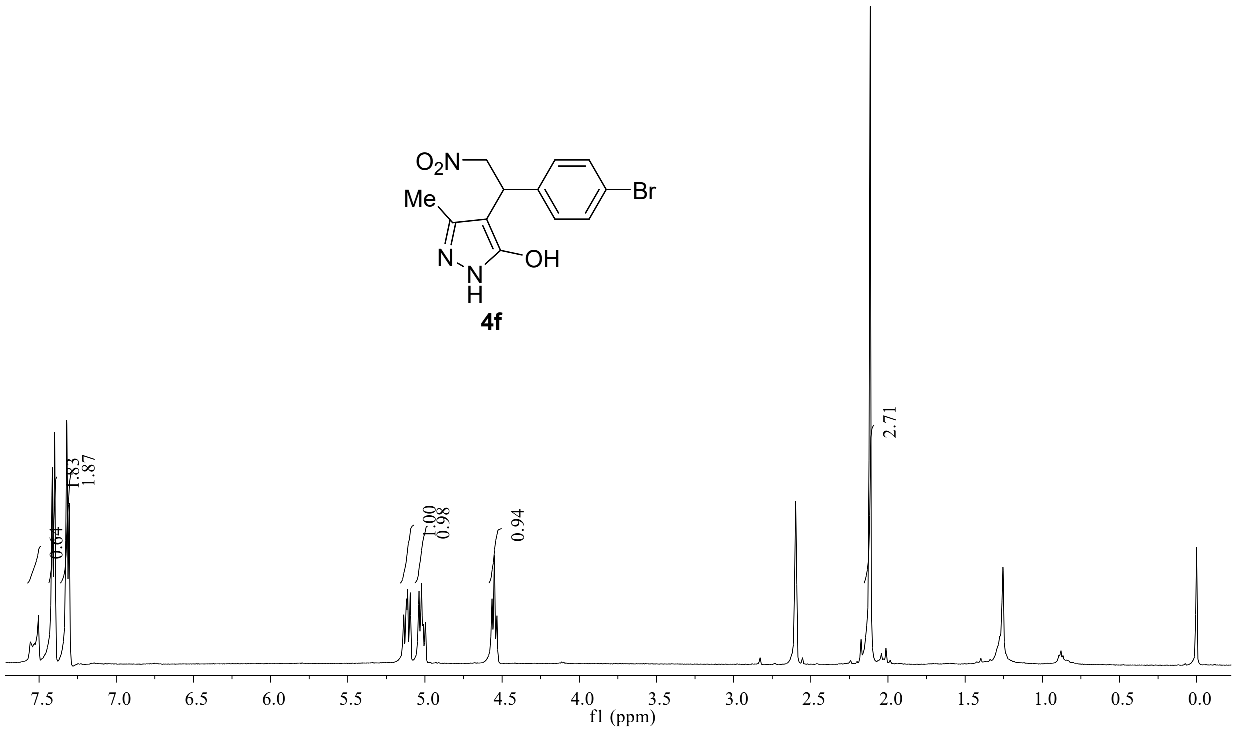

4f
f1 (ppm)

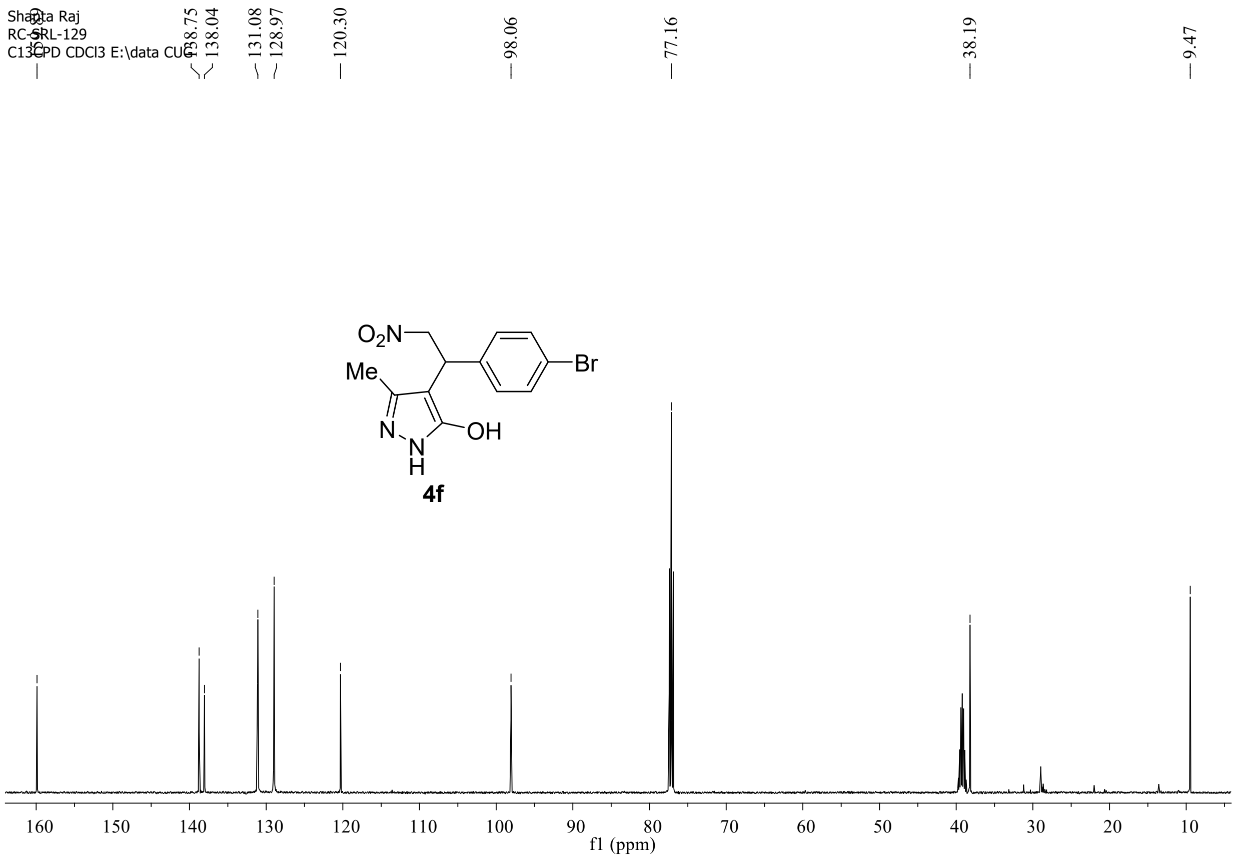

Shanta Raj
RC-SRL-129
C13CPD CDCl3 E:\data [illegible]
4f
f1 (ppm)

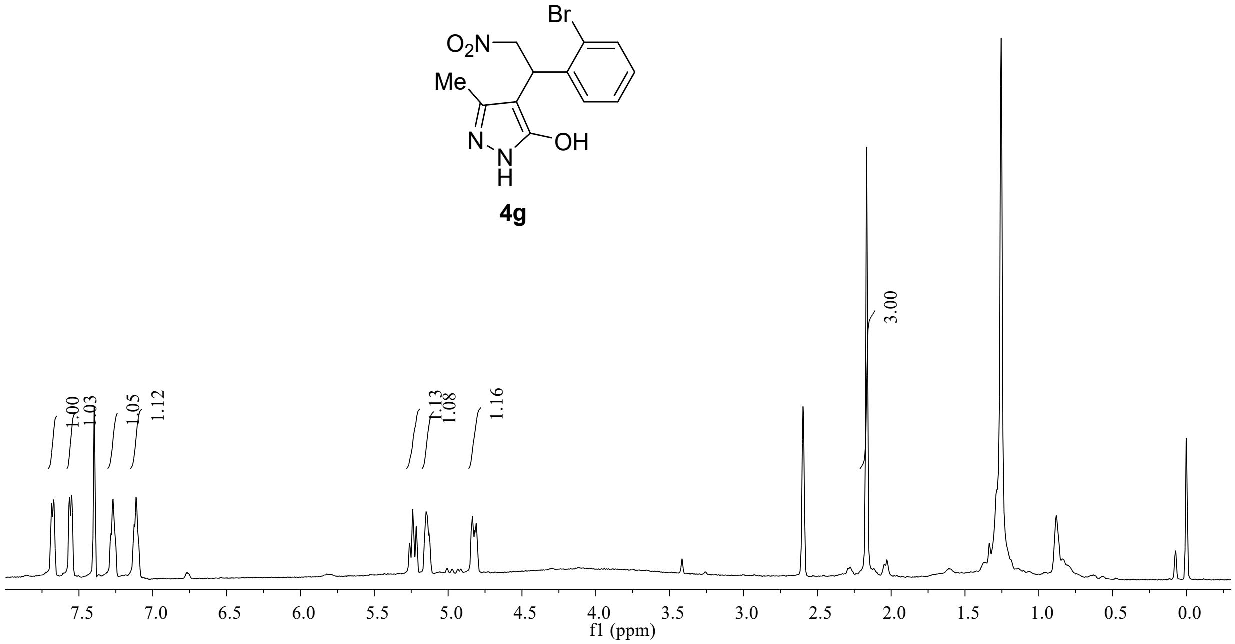
Br
O2N
Me
N
N
H
OH
4g
1.00
1.03
1.05
1.12
1.13
1.08
1.16
3.00
7.5 7.0 6.5 6.0 5.5 5.0 4.5 4.0 3.5 3.0 2.5 2.0 1.5 1.0 0.5 0.0
f1 (ppm)

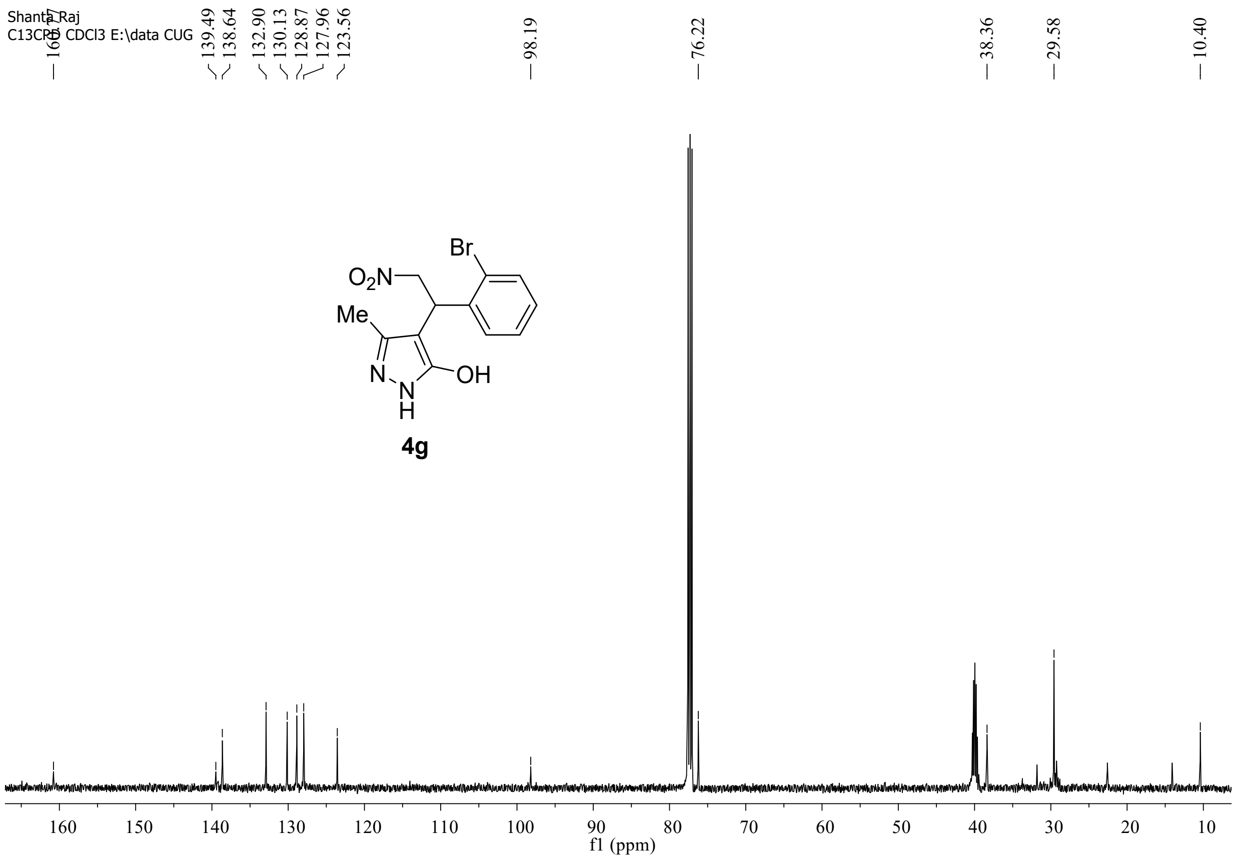
Shanta Raj
C13CPD CDCl3 E:\data CUG
139.49
138.64
132.90
130.13
128.87
127.96
123.56
98.19
76.22
38.36
29.58
10.40
Br
O2N
Me
N
N
H
OH
4g
160 150 140 130 120 110 100 90 80 70 60 50 40 30 20 10
f1 (ppm)

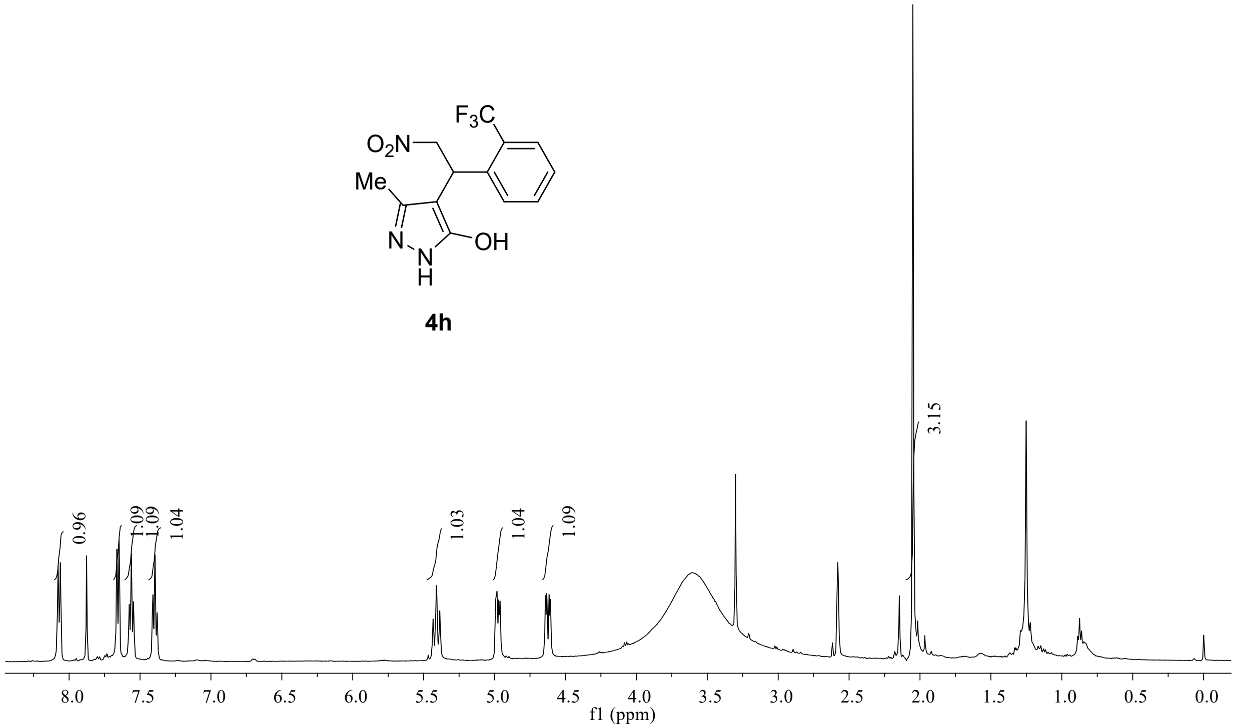

F3C
O2N
Me
N
N
H
OH
4h
0.96
1.09
1.09
1.04
1.03
1.04
1.09
3.15
8.0 7.5 7.0 6.5 6.0 5.5 5.0 4.5 4.0 3.5 3.0 2.5 2.0 1.5 1.0 0.5 0.0
f1 (ppm)

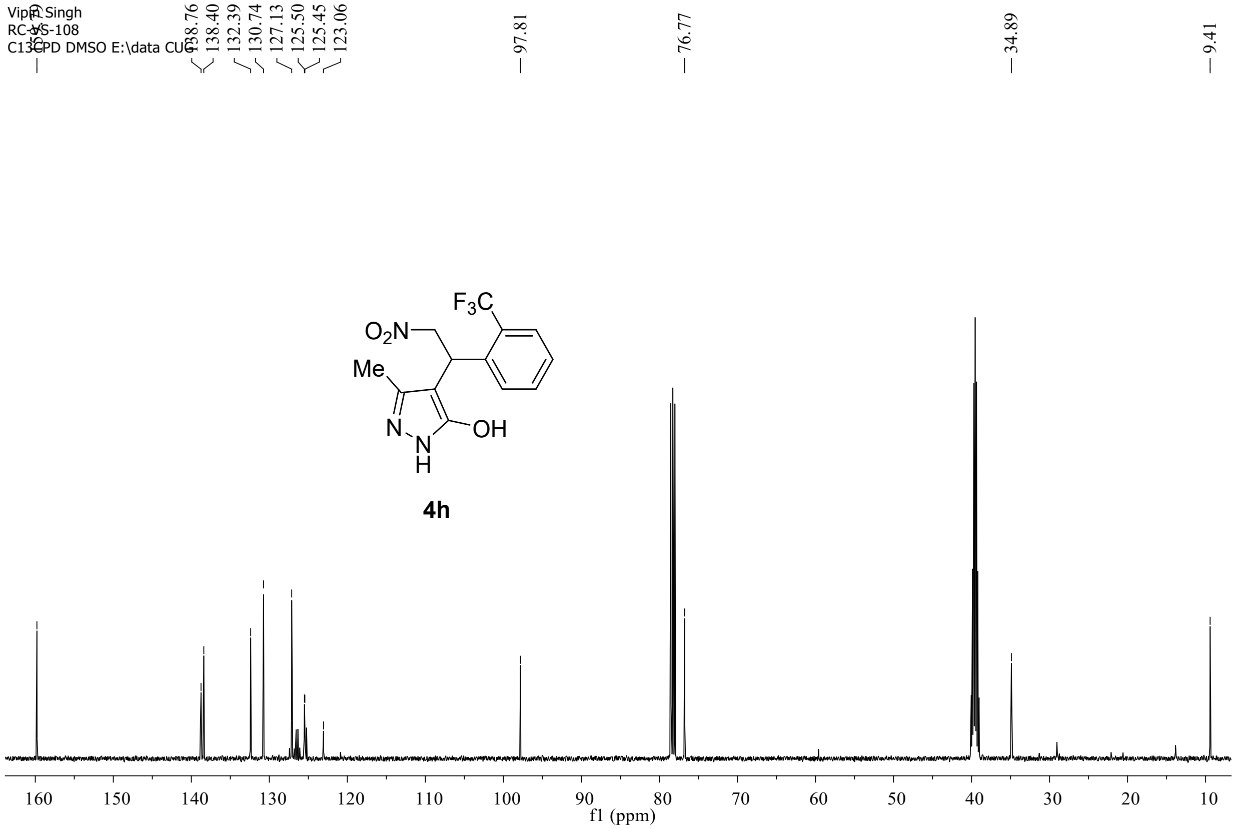

Vipin Singh
RC-VS-108
C13CPD DMSO E:\data CU
138.76
138.40
132.39
130.74
127.13
125.50
125.45
123.06
97.81
76.77
34.89
9.41
F3C
O2N
Me
N
N
H
OH
4h
160 150 140 130 120 110 100 90 80 70 60 50 40 30 20 10
f1 (ppm)

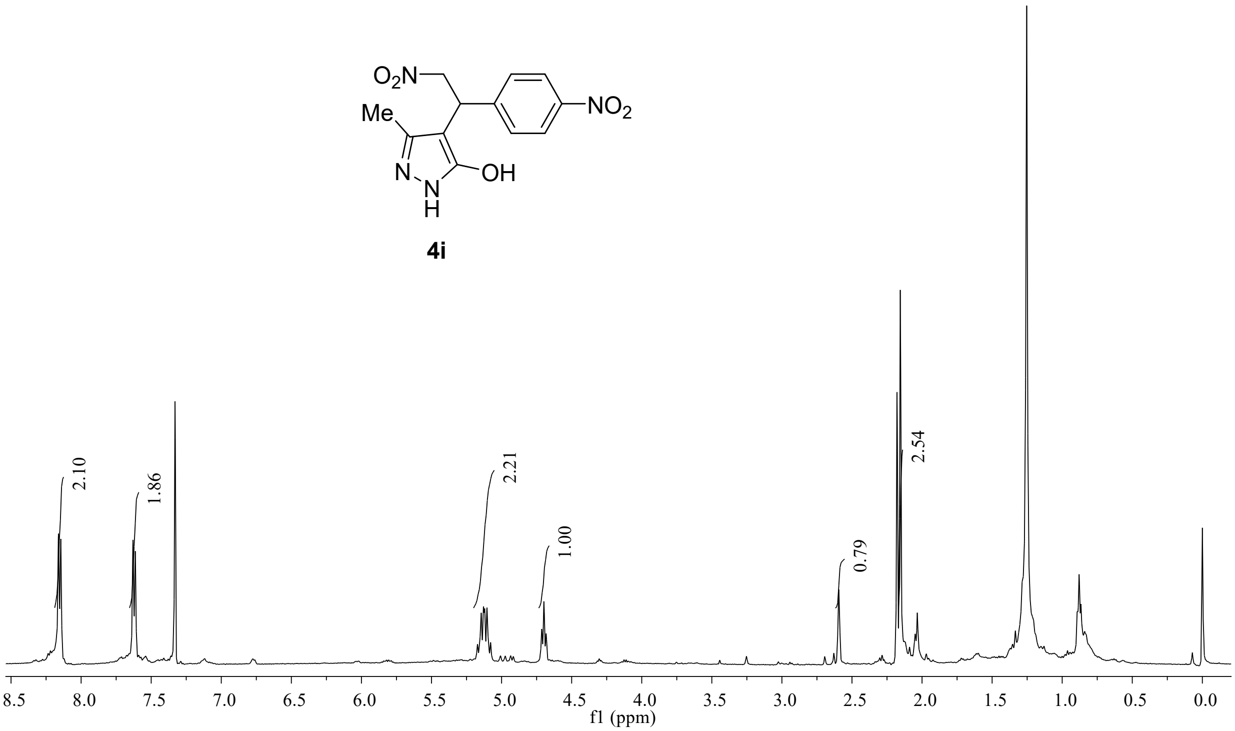
O2N
Me
NO2
N
N
H
OH
4i
2.10
1.86
2.21
1.00
0.79
2.54
8.5 8.0 7.5 7.0 6.5 6.0 5.5 5.0 4.5 4.0 3.5 3.0 2.5 2.0 1.5 1.0 0.5 0.0
f1 (ppm)

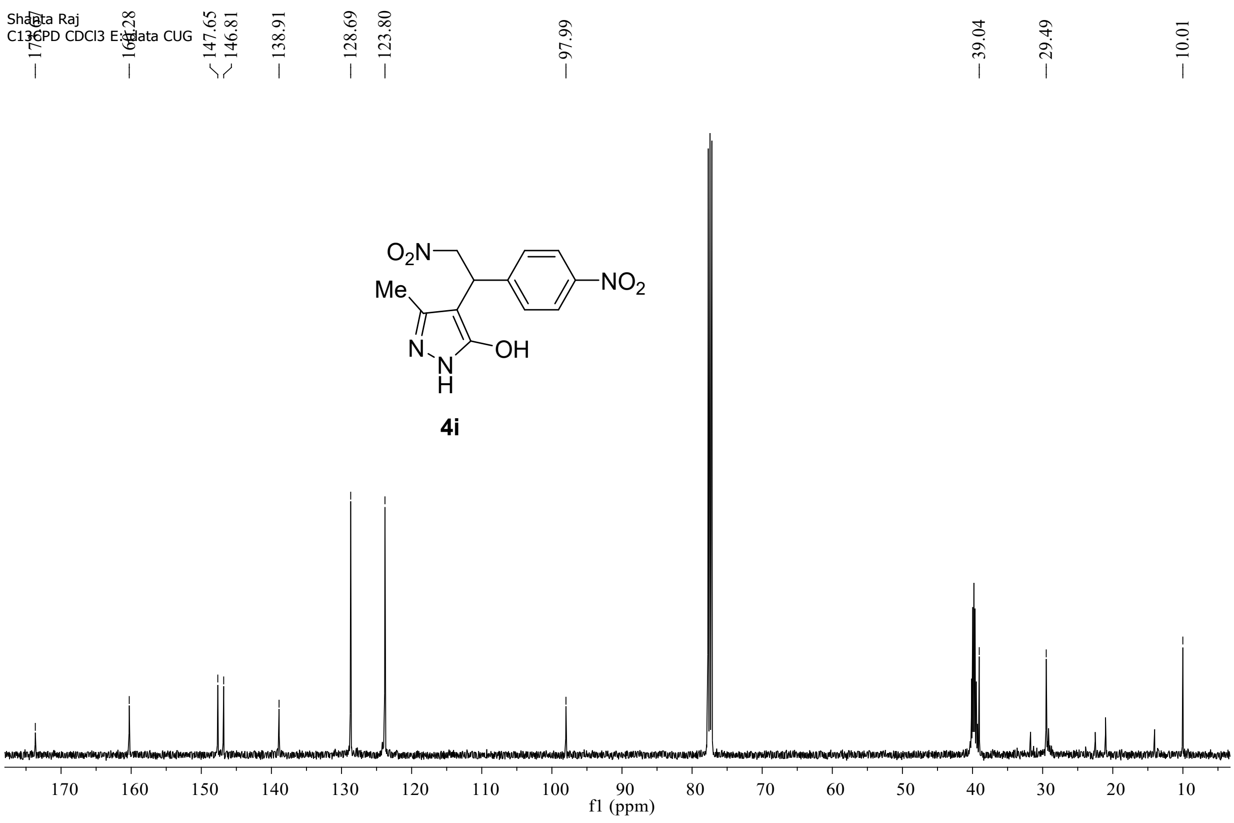
Shanta Raj
C13CPD CDCl3 E:\data CUG
160.28
147.65
146.81
138.91
128.69
123.80
97.99
39.04
29.49
10.01
O2N
Me
NO2
N
N
H
OH
4i
170 160 150 140 130 120 110 100 90 80 70 60 50 40 30 20 10
f1 (ppm)

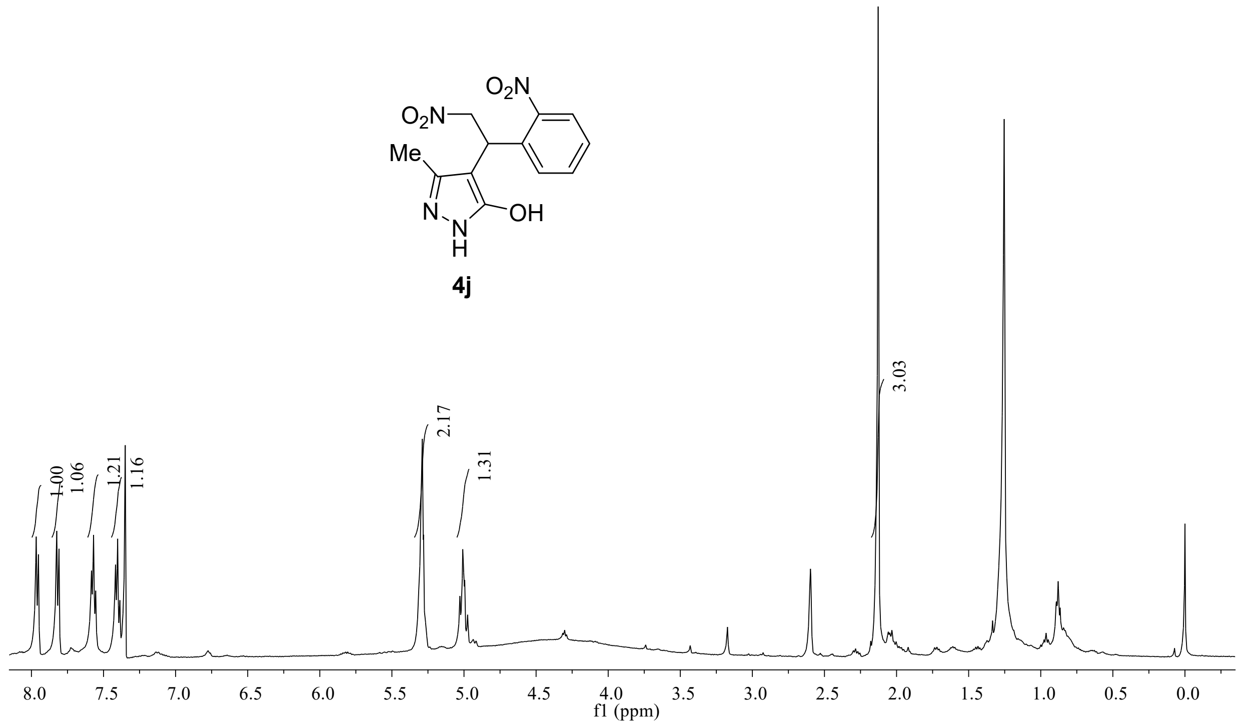
O2N
O2N
Me
N
N
H
OH
4j
1.00
1.06
1.21
1.16
2.17
1.31
3.03
8.0 7.5 7.0 6.5 6.0 5.5 5.0 4.5 4.0 3.5 3.0 2.5 2.0 1.5 1.0 0.5 0.0
f1 (ppm)

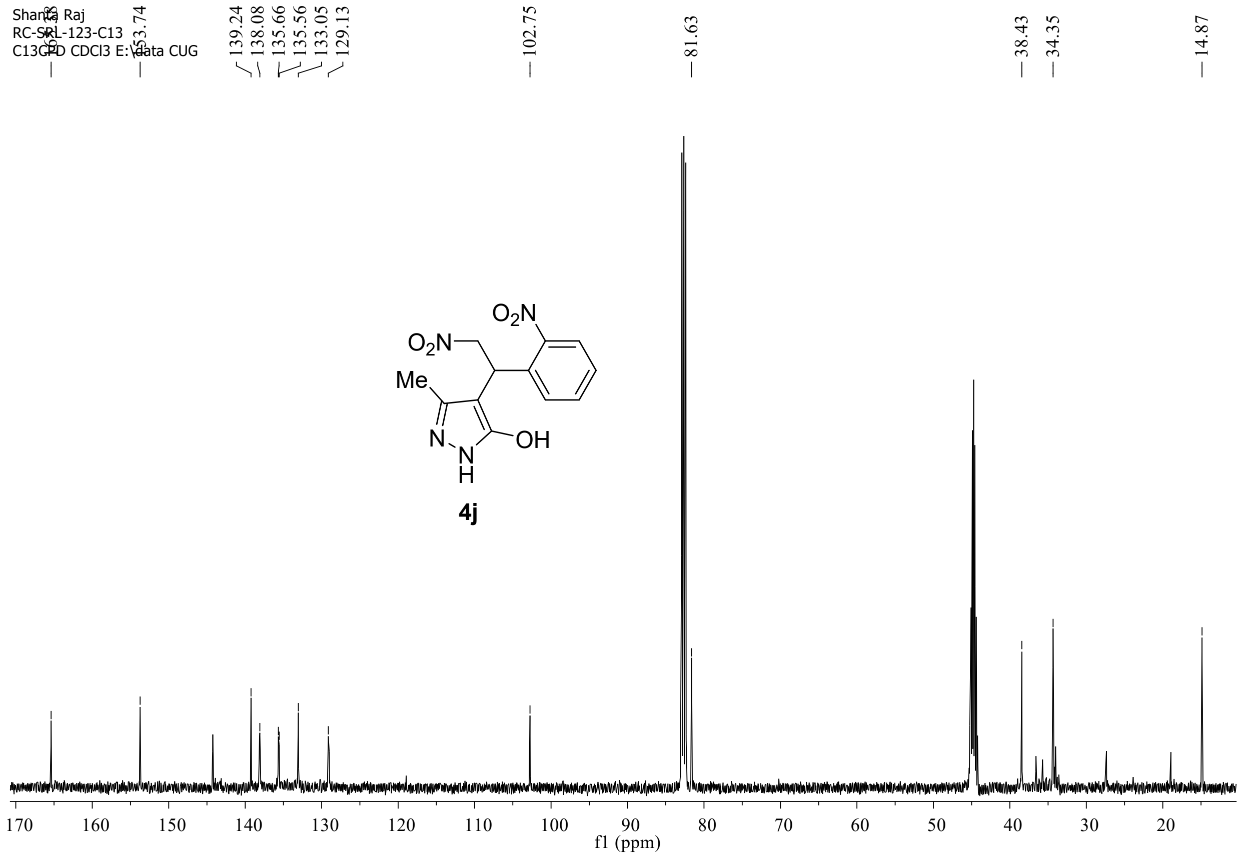
Shanta Raj
RC-SRL-123-C13
C13CPD CDCl3 E:\data CUG
153.74
139.24
138.08
135.66
135.56
133.05
129.13
102.75
81.63
38.43
34.35
14.87
O2N
O2N
Me
N
N
H
OH
4j
170 160 150 140 130 120 110 100 90 80 70 60 50 40 30 20
f1 (ppm)

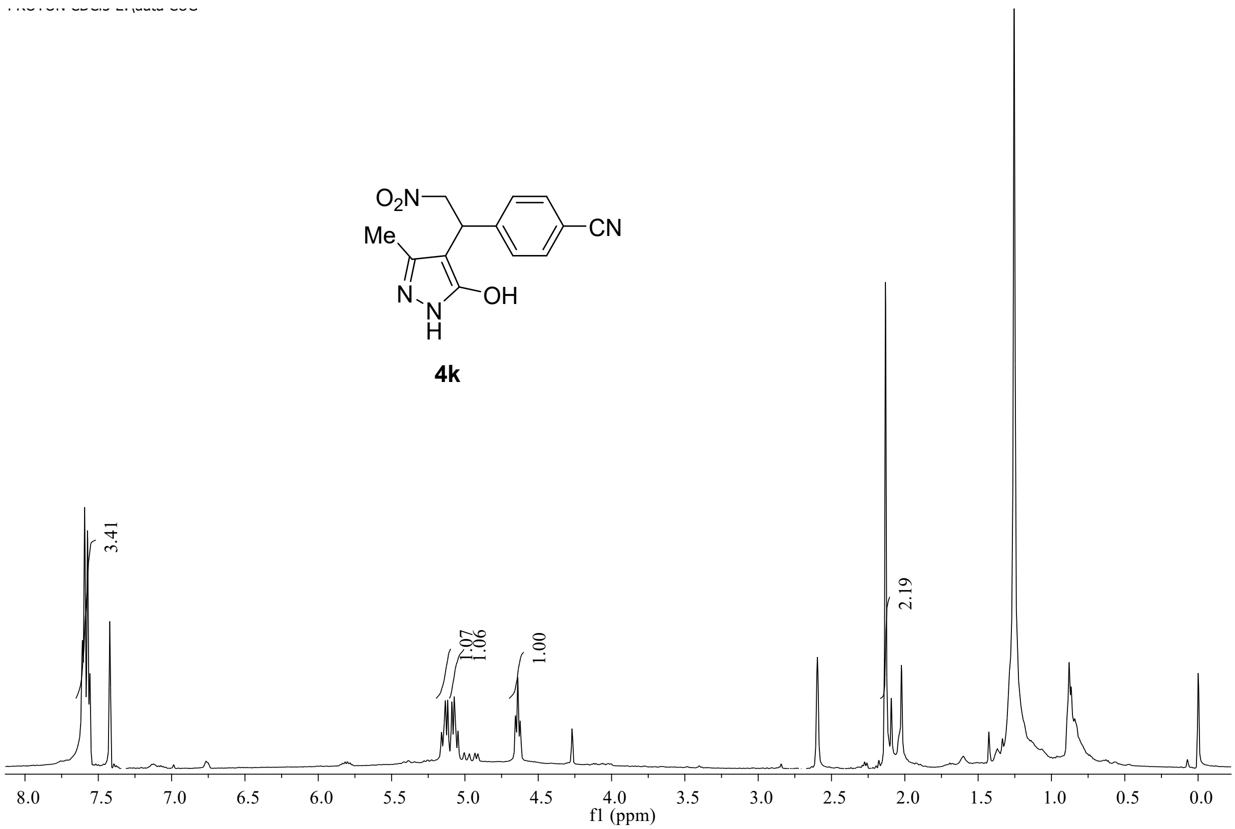
O2N
Me
CN
N
N
H
OH
4k
3.41
1.07
1.06
1.00
2.19
8.0 7.5 7.0 6.5 6.0 5.5 5.0 4.5 4.0 3.5 3.0 2.5 2.0 1.5 1.0 0.5 0.0
f1 (ppm)

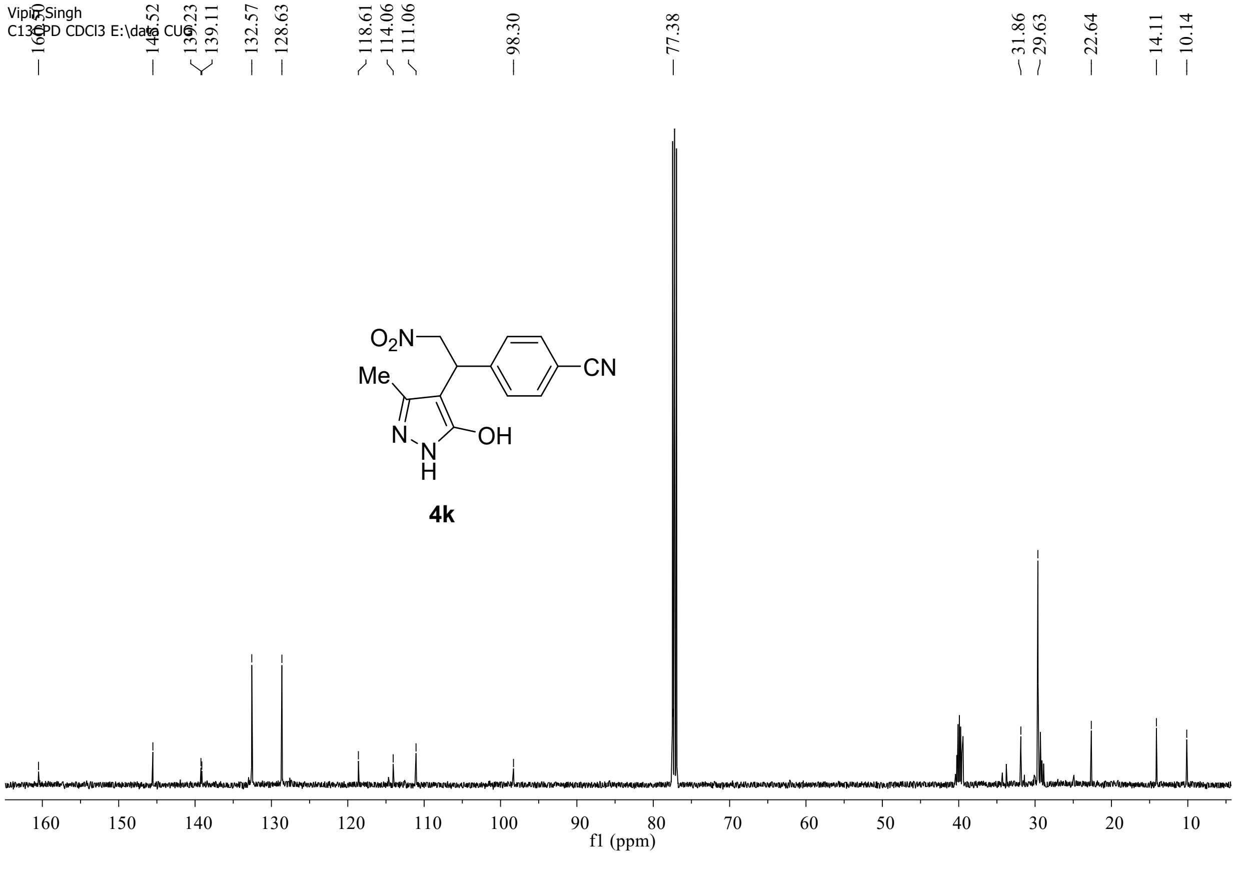
Vipin Singh
139.11
132.57
128.63
118.61
114.06
111.06
98.30
77.38
31.86
29.63
22.64
14.11
10.14
O2N
Me
CN
N
N
H
OH
4k
160 150 140 130 120 110 100 90 80 70 60 50 40 30 20 10
f1 (ppm)

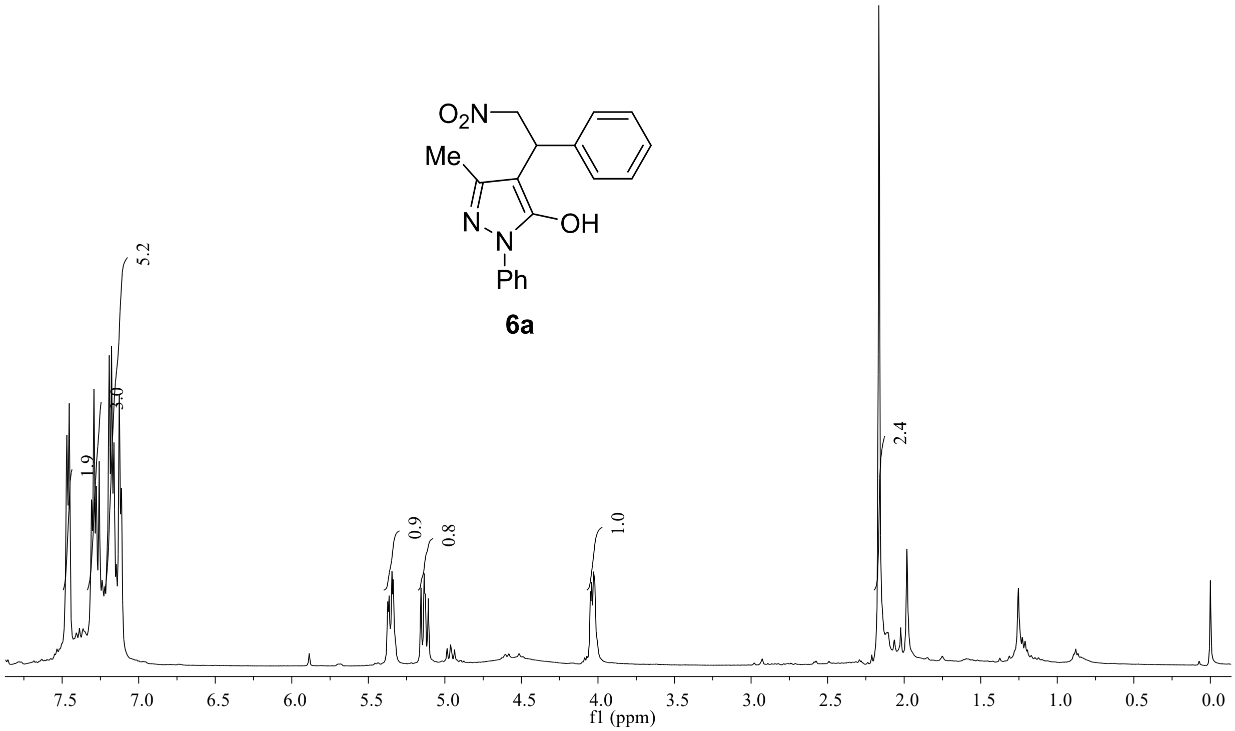
O2N
Me
N
N
OH
Ph
6a
5.2
3.0
1.9
0.9
0.8
1.0
2.4
7.5 7.0 6.5 6.0 5.5 5.0 4.5 4.0 3.5 3.0 2.5 2.0 1.5 1.0 0.5 0.0
f1 (ppm)

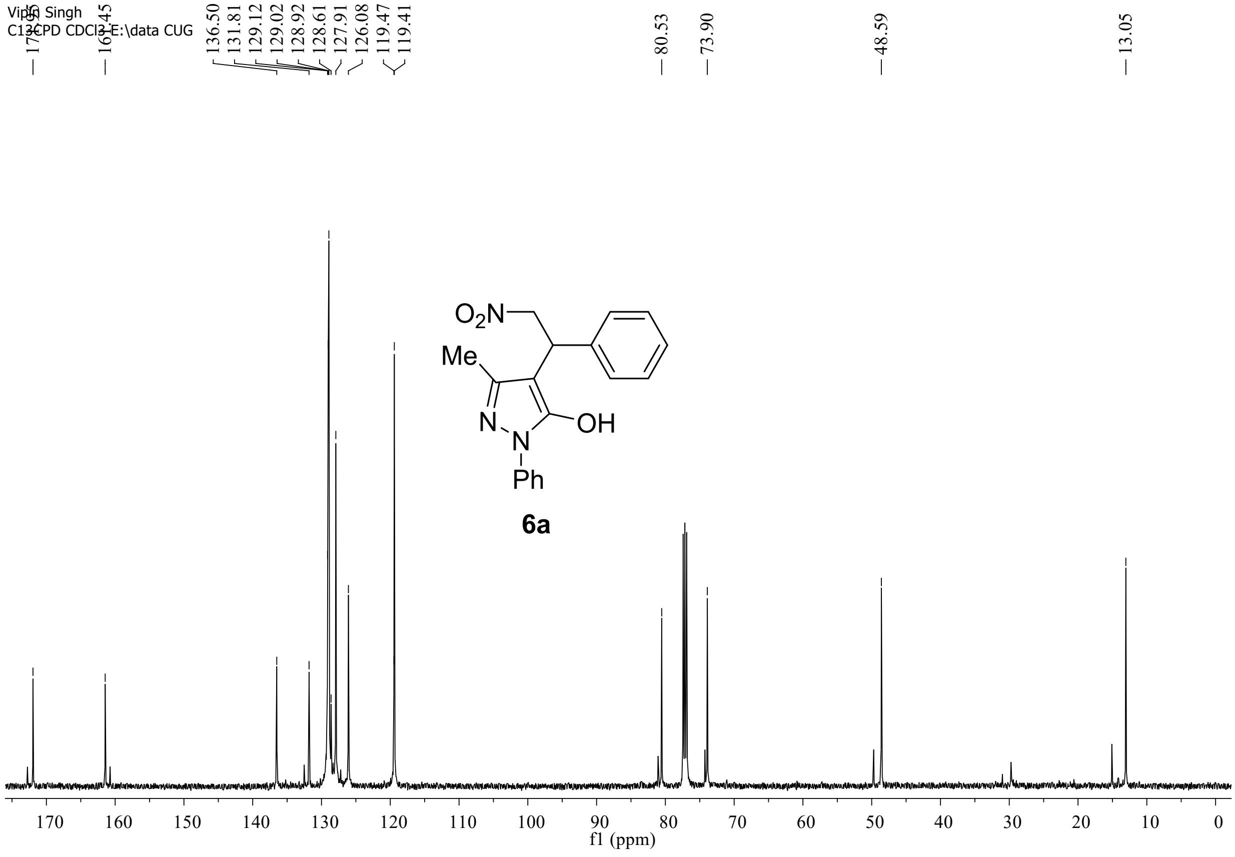
Vipin Singh
C13CPD CDCl3 E:\data CUG
136.50
131.81
129.12
129.02
128.92
128.61
127.91
126.08
119.47
119.41
80.53
73.90
48.59
13.05
O2N
Me
N
N
OH
Ph
6a
170 160 150 140 130 120 110 100 90 80 70 60 50 40 30 20 10 0
f1 (ppm)

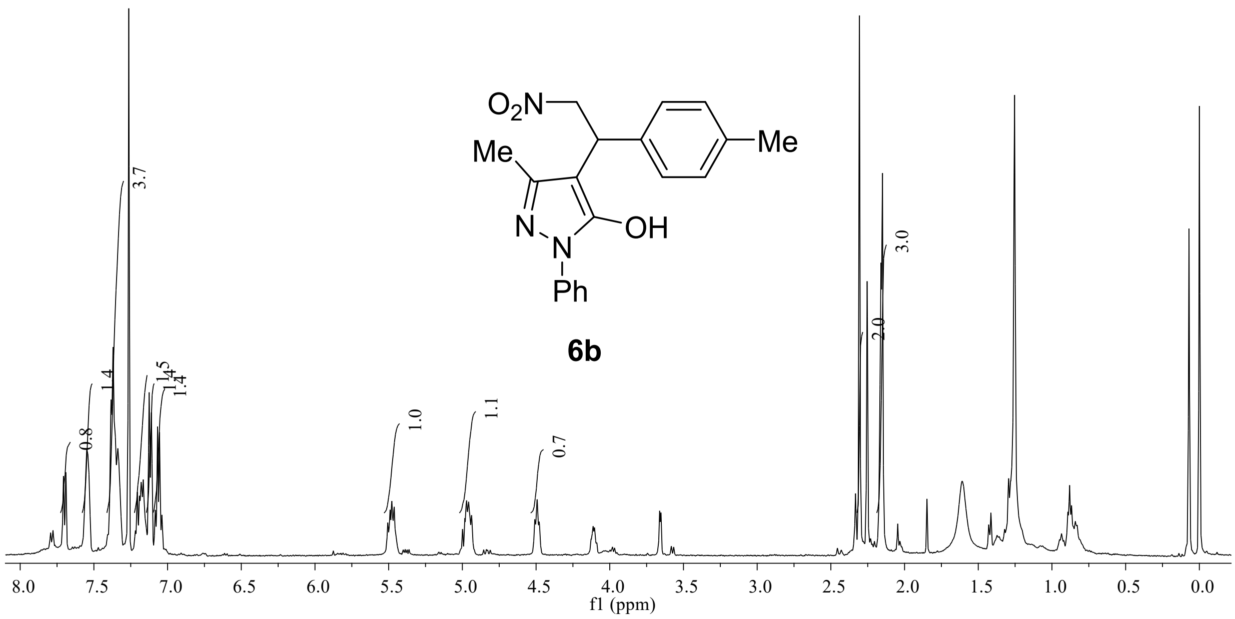
O2N
Me
Me
N
N
OH
Ph
6b
0.8
1.4
3.7
1.5
1.4
1.0
1.1
0.7
2.0
3.0
8.0 7.5 7.0 6.5 6.0 5.5 5.0 4.5 4.0 3.5 3.0 2.5 2.0 1.5 1.0 0.5 0.0
f1 (ppm)

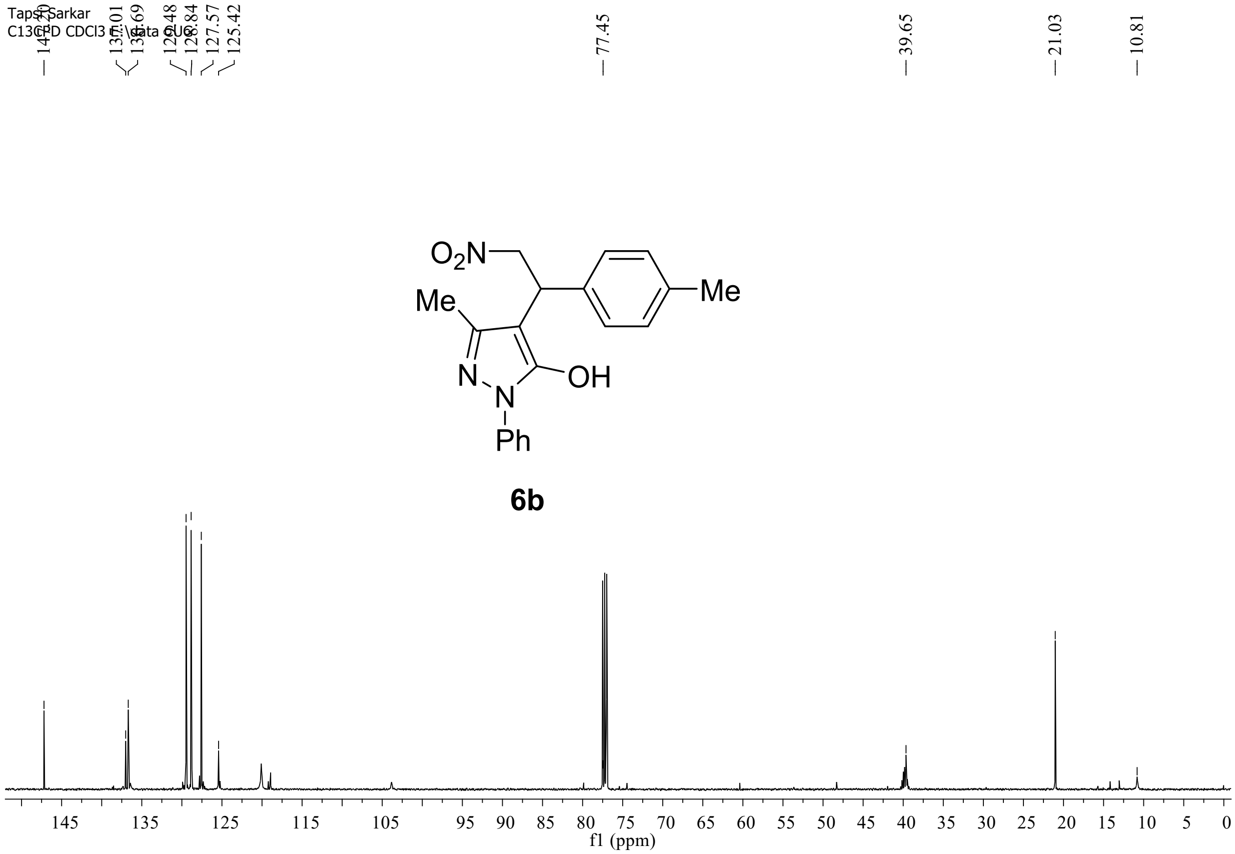
Tapsi Sarkar
C13CPD CDCl3
137.01
136.69
128.48
126.84
127.57
125.42
77.45
39.65
21.03
10.81
O2N
Me
Me
N
N
OH
Ph
6b
145 135 125 115 105 95 90 85 80 75 70 65 60 55 50 45 40 35 30 25 20 15 10 5 0
f1 (ppm)

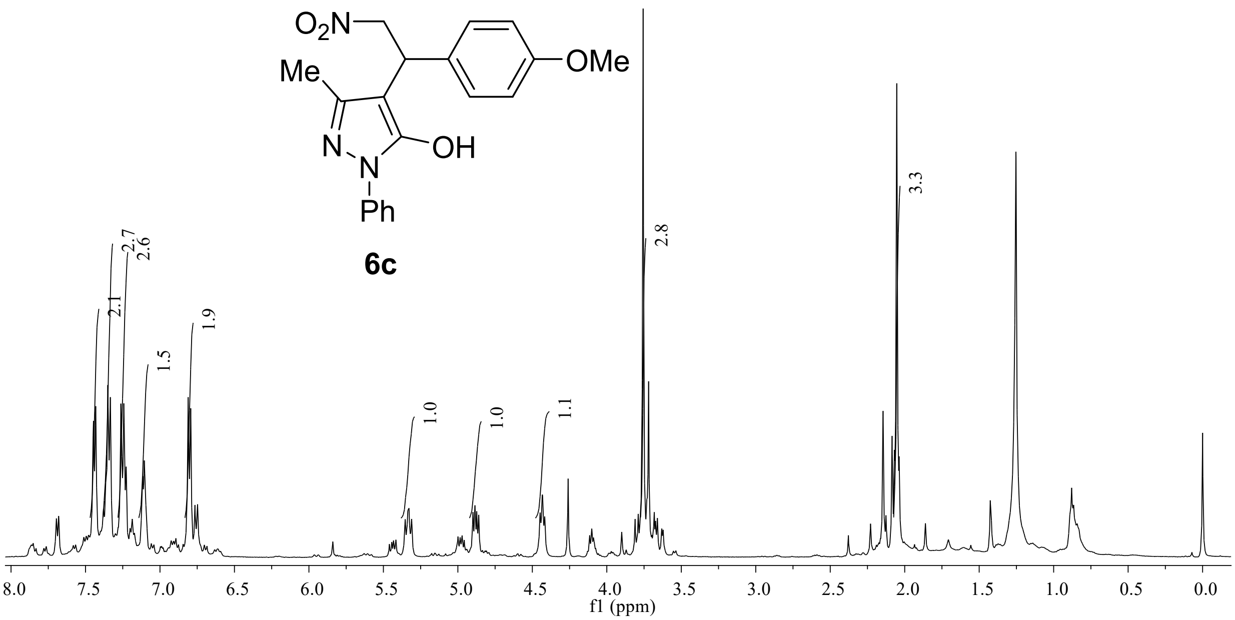
O2N
Me
OMe
N
N
OH
Ph
6c
2.1
2.7
2.6
1.5
1.9
1.0
1.0
1.1
2.8
3.3
8.0 7.5 7.0 6.5 6.0 5.5 5.0 4.5 4.0 3.5 3.0 2.5 2.0 1.5 1.0 0.5 0.0
f1 (ppm)

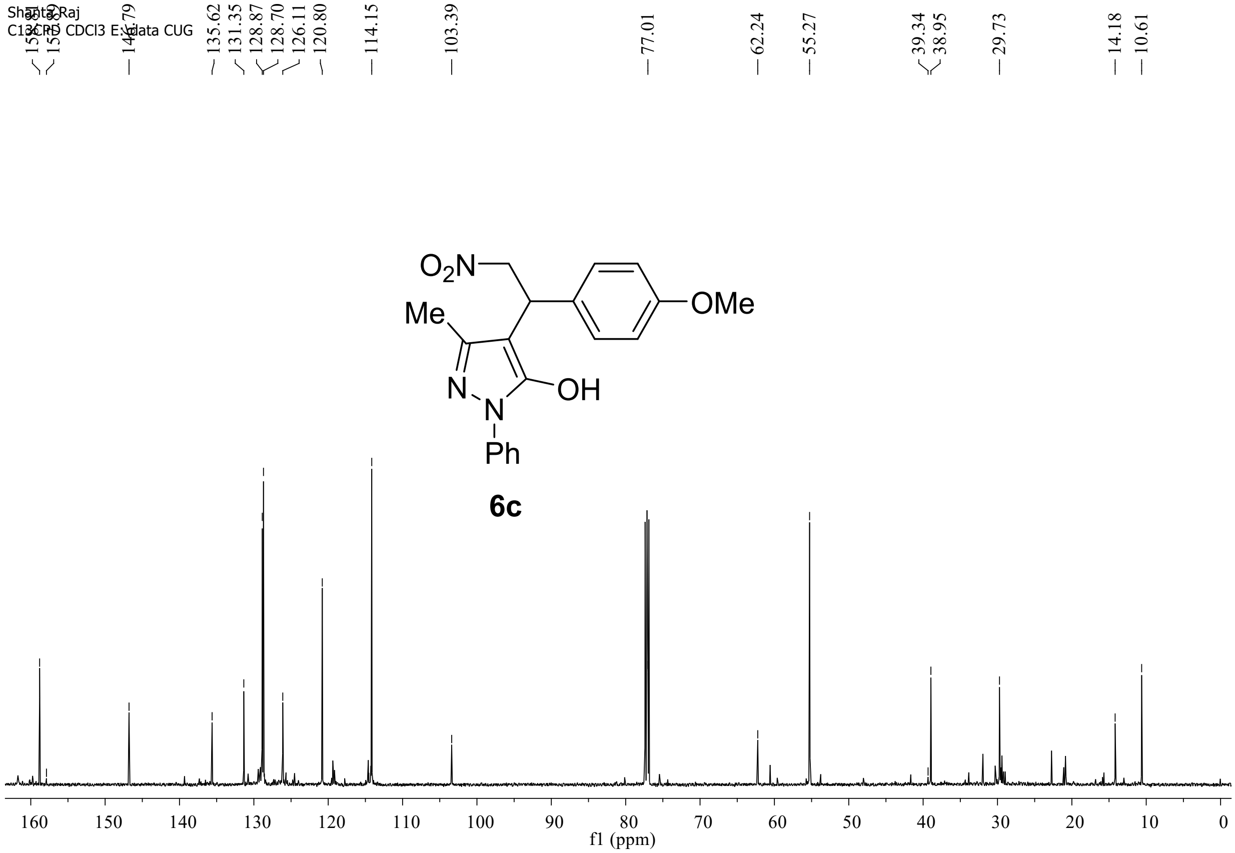
Shanta Raj
C13CPD CDCl3 E:\data CUG
146.79
135.62
131.35
128.87
128.70
126.11
120.80
114.15
103.39
77.01
62.24
55.27
39.34
38.95
29.73
14.18
10.61
O2N
Me
OMe
N
N
OH
Ph
6c
160 150 140 130 120 110 100 90 80 70 60 50 40 30 20 10 0
f1 (ppm)

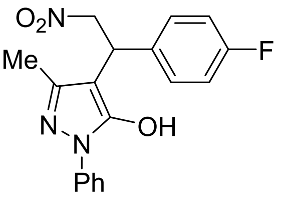

6d

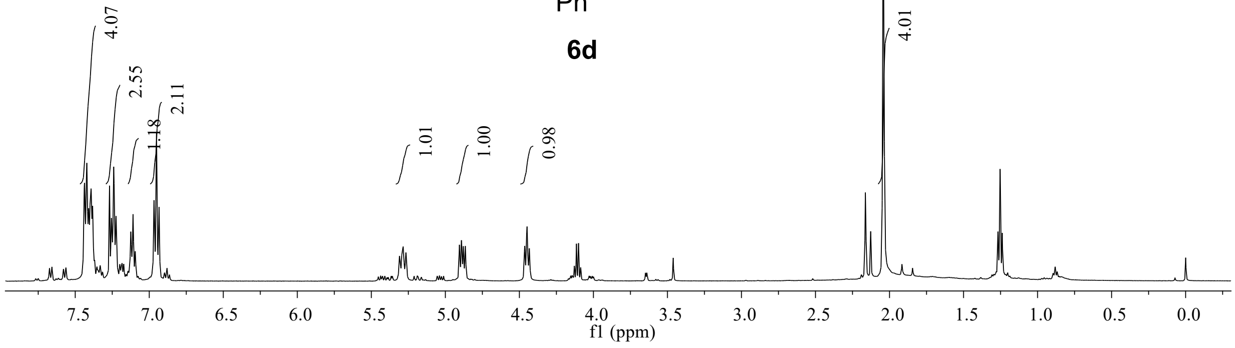
4.07
2.55
1.18
2.11
1.01
1.00
0.98
4.01
7.5 7.0 6.5 6.0 5.5 5.0 4.5 4.0 3.5 3.0 2.5 2.0 1.5 1.0 0.5 0.0
f1 (ppm)

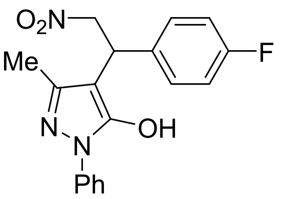

6d

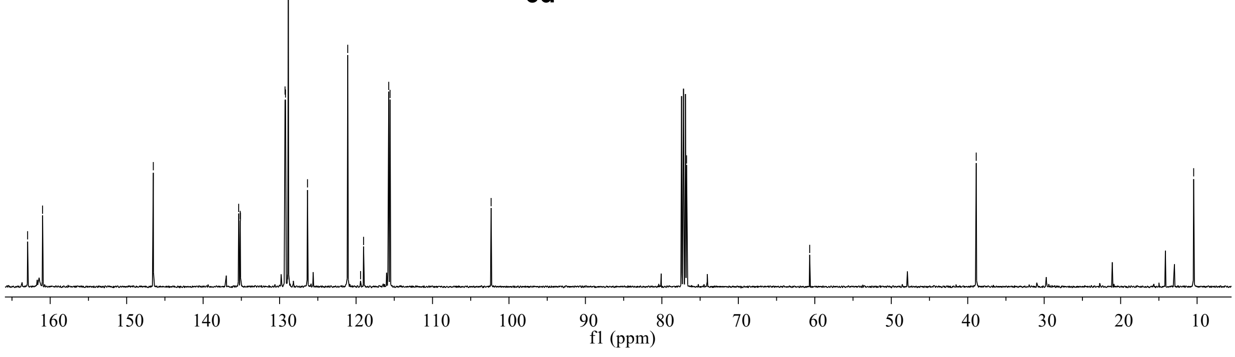
Shanta Raj
RC:SRL-114
C13CPD CDCl3 E:\data CUG
[illegible]
161.00
146.52
135.35
135.15
135.12
129.29
129.23
128.86
126.35
121.09
119.41
119.02
115.72
115.55
102.34
76.77
60.67
38.89
10.45
160 150 140 130 120 110 100 90 80 70 60 50 40 30 20 10
f1 (ppm)

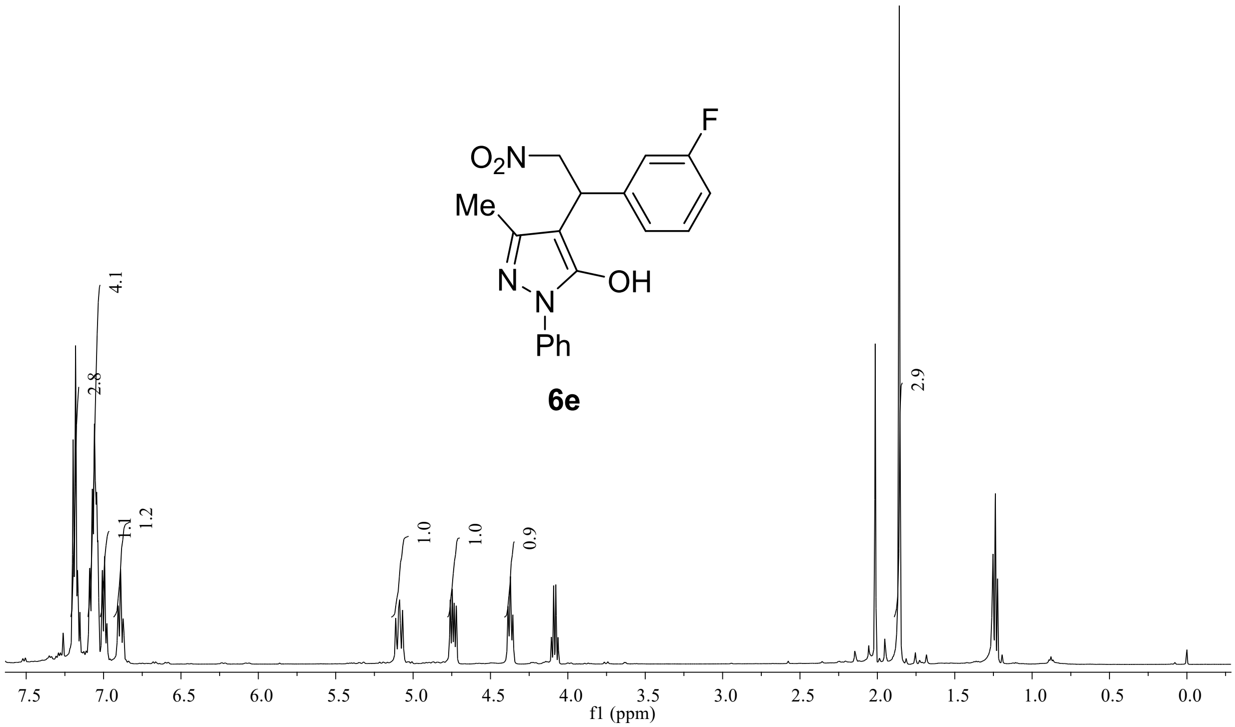
F
O2N
Me
N
N
OH
Ph
6e
4.1
2.8
1.1
1.2
1.0
1.0
0.9
2.9
7.5 7.0 6.5 6.0 5.5 5.0 4.5 4.0 3.5 3.0 2.5 2.0 1.5 1.0 0.5 0.0
f1 (ppm)

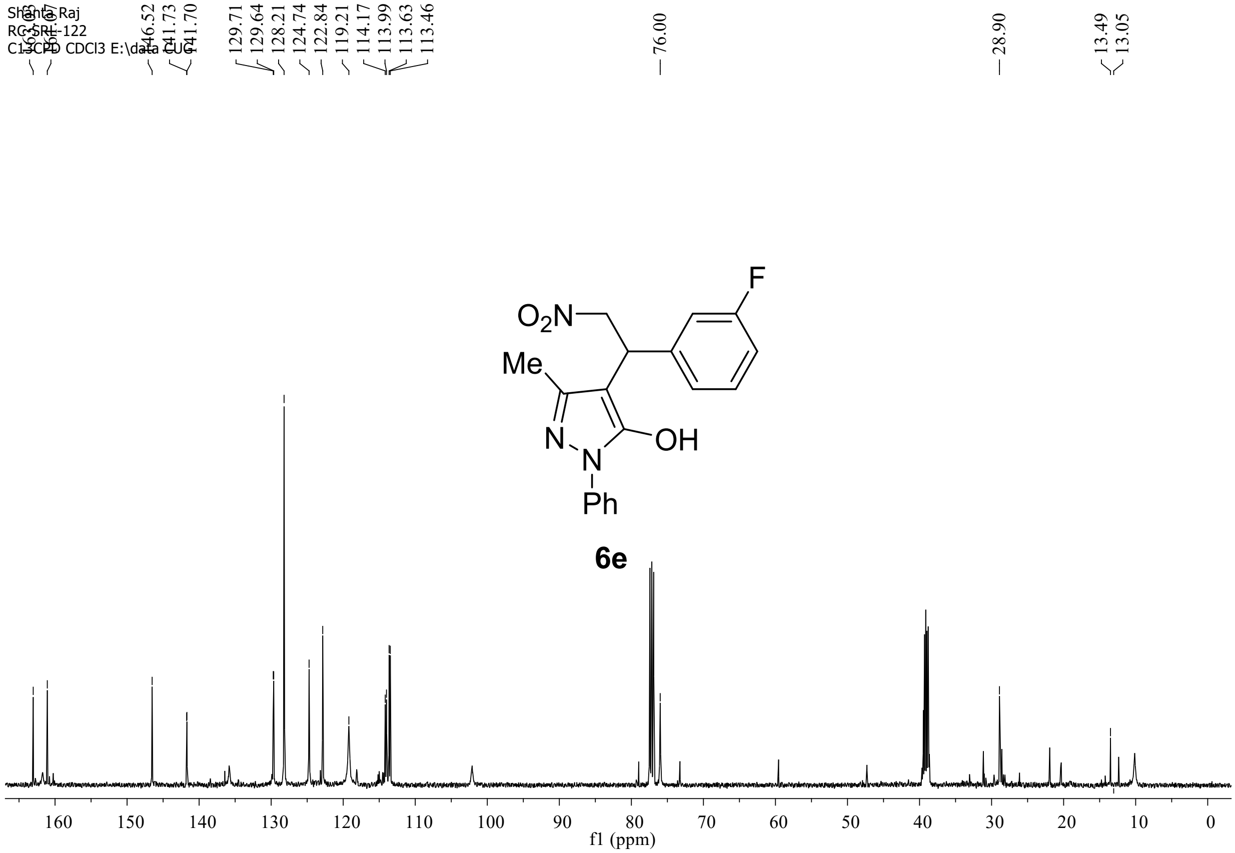
Shanta Raj
RG-SRL-122
C13CPD CDCl3 E:\data CUC
146.52
141.73
141.70
129.71
129.64
128.21
124.74
122.84
119.21
114.17
113.99
113.63
113.46
76.00
28.90
13.49
13.05
F
O2N
Me
N
N
OH
Ph
6e
160 150 140 130 120 110 100 90 80 70 60 50 40 30 20 10 0
f1 (ppm)

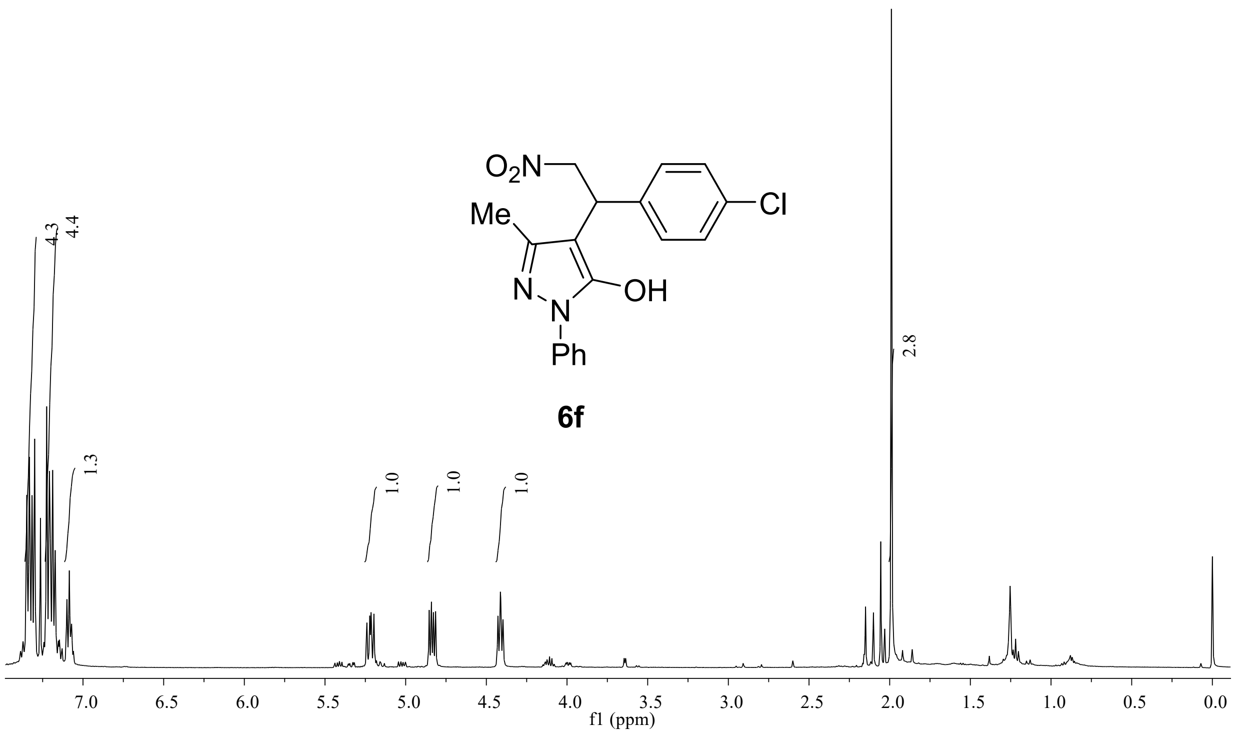
O2N
Me
Cl
N
N
OH
Ph
6f
4.3
4.4
1.3
1.0
1.0
1.0
2.8
7.0 6.5 6.0 5.5 5.0 4.5 4.0 3.5 3.0 2.5 2.0 1.5 1.0 0.5 0.0
f1 (ppm)

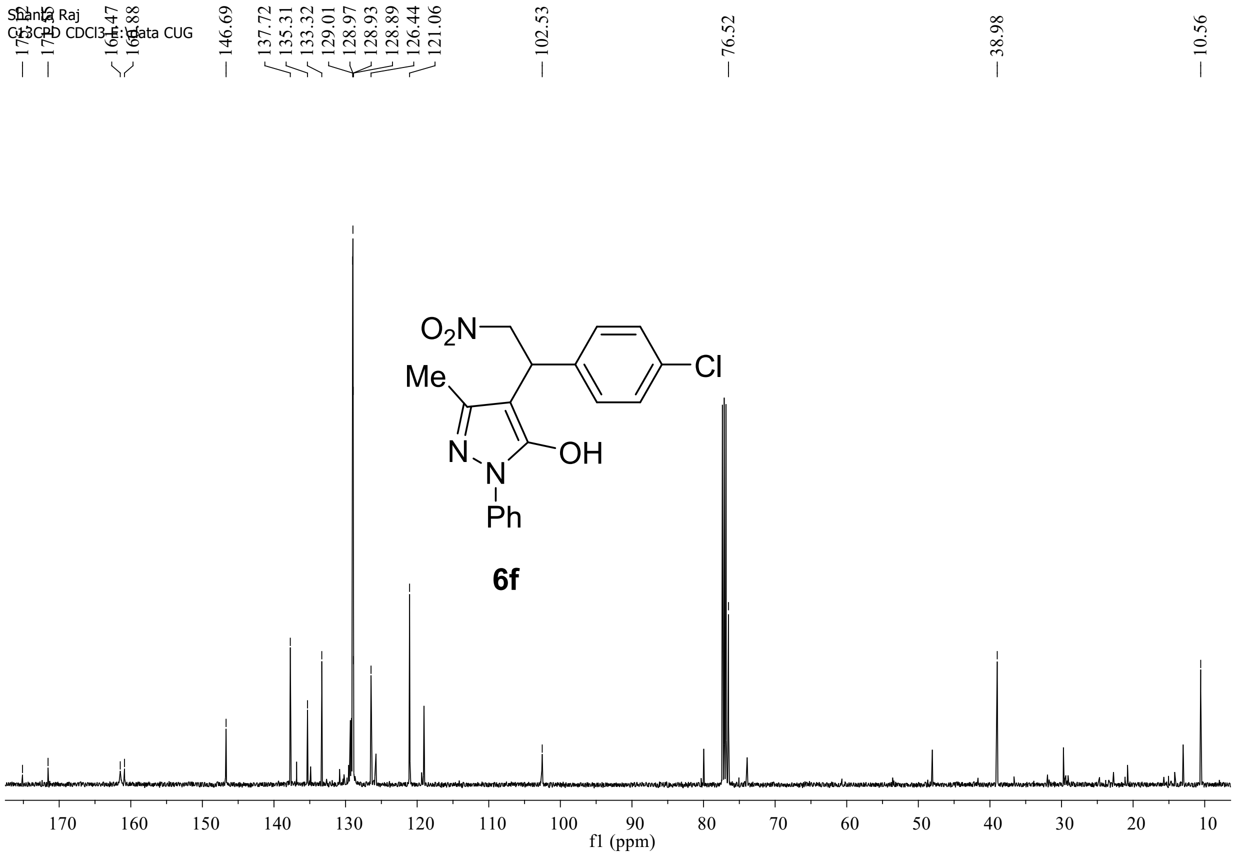
Shanta Raj
C13CPD CDCl3 E:\data CUG
146.69
137.72
135.31
133.32
129.01
128.97
128.93
128.89
126.44
121.06
102.53
76.52
38.98
10.56
O2N
Me
Cl
N
N
OH
Ph
6f
170 160 150 140 130 120 110 100 90 80 70 60 50 40 30 20 10
f1 (ppm)

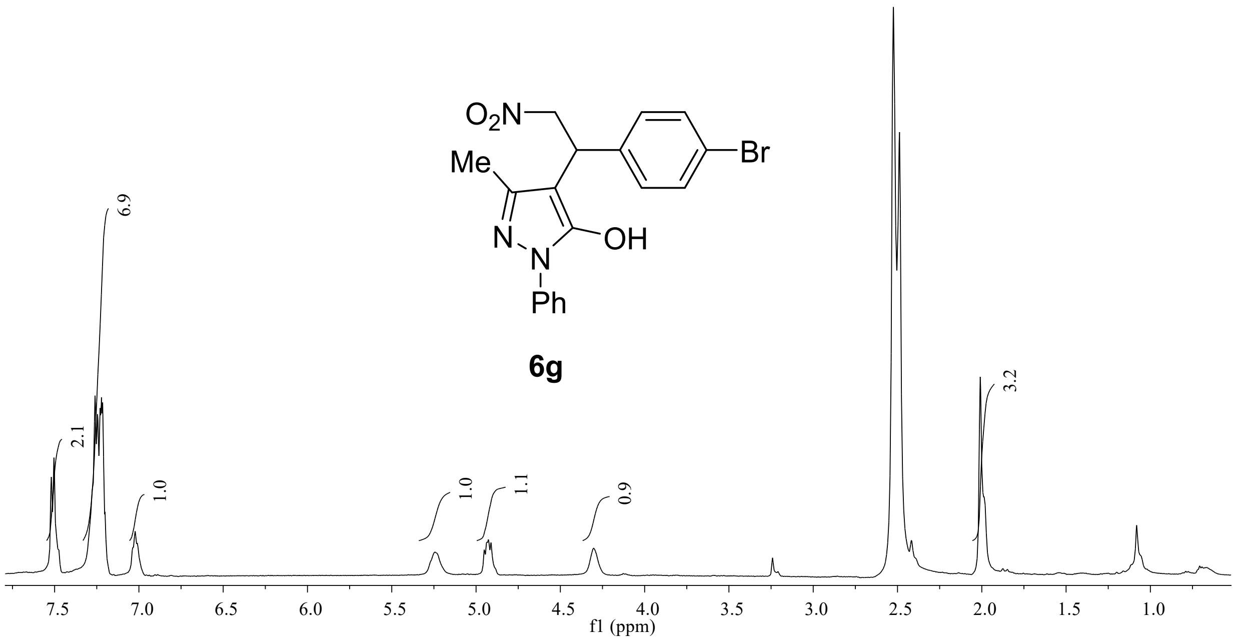

O_2N
Me
Br
N
N
OH
Ph
6g
6.9
2.1
1.0
1.0
1.1
0.9
3.2
7.5 7.0 6.5 6.0 5.5 5.0 4.5 4.0 3.5 3.0 2.5 2.0 1.5 1.0
f1 (ppm)

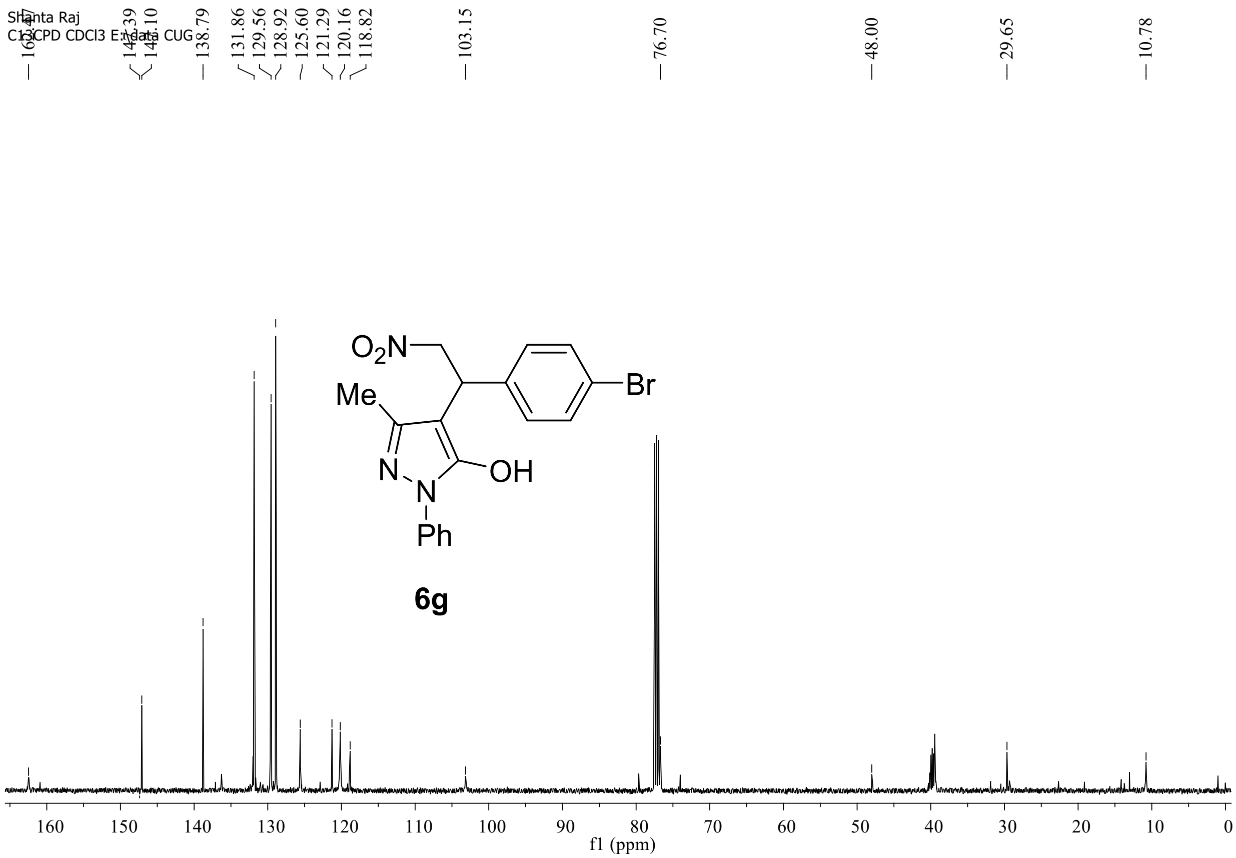

Shanta Raj
138.79
131.86
129.56
128.92
125.60
121.29
120.16
118.82
103.15
76.70
48.00
29.65
10.78
O_2N
Me
Br
N
N
OH
Ph
6g
160 150 140 130 120 110 100 90 80 70 60 50 40 30 20 10 0
f1 (ppm)

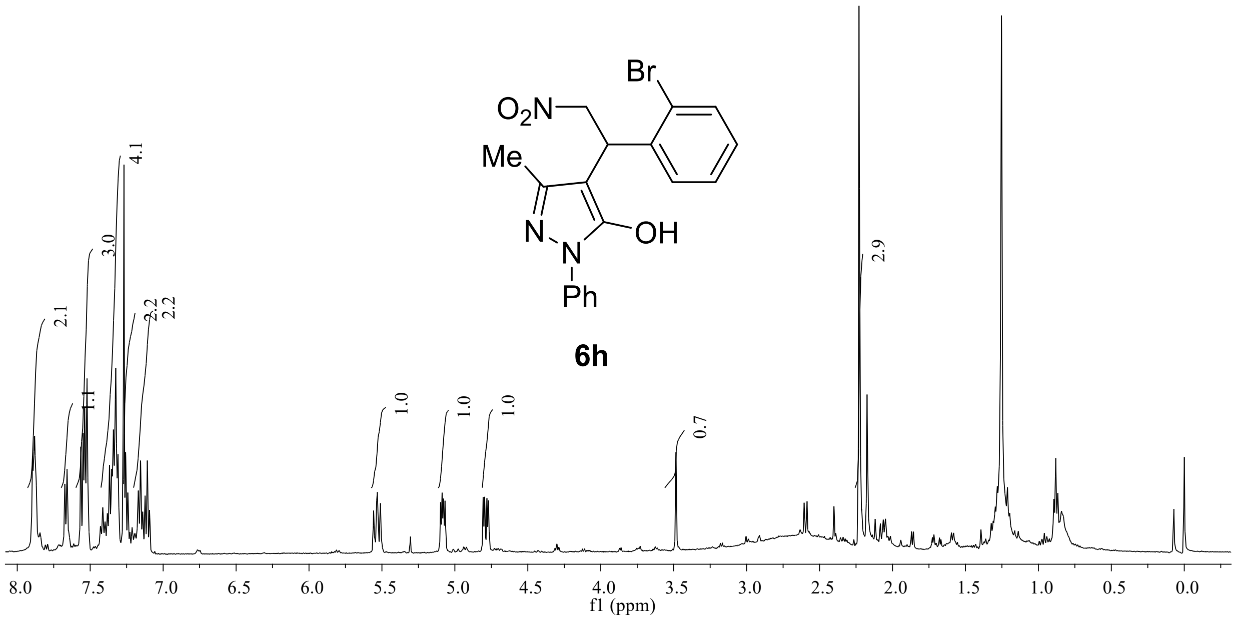
Br
O2N
Me
N
N
OH
Ph
6h
2.1
1.1
3.0
4.1
2.2
2.2
1.0
1.0
1.0
0.7
2.9
f1 (ppm)

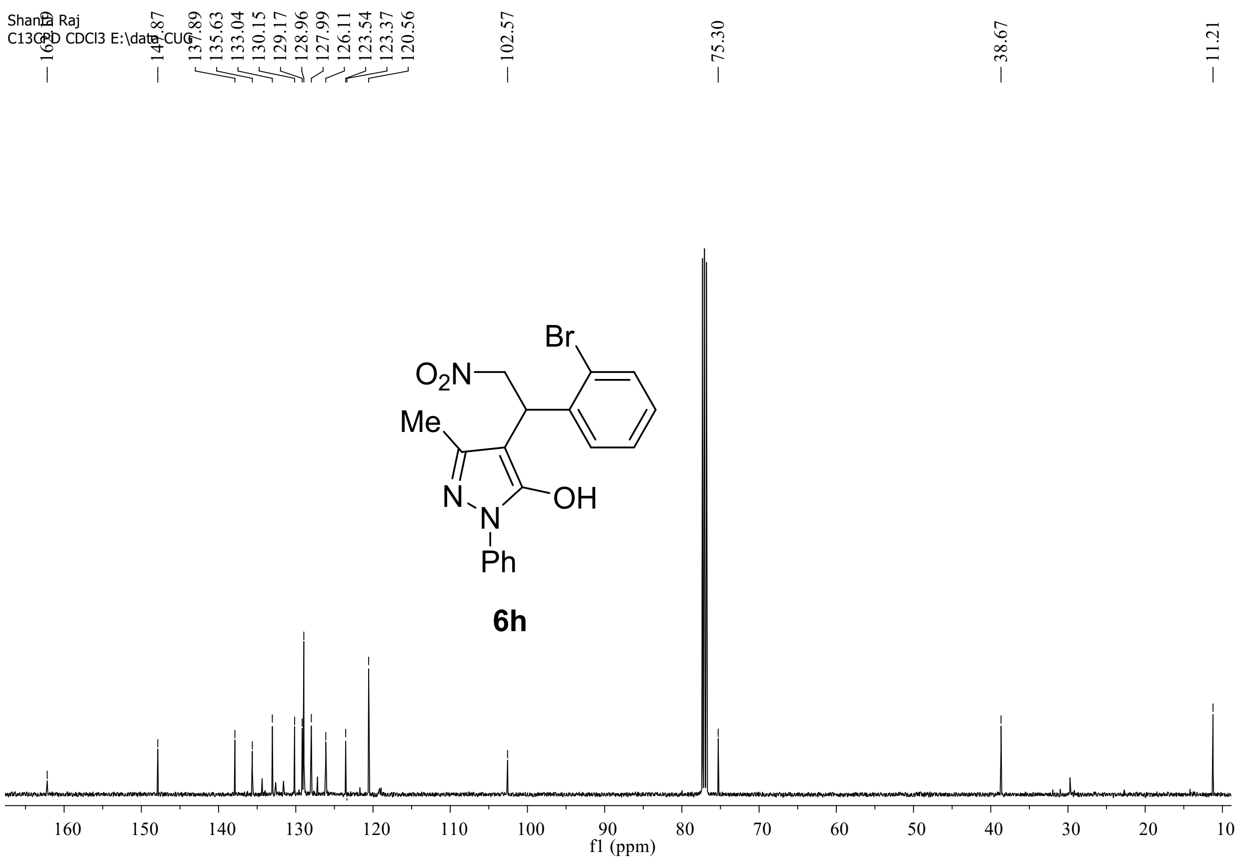
Shanta Raj
C13CPD CDCl3 E:\data CUG
137.89
135.63
133.04
130.15
129.17
128.96
127.99
126.11
123.54
123.37
120.56
102.57
75.30
38.67
11.21
Br
O2N
Me
N
N
OH
Ph
6h
f1 (ppm)

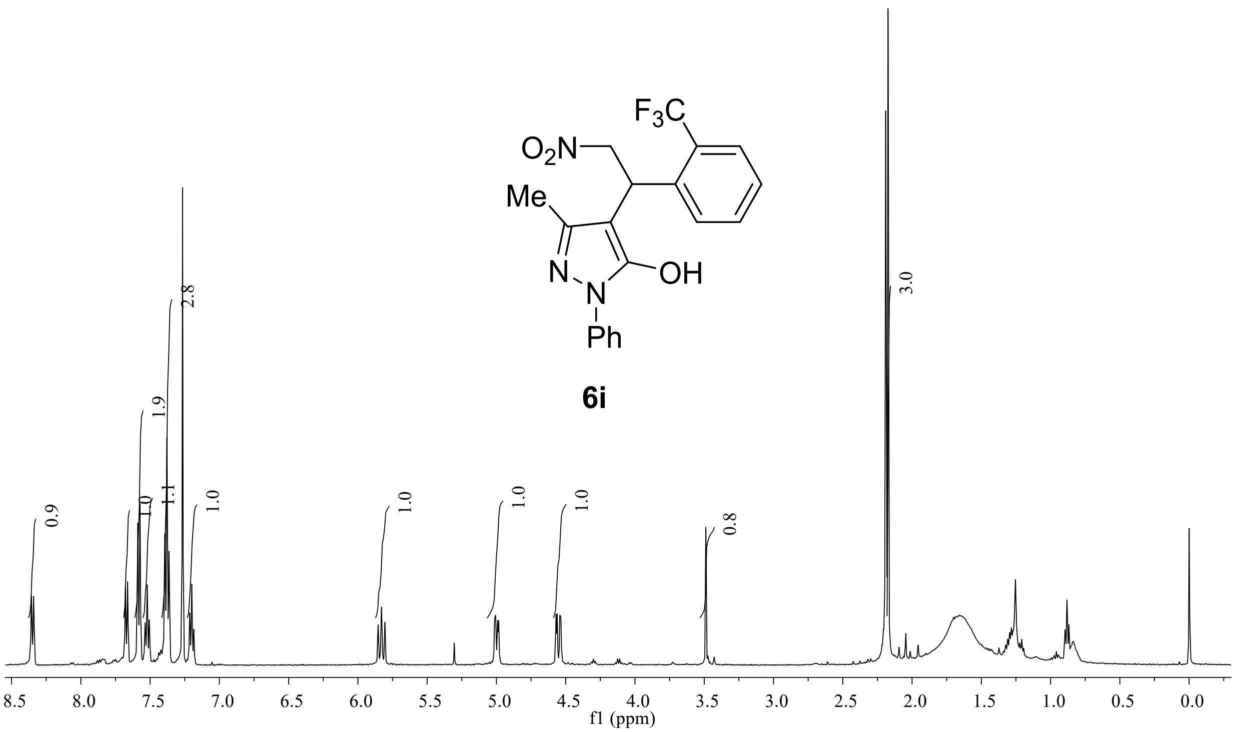
F3C
O2N
Me
N
N
OH
Ph
6i
0.9
1.0
1.1
1.9
2.8
1.0
1.0
1.0
1.0
0.8
3.0
f1 (ppm)

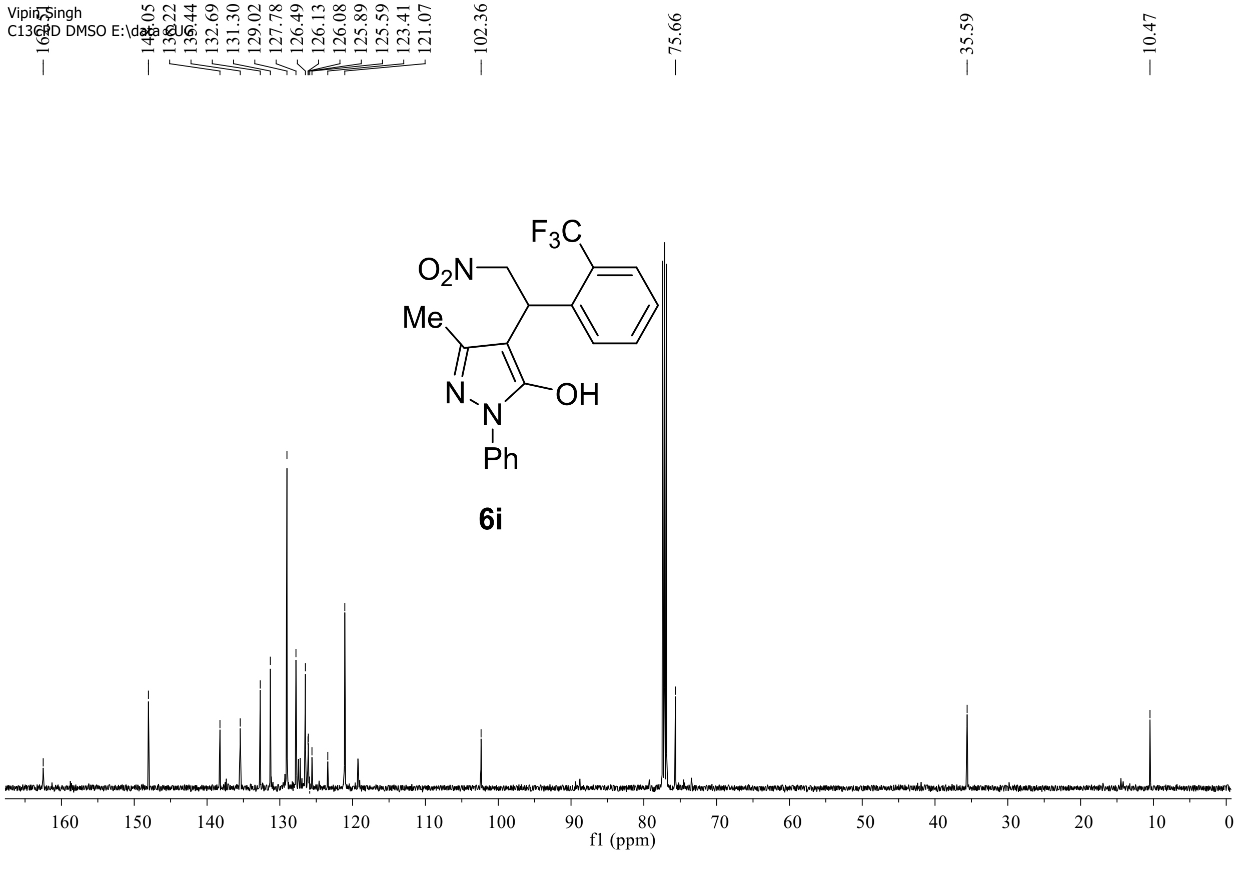
Vipin Singh
C13CPD DMSO E:\data cU6
148.05
138.22
135.44
132.69
131.30
129.02
127.78
126.49
126.13
126.08
125.89
125.59
123.41
121.07
102.36
75.66
35.59
10.47
F3C
O2N
Me
N
N
OH
Ph
6i
f1 (ppm)

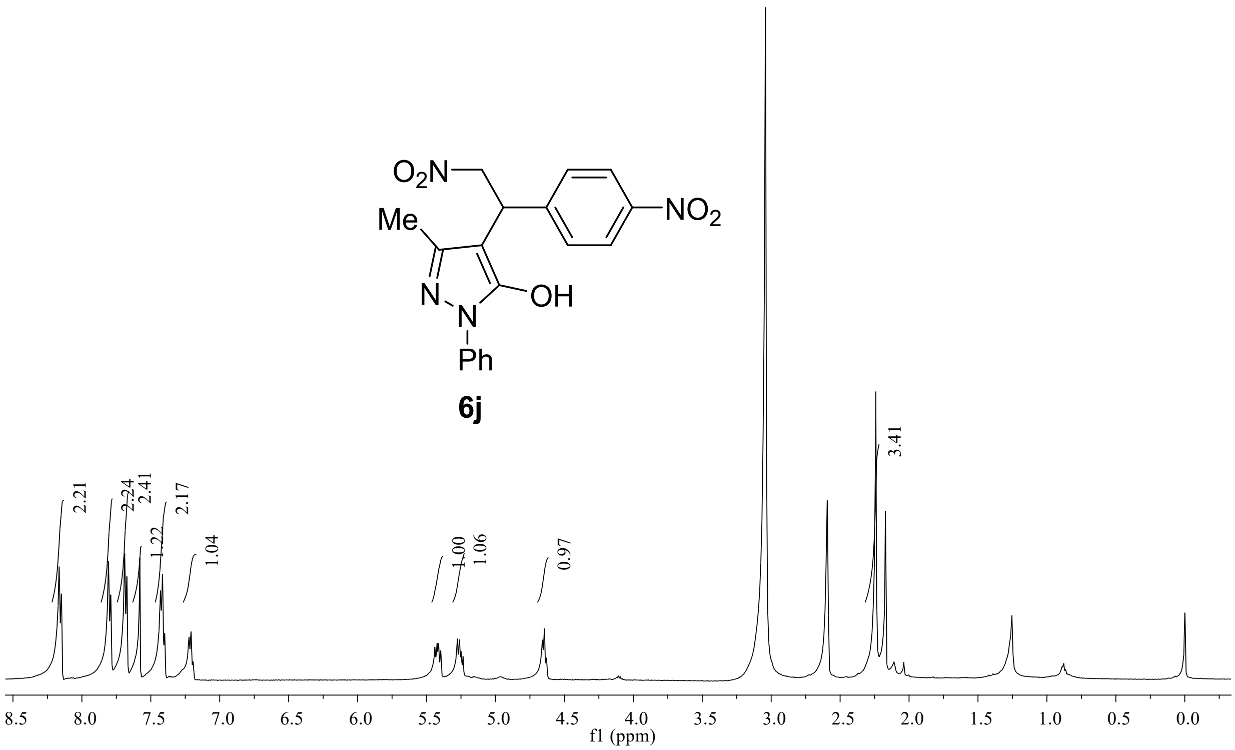

O_2N
Me
NO_2
N
N
OH
Ph
6j
2.21
2.24
2.41
1.22
2.17
1.04
1.00
1.06
0.97
3.41
8.5 8.0 7.5 7.0 6.5 6.0 5.5 5.0 4.5 4.0 3.5 3.0 2.5 2.0 1.5 1.0 0.5 0.0
f1 (ppm)

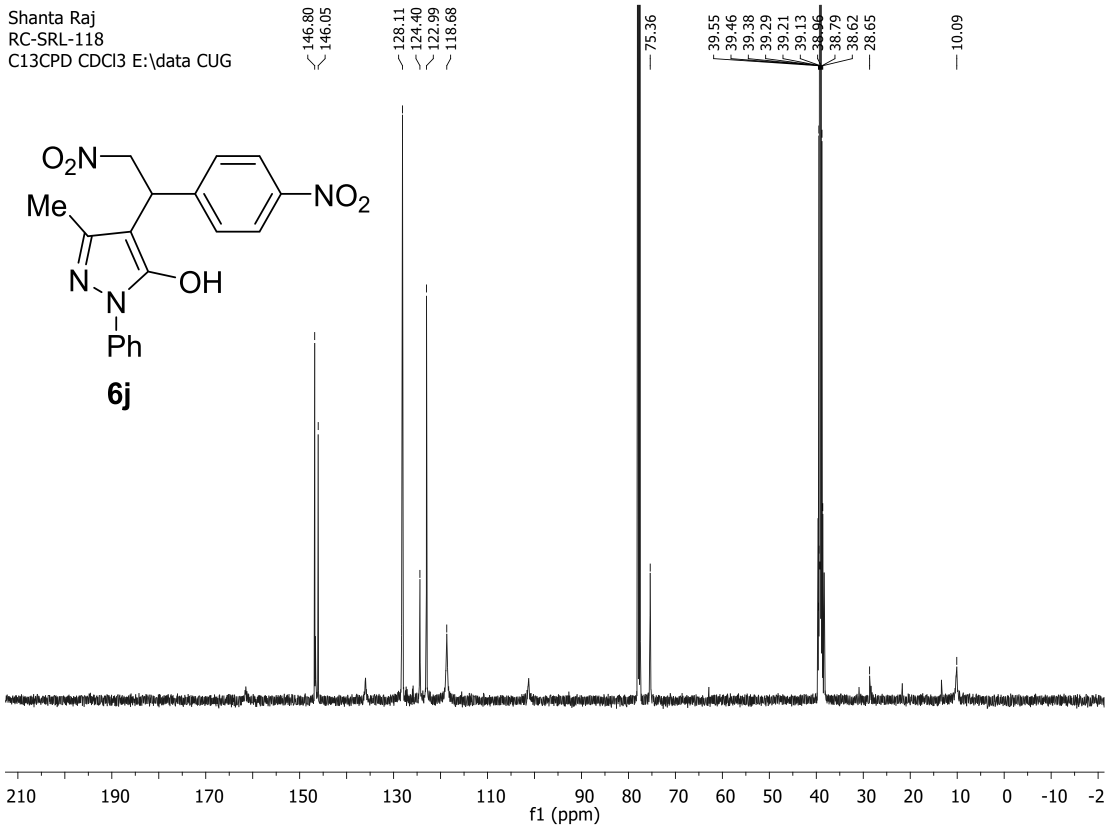

Shanta Raj
RC-SRL-118
C13CPD CDCl3 E:\data CUG
146.80
146.05
128.11
124.40
122.99
118.68
75.36
39.55
39.46
39.38
39.29
39.21
39.13
38.96
38.79
38.62
28.65
10.09
O_2N
Me
NO_2
N
N
OH
Ph
6j
210 190 170 150 130 110 90 80 70 60 50 40 30 20 10 0 -10 -2
f1 (ppm)

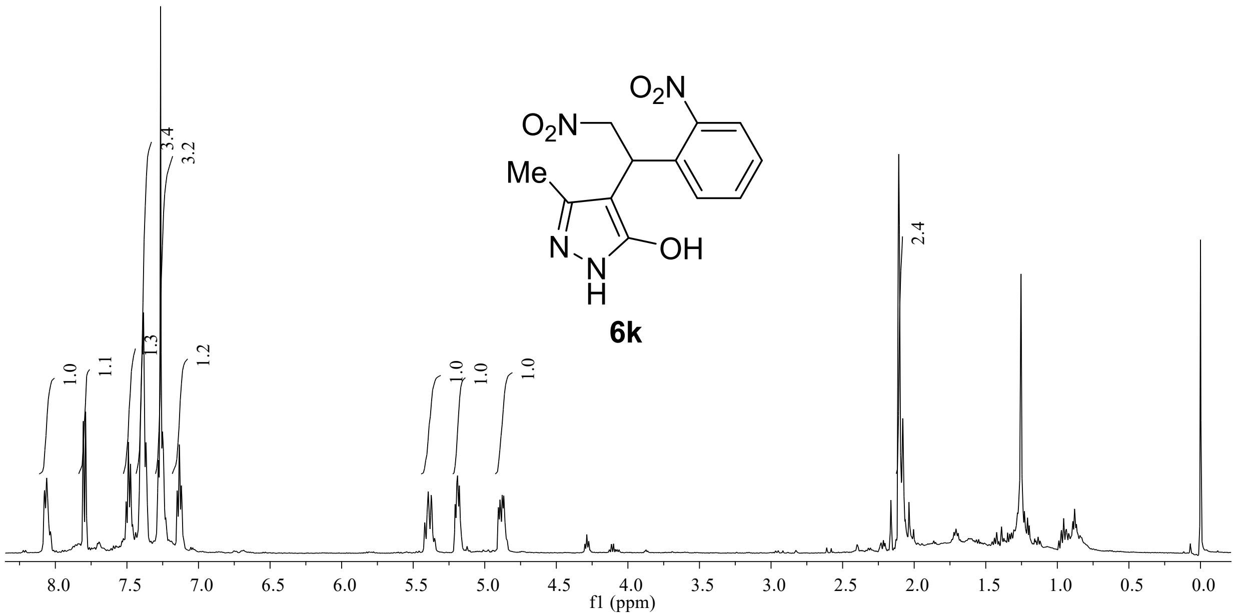
O2N
O2N
Me
N
N
H
OH
6k
1.0
1.1
1.3
3.4
3.2
1.2
1.0
1.0
1.0
2.4
8.0 7.5 7.0 6.5 6.0 5.5 5.0 4.5 4.0 3.5 3.0 2.5 2.0 1.5 1.0 0.5 0.0
f1 (ppm)

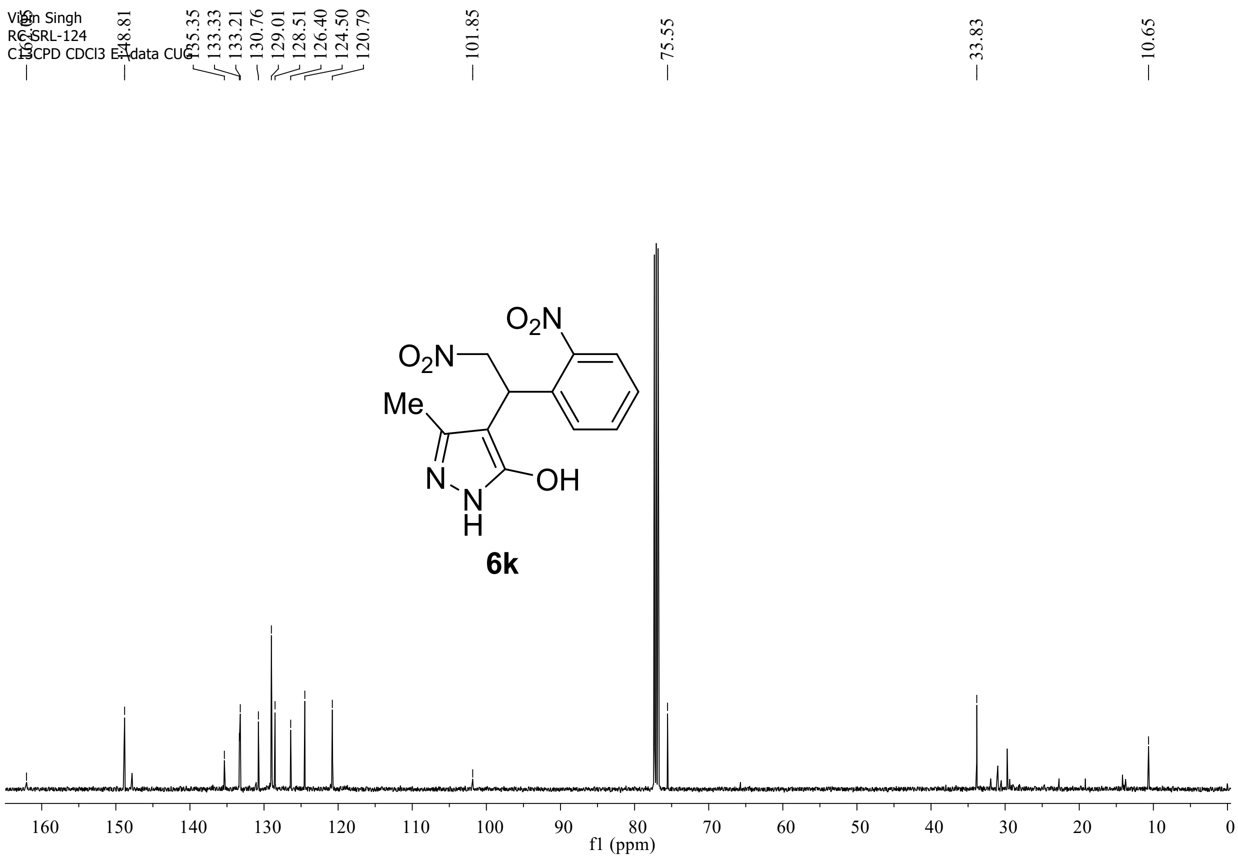
Vipin Singh
RC-SRL-124
C13CPD CDCl3 E-data CUC
148.81
135.35
133.33
133.21
130.76
129.01
128.51
126.40
124.50
120.79
101.85
75.55
33.83
10.65
O2N
O2N
Me
N
N
H
OH
6k
160 150 140 130 120 110 100 90 80 70 60 50 40 30 20 10 0
f1 (ppm)

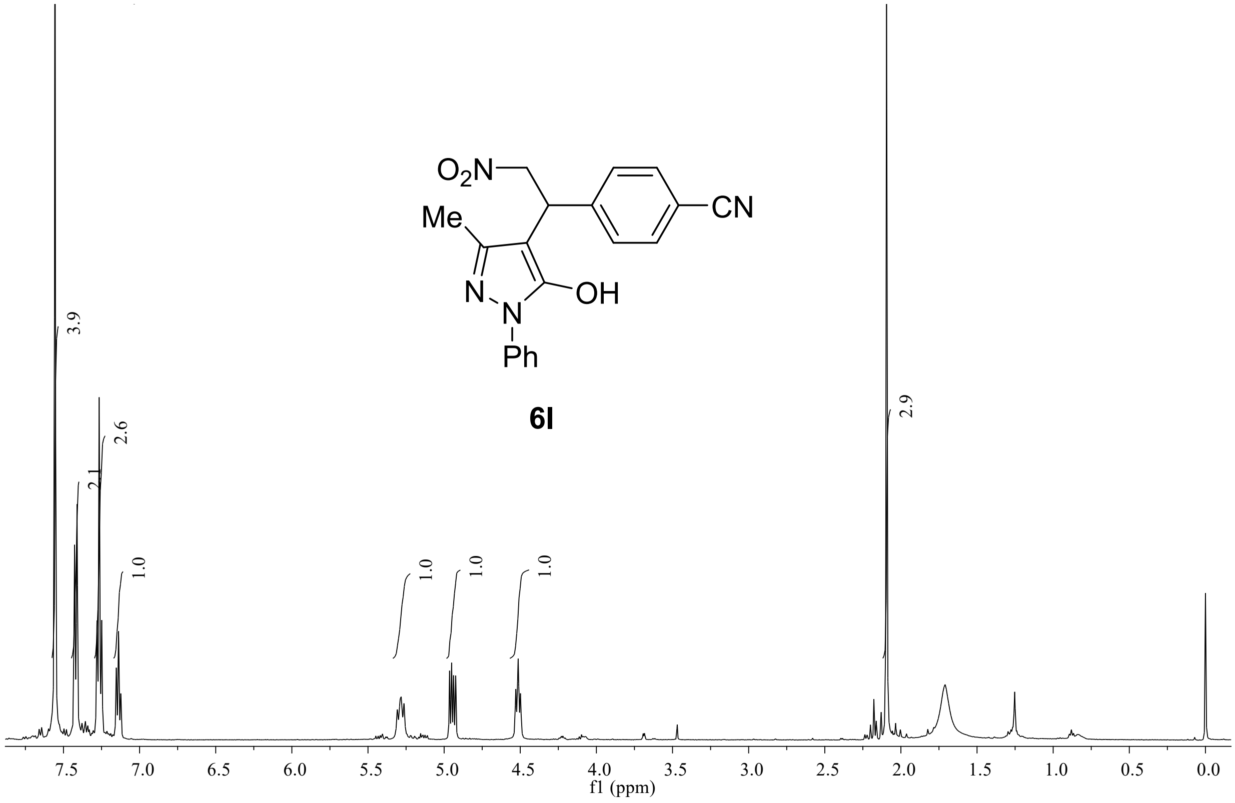

O_2N
Me
CN
N
N
OH
Ph
6l
3.9
2.1
2.6
1.0
1.0
1.0
1.0
2.9
f1 (ppm)

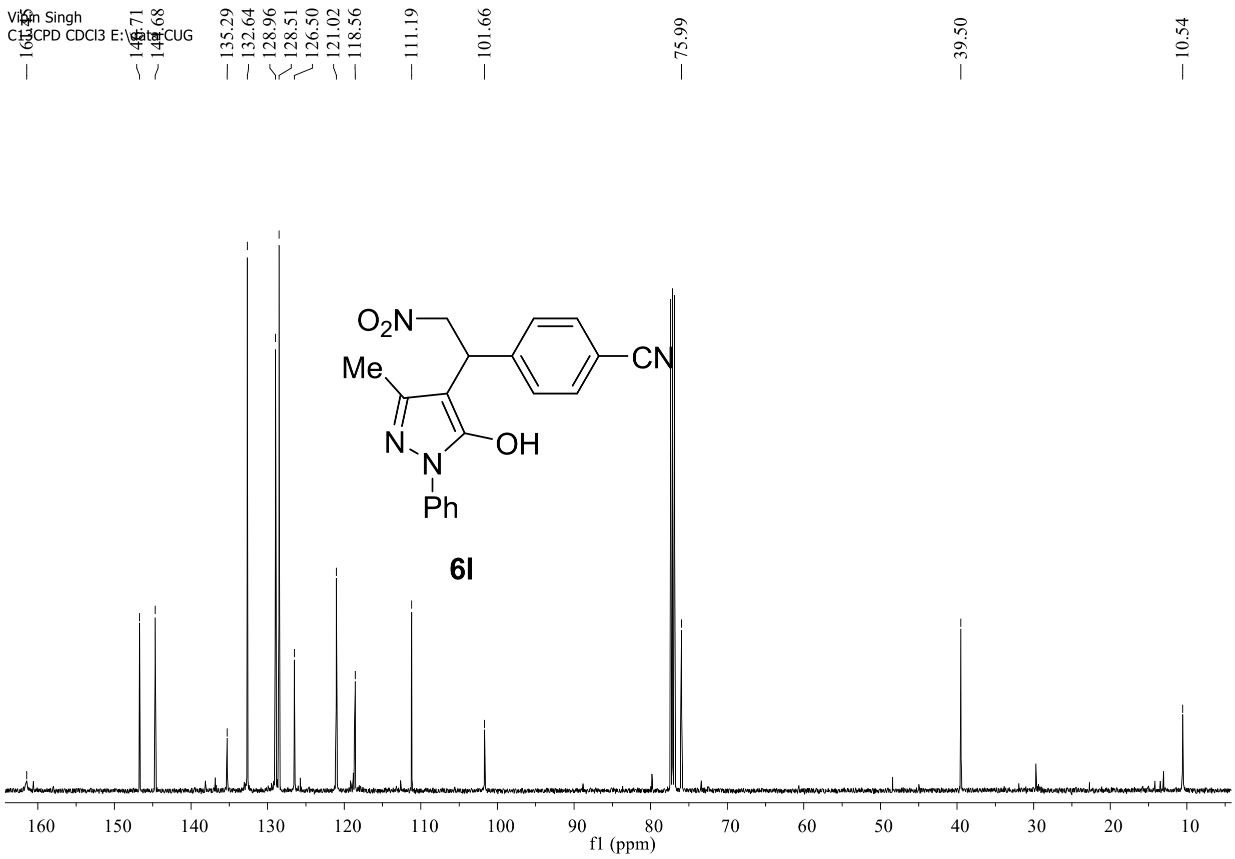

Vipin Singh
C13CPD CDCl3 E:\data\CUG
146.71
144.68
135.29
132.64
128.96
128.51
126.50
121.02
118.56
111.19
101.66
75.99
39.50
10.54
O_2N
Me
CN
N
N
OH
Ph
6l
f1 (ppm)

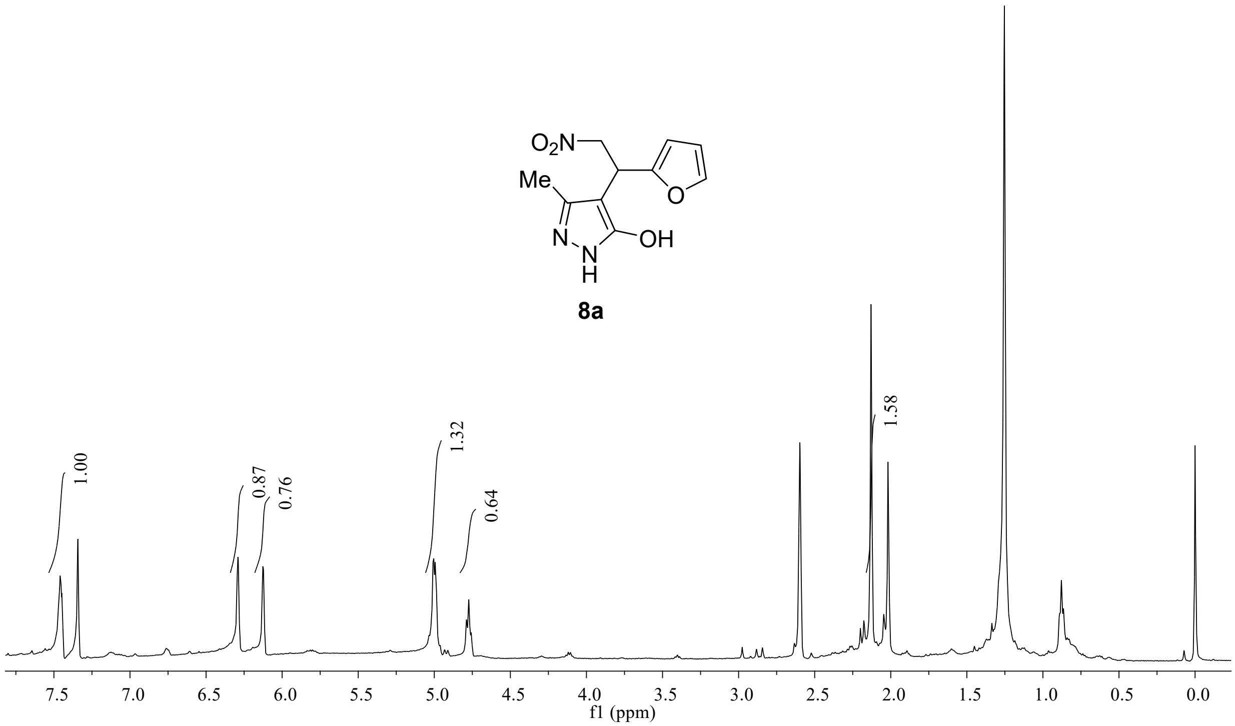

O_2N
Me
N
N
H
OH
O
8a
1.00
0.87
0.76
1.32
0.64
1.58
7.5 7.0 6.5 6.0 5.5 5.0 4.5 4.0 3.5 3.0 2.5 2.0 1.5 1.0 0.5 0.0
f1 (ppm)

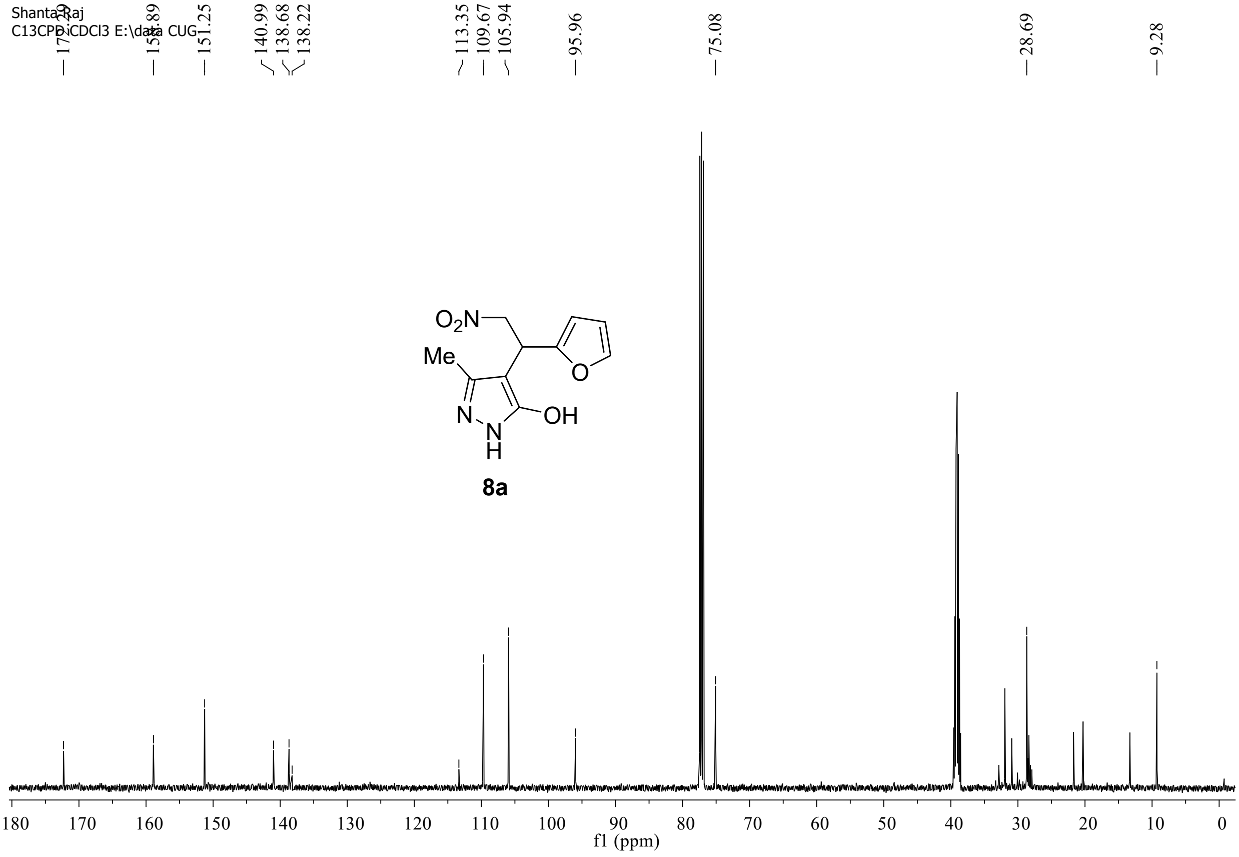

Shanta Raj
C13CPD CDCl3 E:\data CUG
151.25
140.99
138.68
138.22
113.35
109.67
105.94
95.96
75.08
28.69
9.28
O_2N
Me
N
N
H
OH
O
8a
180 170 160 150 140 130 120 110 100 90 80 70 60 50 40 30 20 10 0
f1 (ppm)

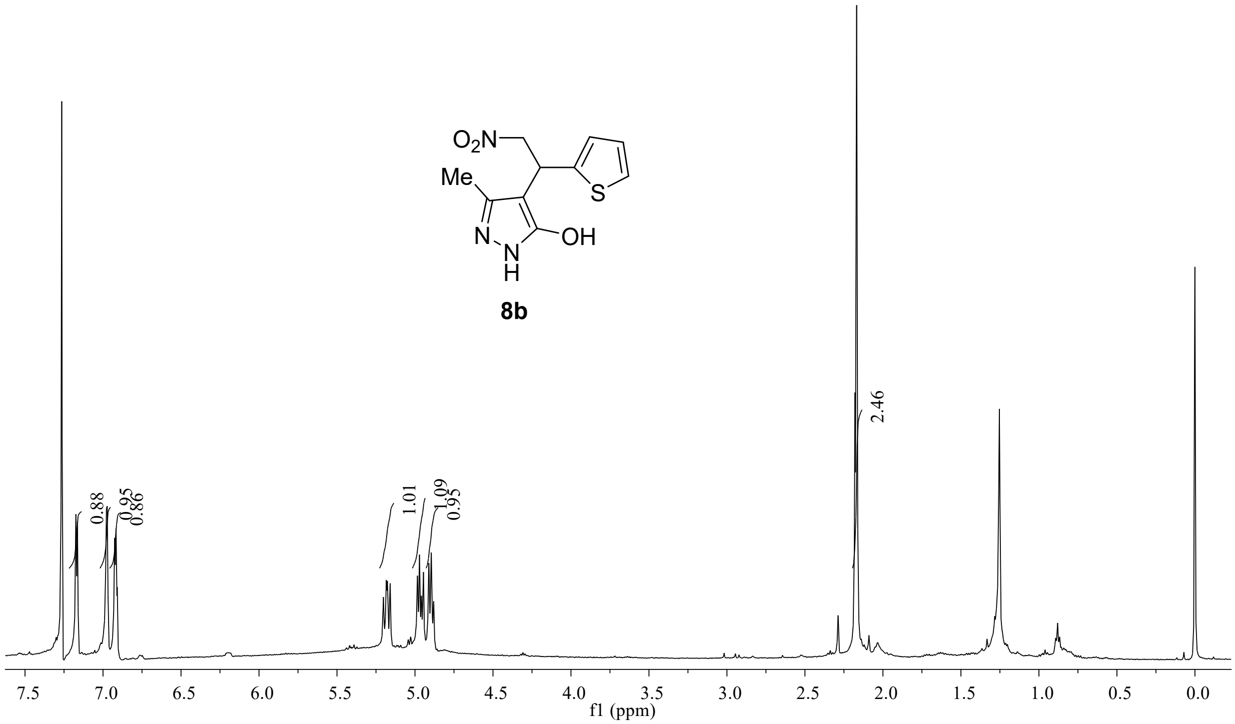

O_2N
Me
S
N
N
H
OH
8b
0.88
0.95
0.86
1.01
1.09
0.95
2.46
7.5 7.0 6.5 6.0 5.5 5.0 4.5 4.0 3.5 3.0 2.5 2.0 1.5 1.0 0.5 0.0
f1 (ppm)

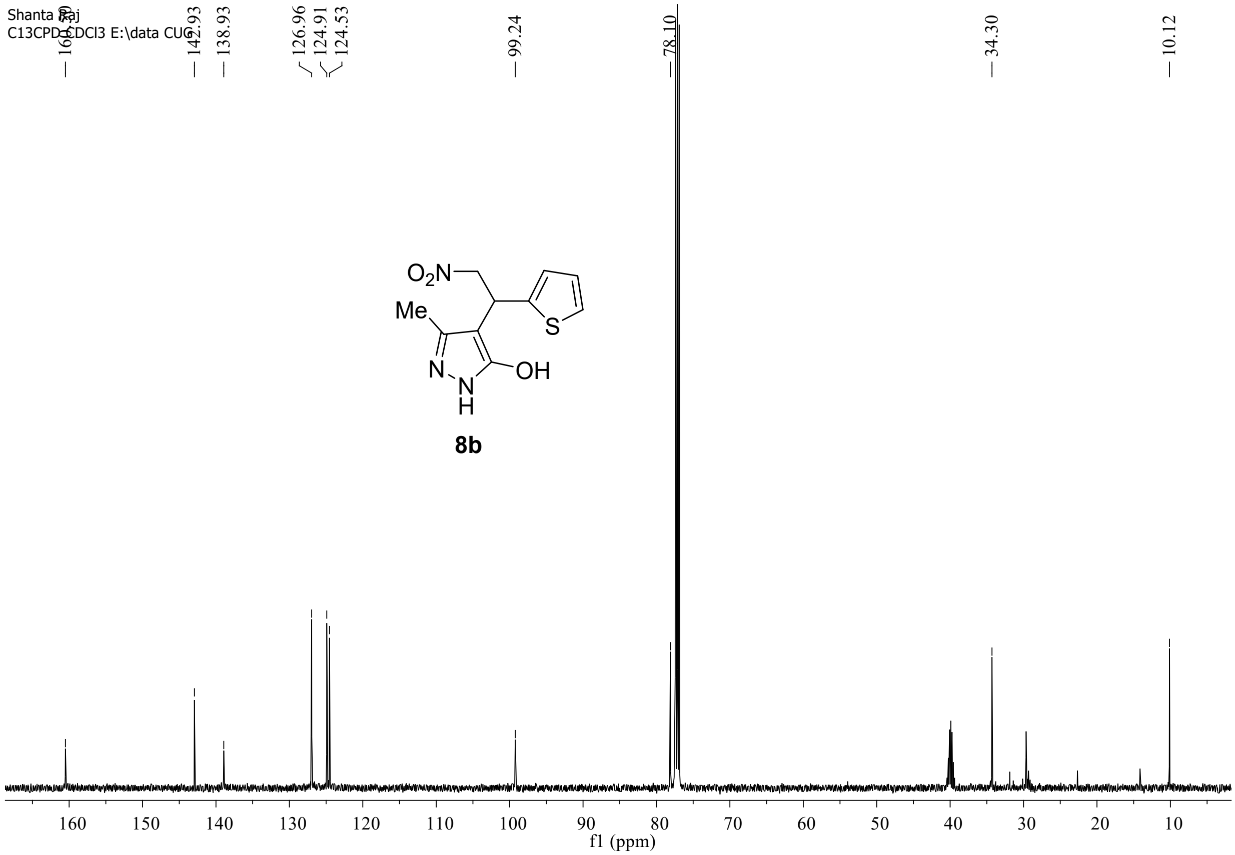

Shanta Raj
C13CPD CDCl3 E:\data CUG
142.93
138.93
126.96
124.91
124.53
99.24
78.10
34.30
10.12
O_2N
Me
S
N
N
H
OH
8b
160 150 140 130 120 110 100 90 80 70 60 50 40 30 20 10
f1 (ppm)

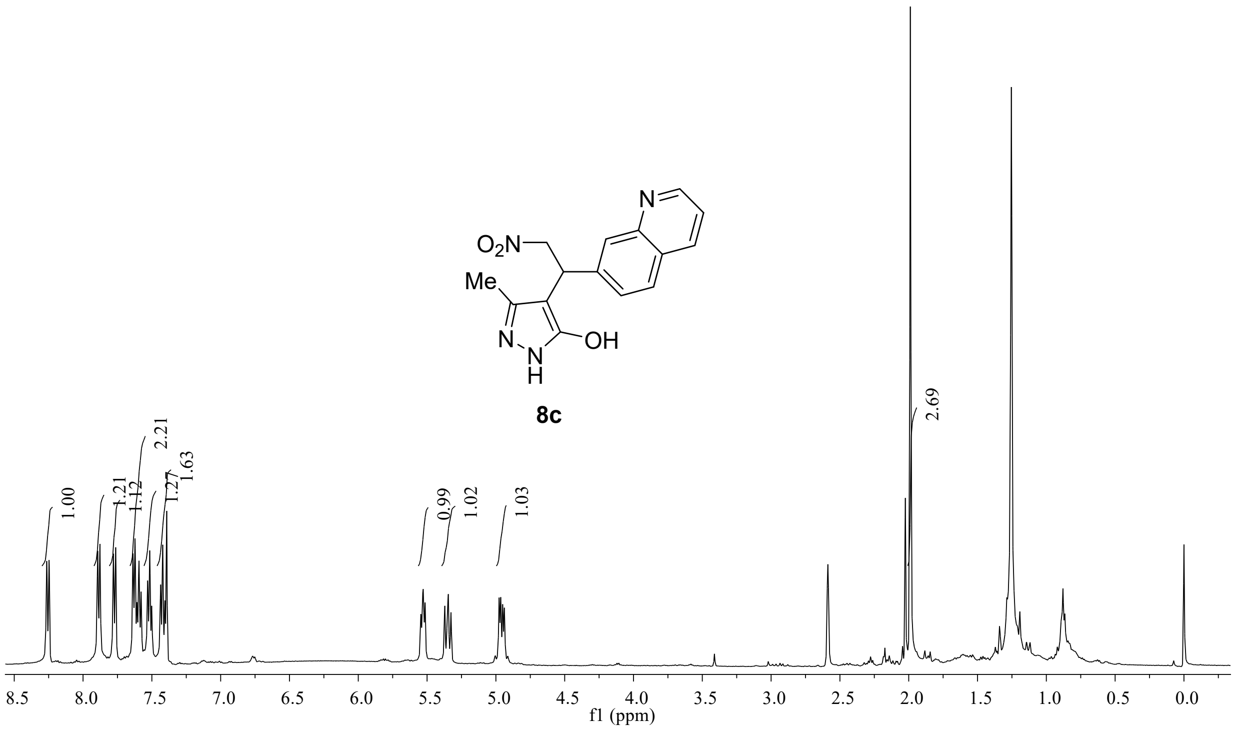

O2N
Me
N
N
H
OH
8c
1.00
1.21
1.12
2.21
1.27
1.63
0.99
1.02
1.03
2.69
f1 (ppm)

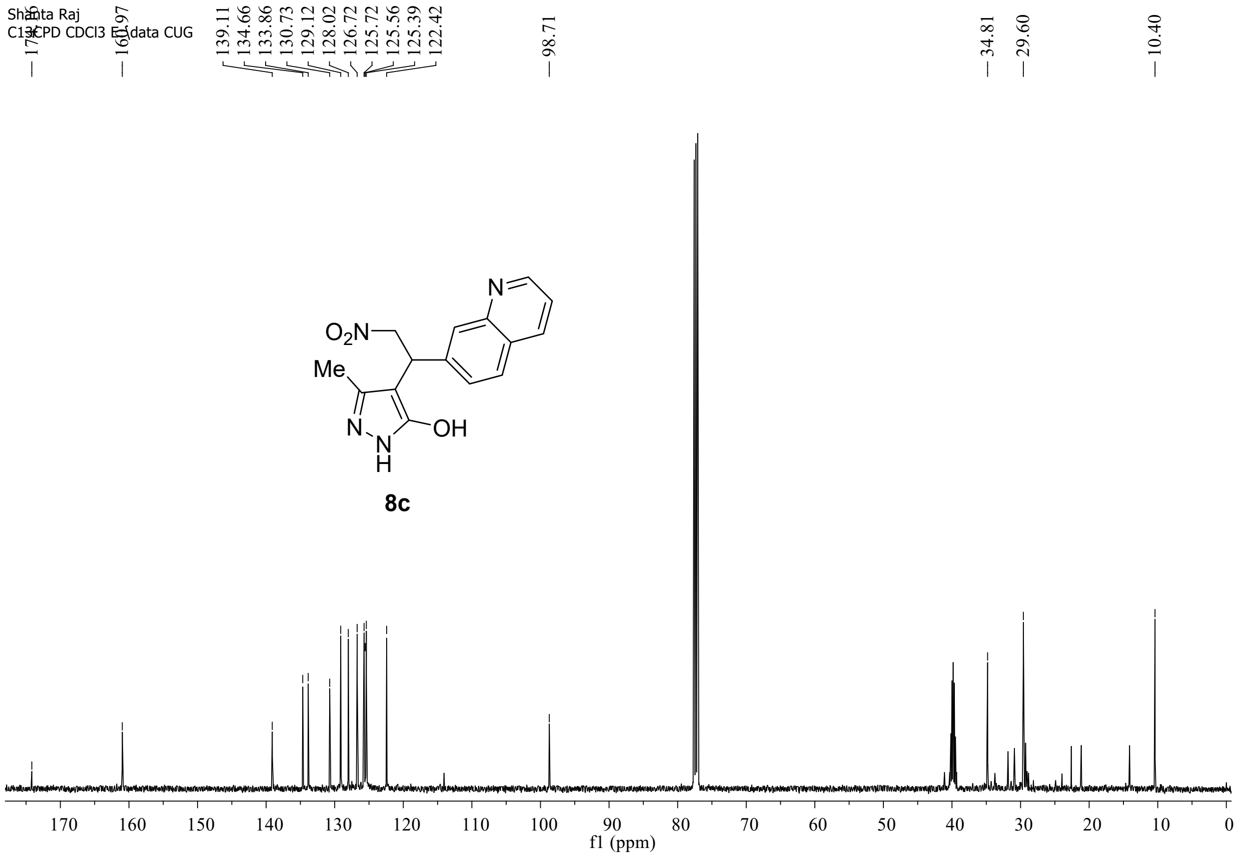

Shanta Raj
C13CPD CDCl3 E:\data CUG
160.97
139.11
134.66
133.86
130.73
129.12
128.02
126.72
125.72
125.56
125.39
122.42
98.71
34.81
29.60
10.40
O2N
Me
N
N
H
OH
8c
f1 (ppm)

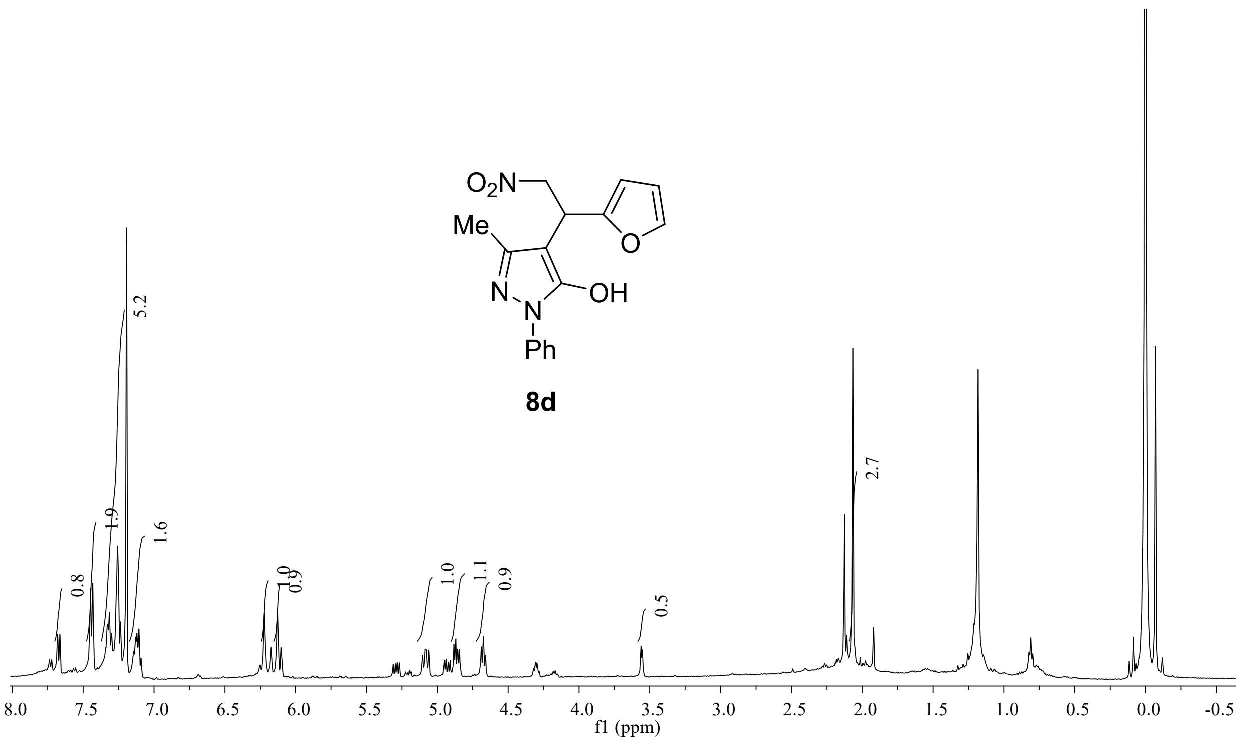

O2N
Me
O
OH
N
N
Ph
8d
5.2
2.7
1.9
1.6
0.8
1.0
0.9
1.0
1.1
0.9
0.5
8.0 7.5 7.0 6.5 6.0 5.5 5.0 4.5 4.0 3.5 3.0 2.5 2.0 1.5 1.0 0.5 0.0 -0.5
f1 (ppm)

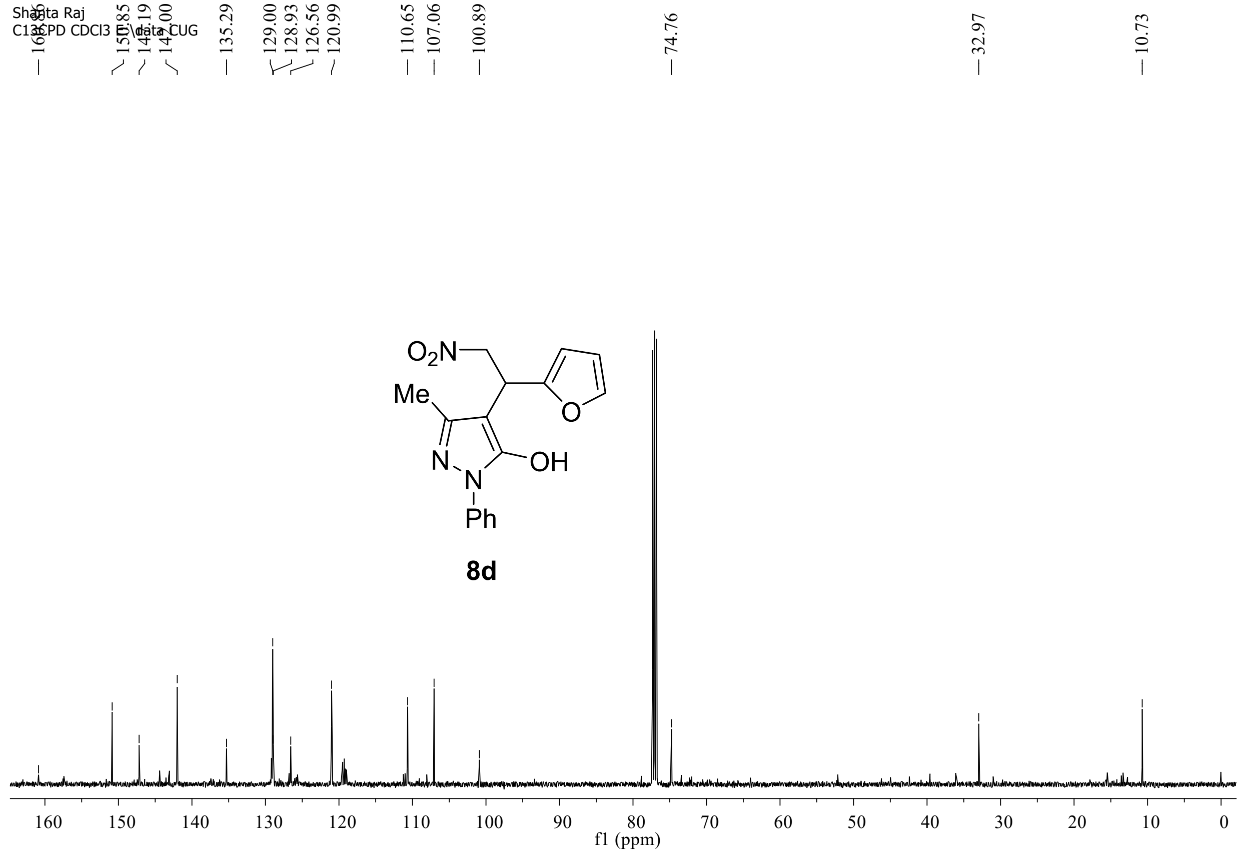

Shanta Raj
C13CPD CDCl3 E:\data CUG
150.85
144.19
142.00
135.29
129.00
128.93
126.56
120.99
110.65
107.06
100.89
74.76
32.97
10.73
O2N
Me
O
OH
N
N
Ph
8d
160 150 140 130 120 110 100 90 80 70 60 50 40 30 20 10 0
f1 (ppm)

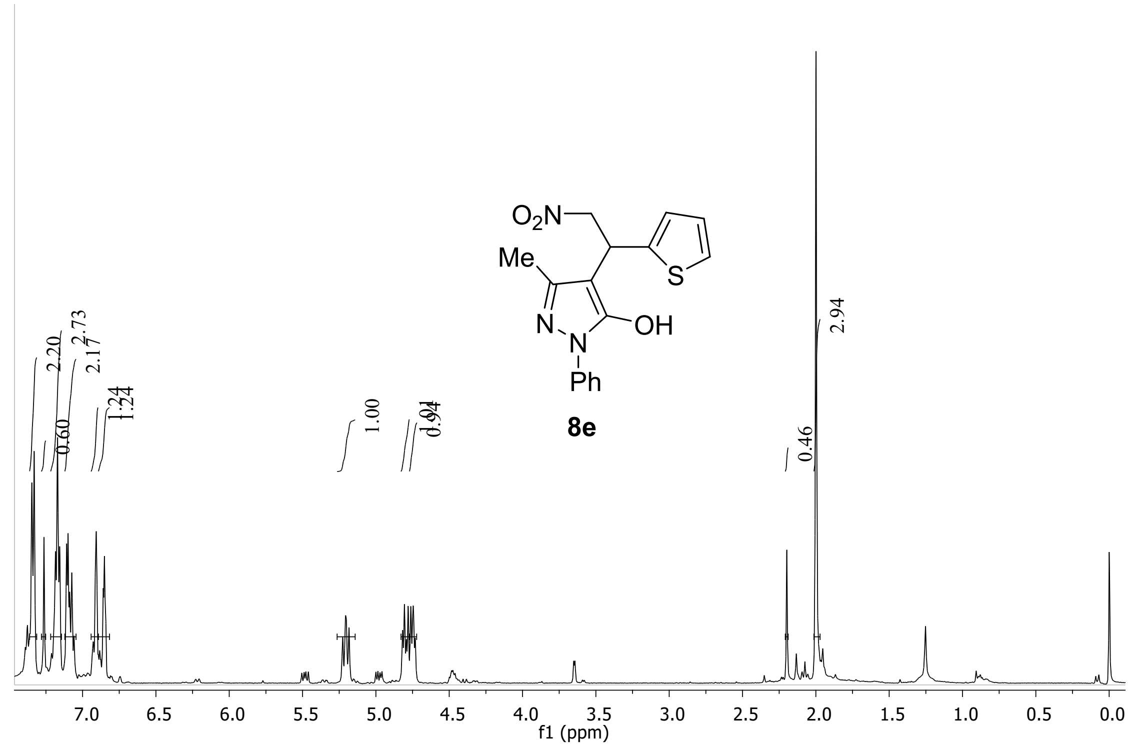

O_2N
Me
N
N
OH
S
Ph
8e
2.20
2.73
2.17
0.60
1.24
1.24
1.00
1.01
0.94
0.46
2.94
7.0 6.5 6.0 5.5 5.0 4.5 4.0 3.5 3.0 2.5 2.0 1.5 1.0 0.5 0.0
f1 (ppm)

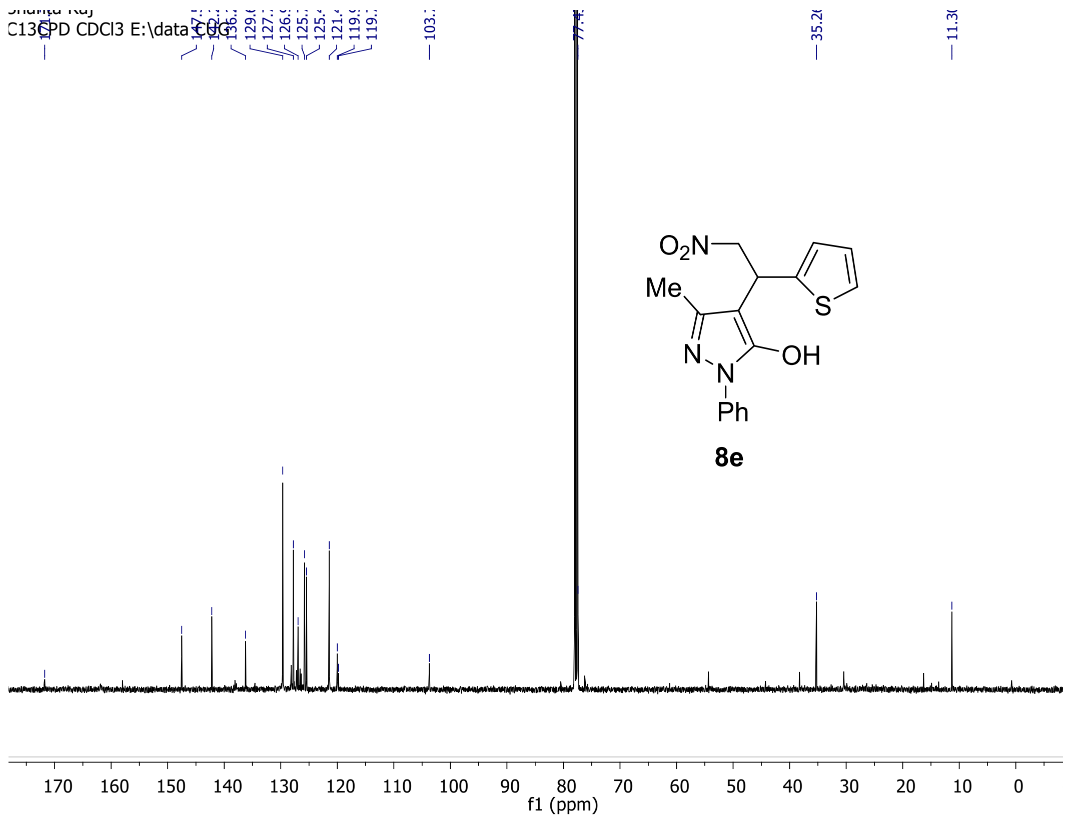

C13CPD CDCl3 E:\data
103.
35.2
11.3
O_2N
Me
N
N
OH
S
Ph
8e
170 160 150 140 130 120 110 100 90 80 70 60 50 40 30 20 10 0
f1 (ppm)

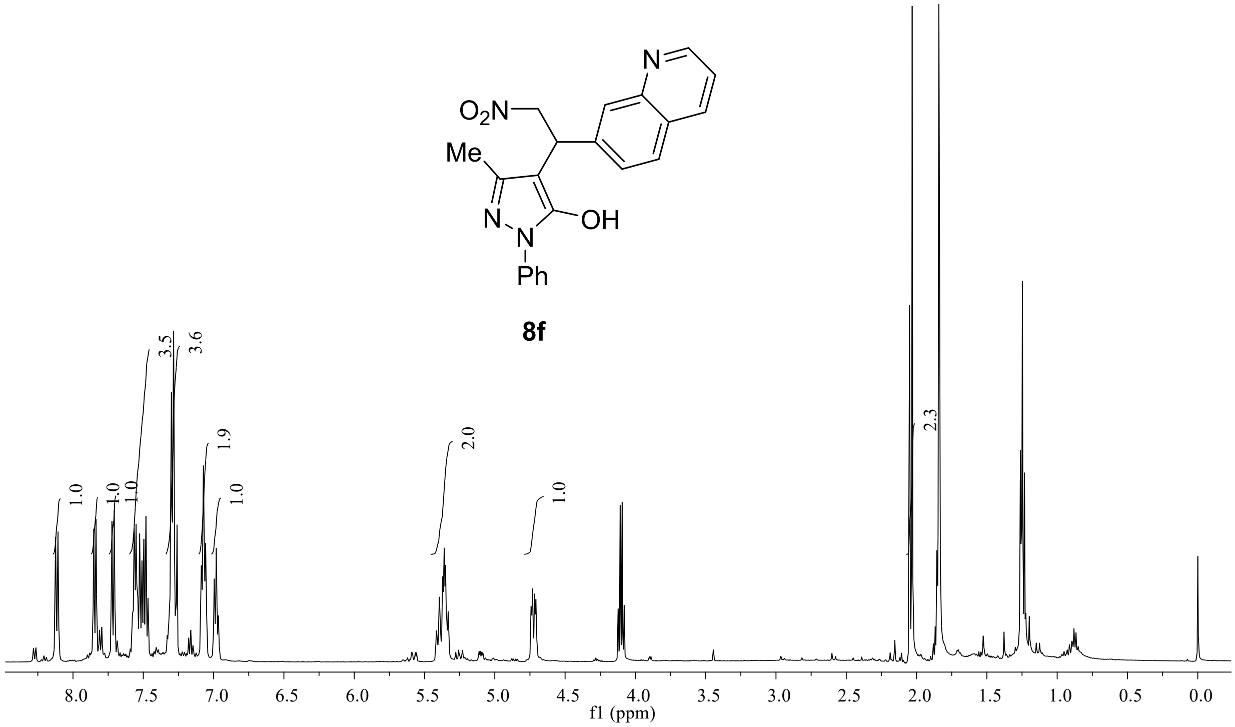
O2N
Me
N
N
OH
Ph
N
8f
1.0
1.0
1.0
3.5
3.6
1.9
1.0
2.0
1.0
2.3
8.0 7.5 7.0 6.5 6.0 5.5 5.0 4.5 4.0 3.5 3.0 2.5 2.0 1.5 1.0 0.5 0.0
f1 (ppm)

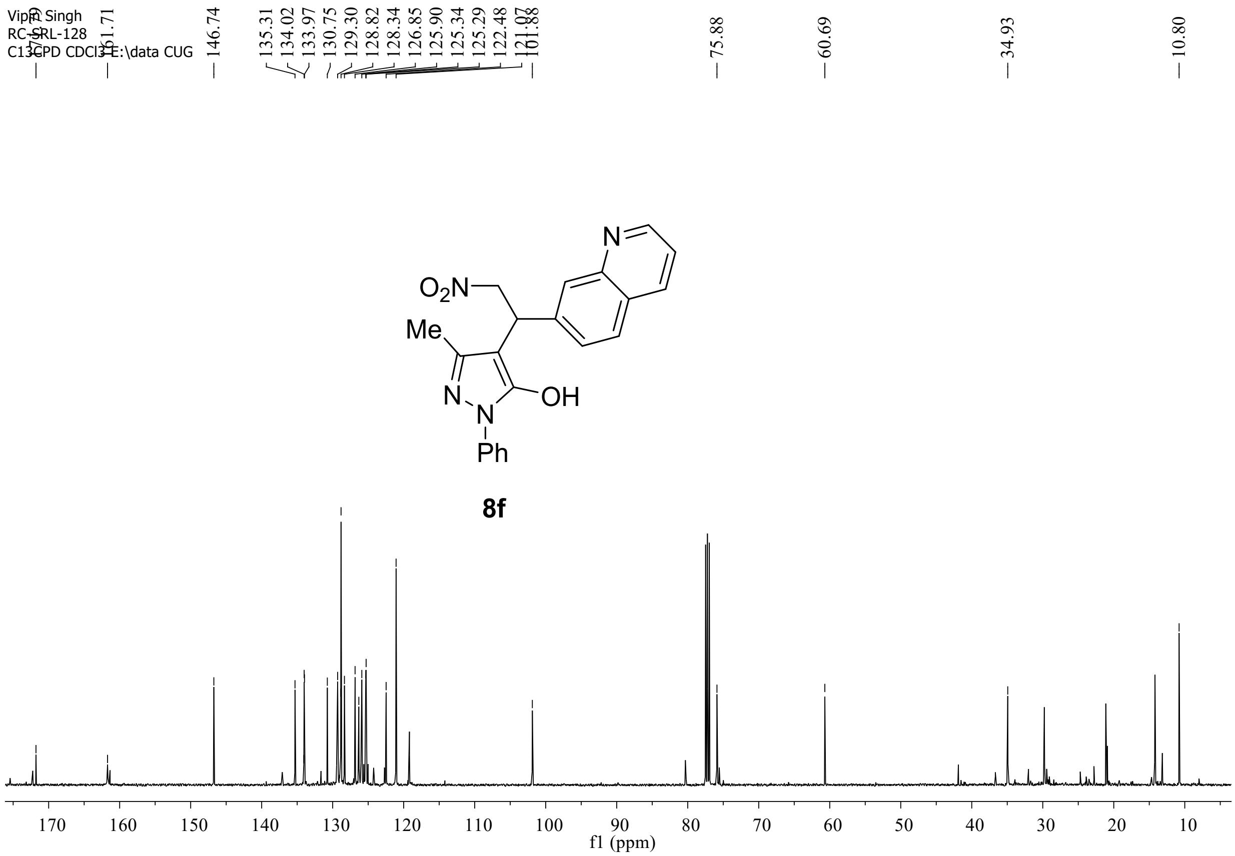
Vipin Singh
RC-SRL-128
C13CPD CDCl3 E:\data CUG
161.71
146.74
135.31
134.02
133.97
130.75
129.30
128.82
128.34
126.85
125.90
125.34
125.29
122.48
121.07
101.88
75.88
60.69
34.93
10.80
O2N
Me
N
N
OH
Ph
N
8f
170 160 150 140 130 120 110 100 90 80 70 60 50 40 30 20 10
f1 (ppm)

FT-IR Data of pyrazol-5-ol derivative (4, 4*a-k*, 6*a-l*, 8*a-d*)

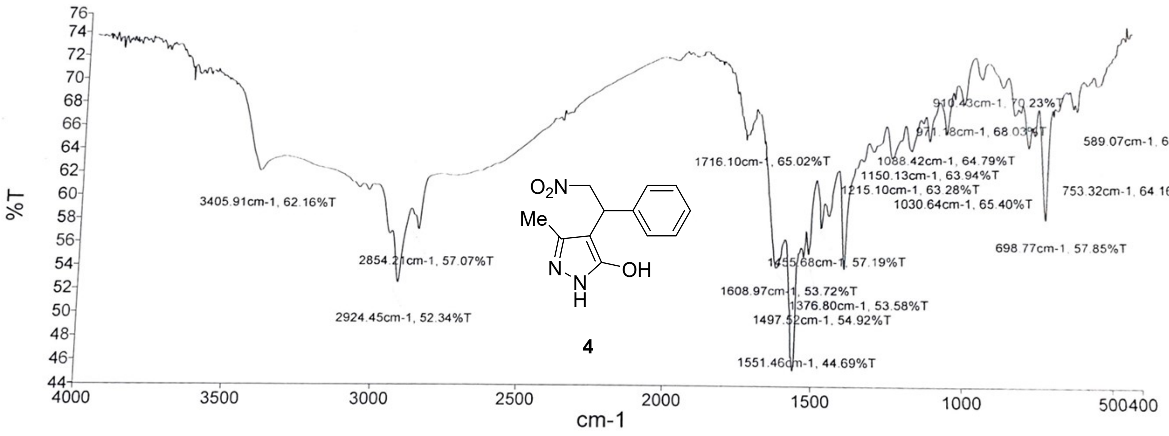

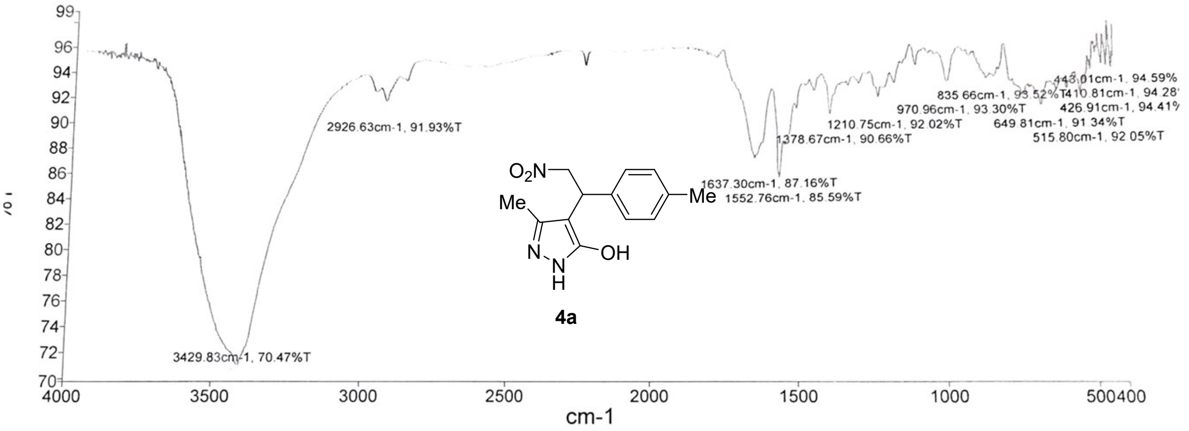

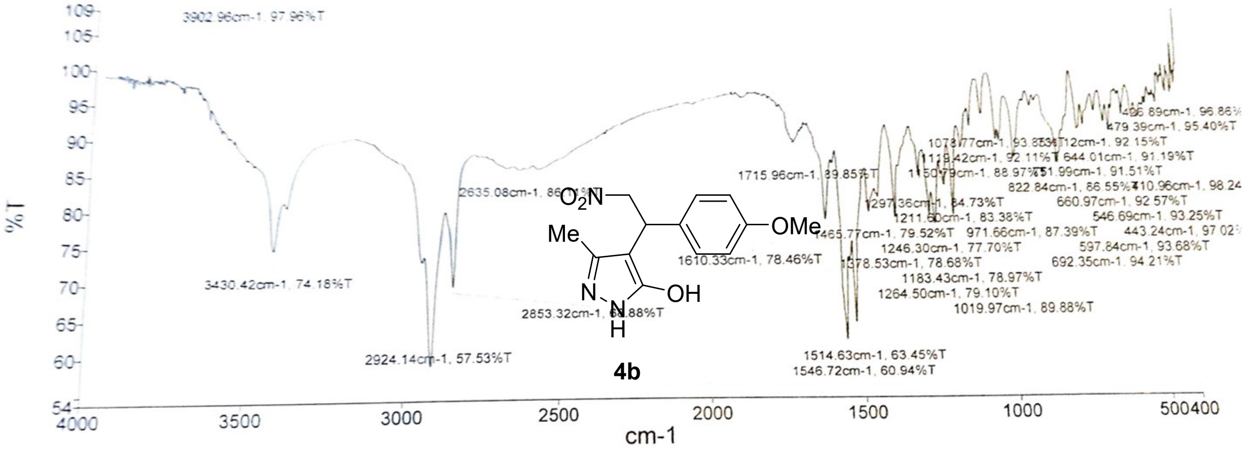

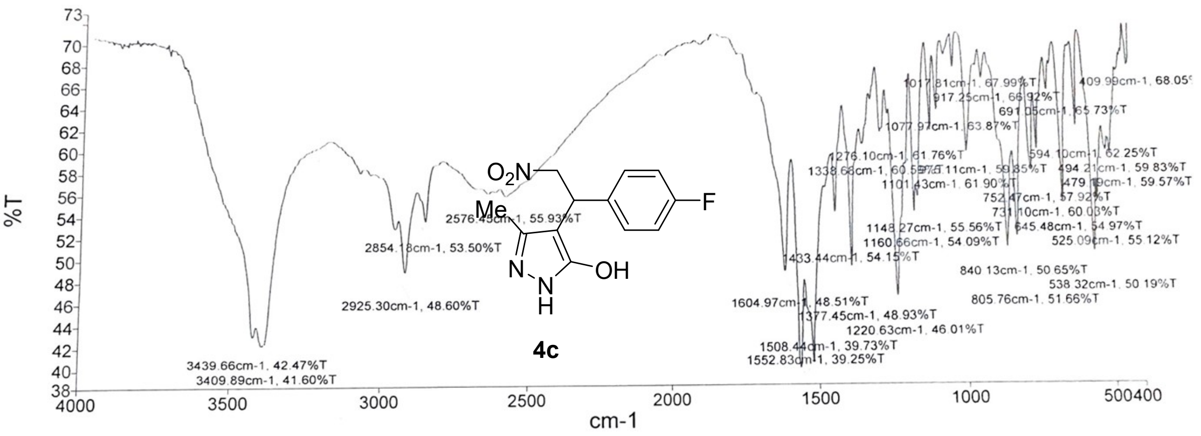

4c

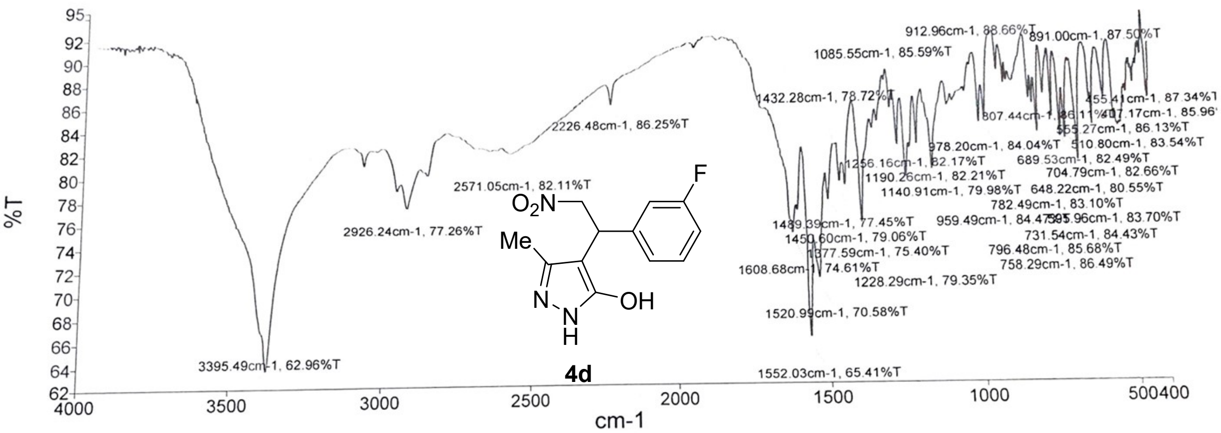

4d

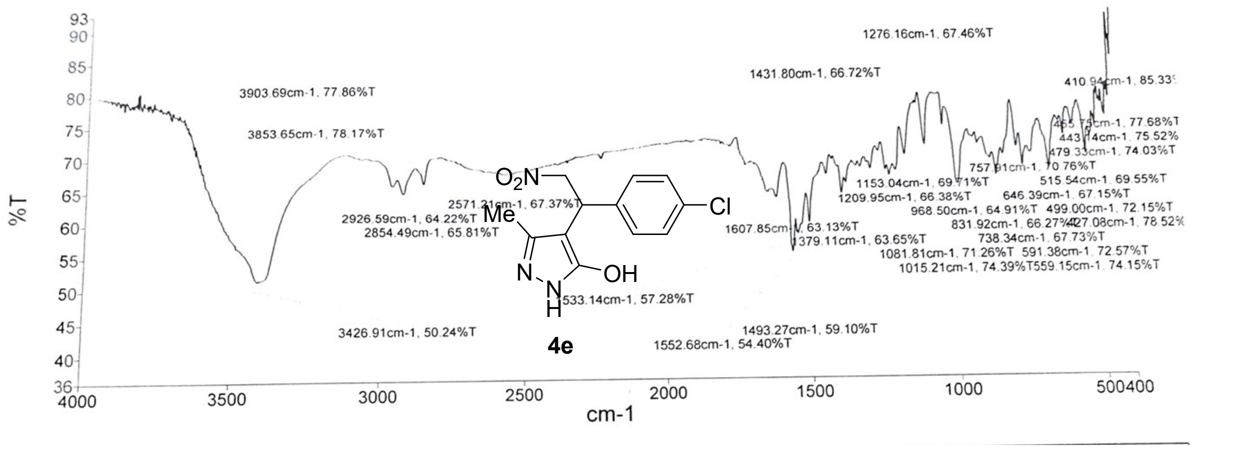

4e

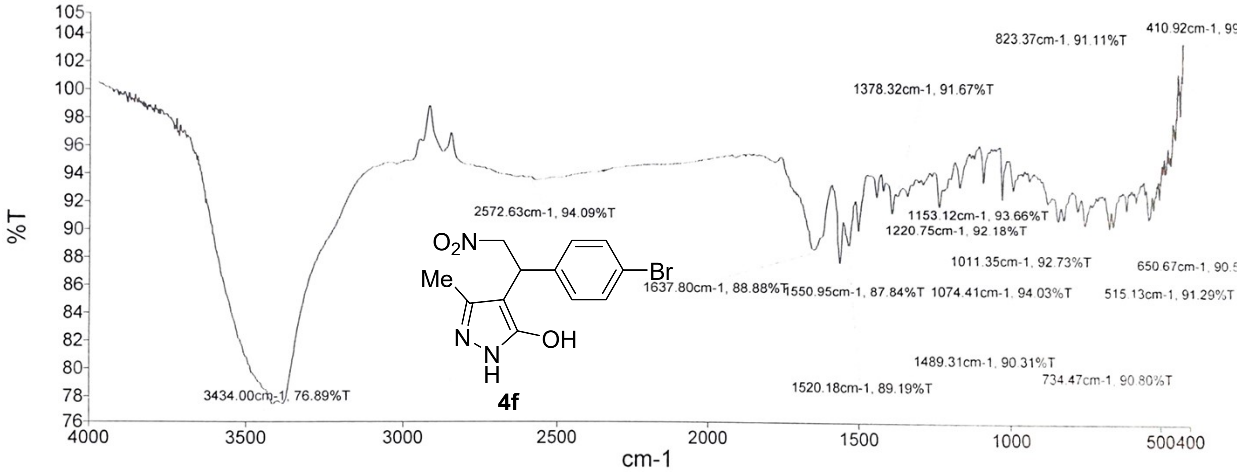
%T
cm-1
4f
O2N
Me
Br
N
N
H
OH
3434.00cm-1, 76.89%T
2572.63cm-1, 94.09%T
1637.80cm-1, 88.88%T
1550.95cm-1, 87.84%T
1520.18cm-1, 89.19%T
1489.31cm-1, 90.31%T
1378.32cm-1, 91.67%T
1220.75cm-1, 92.18%T
1153.12cm-1, 93.66%T
1074.41cm-1, 94.03%T
1011.35cm-1, 92.73%T
823.37cm-1, 91.11%T
734.47cm-1, 90.80%T
515.13cm-1, 91.29%T

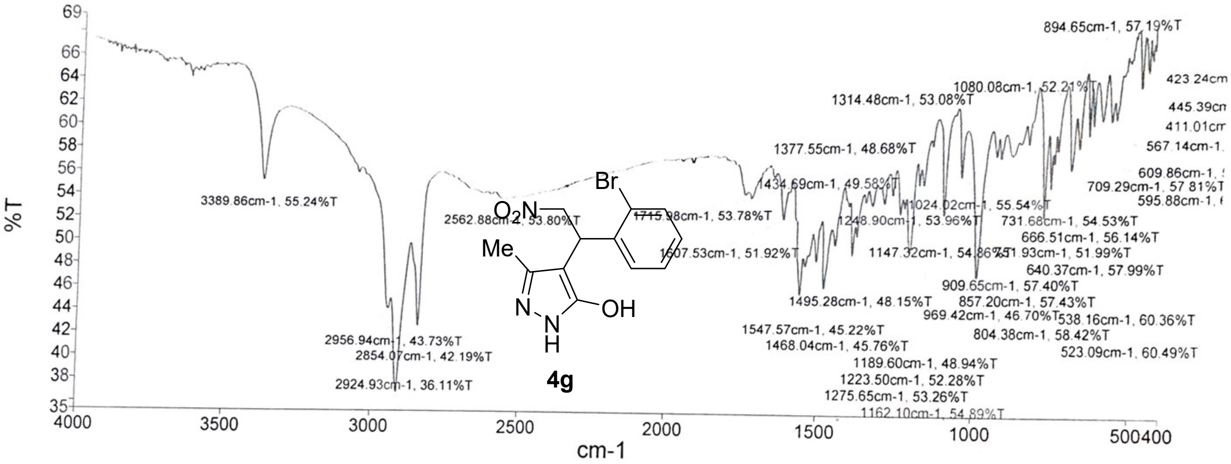
%T
cm-1
4g
O2N
Me
Br
N
N
H
OH
3389.86cm-1, 55.24%T
2956.94cm-1, 43.73%T
2854.07cm-1, 42.19%T
2924.93cm-1, 36.11%T
2562.88cm-1, 53.80%T
1715.98cm-1, 53.78%T
1607.53cm-1, 51.92%T
1547.57cm-1, 45.22%T
1495.28cm-1, 48.15%T
1468.04cm-1, 45.76%T
1377.55cm-1, 48.68%T
1314.48cm-1, 53.08%T
1275.65cm-1, 53.26%T
1223.50cm-1, 52.28%T
1189.60cm-1, 48.94%T
1080.08cm-1, 52.21%T
969.42cm-1, 46.70%T
909.65cm-1, 57.40%T
894.65cm-1, 57.19%T
857.20cm-1, 57.43%T
804.38cm-1, 58.42%T
731.68cm-1, 54.53%T
666.51cm-1, 56.14%T
640.37cm-1, 57.99%T
538.16cm-1, 60.36%T
523.09cm-1, 60.49%T

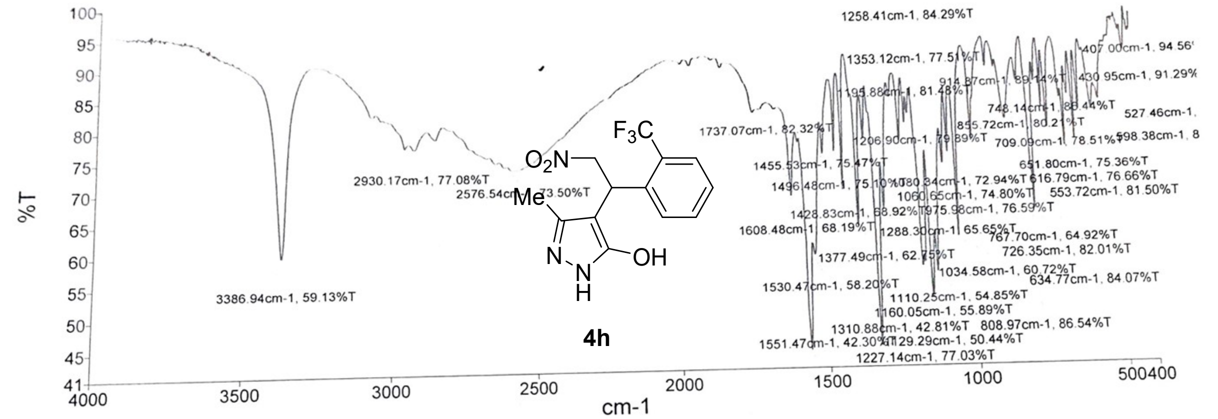
%T
cm-1
4h
O2N
F3C
Me
N
N
H
OH
3386.94cm-1, 59.13%T
2930.17cm-1, 77.08%T
1737.07cm-1, 82.32%T
1608.48cm-1, 68.19%T
1551.47cm-1, 42.30%T
1530.47cm-1, 58.20%T
1455.53cm-1, 75.47%T
1377.49cm-1, 62.75%T
1353.12cm-1, 77.51%T
1310.88cm-1, 42.81%T
1258.41cm-1, 84.29%T
1227.14cm-1, 77.03%T
1160.05cm-1, 55.89%T
1110.25cm-1, 54.85%T
1034.58cm-1, 60.72%T
808.97cm-1, 86.54%T
767.70cm-1, 64.92%T
726.35cm-1, 82.01%T
634.77cm-1, 84.07%T
553.72cm-1, 81.50%T

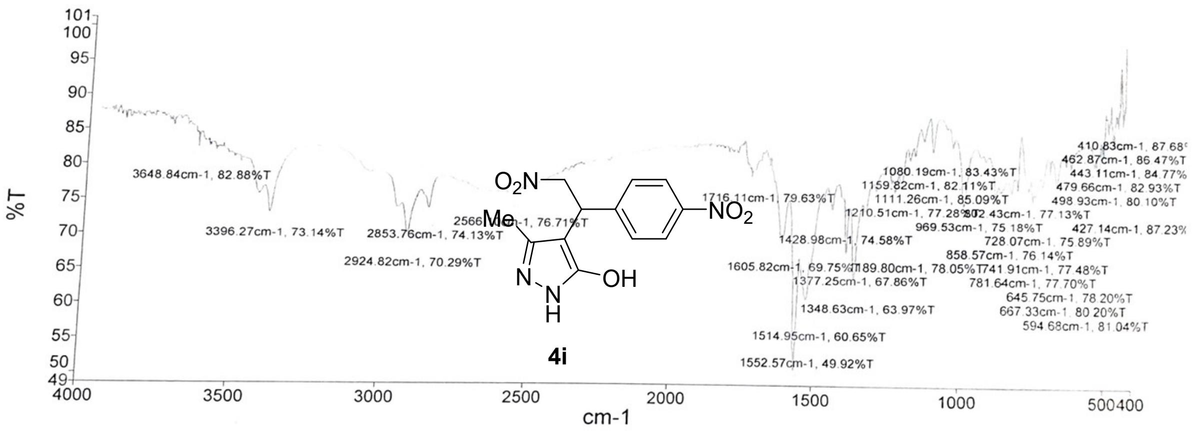

4i

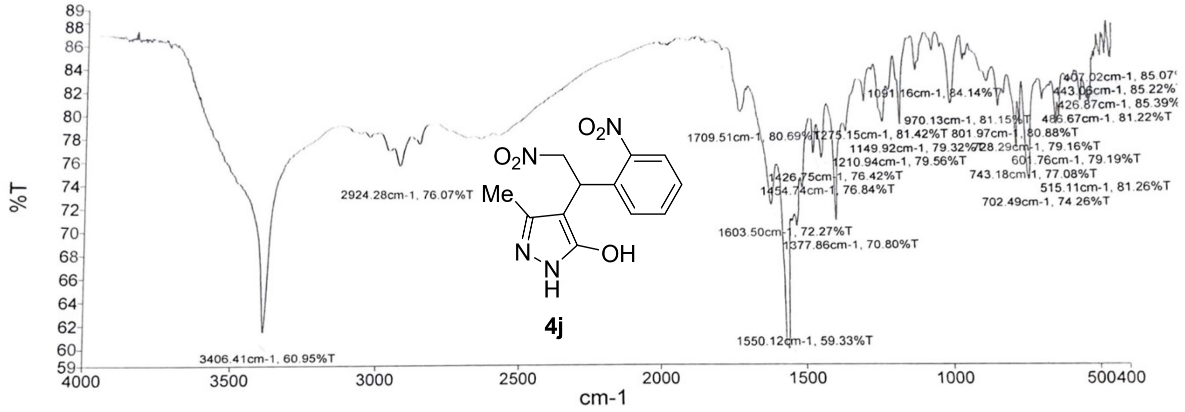

4j

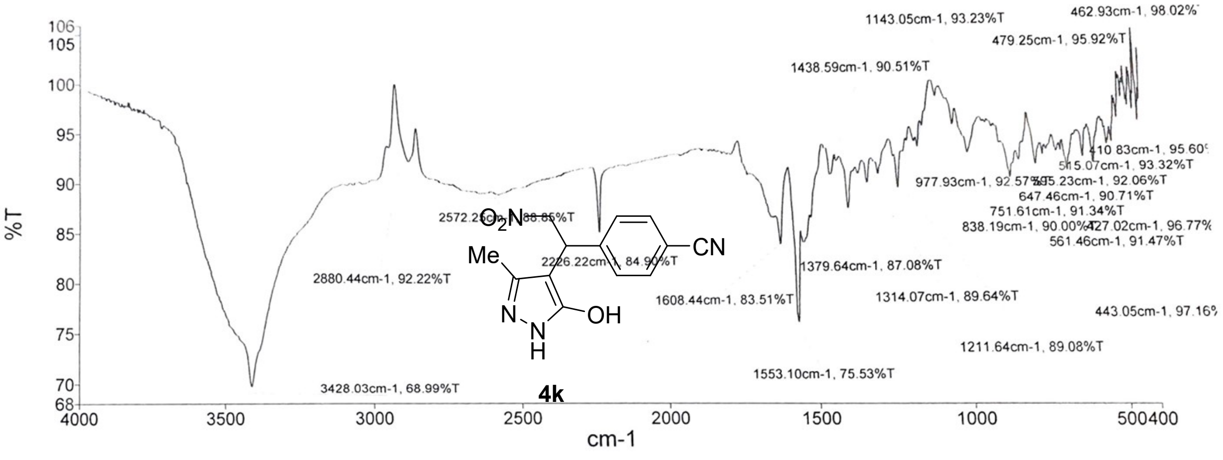

4k

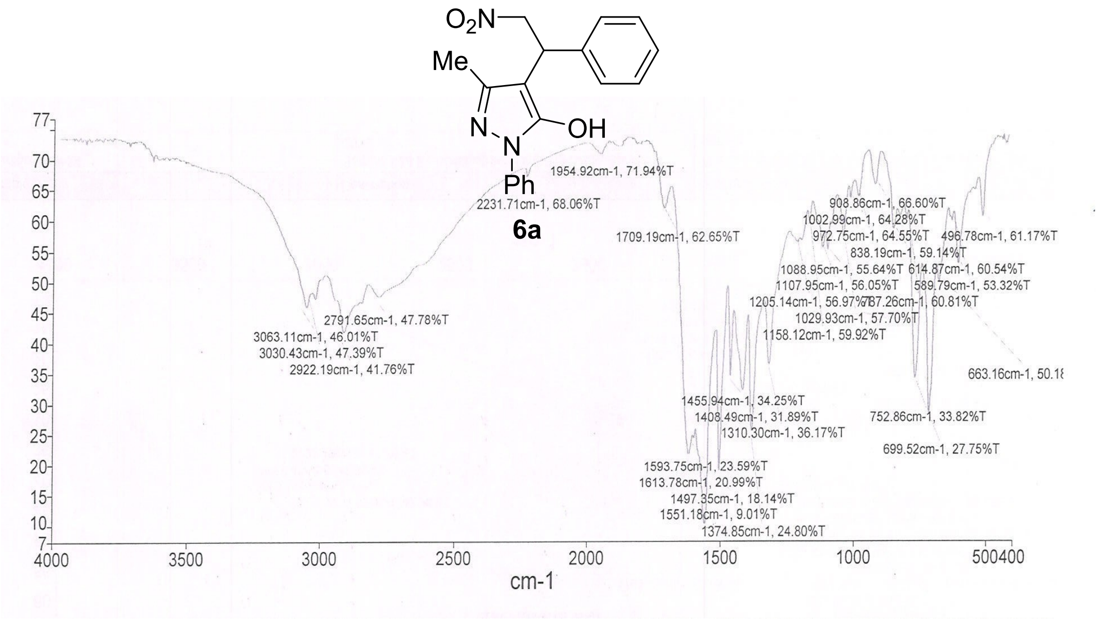

O2N
Me
N
N
OH
Ph
6a
1954.92cm-1, 71.94%T
2231.71cm-1, 68.06%T
1709.19cm-1, 62.65%T
2791.65cm-1, 47.78%T
3063.11cm-1, 46.01%T
3030.43cm-1, 47.39%T
2922.19cm-1, 41.76%T
908.86cm-1, 66.60%T
1002.99cm-1, 64.28%T
972.75cm-1, 64.55%T
496.78cm-1, 61.17%T
838.19cm-1, 59.14%T
1088.95cm-1, 55.64%T
614.87cm-1, 60.54%T
1107.95cm-1, 56.05%T
589.79cm-1, 53.32%T
1205.14cm-1, 56.97%T
767.26cm-1, 60.81%T
1029.93cm-1, 57.70%T
1158.12cm-1, 59.92%T
663.16cm-1, 50.18
1455.94cm-1, 34.25%T
1408.49cm-1, 31.89%T
1310.30cm-1, 36.17%T
752.86cm-1, 33.82%T
699.52cm-1, 27.75%T
1593.75cm-1, 23.59%T
1613.78cm-1, 20.99%T
1497.35cm-1, 18.14%T
1551.18cm-1, 9.01%T
1374.85cm-1, 24.80%T
77
70
65
60
55
50
45
40
35
30
25
20
15
10
7
4000
3500
3000
2500
2000
1500
1000
500400
cm-1

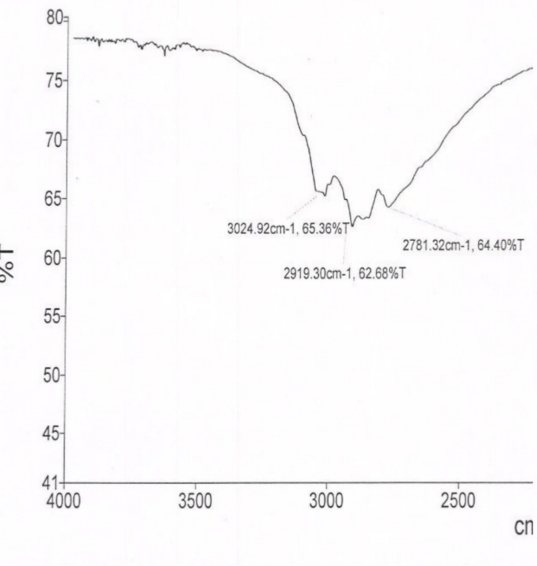
%T
3024.92cm-1, 65.36%T
2919.30cm-1, 62.68%T
2781.32cm-1, 64.40%T
4000
3500
3000
2500

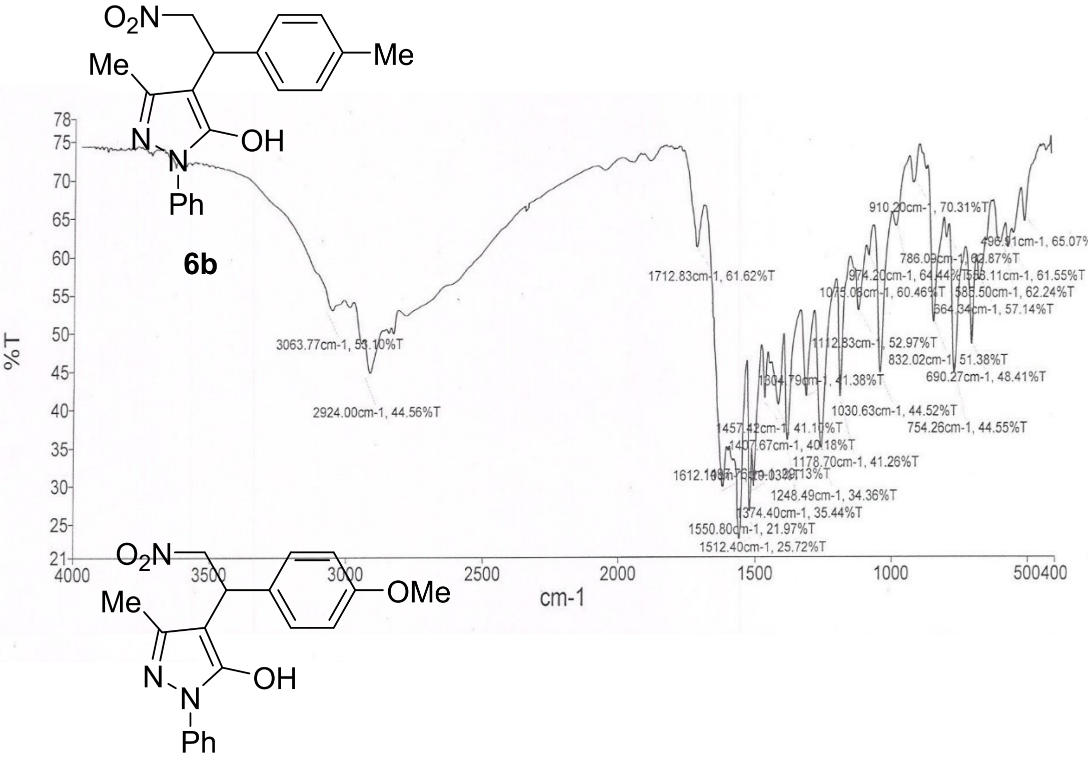
O2N
Me
Me
N
N
OH
Ph
%T
cm-1
3063.77cm-1, 53.10%T
2924.00cm-1, 44.56%T
1712.83cm-1, 61.62%T
910.20cm-1, 70.31%T
496.91cm-1, 65.07%
1075.06cm-1, 60.46%T
1112.83cm-1, 52.97%T
832.02cm-1, 51.38%T
690.27cm-1, 48.41%T
564.34cm-1, 57.14%T
1030.63cm-1, 44.52%T
754.26cm-1, 44.55%T
1178.70cm-1, 41.26%T
1248.49cm-1, 34.36%T
1374.40cm-1, 35.44%T
1550.80cm-1, 21.97%T
1512.40cm-1, 25.72%T
OMe

6b

6c

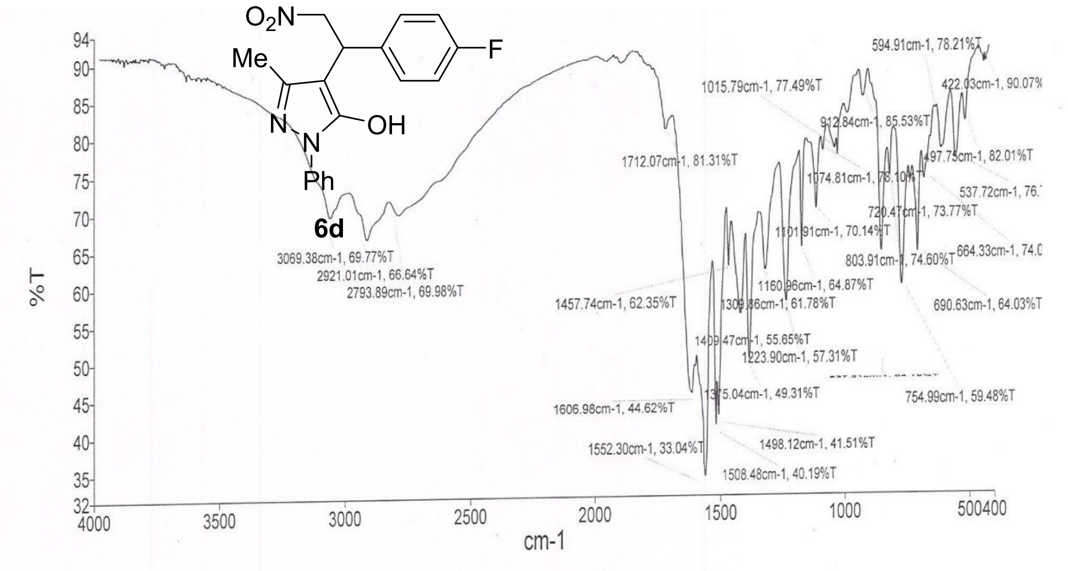
O2N
Me
N
N
OH
Ph
F
6d
%T
cm-1
3069.38cm-1, 69.77%T
2921.01cm-1, 66.64%T
2793.89cm-1, 69.98%T
1712.07cm-1, 81.31%T
1457.74cm-1, 62.35%T
1606.98cm-1, 44.62%T
1552.30cm-1, 33.04%T
1508.48cm-1, 40.19%T
1498.12cm-1, 41.51%T
1375.04cm-1, 49.31%T
1223.90cm-1, 57.31%T
1160.96cm-1, 64.87%T
1015.79cm-1, 77.49%T
912.84cm-1, 85.53%T
803.91cm-1, 74.60%T
754.99cm-1, 59.48%T
690.63cm-1, 64.03%T
594.91cm-1, 78.21%T
422.03cm-1, 90.07%
497.75cm-1, 82.01%T

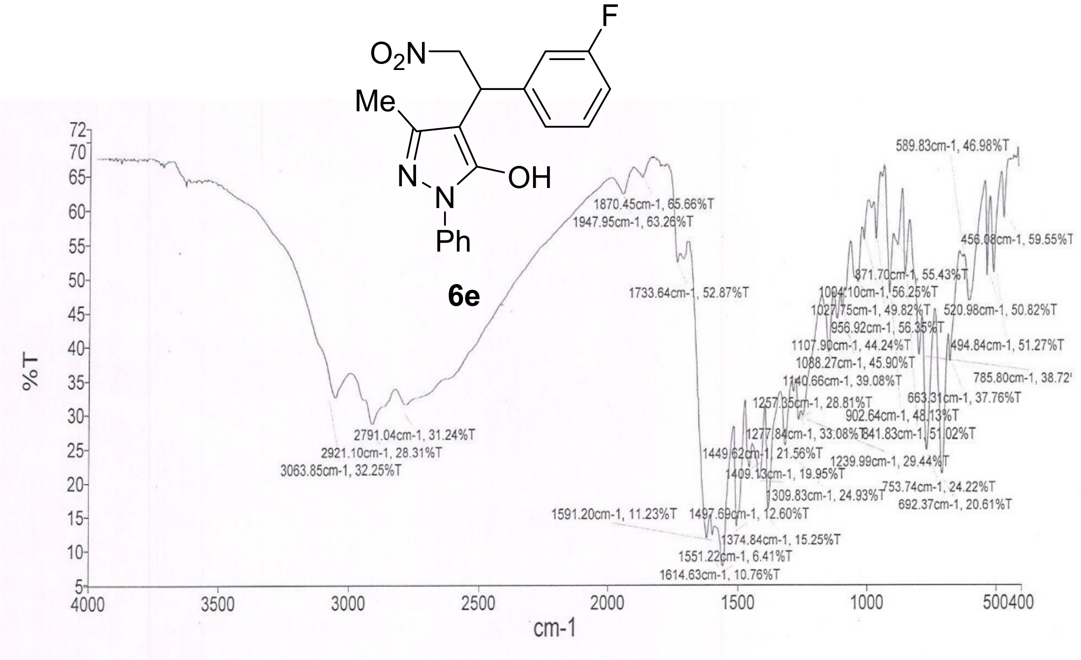
F
O_2N
Me
N
N
OH
Ph
72
70
65
60
55
50
45
40
35
30
25
20
15
10
5
%T
4000
3500
3000
2500
2000
1500
1000
500400
cm-1
589.83cm-1, 46.98%T
1870.45cm-1, 65.66%T
1947.95cm-1, 63.26%T
456.08cm-1, 59.55%T
871.70cm-1, 55.43%T
1733.64cm-1, 52.87%T
1004.10cm-1, 56.25%T
1027.75cm-1, 49.82%T
520.98cm-1, 50.82%T
956.92cm-1, 56.35%T
1107.90cm-1, 44.24%T
494.84cm-1, 51.27%T
1088.27cm-1, 45.90%T
785.80cm-1, 38.72
1140.66cm-1, 39.08%T
663.31cm-1, 37.76%T
1257.35cm-1, 28.81%T
902.64cm-1, 48.13%T
2791.04cm-1, 31.24%T
1277.84cm-1, 33.08%T
841.83cm-1, 51.02%T
2921.10cm-1, 28.31%T
1449.62cm-1, 21.56%T
1239.99cm-1, 29.44%T
3063.85cm-1, 32.25%T
1409.13cm-1, 19.95%T
753.74cm-1, 24.22%T
1309.83cm-1, 24.93%T
692.37cm-1, 20.61%T
1591.20cm-1, 11.23%T
1497.69cm-1, 12.60%T
1374.84cm-1, 15.25%T
1551.22cm-1, 6.41%T
1614.63cm-1, 10.76%T

6e

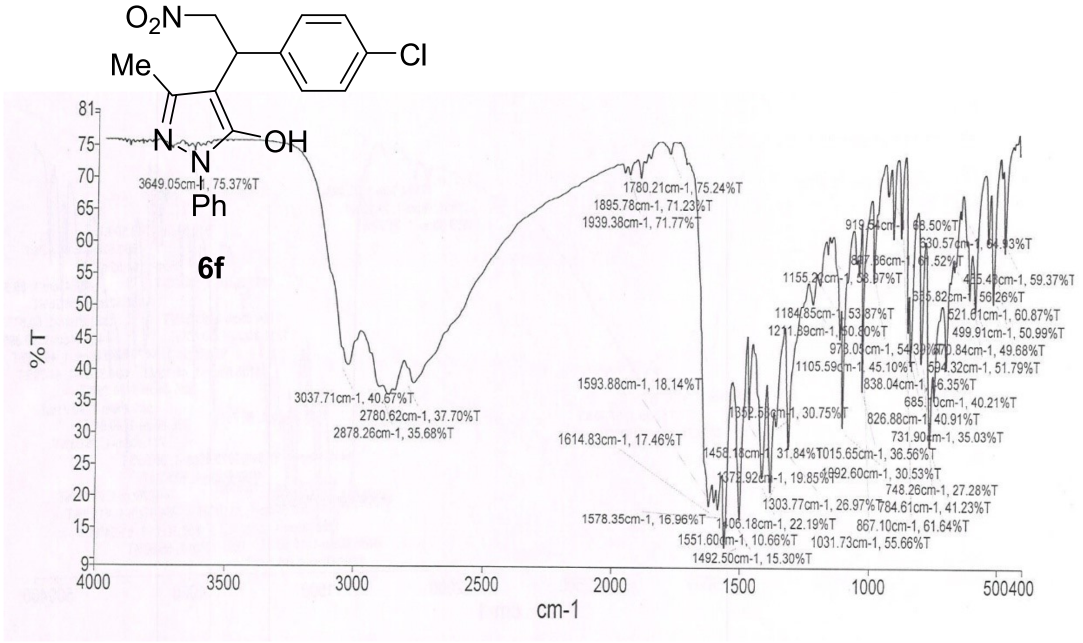

O2N
Me
Cl
N
N
OH
Ph
%T
cm-1
3649.05cm-1, 75.37%T
3037.71cm-1, 40.67%T
2780.62cm-1, 37.70%T
2878.26cm-1, 35.68%T
1780.21cm-1, 75.24%T
1895.78cm-1, 71.23%T
1939.38cm-1, 71.77%T
1593.88cm-1, 18.14%T
1614.83cm-1, 17.46%T
1578.35cm-1, 16.96%T
1551.60cm-1, 10.66%T
1492.50cm-1, 15.30%T
1303.77cm-1, 26.97%T
1031.73cm-1, 55.66%T
867.10cm-1, 61.64%T
748.26cm-1, 27.28%T
731.90cm-1, 35.03%T
499.91cm-1, 50.99%T

6f

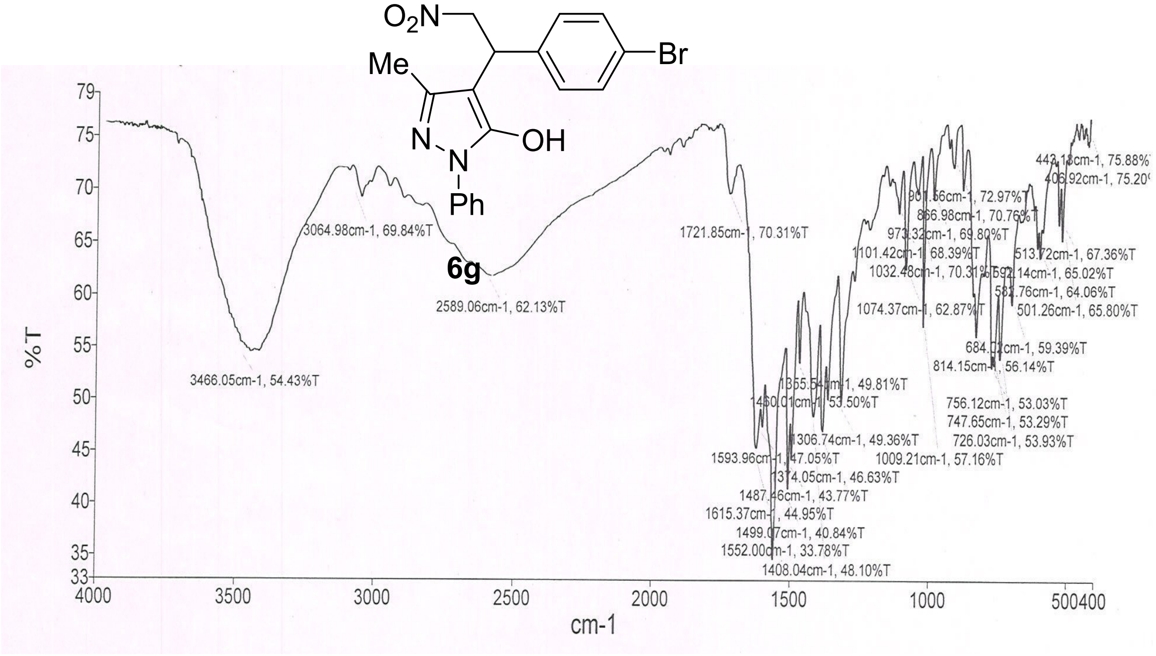

O_2N
Me
Br
N
N
OH
Ph
6g
%T
cm-1
3466.05cm-1, 54.43%T
3064.98cm-1, 69.84%T
2589.06cm-1, 62.13%T
1721.85cm-1, 70.31%T
1306.74cm-1, 49.36%T
1009.21cm-1, 57.16%T
1074.37cm-1, 62.87%T
756.12cm-1, 53.03%T
747.65cm-1, 53.29%T
726.03cm-1, 53.93%T
501.26cm-1, 65.80%T

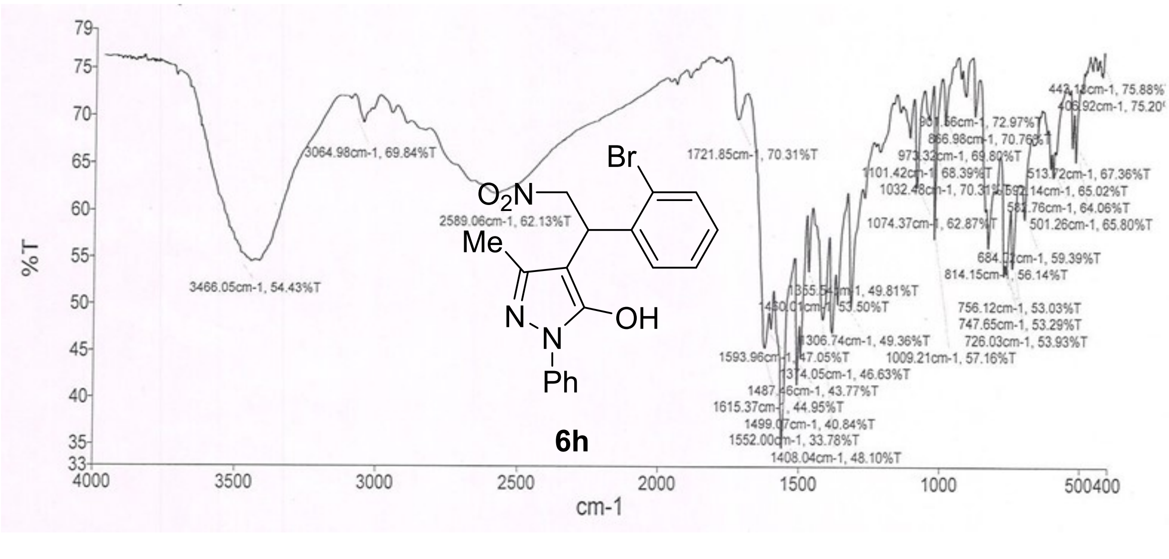

Br
O_2N
Me
N
N
OH
Ph
6h
%T
cm-1
3466.05cm-1, 54.43%T
3064.98cm-1, 69.84%T
2589.06cm-1, 62.13%T
1721.85cm-1, 70.31%T
1306.74cm-1, 49.36%T
1009.21cm-1, 57.16%T
1074.37cm-1, 62.87%T
756.12cm-1, 53.03%T
747.65cm-1, 53.29%T
726.03cm-1, 53.93%T
501.26cm-1, 65.80%T

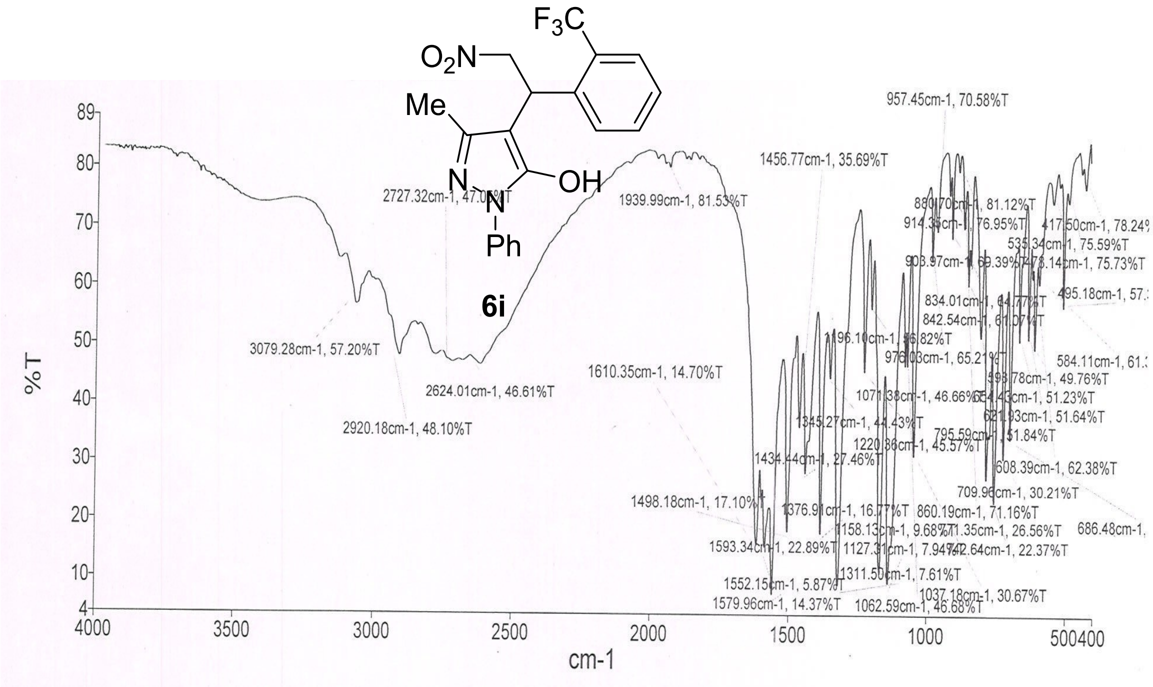

F_3C
O_2N
Me
N
N
OH
Ph
6i
%T
cm-1
957.45cm-1, 70.58%T
1456.77cm-1, 35.69%T
1939.99cm-1, 81.53%T
3079.28cm-1, 57.20%T
2624.01cm-1, 46.61%T
2920.18cm-1, 48.10%T
1610.35cm-1, 14.70%T
1498.18cm-1, 17.10%T
1552.15cm-1, 5.87%T
1062.59cm-1, 46.68%T
1311.50cm-1, 7.61%T
1037.18cm-1, 30.67%T
709.96cm-1, 30.21%T
860.19cm-1, 71.16%T
608.39cm-1, 62.38%T
842.54cm-1, 61.07%T

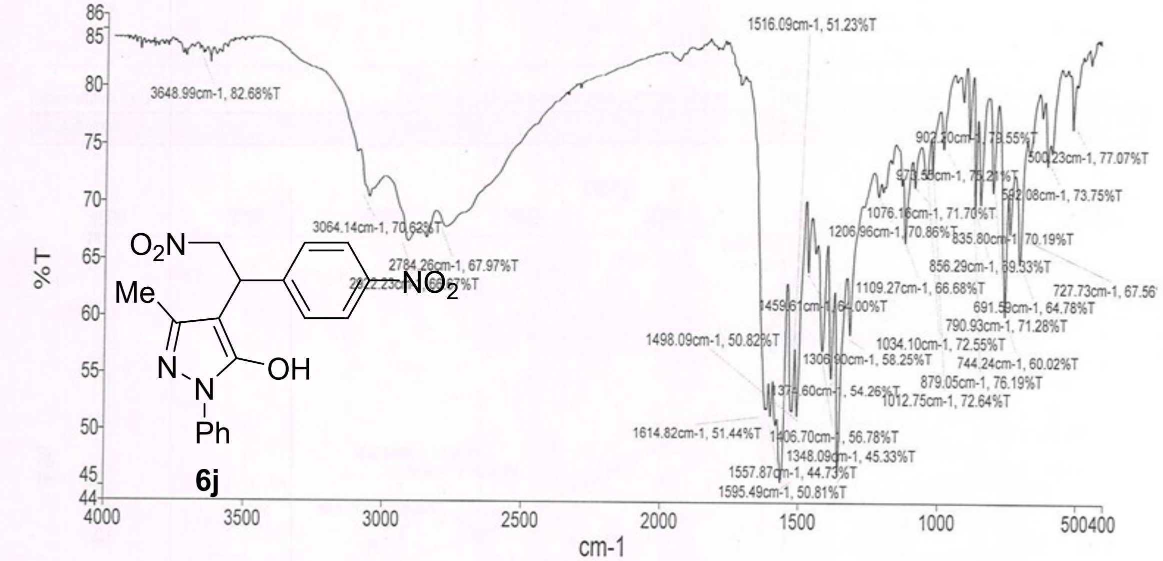

%T
cm-1
3648.99cm-1, 82.68%T
3064.14cm-1, 70.62%T
2784.26cm-1, 67.97%T
1516.09cm-1, 51.23%T
1498.09cm-1, 50.82%T
1614.82cm-1, 51.44%T
1557.87cm-1, 44.73%T
1595.49cm-1, 50.81%T
1406.70cm-1, 56.78%T
1348.09cm-1, 45.33%T
1206.96cm-1, 70.86%T
1076.16cm-1, 71.70%T
1109.27cm-1, 66.68%T
1034.10cm-1, 72.55%T
1012.75cm-1, 72.64%T
902.20cm-1, 78.55%T
856.29cm-1, 69.33%T
835.80cm-1, 70.19%T
879.05cm-1, 76.19%T
790.93cm-1, 71.28%T
744.24cm-1, 60.02%T
691.58cm-1, 64.78%T
727.73cm-1, 67.56
500.23cm-1, 77.07%T
O2N
Me
N
N
OH
Ph
NO2

6j

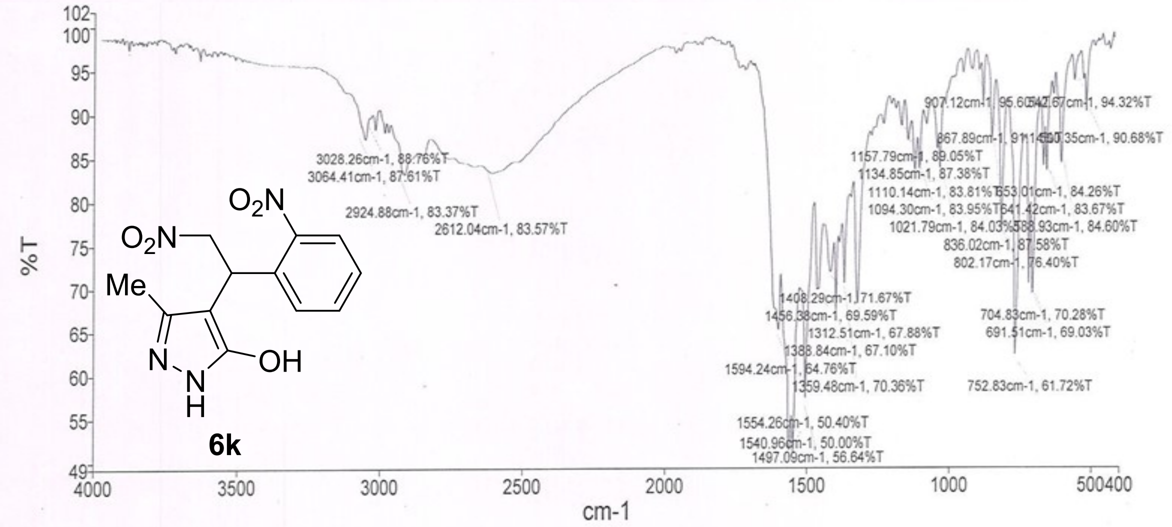

%T
cm-1
3028.26cm-1, 88.76%T
3064.41cm-1, 87.61%T
2924.88cm-1, 83.37%T
2612.04cm-1, 83.57%T
1157.79cm-1, 89.05%T
1134.85cm-1, 87.38%T
1110.14cm-1, 83.81%T
1094.30cm-1, 83.95%T
1021.79cm-1, 84.03%T
907.12cm-1, 95.60%T
867.89cm-1, 91.14%T
836.02cm-1, 87.58%T
802.17cm-1, 76.40%T
1408.29cm-1, 71.67%T
1456.38cm-1, 69.59%T
1312.51cm-1, 67.88%T
1388.84cm-1, 67.10%T
1594.24cm-1, 64.76%T
1359.48cm-1, 70.36%T
1554.26cm-1, 50.40%T
1540.96cm-1, 50.00%T
1497.09cm-1, 56.64%T
704.83cm-1, 70.28%T
691.51cm-1, 69.03%T
752.83cm-1, 61.72%T
O2N
Me
N
N
H
OH

6k

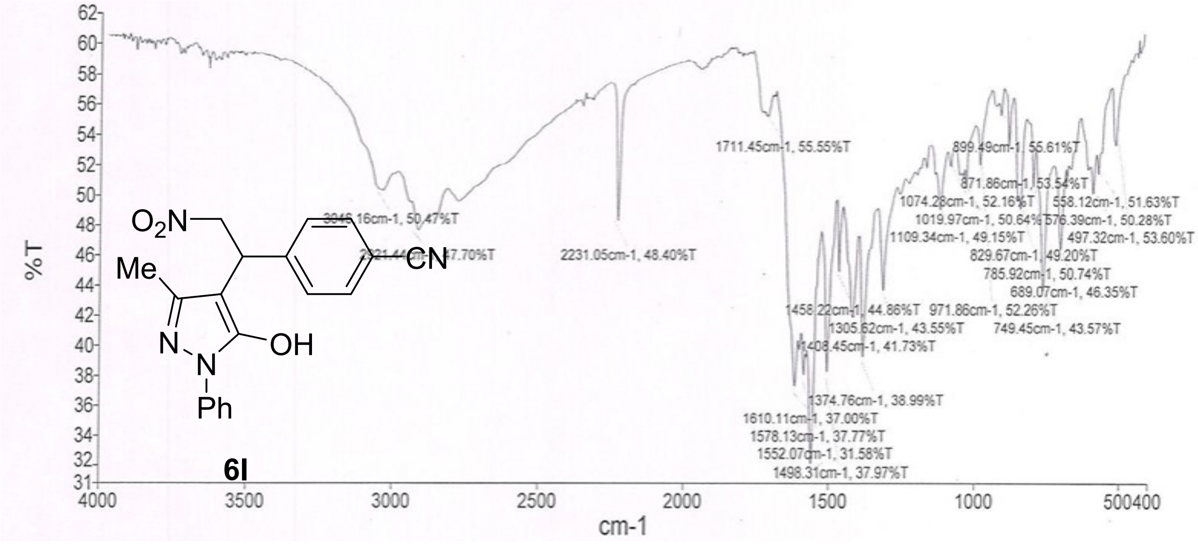

%T
cm-1
2231.05cm-1, 48.40%T
1711.45cm-1, 55.55%T
1074.28cm-1, 52.16%T
1019.97cm-1, 50.64%T
1109.34cm-1, 49.15%T
899.49cm-1, 55.61%T
871.86cm-1, 53.54%T
829.67cm-1, 49.20%T
785.92cm-1, 50.74%T
689.07cm-1, 46.35%T
558.12cm-1, 51.63%T
576.39cm-1, 50.28%T
497.32cm-1, 53.60%T
1458.22cm-1, 44.86%T
971.86cm-1, 52.26%T
1305.62cm-1, 43.55%T
749.45cm-1, 43.57%T
1408.45cm-1, 41.73%T
1374.76cm-1, 38.99%T
1610.11cm-1, 37.00%T
1578.13cm-1, 37.77%T
1552.07cm-1, 31.58%T
1498.31cm-1, 37.97%T
O2N
Me
N
N
OH
Ph
CN

6l

PerkinElmer Spectrum Version 10.00.00
09 April 2018 21:33:16

Analyst Administrator
Date 09 April 2018 21:33:16

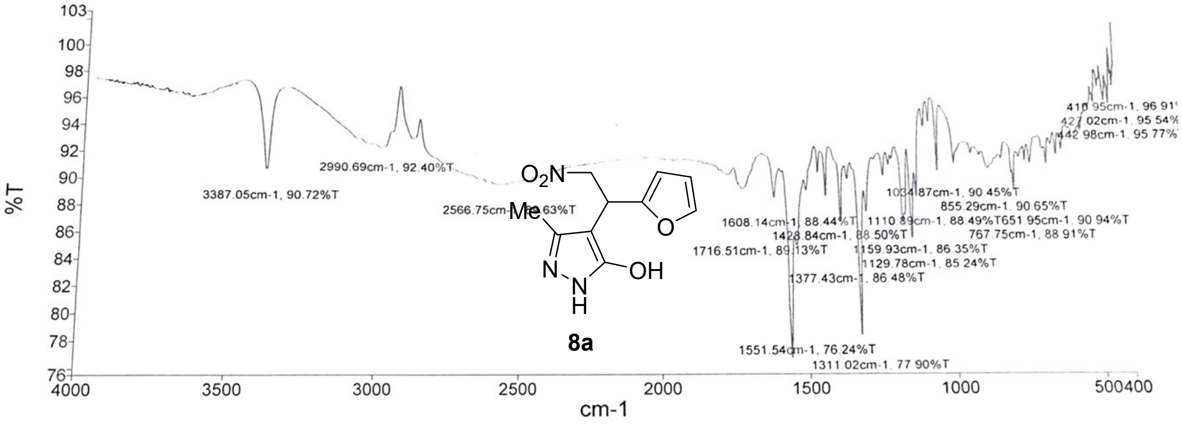

8a

Sample Name	Description	Quality Checks
FURFUIRAL HH_1	Sample 007 By Administrator Date Monday, April 09 2018	The Quality Checks give rise to a Weak Bands warning for the sample.

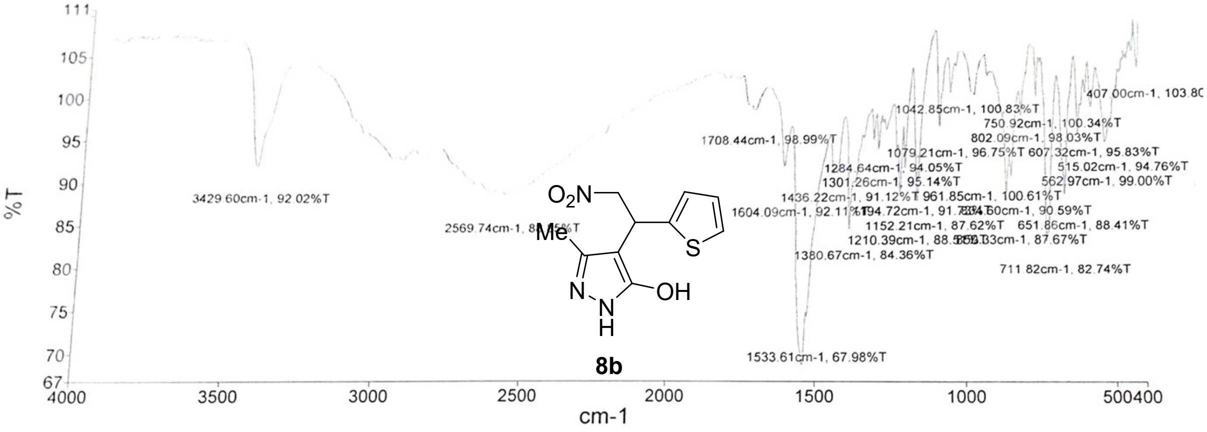

8b

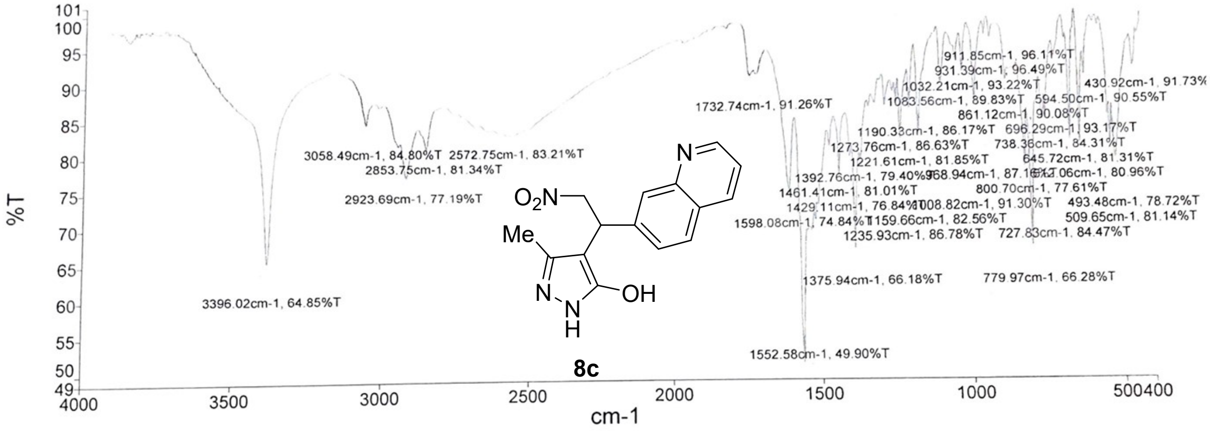

8c

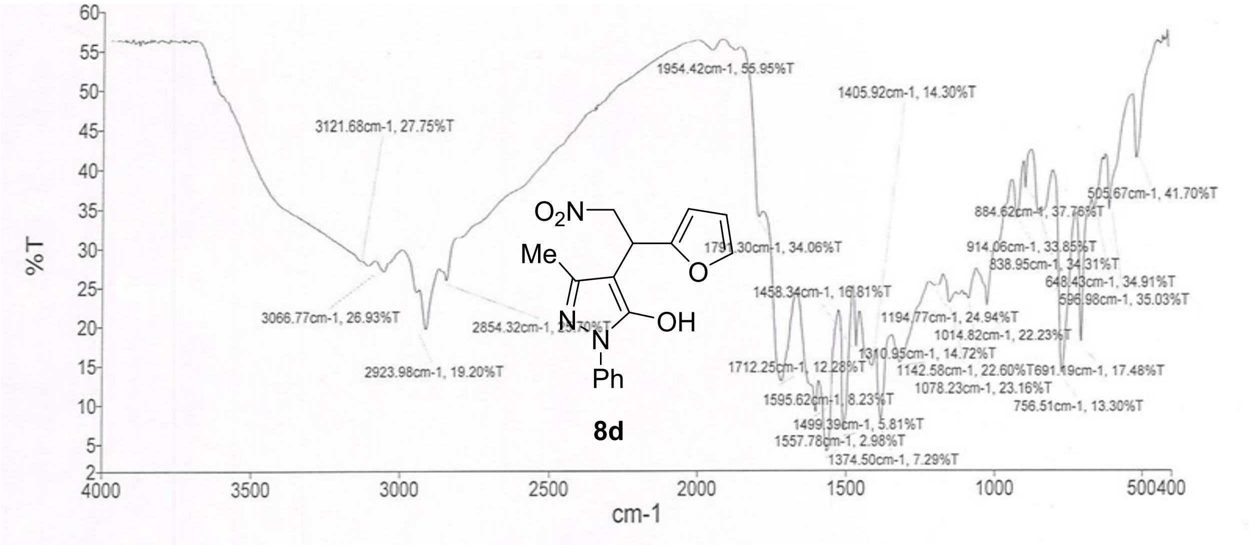
%T
cm-1
3121.68cm-1, 27.75%T
3066.77cm-1, 26.93%T
2923.98cm-1, 19.20%T
1954.42cm-1, 55.95%T
1405.92cm-1, 14.30%T
1458.34cm-1, 16.81%T
1712.25cm-1, 12.28%T
1595.62cm-1, 8.23%T
1499.39cm-1, 5.81%T
1557.78cm-1, 2.98%T
1374.50cm-1, 7.29%T
1310.95cm-1, 14.72%T
1194.77cm-1, 24.94%T
1142.58cm-1, 22.60%T
1078.23cm-1, 23.16%T
1014.82cm-1, 22.23%T
914.06cm-1, 33.85%T
884.62cm-1, 37.78%T
838.95cm-1, 34.31%T
756.51cm-1, 13.30%T
691.19cm-1, 17.48%T
648.43cm-1, 34.91%T
596.98cm-1, 35.03%T
505.67cm-1, 41.70%T
O_2N
Me
N
N
OH
O
Ph
8d

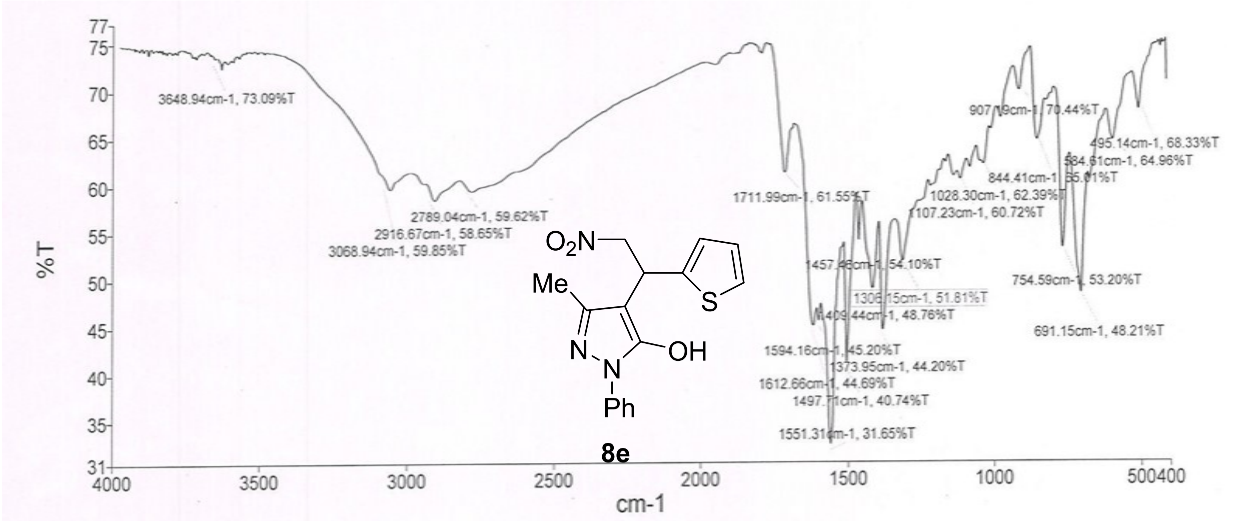
%T
cm-1
3648.94cm-1, 73.09%T
2789.04cm-1, 59.62%T
2916.67cm-1, 58.65%T
3068.94cm-1, 59.85%T
1711.99cm-1, 61.55%T
1457.46cm-1, 54.10%T
1306.15cm-1, 51.81%T
1409.44cm-1, 48.76%T
1594.16cm-1, 45.20%T
1373.95cm-1, 44.20%T
1612.66cm-1, 44.69%T
1497.71cm-1, 40.74%T
1551.31cm-1, 31.65%T
1107.23cm-1, 60.72%T
1028.30cm-1, 62.39%T
907.19cm-1, 70.44%T
844.41cm-1, 65.01%T
754.59cm-1, 53.20%T
691.15cm-1, 48.21%T
584.61cm-1, 64.96%T
495.14cm-1, 68.33%T
O_2N
Me
N
N
OH
S
Ph
8e

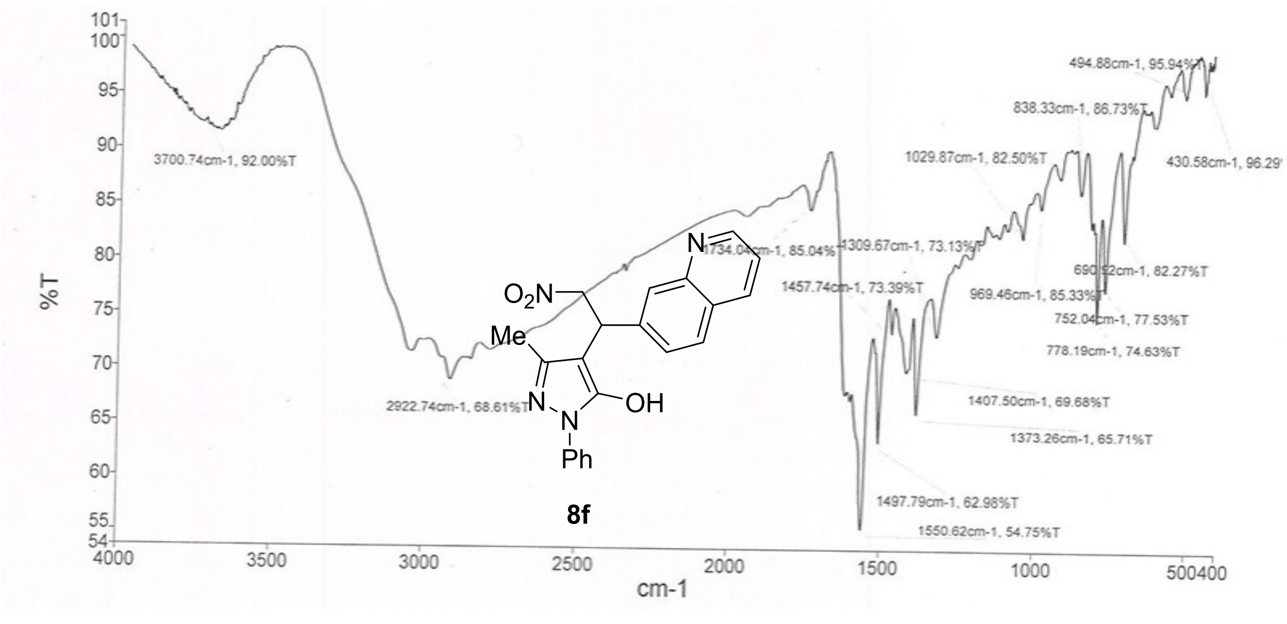
%T
cm-1
3700.74cm-1, 92.00%T
2922.74cm-1, 68.61%T
1734.04cm-1, 85.04%T
1309.67cm-1, 73.13%T
1457.74cm-1, 73.39%T
1407.50cm-1, 69.68%T
1373.26cm-1, 65.71%T
1497.79cm-1, 62.98%T
1550.62cm-1, 54.75%T
1029.87cm-1, 82.50%T
969.46cm-1, 85.33%T
838.33cm-1, 86.73%T
690.52cm-1, 82.27%T
752.04cm-1, 77.53%T
778.19cm-1, 74.63%T
494.88cm-1, 95.94%T
430.58cm-1, 96.29
O_2N
Me
N
N
N
OH
Ph
8f
